# Catalyst control of selectivity in the C–O bond alumination of biomass derived furans†

**DOI:** 10.1039/d0sc01918f

**Published:** 2020-07-08

**Authors:** Thomas N. Hooper, Ryan K. Brown, Feriel Rekhroukh, Martí Garçon, Andrew J. P. White, Paulo J. Costa, Mark R. Crimmin

**Affiliations:** Department of Chemistry, Molecular Sciences Research Hub, Imperial College London 80 Wood Lane, Shepherds Bush London W12 0BZ UK m.crimmin@imperial.ac.uk; BioISI – Biosystems & Integrative Sciences Institute, Faculty of Sciences, University of Lisboa 1749-016 Lisboa Portugal

## Abstract

Non-catalysed and catalysed reactions of aluminium reagents with furans, dihydrofurans and dihydropyrans were investigated and lead to ring-expanded products due to the insertion of the aluminium reagent into a C–O bond of the heterocycle. Specifically, the reaction of [{(ArNCMe)_2_CH}Al] (Ar = 2,6-di-iso-propylphenyl, **1**) with furans proceeded between 25 and 80 °C leading to dearomatised products due to the net transformation of a sp^2^ C–O bond into a sp^2^ C–Al bond. The kinetics of the reaction of **1** with furan were found to be 1st order with respect to **1** with activation parameters Δ*H*^‡^ = +19.7 (±2.7) kcal mol^−1^, Δ*S*^‡^ = −18.8 (±7.8) cal K^−1^ mol^−1^ and Δ*G*^‡^_298 K_ = +25.3 (±0.5) kcal mol^−1^ and a KIE of 1.0 ± 0.1. DFT calculations support a stepwise mechanism involving an initial (4 + 1) cycloaddition of **1** with furan to form a bicyclic intermediate that rearranges by an α-migration. The selectivity of ring-expansion is influenced by factors that weaken the sp^2^ C–O bond through population of the σ*-orbital. Inclusion of [Pd(PCy_3_)_2_] as a catalyst in these reactions results in expansion of the substrate scope to include 2,3-dihydrofurans and 3,4-dihydropyrans and improves selectivity. Under catalysed conditions, the C–O bond that breaks is that adjacent to the sp^2^C–H bond. The aluminium(iii) dihydride reagent [{(MesNCMe)_2_CH}AlH_2_] (Mes = 2,4,6-trimethylphenyl, **2**) can also be used under catalytic conditions to effect a dehydrogenative ring-expansion of furans. Further mechanistic analysis shows that C–O bond functionalisation occurs *via* an initial C–H bond alumination. Kinetic products can be isolated that are derived from installation of the aluminium reagent at the 2-position of the heterocycle. C–H alumination occurs with a KIE of 4.8 ± 0.3 consistent with a turnover limiting step involving oxidative addition of the C–H bond to the palladium catalyst. Isomerisation of the kinetic C–H aluminated product to the thermodynamic C–O ring expansion product is an intramolecular process that is again catalysed by [Pd(PCy_3_)_2_]. DFT calculations suggest that the key C–O bond breaking step involves attack of an aluminium based metalloligand on the 2-palladated heterocycle. The new methodology has been applied to important platform chemicals from biomass.

## Abstract 2

Non-catalysed and catalysed reactions of aluminium reagents with furans, dihydrofurans and dihydropyrans were investigated and lead to ring-expanded products due to the insertion of the aluminium reagent into a C–O bond of the heterocycle.

## Graphical abstract

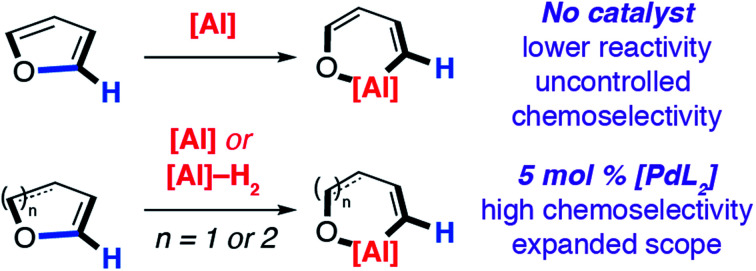

## Introduction

There is increasing interest in upgrading platform chemicals from biomass.^1,2^ The future chemical industry will likely be based on the biorefinery concept.^3,4^ As such, new types of reactions will need to be developed to accommodate the change of feedstock from crude oil to lignocellulosic biomass. These reactions will inevitably involve the chemical manipulation of molecules that contain a high composition of elemental oxygen. While C–H functionalisation reactions have long been important for upgrading petrochemicals,^5,6^ the abundance of C–O bonds in molecules from biomass means C–O functionalisation will be crucial for the valorisation of these substrates. Vital to the long-term success of this venture is achieving a deep understanding of the mechanisms involved in C–O bond breaking. This information will likely transcend individual catalysts and catalytic reactions and form a keystone for developments in the field.

Furan and substituted furans are available from renewable sources, namely the dehydration and aromatisation of sugars.^7^ Furfuraldehyde has been identified as one of the most important platform chemicals from biomass and its global production is estimated at 280 kt per year.^8^ Partial or complete hydrogenation of furans leads to dihydrofurans and tetrahydrofurans respectively. Despite the growth in the applications of furan and its derivatives, the majority of downstream chemistry involves molecules in which the heterocyclic ring remains intact. For example, 2-methyltetrahydrofuran is being applied as a green solvent,^9^ while furfuraldehyde derived building blocks are being using as monomers in the production of renewable polymers.^10^ Although the complete hydrogenation of furans to produce hydrocarbons is being investigated for the replacement of petrochemical fuels,^11^ arguably more value can be derived from retaining or modifying the heterocycle.

There are clear challenges with developing catalytic reactions that ring-open furans to produce useful chemical building blocks. These include overcoming the energetic penalty associated with dearomatisation along with controlling the selectivity of the reaction. There are two major issues of selectivity to overcome. The first is the chemoselectivity for C–O over C–H bonds; the 2-position of these heterocycles are reactive toward C–H functionalisation. The second is the selectivity over which C–O bond breaks in asymmetric heterocycles.

**Fig. 1:**
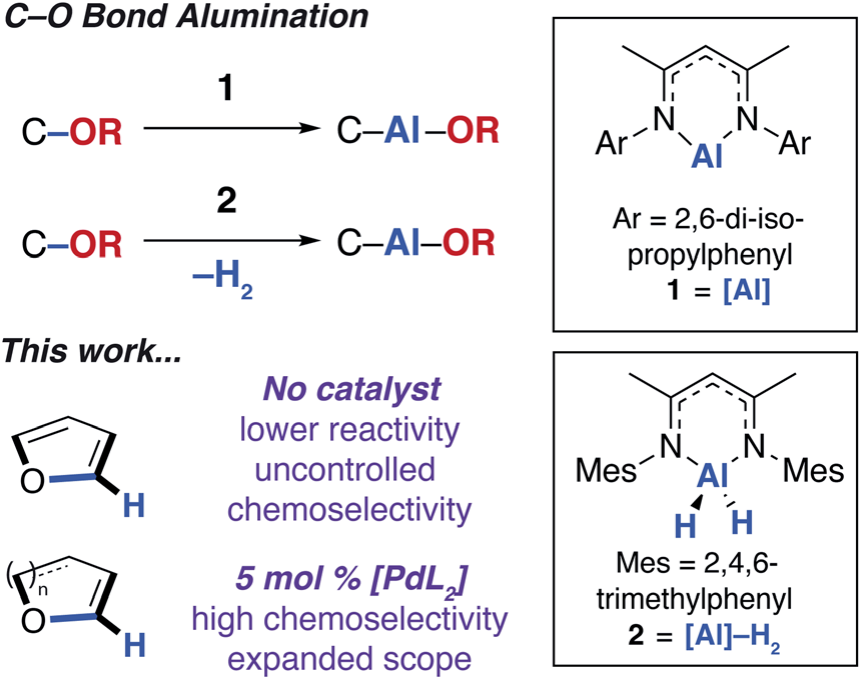
C–O bond alumination of furans.

In 2015, our group^12^ and others^13^ showed that the ring-expansion of benzofuran and tetrahydrofuran could be achieved using a low-valent aluminium(i) reagent (**1**, Fig. 1).^14^ A related study on the unusual ring-expansion of an oxazol-2-ylidene ligand by **1** was reported in 2016.^15^ Our study was inspired by a catalytic protocol we disclosed two years earlier. In 2013, we demonstrated that aluminium(iii) hydride reagents (**2**, Fig. 1) were capable of the dearomatisation and ring-expansion of benzofuran using a zirconocene catalyst and *t*-butylethylene as a hydrogen-acceptor.^16^ We later showed that the efficiency of this reaction could be improved using [Pd(PCy_3_)_2_] in the place of the zirconium catalyst. In both the non-catalysed and catalysed processes, the aluminium reagent (either **1** or **2**) inserts selectively into one of the sp^2^ C–O bonds of the 5-membered ring of benzofuran.^17^ For the palladium catalyst, a mechanism was proposed involving an apparent C–H functionalisation of the 2-position of benzofuran.

A number of related catalytic protocols have been developed for the C–O functionalisation of benzofurans. These include, nickel catalysed ring-expansion borylation reported in 2016,^18,19^ and copper catalysed ring-opening silylation reported in 2018.^20^ Silyl lithium reagents react directly with benzofuran to effect the dearomatisation and ring-opening.^21^ Despite these elegant advances, to date the reaction scope of the catalytic ring-expansion chemistry has not been extended to include furans, arguably a more important target due to their availability from biomass. There are scattered reports in which 2-substituted furans can act as precursors to dearomatised products. Specifically, 2-borylated or 2-silylated furans can undergo ring-expansion reactions to form 6-membered heterocycles which incorporate the boron or silicon atom by photolysis or flash vacuum photolysis respectively.^22,23^

In this paper, we report the C–O functionalisation, dearomatisation and ring-expansion of a broad range of furans along with 2,3-dihydrofuran and 3,4-dihydro-2*H*-pyran. The reaction scope includes derivatives of furfuraldehyde. Remarkably, we found that reactions can occur in both the absence and presence of a catalyst, [Pd(PCy_3_)_2_]. There are clear differences in reaction rates, scope and selectivity between catalysed and non-catalysed routes. These differences can be traced to changes in the mechanism of C–O bond functionalisation which have been interrogated through a combination of kinetics and DFT calculations. The new methodology allows precise control over which C–O bond breaks and holds promise as a means to upgrade and synthetically diversify platform chemicals from biomass.

## Results and discussion

### C–O bond alumination and dearomatisation of furans

**Fig. 2:**
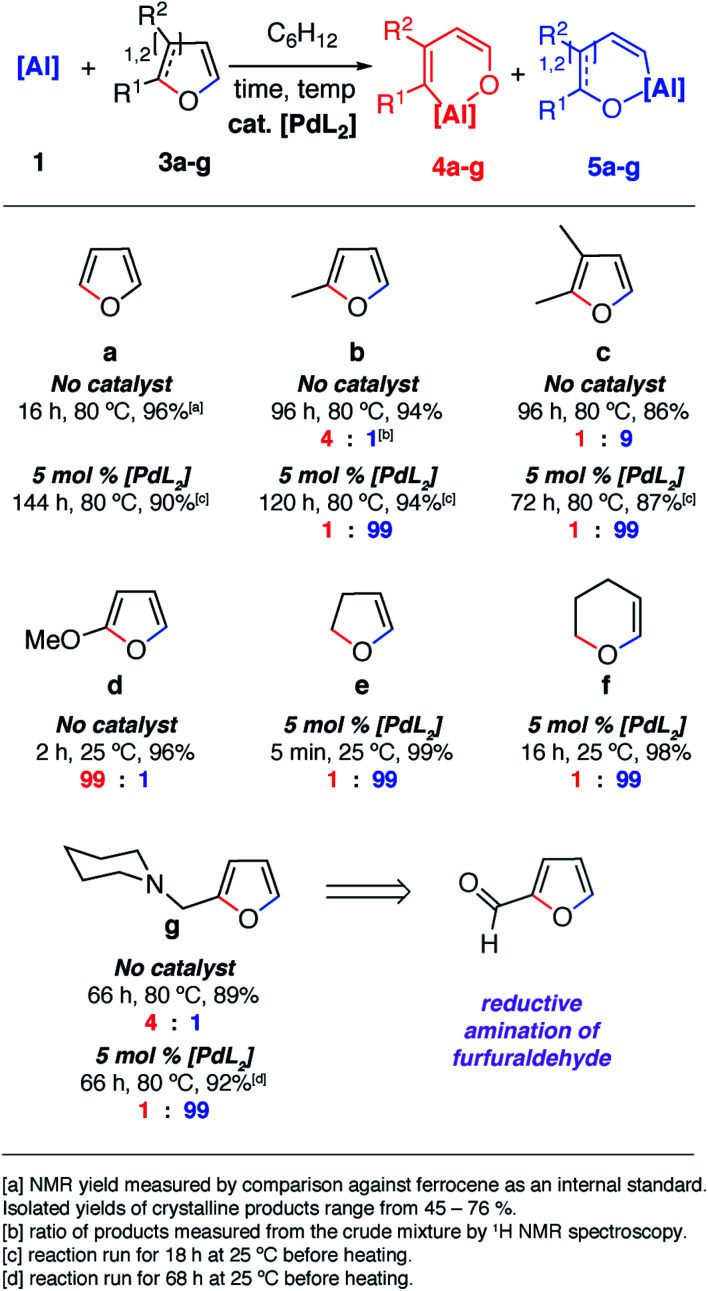
Scope and selectivity of the ring-expansion of furans with **1** under both catalysed and non-catalysed conditions.

**Fig. 3:**
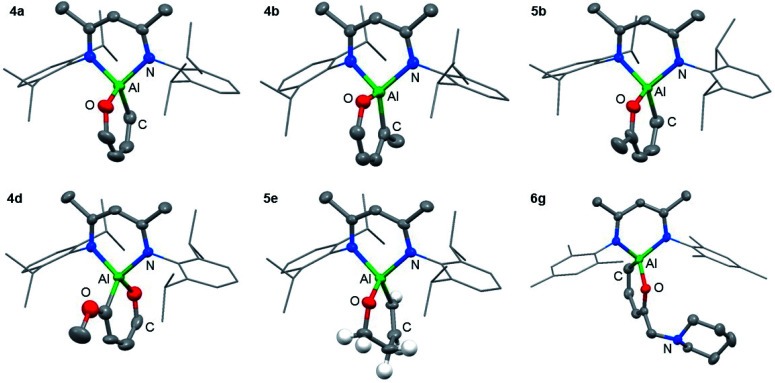
Structures of **4a**, **4b**, **4d**, **5b**, **5e** and **6g** determined by single crystal X-ray diffraction studies. Selected hydrogen atoms and components of disorder omitted for clarity. 50% probability ellipsoids.

The reaction of **1** with furans leads to ring-expansion products under mild conditions. For example, the reaction of **1** with 2 equiv. of furan (**3a**) in cyclohexane at 80 °C resulted in insertion of **1** into the sp^2^ C–O bond and formation of an aluminated cyclic product **4a**. When 2-methylfuran (**3b**) was used, a mixture of isomers was observed with the major product **4b** resulting from the alumination of the more hindered sp^2^ C–O bond, adjacent to the methyl group. The minor product **5b** derives from reaction of the less hindered sp^2^ C–O bond. **4b** could be isolated and purified by recrystallisation. If 2,3-dimethylfuran (**3c**) was used, the selectivity switched to alumination of the less hindered sp^2^ C–O bond (**5c**). Inclusion of an electron donating methoxy group in the 2-position resulted in the reaction occurring at 25 °C with complete selectivity for the most hindered site and formation of **4d**. The products were isolated and fully characterised. Single crystal X-ray diffraction experiments unambiguously confirmed the assignment of the isomers (Fig. 2 and 3).

Notable changes in selectivity and reaction times were observed when conducting the same reactions in the presence of 5 mol% [Pd(PCy_3_)_2_]. In all cases, C–O bond alumination occurs exclusively at reaction sites adjacent to a C–H bond and the least hindered site of the furan. We have previously reported the isolation of C–H functionalised intermediates in the C–O bond alumination of benzofuran.^17^ Metallation at the 2-position provides a kinetic product which, under more forcing conditions, converts to the thermodynamic ring-expansion product. Catalyst control not only results in higher and complementary selectivity in the ring-expansion of asymmetric furans when compared to the non-catalysed reaction (Fig. 2b and g), it also allows expansion of the reaction scope to include 2,3-dihydrofuran and 3,4-dihydro-2*H*-pyran (Fig. 2e and f). These latter substrates do not lead to tractable products in the absence of a catalyst. **2g** was prepared by the reductive amination of furfuraldehyde, providing a direct application of the methodology to biomass-derived substrates.

**Fig. 4:**
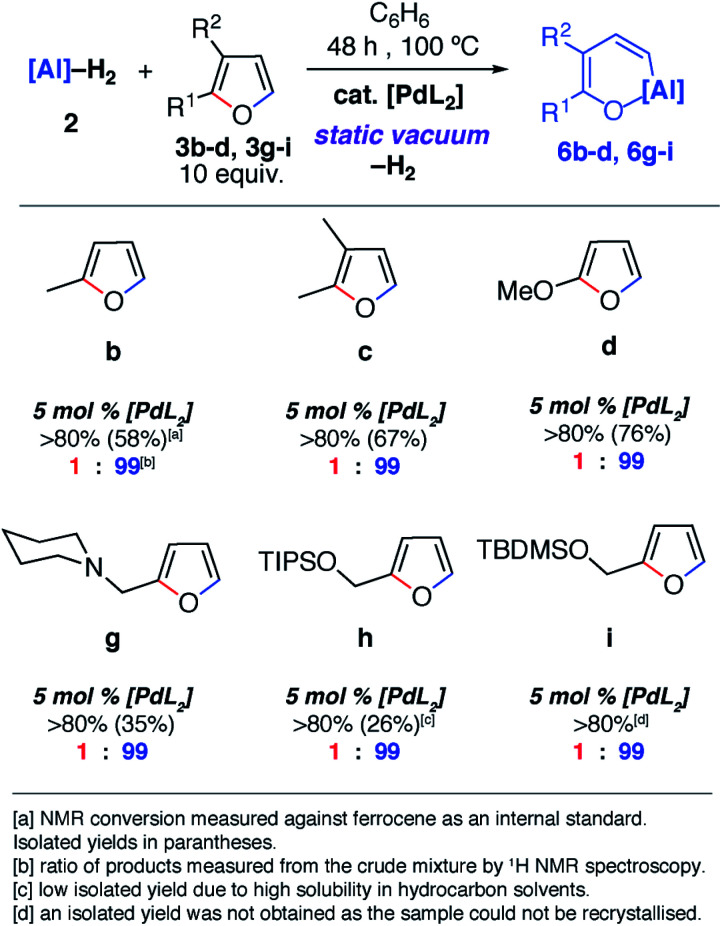
Scope and selectivity of the ring-expansion of furans with **2** under catalysed conditions.

Despite the noteworthy reactivity of **1** with furans, broad applications of this methodology are complicated by the challenging synthesis of the aluminium(i) reagent. The use of the aluminium(iii) dihydride **2** would allow preparations to be scaled up significantly when compared to using **1**. **2** is readily available on a multi-gram scale from LiAlH_4_.^24^ The scope of the palladium-catalysed C–O bond alumination of furans was expanded to include the aluminium dihydride **2**. The removal of H_2_ from the mixture is essential for high conversions and this can be achieved by running reactions in a sealed vessel under a static vacuum. Heating **2** with 10 equiv. of the furan derivatives and 5 mol% of [Pd(PCy_3_)_2_] in a benzene solution under static vacuum results in the formation of the alumination products **6b–d** and **6g–i** (Fig. 3 and 4). The selectivity of these reactions mirror those from the palladium catalysed reactions from **1**. Although the unoptimised yields are modest due to isolation of the products by recrystallisation, the high conversions make this reaction a promising way to form reactive building blocks from furans. Notably the substrate scope includes a number of biomass derived building blocks from furfuraldehyde and furfuryl alcohol. Under these conditions, furan itself did not react cleanly with **2**.

### Mechanism and origin of selectivity for ring-expansion of furans

A combination of kinetic experiments (KIEs, Eyring analysis) and calculations (DFT) were used to gain insight into the mechanisms of C–O bond functionalisation under both non-catalysed and catalysed conditions. The approach was used to elucidate the origin of selectivity.

#### Non-catalysed conditions

**Fig. 5:**
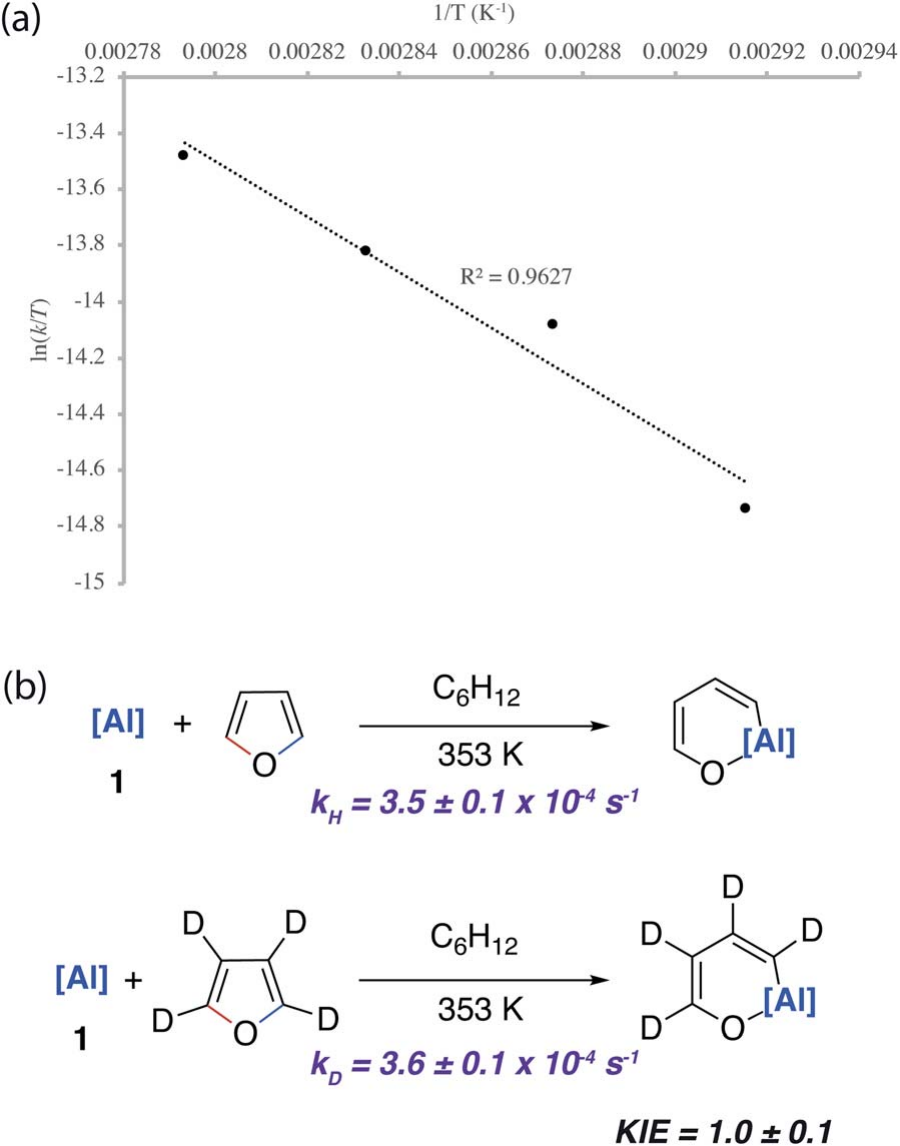
(a) Eyring analysis and (b) KIE for the reaction of **1** with furan and d_4_-furan.

The reaction of **1** with furan under pseudo-first order conditions in cyclohexane solvent was followed as a function of time by ^1^H NMR spectroscopy and reaction rates determined. Kinetics followed the expected 1^st^ order behaviour over the entire timeframe of the measurement (3 half-lives). An Eyring analysis of the reaction gave thermodynamic parameters of Δ*H*^‡^ = +19.7 (±2.7) kcal mol^−1^, Δ*S*^‡^ = −18.8 (±7.8) cal K^−1^ mol^−1^ and Δ*G*^‡^_298 K_ = +25.3 (±0.5) kcal mol^−1^ (Fig. 5a). A KIE of 1.0 ± 0.1 was measured by independent measurement of the rate constants for reaction of **1** with furan and d_4_-furan. The KIE shows that the breaking of a C–H bond is unlikely to be involved in the rate-limiting step. While the KIE is very close to 1, based on the error of the measurement, we cannot rule out either normal or inverse secondary KIE resulting from a rehybridisation of sp^3^ to sp^2^ C–H bonds (or *vice versa*) during the rate-limiting step (Fig. 5b).

**Fig. 6:**
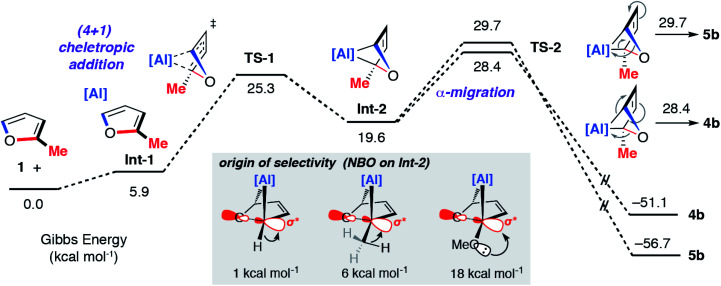
Calculated pathway for the reaction of **1** with 2-methylfuran. Inset shows the results of NBO calculations on **Int-2** specifically donor–acceptor interactions from 2^nd^ order perturbation analysis.

The concerted oxidative addition of the C–O bond of furan to **1** has been modelled by DFT calculations by others prior to our experimental work.^25^ The fidelity of the study is such that we cannot at this time make a clear comparison against our mechanistic analysis. Our DFT calculations support a stepwise mechanism. The reaction between **1** and 2-methylfuran proceeds by an initial (4 + 1) cycloaddition *via***TS-1** to form a bicyclic intermediate **Int-2**. We have recently reported the cheletropic reaction of **1** with dienes and aromatic hydrocarbons.^26^ Based on analysis of the NPA charges, the Wiberg bond indices and the bond angles and lengths from Al to the β-diketiminate ligand, this step involves a redox transformation of Al(i) to Al(iii) and the dearomatisation of 2-methylfuran. The (4 + 1) intermediate rearranges *via* two possible isomeric transition states (**TS-2**) to form the isomers of the product (**4b**/**5b**) (Fig. 6). An analogous pathway was determined for furan (see ESI†) and the computationally determined activation barrier for the α-migration step (Δ*G*^‡^ = +27.0 kcal mol^−1^) matches well with that determined by the Eyring analysis (Δ*G*^‡^ = +25.3 ± 0.5 kcal mol^−1^).

The selectivity determining step is the framework rearrangement *via***TS-2**. **TS-2** involves a rearrangement of a [2.1.1] aluminoxabicyclohexane. This key step can be conceptualised as an α-migration of the C–O σ-bond that is adjacent to the aluminium centre. Considering **Int-2**, migratory aptitude determines the selectivity: this equates to which of the two C–O σ-bonds breaks. In general, electron donating substituents favour C–O σ-bond breaking (MeO > Me > H). This origin of selectivity can be traced to these substituents weakening the C–O σ-bond through population of the C–O σ* orbital through hyperconjugation or anomeric effects. Consistent with this argument DFT calculations predict the experimentally observed selectivity for 2-methylfuran and 2-methoxyfuran. The formation of **4b** is calculated to be slightly more favourable than **5b** (ΔΔ*G*^‡^ = +1.3 kcal mol^−1^) but **4d** is considerably more favourable than **5d** (ΔΔ*G*^‡^ = +9.4 kcal mol^−1^).

Further DFT calculations were undertaken to understand the effects of steric in these reactions and the role substitution at the 3-position plays in determining selectivity. Experimentally, it was found that 2,3-dimethylfuran reacts with **1** with a 9 : 1 ratio for C–O bond functionalisation of the least sterically hindered position (Fig. 2c). Hence, introduction of a substituent at the 3-position appears to override the electronic factors described above. DFT calculations on the addition of **1** to 2,3-dimethylfuran did not allow an accurate prediction of this selectivity (Fig. S6.6†). Further calculations on the reaction of **1** with 3-methylfuran allowed steric and electronic effects to be deconvoluted and now accurately predict the reaction at the least sterically hindered site (Fig. S6.7†).

#### Catalysed conditions

**Fig. 7:**
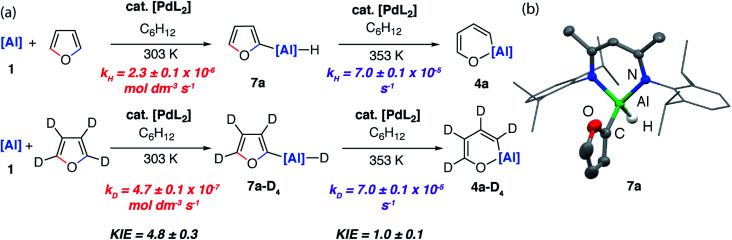
(a) Isolation of C–H aluminated intermediate and KIEs for the palladium-catalysed reaction of **1** with furan and d_4_-furan. (b) Structure of **7a** determined by single crystal X-ray diffraction study, ellipsoids at 50% probability, selected hydrogen atoms omitted for clarity.

The palladium-catalysed reaction occurs by a completely different mechanism. Experimentally, it was determined that the formation of **4a** from furan and **1** proceeds *via* the C–H aluminated product **7a** which formed in 24 h at 25 °C (Fig. 7).

If the reaction mixture was heated to 80 °C the C–O aluminated product **4a** could be formed cleanly from **7a**. **7a** has been isolated and fully characterised. Re-exposing **7a** to the reaction conditions results in its catalytic isomerisation to **4a**. This reaction does not proceed in the absence of a catalyst. Similarly, for 2-methylfuran (**3b**) and 2,3-dimethylfuran (**3c**) the corresponding C–H alumination products **7b** and **7c** could be identified by ^1^H NMR spectroscopy at early time points in the reaction. For example, **7b** forms as a mixture with **5b** after heating reaction mixtures of **1** and 2-methylfuran to 40 °C for 72 h. Increasing the temperature to 80 °C for 120 h resulted in full conversion to **5b** (see ESI for details†). From monitoring these reactions as a function of time it is clear that C–H functionalisation occurs *en route* to C–O functionalisation and dearomatisation.

Kinetics experiments were used to compare the palladium-catalysed reactions of **1** with furan and d_4_-furan to form the C–H aluminated product **7a** and **7a-D4** respectively. The reaction has a strong primary KIE of 4.8 ± 0.3 at 303 K (Fig. 7a). A similar large primary KIE of 5.8 ± 0.1 was measured for the C–H alumination of benzene with the same catalyst at the same temperature.^27^ The KIE for the second step of the C–O alumination reaction using [Pd(PCy_3_)_2_] was measured at 353 K, again in independent experiments, using isolated samples of **7a** and **7a-D4** and was found to be 1.0 ± 0.1 (Fig. 7a). Hence, the C–H bond breaks during the turnover-limiting step of the catalytic pathway for the C–H functionalisation but not the C–O bond functionalisation.

**Fig. 8:**
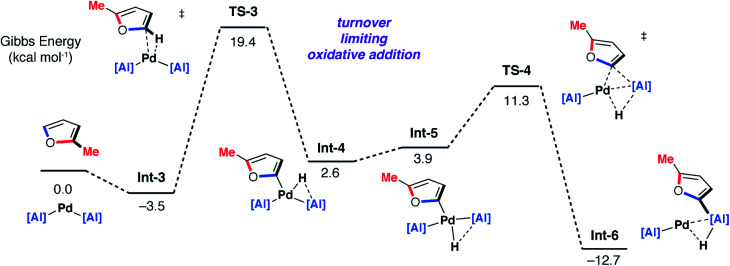
DFT calculated pathway for the palladium-catalysed C–H alumination reaction of **1** with 2-methylfuran.

**Fig. 9:**
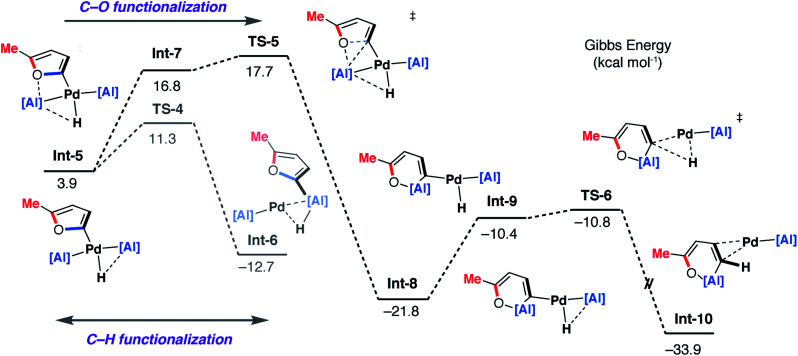
DFT calculated pathway for the palladium-catalysed C–O alumination reaction involving isomerisation of **7b**.

DFT calculations on the mechanism of the palladium-catalysed reaction of **1** with 2-methylfuran support a stepwise C–H then C–O alumination pathway (Fig. 8 and 9). We have previously shown that for the C–H alumination of arenes with **1**, the active catalytic species is likely a two-coordinate palladium complex of the form [Pd(**1**)_2_] which derives from phosphine dissociation from [Pd(**1**)_2_(PCy_3_)]. The latter complex forms on reaction of **1** and [Pd(PCy_3_)_2_] has been isolated and crystallographically characterised.^27^ The same assumption is made here. This active palladium species forms a π-complex with 2-methylfuran to form **Int-3** and then proceeds through **TS-3** (Δ*G*^‡^ = +22.9 kcal mol^−1^) to oxidatively add the C–H bond at the 5-position of 2-methylfuran to the Pd centre. This is the highest barrier in the C–H alumination reaction sequence. The calculated pathway is consistent with the large primary KIE observed experimentally for the palladium catalysed reactions of furan and d_4_-furan with **1**. **TS-3** is almost entirely palladium-centered. **Int-4** forms directly from this oxidative addition and can undergo a *cis*/*trans* isomerisation to give **Int-5**. At this point, the C–H aluminated product can be formed *via* a double-migration^28^ of the hydride and 2-methylfuryl ligands through **TS-4** (local barrier, Δ*G*^‡^ = +8.7 kcal mol^−1^) from the Pd to Al centre to form **Int-6**. The calculations predict that this is the lowest energy pathway from this intermediate. **Int-6** contains the kinetic C–H aluminated product **7b** bound to Pd.

Alternatively, **Int-5** can undergo ring-expansion chemistry by a higher energy pathway that leads to the thermodynamic product **5b**. Coordination of the oxygen lone pair of the 2-methylfuryl ligand to the Al centre in **Int-5** leads to **Int-7**. This intermediate can then undergo an addition of the C–O bond to the Al centre by **TS-5**. The Pd–C bond is not broken in this step. The difference in energy between **TS-4** and **TS-5** (ΔΔ*G*^‡^ = 6.4 kcal mol^−1^) determines the selectivity for the kinetic C–H alumination product. A subsequent reductive elimination step from **Int-8***via* formation of the unstable intermediate **Int-9** and then **TS-6** forms **Int-10** which is a weakly bound palladium complex of the observed product **5b**. The isomer **4b** is not accessible by this mechanism. This pathway explains the high selectivity of palladium catalysis, essentially the breaking of the C–H bond of the 2-position of the heterocycle in the reaction pathway results in precise control over which C–O bond reacts. The mechanism also explains why, for the reaction of furan with **1**, isolated samples of **7a** can isomerise to **4a**. **7a** is the kinetic product while **4a** is the thermodynamic product. Isomerisation of **7a** to **4a** can occur *via***Int-5** and is initiated by the microscopic reverse of the double migration step.

The lack of a KIE in the isomerisation of **7a** → **4a** recorded during the reactions of furan as a substrate (Fig. 5a) is consistent with the DFT calculations on 2-methylfuran. The calculations suggest that catalyst turnover for the isomerisation of the C–H to C–O functionalised product is determined by the energy span between **Int-6** and **TS-5**. These steps involve a hydride ligand migrating between Al and Pd but not reformation of the C–H bond. The reductive elimination and reformation of the C–H bond occurs after the turnover limiting steps. The intramolecular nature of the isomerisation of **7a** → **4a** is supported by cross-over experiments (see ESI for details†).

## Conclusions

In summary, we have shown that the monomeric aluminium(i) reagent **1** can react with substituted furans to form ring-expanded C–O aluminated products. This reaction involves the dearomatisation of the furan ring and the transformation of a sp^2^ C–O bond into a sp^2^ C–Al bond. Analysis of the mechanism by kinetics and DFT calculations provides evidence for a stepwise mechanism involving an initial (4 + 1) cycloaddition of **1** with the furan to form a bicyclic intermediate which then undergoes an α-migration event to break the strong C–O bond. The selectivity of which C–O bond breaks is driven primarily by electronic factors. Weakening of the C–O σ-bond through population of the σ*-orbital leads to lower energy transition states for ring-expansion. These electronic effects can apparently be overridden by steric effects when the heterocycle is substituted at the 3-position.

Running the same reactions in the presence of catalytic quantities of [Pd(PCy_3_)_2_] not only leads to higher and complementary selectivity, it allows expansion of the substrate scope beyond furans to 2,3-dihydrofuran and 3,4-dihydropyran. These latter two substrates do not lead to tractable products under non-catalysed conditions. Using catalysis, the aluminium(iii) dihydride **2** can also be used as a reagent in furan ring expansion leading to a dehydrogenative protocol that can be applied on modest scales due to the ease of synthesis of **2**. Through a combination of kinetics and DFT calculations, we propose that the catalytic mechanism occurs by a stepwise process involving the formation of kinetic C–H alumination products which can undergo a subsequent intramolecular catalytic isomerisation to thermodynamic C–O alumination products. This mechanism results in a strict control over the selectivity of which C–O bond breaks, as it is always the site adjacent to a C–H bond.

The new reactions we report have potential in upgrading furans, dihydrofurans and dihydropyrans from biomass and the methods have been applied to derivatives of furfuraldehyde. Moreover, the mechanistic information gained from this study provides new insight in how to break strong C–O bonds with main group reagents and transition metal catalysts.

## Author contributions

TNH and RKB conducted the experimental work. FR and RKB conducted calculations on the Pd-catalysed system, RKB and MG conducted calculations on the non-catalysed system. AJPW and TNH collected and analysed single crystal X-ray diffraction data. PJC and MRC managed the project. The manuscript was written through contributions of all authors. All authors have given approval to the final version of the manuscript.

## Conflicts of interest

There are no conflicts to declare.

## Supplementary Material

SC-011-D0SC01918F-s001

SC-011-D0SC01918F-s002

SC-011-D0SC01918F-s003
