# Supplementary material for: Catalyst control of selectivity in the C–O bond alumination of biomass derived furans
Source: Chem Sci. 2020 Jul 8;11(30):7850–7. doi: 10.1039/d0sc01918f (PMC8163288; doi:10.1039/d0sc01918f)
Supplement: SC-011-D0SC01918F-s002 [file SC-011-D0SC01918F-s002.pdf]

Electronic Supplementary Material (ESI) for Chemical Science.
This journal is © The Royal Society of Chemistry 2020

Electronic Supplementary Information for:

Catalytic Control of Chemoselectivity in the C–O Bond Alumination of Biomass Derived Furans

Thomas N. Hooper,[a] Ryan K. Brown,[a] Feriel Rekhroukh,[b] Martí Garçon,[a] Andrew J. P. White[a], Paulo J. Costa,[b] and Mark R. Crimmin*,[a]

a) *Department of Chemistry, Molecular Sciences Research Hub, Imperial College London, 80 Wood Lane, Shepherds Bush, London, W12 0BZ, UK.*

b) *BioISI – Biosystems & Integrative Sciences Institute, Faculty of Sciences, University of Lisboa, 1749-016 Lisboa, Portugal.*

**Corresponding author. E-mail: m.crimmin@imperial.ac.uk*

Table of contents

1	General Experimental	S3
2	Experimental Results	S4
3	X-ray Crystallographic Data	S25
4	Kinetic Studies by NMR Spectroscopy	S40
5	Crossover Experiments	S48
6	Computational Details	S51

1. General Experimental, Preparation of Materials and Instruments

1.1 Materials

Dipp-*BDI*Al(I) (**1**) and Mes-*BDI*AlH_2 (**2**) were synthesised by the literature procedures (Ar-*BDI* = $\{(ArNCMe)_2CH\}^-$, Ar = 2,4,6-$Me_3C_6H_2$ (Mes), 2,6-$^iPr_2C_6H_3$ (Dipp)).[1] $[Pd(PCy_3)_2]$ was synthesised as an analytically pure pale brown crystalline solid from $PdCl_2$ in a 3 step procedure (via intermediates $[Pd(\eta^3\text{-}C_3H_4Ph)(\mu\text{-}Cl)]_2$ and $[CpPd(\eta^3\text{-}C_3H_4Ph)]$) based on literature reactions.[2] C–O substrates were purchased from common suppliers (e.g. Sigma-Aldrich, Fluorochem, Merck, Alfa Aesar, TCI etc. unless otherwise stated below), liquid reagents were stored over activated 3 Å molecular sieves, degassed by the freeze-pump-thaw method (x 3) and stored in the glove box; solid reagents were dried under high vacuum and stored in the glove box. All other reagents were purchased from common suppliers and used without further purification.

Solvents for air sensitive procedures (toluene, *n*-hexane) were dried using a solvent purification system (SPS) and stored over activated 3 Å molecular sieves under an inert atmosphere of N_2 or argon before use. C_6H_6 (Sigma-Aldrich anhydrous grade), *n*-heptane, cyclohexane, C_6D_6 and toluene-D_8 were degassed by the freeze-pump-thaw method (x 3) and stored under inert atmosphere over activated 3 Å molecular sieves. Silica gel (technical grade, 230–400 mesh particle size) for column chromatography was purchased from Sigma-Aldrich.

1.2 Instruments

^1H NMR and ^{13}C NMR spectra were recorded and analysed using Bruker 400 MHz Spectrometer at 298 K. The reported values for ^1H and ^{13}C NMR data are as follows: chemical shifts (δ ppm), multiplicity (where s = singlet, d = doublet, t = triplet, m = multiplet), integration (not ^{13}C) and coupling constant, *J* (Hz). AT-IR spectra were recorded on an Agilent Technologies Cary 630 FTIR spectrometer. Elemental analysis was carried out by Stephen Boyer, London Metropolitan University.

[1] (a) S. Yow, S. J. Gates, A. J. P. White, M. R. Crimmin, *Angew. Chem. Int. Ed.,* 2012, **51**, 12559. (b) C. Cui, H.W. Roesky, H.-G. Schmidt, M. Noltemeyer, H. Hao and F. Cimpoesu, *Angew. Chem. Int. Ed.*, 2000, **39**, 4274.

[2] (a) P. R. Auburn, P. B. Mackenzie, B. Bosnich, *J. Am. Chem. Soc.*, 1985, **107**, 2033. (b) S. D. Robinson , B. L. Shaw, *J. Chem. Soc.*, 1963, 4806. (c) T. Yoshida, S. Otsuka, *Inorg. Synth*., 1990, **28**, 114.

2. Experimental Results

2.1 Synthesis of Protected C–O Substrates

2.1.1 Synthesis of 2-($CH_2NC_5H_{10}$)C_4H_3O (3g)

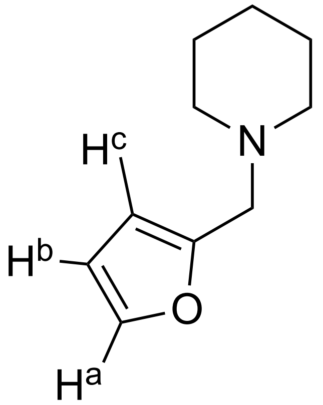

2-($CH_2NC_5H_{10}$)C_4H_3O was synthesised from furfural using standard reductive amination chemistry. Under Ar, furfural (1 mL, 12.1 mmol) was dissolved alongside piperidine (1.2 mL, 12.01 mmol) in MeOH (20 mL) and stirred for 2 h generating a red solution. Na[BH_4] (0.55 g, 14.5 mmol) was added causing an immediate colour change to pale-yellow and the solution was stirred for a further 12 h. Volatiles were removed under reduced pressure to afford a crude yellow oil which was purified by column chromatography (15:1 hexane : ethyl acetate) over silica gel, generating a colourless oil. The product was dried over CaH_2 and distilled prior to use. Yield 0.35 g, 2.12 mmol, (18 % unoptimised yield). The NMR spectra for **3g** have been previously reported in $CDCl_3$[3] and are presented here in C_6D_6 for completeness. **^{1}H NMR** (400 MHz, C_6D_6, 298 K, delay (d1) 20 s): δ 7.14 (dd, $^3J_{HH}$ = 1.8 Hz, $^4J_{HH}$ = 0.9 Hz, 1H, H^a), 6.11 (dd, $^3J_{HH}$ = 3.1 Hz, $^3J_{HH}$ = 1.9 Hz,1H, H^b), 6.07 (appears as dq, $^3J_{HH}$ = 3.1 Hz, $^4J_{HH}$ = 0.7 Hz ,1H, H^c), 3.39 (s, 2H, CH_2), 2.30 (m, 4H), 1.46 (m, 4H), 1.24 (m, 2H) ppm; **^{13}C{^{1}H} NMR:** δ 153.10, 141.47 (s, C–H^a), 109.98 (s, C–H^b), 107.88 (s, C–H^c), 55.64 (s, CH_2), 54.00, 26.05, 24.31.

2.1.2 Synthesis of 2-($CH_2OSi(CH(CH_3)_2)_3$)C_4H_3O (3h)

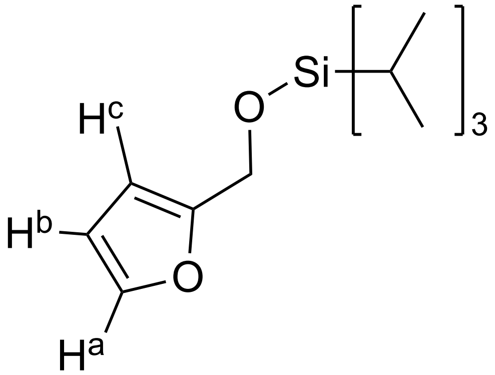

2-($CH_2OSi(CH(CH_3)_2)_3$)C_4H_3O was synthesised following a modified literature procedure.[4] Under Ar, to a stirring 0 °C solution of imidazole (2.77g, 40.8 mmol) in CH_2Cl_2 (30 mL) was added freshly distilled furfuryl alcohol (2.26 mL, 20.4 mmol). To this was added triisopropylchloride (TIPSCl) (4.36 mL, 20.4 mmol) via syringe. The solution was allowed to warm slowly to 25 ºC and stirred overnight. The next day the solution was neutralised with $NaCO_3$ (aq, 20 mL) and the product extracted into CH_2Cl_2 (3 x 5 mL). The washings were then collected and dried over $Mg(SO_4)_2$, filtered and then volatiles were removed under reduced pressure generating a pale yellow oil. The oil was purified by column chromatography (20:1 hexane:CH_2Cl_2) and then dried over CaH_2 and

[3] H. Heaney, G. Papageorgiou, R.F.Wilkins, *Tetrahedron*, 1997, **53**, 2941.
[4] F. Rivas, J. Jin, R. J. Aversa, K. C. Nicolaou, *J. Am. Chem. Soc.*, 2010, **132**, 6855-6861.

vacuum distilled at 50–60 °C to generate a pure colourless oil. Yield: 3.20 g, 12.58 mmol, (62 %) **^{1}H NMR** (400 MHz, C_6D_6, 298 K, delay (d1) 20s): δ 7.10 (dd, $^{3}J_{HH}$ = 1.8 Hz, $^{4}J_{HH}$ = 0.8 Hz, 1H, H^{a}), 6.12 (dd, $^{3}J_{HH}$ = 3.2 Hz, $^{4}J_{HH}$ = 0.7 Hz, 1H, H^{c}), 6.08 (dd, $^{3}J_{HH}$ = 3.2 Hz, $^{3}J_{HH}$ = 1.9 Hz, 1H, H^{b}), 4.60 (s, 2H, CH_2), 1.08 (m, 21H, TIPS) ppm; **^{13}C{^{1}H} NMR** (100.6 MHz, C_6D_6, 298 K): δ 154.69, 141.65 (s, C–H^{a}), 110.04 (s, C–H^{b}), 106.80 (s, C–H^{c}), 58.43, 17.78, 12.01.

2.1.3 Synthesis of 2-($CH_2OSi(CH_3)_2C(CH_3)_3$)C_4H_3O (3i)

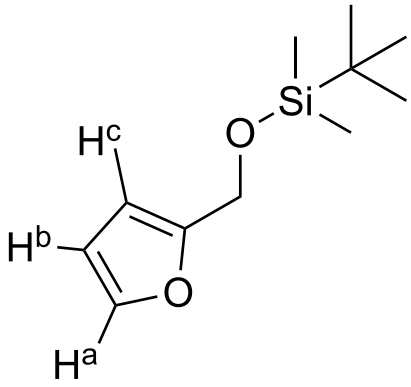

2-($CH_2OSi(CH_3)_2C(CH_3)_3$)C_4H_3O was synthesised using the same method described above, with the modification of using the appropriate silyl chloride reagent, tert-butyldimethylsilylchloride (TBDMSCl) (4.61 g, 30.6 mmol). Product was dried over CaH_2 and purified by fractional distillation under reduced pressure (0.01 mbar, >150 °C) to generate a colourless oil. Yield: 3.04 g, 14.32 mmol, (70 %) .**^{1}H NMR** (400 MHz, C_6D_6, 298 K, delay 20s): δ 7.08 (dd, $^{3}J_{HH}$ = 1.7 Hz, $^{4}J_{HH}$ = 1.0 Hz , 1H, H^{a}), 6.07–6.04 (overlapping signals, 2H, H^{b} and H^{c}), 4.50 (s, 2H, CH_2), 0.95 (s, 9H, ^{t}Bu), 0.04 (s, 6H, Si–CH_3) **^{13}C{^{1}H} NMR** (100.6 MHz, C_6D_6, 298 K): δ 154.52 (s, **C**CH_2OTIPS), 141.78 (s, C–H^{a}), 110.09 (s, C–H^{b}), 107.11 (s, C–H^{c}), 57.93 (s, CH_2), 25.67 (s, C^{tBu}–H_3), 18.15 (s, **C**CH_3), -5.53 (Si-CH_3).

2.2 Aluminated Products from Uncatalysed Insertion into the C–O Bonds of Furan and Derivatives

2.2.1 Synthesis of Dipp-*BDI*Al(OC_4H_4) (4a)

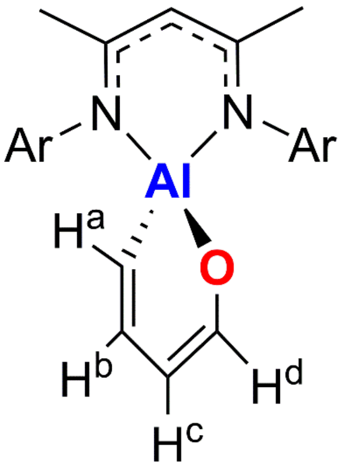

Ar = Dipp

In a glovebox, Dipp-*BDI*Al(I) (**1**, 5.0 mg, 0.0113 mmol) was dissolved in cyclohexane (0.55 mL) and furan (1.6 µL, 0.225 mmol, 2 equiv.) was added by micropipette. The solution was mixed and transferred to a Young's NMR tube, sealed and removed from the glovebox. The tube was heated to 80 °C for 16 hours with monitoring by NMR spectroscopy. Once the reaction was complete (in situ yield 96%) the tube was returned to the glovebox and solution was transferred to a 4 ml vial. The solvent was removed *in vacuo*. The oily product was dissolved in *n*-heptane (0.3 mL) and the mixture stored at -35 °C. Colourless crystals of the product formed, the mother liquor was decanted and the product **4a** dried *in vacuo*. An isolated yield was obtained from a scaled-up reaction using the same conditions and 15.0 mg (0.0338 mmol) of **1**. Isolated yield: 13 mg (75%). **^{1}H NMR** (400 MHz, C_6D_6, 298 K): δ 7.09-7.00 (overlapping m, 7H, Dipp-Ar-H + H^b), 6.93 (dt, $^3J_{HH}$ = 5.7 Hz, $^4J_{HH}$ = 1.2 Hz, 1H, H^d), 5.83 (d, $^3J_{HH}$ = 15.8 Hz, 1H, H^a), 4.95 (overlapping s, 1H, C-H), 4.93 (td, $^3J_{HH}$ = 5.7 Hz, $^3J_{HH}$ = 1.2 Hz, 1H, H^c), 3.51 (sept, $^3J_{HH}$ = 6.8 Hz, 2H, -$CHMe_2$), 3.27 (sept, $^3J_{HH}$ = 6.8 Hz, 2H, -$CHMe_2$), 1.53 (s, 6H, CH_3), 1.38 (overlapping d, $^3J_{HH}$ = 6.8 Hz, 6H, Dipp-CH_3), 1.36 (overlapping d, $^3J_{HH}$ = 6.8 Hz, 6H, Dipp-CH_3), 1.15 (d, $^3J_{HH}$ = 6.8 Hz, 6H, Dipp-CH_3), 1.08 (d, $^3J_{HH}$ = 6.8 Hz, 6H, Dipp-CH_3) ppm; **^{13}C{^{1}H} NMR** (100.6 MHz, C_6D_6, 298 K): δ 169.92, 150.29 (s, C-H^d), 148.04, 144.96, 142.97, 139.33, 127.36, 124.52, 123.60 (s, C-H^b), 104.96 (s, C-H^c), 97.49, 28.76, 28.09, 25.87, 24.42, 24.21, 23.87, 22.90 ppm. The C–Al resonance could not be observed due to line-broadening associated with coupling to the quadrapolar *I* = 5/2 ^{27}Al nucleus. **Elemental analysis**: calc. for $C_{33}H_{45}AlN_2O$ – C 77.31%, H 8.85%, N 5.46%; found – C 77.35%, H 8.99%, N 5.45%.

2.2.2 Synthesis of Dipp-*BDI*Al(OC_4H_3Me) (4b)

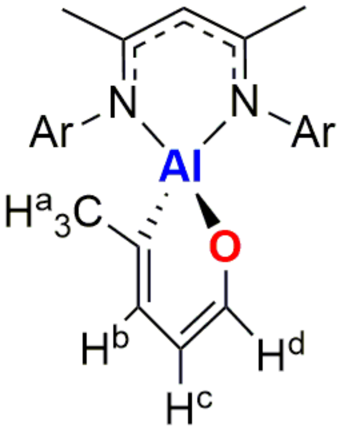

Ar = Dipp

In a glovebox, Dipp-*BDI*Al(I) (**1**, 5.0 mg, 0.0113 mmol) was dissolved in cyclohexane (0.55 mL) and 2-methylfuran (2.0 µL, 0.225 mmol, 2 equiv.) was added by micropipette. The solution was mixed and transferred to a Young's NMR tube and sealed and removed from the glovebox. The tube was heated to 80 °C for 96 hours with monitoring by NMR spectroscopy. ^{1}H NMR spectroscopy showed formation of a major and minor product in a ratio of 80:20 (combined in situ yield 96%). The major product could be isolated as a pure compound. The tube was returned to the glovebox, the solution was transferred to a 4 ml vial and the solvent was removed *in vacuo*. The oily product was dissolved in *n*-heptane (0.3 mL) and the mixture stored at -35 °C. Colourless crystals of the product formed, the mother liquor was decanted. The solid was redissolved in *n*-heptane (0.3 mL) and the solid recrystallized at -35 °C. The product **4b** was dried *in vacuo*. An isolated yield was obtained from a scaled-up reaction using the same conditions and 15.0 mg (0.0338 mmol) of **1**. Isolated yield: 8 mg (45%). **^{1}H NMR** (400 MHz, C_6D_6, 298 K): δ 7.10-7.02 (m, 6H, Dipp-Ar-H), 6.78 (d, $^{3}J_{HH}$ = 5.1 Hz, 1H, H^d), 6.70 (d, $^{3}J_{HH}$ = 5.7 Hz, 1H, H^b), 4.91 (overlapping s, 1H, C-H), 4.89 (overlapping m, 1H, H^c), 3.50 (sept, $^{3}J_{HH}$ = 6.8 Hz, 2H, -$CHMe_2$), 3.26 (sept, $^{3}J_{HH}$ = 6.8 Hz, 2H, -$CHMe_2$), 1.97 (s, 3H, $CH^a{}_3$), 1.53 (s, 6H, CH_3), 1.40 (d, $^{3}J_{HH}$ = 6.8 Hz, 6H, Dipp-CH_3), 1.33 (d, $^{3}J_{HH}$ = 6.8 Hz, 6H, Dipp-CH_3), 1.17 (d, $^{3}J_{HH}$ = 6.8 Hz, 6H, Dipp-CH_3), 1.12 (d, $^{3}J_{HH}$ = 6.8 Hz, 6H, Dipp-CH_3) ppm; **^{13}C{^{1}H} NMR** (100.6 MHz, C_6D_6, 298 K): δ 169.93, 148.67 (s, C-H^d), 144.73, 143.49 (s, C-H^b), 143.30, 139.70, 127.26, 124.42, 123.79, 104.03 (s, C-H^c), 97.92, 28.36, 28.32, 26.12 (s, C-H^a), 25.39, 24.48, 24.29 (2 overlapping peaks), 22.96. The C-Al resonance could not be observed due to line-broadening associated with coupling to the quadrapolar I = 5/2 ^{27}Al nucleus. **Elemental analysis**: calc. for $C_{34}H_{47}AlN_2O$ – C 77.53%, H 8.99%, N 5.32%; found – C 77.75%, H 9.14%, N 5.30%.

2.2.3 Synthesis of Dipp-*BDI*Al($OC_4H_2Me_2$) (5c)

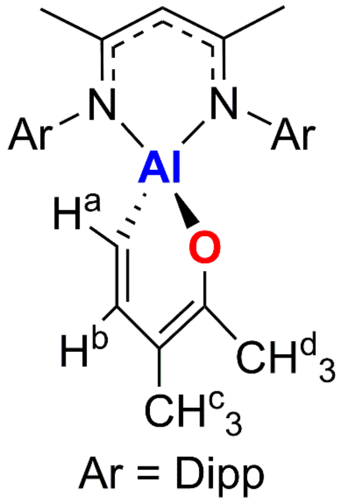

In a glovebox, Dipp-*BDI*Al(I) (**1**, 5.0 mg, 0.0113 mmol) was dissolved in cyclohexane (0.55 mL) and 2,3-dimethylfuran (2.4 µL, 0.225 mmol, 2 equiv.) was added by micropipette. The solution was mixed and transferred to a Young's NMR tube, sealed and removed from the glovebox. The tube was heated to 80 °C for 96 hours with monitoring by NMR spectroscopy. ^{1}H NMR spectroscopy showed formation of a major and minor product in a ratio of 90:10 (combined in situ yield 86%). The major product could be isolated as a pure compound. The tube was returned to the glovebox, the solution was transferred to a 4 ml vial and the solvent was removed *in vacuo*. The oily product was dissolved in *n*-heptane (0.3 mL) and the mixture stored at -35 °C. Colourless crystals of the product formed, the mother liquor was decanted and the product **5c** was dried *in vacuo*. An isolated yield was obtained from a scaled-up reaction using the same conditions and 15.0 mg (0.0338 mmol) of **1**. Isolated yield: 10 mg (55%). **^{1}H NMR** (400 MHz, C_6D_6, 298 K): δ 7.09-7.02 (overlapping m, 6H, Dipp-Ar-H), 7.00 (overlapping d, $^{3}J_{HH}$ = 16.0 Hz, 1H, H^b), 5.63 (d, $^{3}J_{HH}$ = 16.0 Hz, 1H, H^a), 4.93 (s, 1H, C-H), 3.59 (sept, $^{3}J_{HH}$ = 6.8 Hz, 2H, -$CHMe_2$), 3.28 (sept, $^{3}J_{HH}$ = 6.8 Hz, 2H, -$CHMe_2$), 2.02 (s, 3H, $CH^d{}_3$), 1.61 (s, 3H, $CH^c{}_3$), 1.53 (s, 6H, CH_3), 1.38 (d, $^{3}J_{HH}$ = 6.8 Hz, 6H, Dipp-CH_3), 1.31 (d, $^{3}J_{HH}$ = 6.8 Hz, 6H, Dipp-CH_3), 1.20 (d, $^{3}J_{HH}$ = 6.8 Hz, 6H, Dipp-CH_3), 1.08 (d, $^{3}J_{HH}$ = 6.8 Hz, 6H, Dipp-CH_3) ppm; **^{13}C{^{1}H} NMR** (100.6 MHz, C_6D_6, 298 K): δ 169.71, 155.41 (s, C-H^b), 152.28 (s, **C**-$CH^d{}_3$), 144.93, 143.03, 139.76, 127.24, 124.39, 123.62, 105.37 (s, **C**-$CH^c{}_3$), 97.81, 28.73, 27.87, 25.28, 24.33, 24.18, 24.04, 22.97, 22.20 (s, $CH^d{}_3$), 19.14 (s, $CH^c{}_3$). The C–Al resonance could not be observed due to line-broadening associated with coupling to the quadrapolar *I* = 5/2 ^{27}Al nucleus.

2.2.4 Synthesis of Dipp-*BD*IAl(OC$_4$H$_3$(OMe)) (4d)

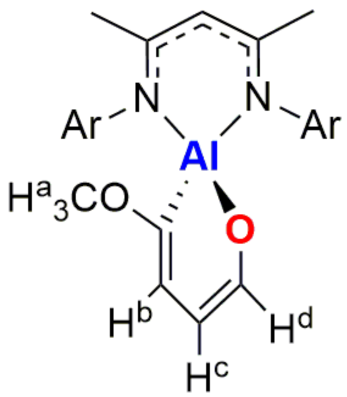

In a glovebox, Dipp-*BD*IAl(I) (**1**, 5.0 mg, 0.0113 mmol) was dissolved in cyclohexane (0.55 mL) and 2-methoxyfuran (2.1 µL, 0.225 mmol, 2 equiv.) was added by micropipette. The solution was mixed and transferred to a Young's NMR tube, sealed and removed from the glove box. The tube was left at room temperature for 2 hours with monitoring by NMR spectroscopy. Once the reaction was complete (in situ yield 96%), the solution was returned to the glove box, transferred to a 4 mL and the solvent removed *in vacuo*. The oily product was dissolved in *n*-heptane (0.3 mL) and stored at -35 °C. Colourless crystals of the product **4d** formed, the mother liquor was decanted and the product dried *in vacuo*. An isolated yield was obtained from a scaled-up reaction using the same conditions and 15.0 mg (0.0338 mmol) of **1**. Isolated yield: 14 mg (76%). **^{1}H NMR** (400 MHz, C_6D_6, 298 K): δ 7.10-7.05 (overlapping m, 6H, Dipp-Ar-H), 6.64 (d, $^3J_{HH}$ = 5.6 Hz, 1H, H^d), 5.80 (d, $^3J_{HH}$ = 7.3 Hz, 1H, H^b), 4.90 (s, 1H, C-H), 4.76 (dd, $^3J_{HH}$ = 7.3 Hz, $^3J_{HH}$ = 5.6 Hz, 1H, H^c), 3.47 (overlapping sept, $^3J_{HH}$ = 6.8 Hz, 2H, -CHMe$_2$), 3.44 (overlapping sept, $^3J_{HH}$ = 6.8 Hz, 2H, -CHMe$_2$), 3.14 (s, 3H, OCH$^a_3$), 1.53 (s, 6H, CH$_3$), 1.44 (d, $^3J_{HH}$ = 6.8 Hz, 6H, Dipp-CH$_3$), 1.35 (d, $^3J_{HH}$ = 6.8 Hz, 6H, Dipp-CH$_3$), 1.16 (overlapping d, $^3J_{HH}$ = 6.8 Hz, 6H, Dipp-CH$_3$), 1.15 (overlapping d, $^3J_{HH}$ = 6.8 Hz, 6H, Dipp-CH$_3$) ppm; **^{13}C{^{1}H} NMR** (100.6 MHz, C_6D_6, 298 K): δ 170.23, 144.69, 143.94, 143.78 (s, C-H^d), 139.13, 127.37, 124.24, 123.85, 115.40 (s, C-H^b), 100.88 (s, C-H^c), 97.72, 52.27 (s, C-H^a), 28.48, 28.39, 25.32, 24.69, 24.39, 24.31, 22.92 ppm. The C–Al resonance could not be observed due to line-broadening associated with coupling to the quadrupolar *I* = 5/2 ^{27}Al nucleus. **Elemental analysis**: calc. for $C_{34}H_{47}AlN_2O_2$ – C 75.24%, H 8.73%, N 5.16%; found – C 75.29%, H 8.89%, N 5.14%.

2.2.5 Synthesis of Dipp-*BDI*Al(OC_4H_4) (4g)

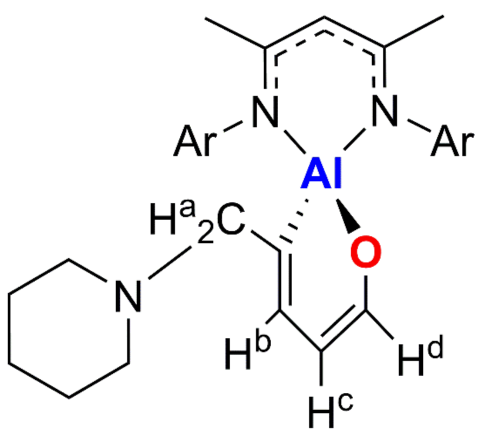

In a glovebox, Dipp-*BDI*Al(I) (**1**, 5.0 mg, 0.0113 mmol) was dissolved in cyclohexane (0.55 mL) and **3g** (2.4 µL, 0.225 mmol, 2 equiv.) was added by micropipette. The solution was mixed and transferred to a Young's NMR tube, sealed and removed from the glovebox. The tube was heated to 80 °C for 66 hours with monitoring by NMR spectroscopy. ^{1}H NMR spectroscopy showed formation of a major and minor product in a ratio of 76:24 (combined in situ yield 89%). The major product could be isolated as a pure compound. The tube was returned to the glovebox and solution was transferred to a 4 mL vial. The solvent was removed *in vacuo*. The oily product was dissolved in *n*-heptane (0.3 mL) and the mixture stored at -35 °C. Colourless crystals of the product formed, the mother liquor was decanted. The solid was redissolved in *n*-heptane (0.5 mL), filtered through a glass fibre plug and the solution concentrated *in vacuo* to recrystallize the solid at -35 °C. The product **4g** was dried *in vacuo*. An isolated yield was obtained from a scaled-up reaction using the same conditions and 15.0 mg (0.0338 mmol) of **1**. Isolated yield: 9 mg (45%). **^{1}H NMR** (400 MHz, C_6D_6, 298 K): δ 7.18 (d, $^3J_{HH}$ = 6.8 Hz, 1H, H^b), 7.14-7.07 (overlapping m, 6H, Dipp-Ar-H), 6.97 (d, $^3J_{HH}$ = 5.4 Hz, 1H, H^d), 5.12 (dd, $^3J_{HH}$ = 6.8 Hz, $^3J_{HH}$ = 5.4 Hz, 1H, H^c), 5.03 (s, 1H, C-H), 3.54 (sept, $^3J_{HH}$ = 6.8 Hz, 2H, -CHMe$_2$), 3.27 (sept, $^3J_{HH}$ = 6.8 Hz, 2H, -CHMe$_2$), 2.91 (s, 2H, CH^a$_2$), 2.05 (br, 4H, N–C^{cyclic}H$_2$), 1.56 (s, 6H, CH$_3$), 1.43 (d, $^3J_{HH}$ = 6.8 Hz, 6H, Dipp-CH$_3$), 1.35-1.22 (overlapping br m, 6H, C^{cyclic}H$_2$), 1.33 (overlapping d, $^3J_{HH}$ = 6.8 Hz, 6H, Dipp-CH$_3$), 1.20 (d, $^3J_{HH}$ = 6.8 Hz, 6H, Dipp-CH$_3$), 1.09 (d, $^3J_{HH}$ = 6.8 Hz, 6H, Dipp-CH$_3$) ppm; **^{13}C{^{1}H} NMR** (100.6 MHz, C_6D_6, 298 K): δ 169.92, 149.44 (s, C-H^d), 145.09, 143.10 (s, C-H^b), 142.84, 140.40, 127.09, 124.51, 123.63, 104.34 (s, C-H^c), 98.81, 67.18 (s, C-H^a$_2$), 54.91, 28.70, 28.19, 25.89, 25.67, 24.91, 24.44, 24.31, 24.24, 23.10. The C–Al resonance could not be observed due to line-broadening associated with coupling to the quadrupolar *I* = 5/2 ^{27}Al nucleus.

2.3 Aluminated Products from Palladium Catalysed Insertion into the C–O Bonds of Furan and Derivatives

2.3.1 Synthesis of Dipp-*BDI*Al(OC_4H_4) (4a)

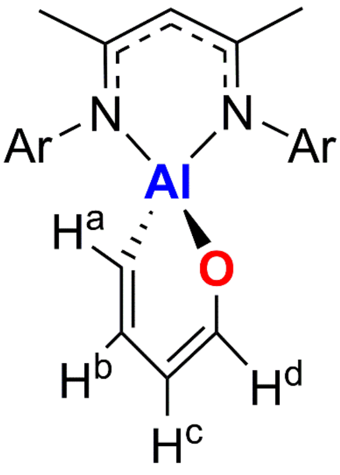

Ar = Dipp

In a glove box, Dipp-*BDI*Al(I) (**1**, 5.0 mg, 0.0113 mmol) and [Pd(PCy_3)$_2$] (50 µL, 0.0113 M solution in cyclohexane, 0.00056 mmol, 5 mol%) were dissolved in cyclohexane (0.50 mL) and furan (1.6 µL, 0.225 mmol, 2 equiv.) was added by micropipette. The solution was mixed and transferred to a Young's NMR tube, sealed and removed from the glove box. The tube was allowed to stand at 25 °C for 18 hours with monitoring by NMR spectroscopy. Once consumption of **1** and formation of Dipp-*BDI*AlH(C_4H_3O) (**7a**) was complete the tube was heated to 80 °C for 144 hours. Once the reaction was complete (in situ yield 90%) the solution was transferred to a 4 mL vial in the glove box and the solvent removed *in vacuo*. The oily product was dissolved in *n*-heptane (0.3 mL) and stored at -35 °C. Colourless crystals of the product formed, the mother liquor was decanted and the product **4a** dried *in vacuo*. The NMR spectra of the complex matched those for the same product generated via the uncatalysed reaction described in section 2.2.1.

2.3.2 Synthesis of Dipp-*BDI*Al(OC_4H_3Me) (5b)

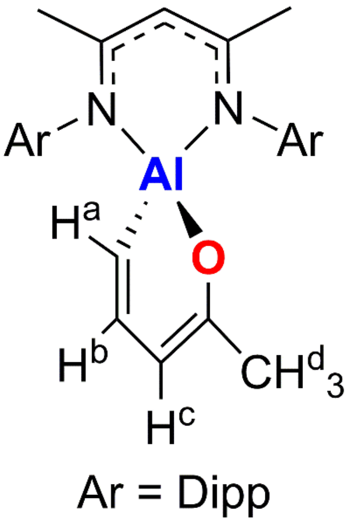

In a glove box, Dipp-*BDI*Al(I) (**1**, 5.0 mg, 0.0113 mmol) and [Pd(PCy_3)$_2$] (50 µL, 0.0113 M solution in cyclohexane, 0.00056 mmol, 5 mol%) were dissolved in cyclohexane (0.50 mL) and 2-methylfuran (1.6 µL, 0.225 mmol, 2 equiv.) was added by micropipette. The solution was mixed and transferred to a Young's NMR tube, sealed and removed from the glove box. The tube was allowed to stand at 25 °C for 18 hours with monitoring by NMR spectroscopy. Once consumption of **1** was complete the tube was heated to 80 °C for 120 hours. Once the reaction was complete (in situ yield 94%) the solution was returned to the glove box, transferred to a 4 mL vial and the solvent removed *in vacuo*. The oily product was dissolved in *n*-heptane (0.3 mL) and stored at -35 °C. Colourless crystals of the product formed, the mother liquor was decanted and the product **5b** dried *in vacuo*. An isolated yield was obtained from a scaled-up reaction using the same conditions and 15.0 mg (0.0338 mmol) of **1**. Isolated yield: 11 mg (62%). **^{1}H NMR** (400 MHz, C_6D_6, 298 K): δ 7.11-7.02 (overlapping m, 7H, Dipp-Ar-H + H^b), 5.65 (d, $^3J_{HH}$ = 15.9 Hz, 1H, H^a), 4.95 (overlapping d, $^3J_{HH}$ ≈ 6.5 Hz, 1H, H^c), 4.93 (overlapping s, 1H, C-H), 3.57 (sept, $^3J_{HH}$ = 6.8 Hz, 2H, -CHMe$_2$), 3.28 (sept, $^3J_{HH}$ = 6.8 Hz, 2H, -CHMe$_2$), 1.98 (s, 3H, CH$^d{}_3$), 1.52 (s, 6H, CH_3), 1.37 (overlapping d, $^3J_{HH}$ = 6.8 Hz, 6H, Dipp-CH_3), 1.34 (overlapping d, $^3J_{HH}$ = 6.8 Hz, 6H, Dipp-CH_3), 1.19 (d, $^3J_{HH}$ = 6.8 Hz, 6H, Dipp-CH_3), 1.08 (d, $^3J_{HH}$ = 6.8 Hz, 6H, Dipp-CH_3) ppm; **^{13}C{^{1}H} NMR** (100.6 MHz, C_6D_6, 298 K): δ 169.81, 157.87, 149.05 (s, C-H^b), 144.92, 143.04, 139.66, 127.29, 124.41, 123.66, 102.29 (s, C-H^c), 97.89, 28.76, 28.02, 25.39, 24.61 (s, C-H$^d{}_3$), 24.37, 24.17, 24.02, 22.99 ppm. The C–Al resonance could not be observed due to line-broadening associated with coupling to the quadrupolar *I* = 5/2 ^{27}Al nucleus.

2.3.3 Synthesis of Dipp-*BDI*Al($OC_4H_2Me_2$) (5c)

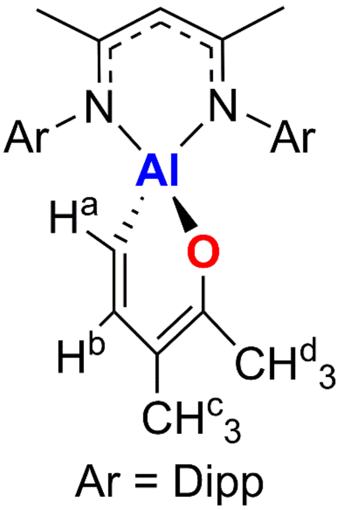

In a glove box, Dipp-*BDI*Al(I) (**1**, 5.0 mg, 0.0113 mmol) and [Pd(PCy_3)$_2$] (50 µL, 0.0113 M solution in cyclohexane, 0.00056 mmol, 5 mol%) were dissolved in cyclohexane (0.50 mL) and 2,3-dimethylfuran (2.4 µL, 0.225 mmol, 2 equiv.) was added by micropipette. The solution was mixed and transferred to a Young's NMR tube, sealed and removed from the glove box. The tube was allowed to stand at 25 °C for 18 hours with monitoring by NMR spectroscopy. Once consumption of **1** was complete the tube was heated to 80 °C for 72 hours. Once the reaction was complete (in situ yield 87%) the solution was transferred to a 4 mL vial in the glove box and the solvent removed *in vacuo*. The oily product was dissolved in *n*-heptane (0.3 mL) and stored at -35 °C. Colourless crystals of the product formed, the mother liquor was decanted and the product **5c** dried *in vacuo*. The NMR spectra of the complex matched those for the same product generated via the uncatalysed reaction described in section 2.2.3.

2.3.4 Synthesis of Dipp-*BDI*Al(OC_4H_6) (5e)

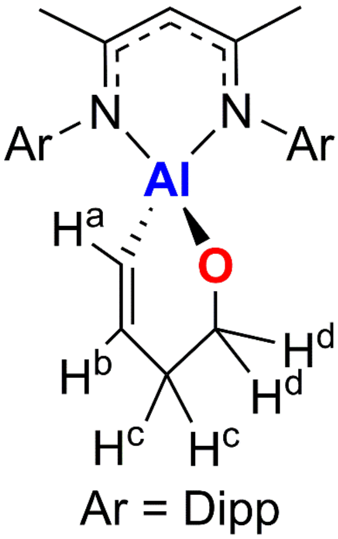

In a glove box, Dipp-*BDI*Al(I) (**1**, 5.0 mg, 0.0113 mmol) and [Pd(PCy_3)$_2$] (50 μL, 0.0113 M solution in cyclohexane, 0.00056 mmol, 5 mol%) were dissolved in cyclohexane (0.50 mL) and 2,3-dihydrofuran (1.7 μL, 0.225 mmol, 2 equiv.) was added by micropipette. The solution was mixed and transferred to a Young's NMR tube, sealed and removed from the glove box. The tube was allowed to stand at 25 °C for 5 minutes (in situ yield 99%). The solution was returned to the glove box, transferred to a 4 mL and the solvent removed *in vacuo*. The oily product was dissolved in *n*-heptane (0.3 mL) and stored at -35 °C. Colourless crystals of the product formed, the mother liquor was decanted and the product **5e** dried *in vacuo*. An isolated yield was obtained on a scaled up reaction using the same conditions and 15.0 mg (0.0338 mmol) of **1**. Isolated yield: 9 mg (52%). **^{1}H NMR** (400 MHz, C_6D_6, 298 K): δ 7.16-7.01 (overlapping m, 7H, Dipp-Ar-H + H^b), 6.02 (d, $^3J_{HH}$ = 15.5 Hz, 1H, H^a), 4.91 (s, 1H, C-H), 3.73 (d, $^3J_{HH}$ = 5.3 Hz, 2H, H^d), 3.48 (sept, $^3J_{HH}$ = 6.8 Hz, 2H, -$CHMe_2$), 3.26 (sept, $^3J_{HH}$ = 6.8 Hz, 2H, -$CHMe_2$), 1.73 (m, 2H, H^c), 1.59 (s, 6H, CH_3), 1.55 (d, $^3J_{HH}$ = 6.8 Hz, 6H, Dipp-CH_3), 1.38 (d, $^3J_{HH}$ = 6.8 Hz, 6H, Dipp-CH_3), 1.14 (d, $^3J_{HH}$ = 6.8 Hz, 6H, Dipp-CH_3), 1.09 (d, $^3J_{HH}$ = 6.8 Hz, 6H, Dipp-CH_3) ppm; **^{13}C{^{1}H} NMR** (100.6 MHz, C_6D_6, 298 K): δ 169.92, 151.56 (s, C-H^b), 145.09, 143.77, 139.22, 127.17, 124.31, 123.80, 96.25, 63.77 (s, C-H^d), 35.68 (s, C-H^c), 28.39, 27.97, 24.81, 24.77, 24.53, 24.47, 22.89 ppm. The C–Al resonance could not be observed due to line-broadening associated with coupling to the quadrupolar I = 5/2 ^{27}Al nucleus.

2.3.5 Synthesis of Dipp-*BDI*Al(OC_5H_8) (5f)

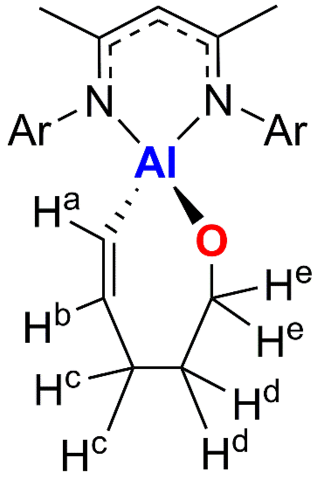

In a glove box, Dipp-*BDI*Al(I) (**1**, 5.0 mg, 0.0113 mmol) and [Pd(PCy_3)$_2$] (50 µL, 0.0113 M solution in cyclohexane, 0.00056 mmol, 5 mol%) were dissolved in cyclohexane (0.50 mL) and 3,4-dihydro-*2H*-pyran (2.1 µL, 0.225 mmol, 2 equiv.) was added by micropipette. The solution was mixed and transferred to a Young's NMR tube, sealed and removed from the glove box. The tube was allowed to stand at 25 °C for 16 hours with monitoring by NMR spectroscopy. Once the reaction was complete (in situ yield 98%) the solution was returned to the glove box, transferred to a 4 mL vial and the solvent removed *in vacuo*. The oily product was dissolved in *n*-heptane (0.2 mL) and stored at -35 °C. Colourless crystals of the product formed, the mother liquor was decanted and the product **5f** dried *in vacuo*. An isolated yield was obtained on a scaled up reaction using the same conditions and 20.0 mg (0.0450 mmol) of **1**. Isolated yield: 10 mg (42%). **^{1}H NMR** (400 MHz, C_6D_6, 298 K): δ 7.18-7.06 (overlapping m, 6H, Dipp-Ar-H), 6.92 (dt, $^3J_{HH}$ = 15.7 Hz, $^3J_{HH}$ = 4.4 Hz, 1H, H^b), 5.94 (d, $^3J_{HH}$ = 15.7 Hz, 1H, H^a), 4.88 (s, 1H, C-H), 3.64 (overlapping m, 2H, H^e), 3.58 (overlapping sept, $^3J_{HH}$ = 6.8 Hz, 2H, -CHMe$_2$), 3.28 (sept, $^3J_{HH}$ = 6.8 Hz, 2H, -CHMe$_2$), 1.99 (m, 2H, H^c), 1.58 (s, 6H, CH_3), 1.52 (d, $^3J_{HH}$ = 6.8 Hz, 6H, Dipp-CH_3), 1.36 (d, $^3J_{HH}$ = 6.8 Hz, 6H, Dipp-CH_3), 1.25-1.19 (m, 2H, H^d), 1.16 (overlapping d, $^3J_{HH}$ = 6.8 Hz, 6H, Dipp-CH_3), 1.14 (overlapping d, $^3J_{HH}$ = 6.8 Hz, 6H, Dipp-CH_3) ppm; **^{13}C{^{1}H} NMR** (100.6 MHz, C_6D_6, 298 K): δ 169.45, 151.14 (s, C-H^b), 144.94, 144.07, 139.87, 135.53 (s, C-H^a), 127.05, 124.14, 123.96, 96.31, 65.02 (s, C-H^e), 34.74 (s, C-H^c), 33.83 (s, C-H^d), 28.43, 27.68, 25.06, 24.88, 24.77, 24.47, 23.08 ppm. The C-Al resonance was detected by the HMQC experiment.

2.3.6 Synthesis of Dipp-*BDI*Al(OC_4H_4) (5g)

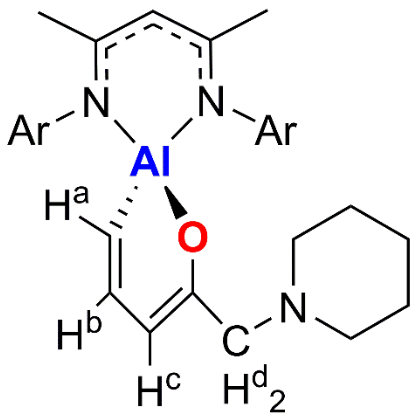

Ar = Dipp

In a glove box, Dipp-*BDI*Al(I) (**1**, 5.0 mg, 0.0113 mmol) and [Pd(PCy_3)$_2$] (50 µL, 0.0113 M solution in cyclohexane, 0.00056 mmol, 5 mol%) were dissolved in cyclohexane (0.50 mL) and **3g** (2.4 µL, 0.225 mmol, 2 equiv.) was added by micropipette. The solution was mixed and transferred to a Young's NMR tube, sealed and removed from the glove box. The tube was allowed to stand at 25 °C for 68 hours with monitoring by NMR spectroscopy. Once consumption of **1** was complete the tube was heated to 80 °C for 66 hours. Once the reaction was complete (in situ yield 92%) the tube was returned to the glove box, the solution was transferred to a 4 mL vial and the solvent removed *in vacuo*. The oily product was dissolved in *n*-heptane (0.3 mL) and stored at -35 °C. Colourless crystals of the product formed, the mother liquor was decanted and the product **5g** dried *in vacuo*. An isolated yield was obtained on a scaled up reaction using the same conditions and 15.0 mg (0.0338 mmol) of **1**. Isolated yield: 12 mg (58%). **^{1}H NMR** (400 MHz, C_6D_6, 298 K): δ 7.21 (dd, $^3J_{HH}$ = 15.6 Hz, $^3J_{HH}$ = 6.5 Hz, 1H, H^b), 7.13-7.02 (overlapping m, 6H, Dipp-Ar-H), 5.78 (d, $^3J_{HH}$ = 15.6 Hz, 1H, H^a), 5.45 (d, $^3J_{HH}$ = 6.5 Hz, 1H, H^c), 4.93 (s, 1H, C-H), 3.51 (sept, $^3J_{HH}$ = 6.8 Hz, 2H, -CHMe$_2$), 3.29 (sept, $^3J_{HH}$ = 6.8 Hz, 2H, -CHMe$_2$), 3.05 (s, 2H, H^d), 2.37 (m, 4H, N-C^{cyc}H$_2$), 1.53 (s, 6H, CH_3), 1.50-1.17 (overlapping m, 6H, C^{cyc}H$_2$), 1.42 (overlapping d, $^3J_{HH}$ = 6.8 Hz, 6H, Dipp-CH_3), 1.36 (overlapping d, $^3J_{HH}$ = 6.8 Hz, 6H, Dipp-CH_3), 1.20 (overlapping m, d, $^3J_{HH}$ = 6.8 Hz, 6H, Dipp-CH_3), 1.09 (d, $^3J_{HH}$ = 6.8 Hz, 6H, Dipp-CH_3) ppm; **^{13}C{^{1}H} NMR** (100.6 MHz, C_6D_6, 298 K): δ 169.82, 157.78, 148.33 (s, C-H^b), 144.71, 143.31, 139.59, 127.24, 126.12 (s, C-H^a), 124.28, 123.79, 100.97 (s, C-H^c), 97.76, 64.96 (s, C-H$^d{}_2$), 55.35, 28.50, 28.08, 26.29, 25.64, 24.63, 24.48, 24.29, 24.24, 23.01. The C-Al resonance was detected by the HMQC experiment.

2.4 Aluminated Products from Palladium Catalysed Insertion into C–O Bonds from Aluminium Dihydride

2.4.1 General procedure for determining the NMR yield: Once the reaction mixture was transferred to the ampoule, but before heating, an aliquot was taken and transferred into an J Young NMR tube that contained a capillary tube of ferrocene (0.22M in C_6D_6) and a t_0 spectrum was recorded. The aliquot was then returned to the ampoule, the reaction mixture freeze-pump-thaw degassed and heated to 100 ºC. At the end of the reaction, an aliquot was taken again and transferred to the same J Young tube containing the same capillary standard. Yields are based on the relative integrations of starting material and product peaks: Based on the C-H region (4-5 ppm) and where possible the CCH_3 (1-2 ppm) region of the ^{1}H NMR spectrum.

2.4.2 Synthesis of Mes-*BDI*Al(C_4H_3MeO) (6b)

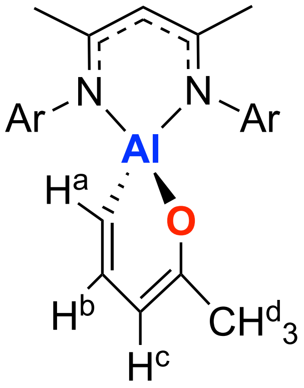

Ar = Mes

In a glovebox, Mes-*BDI*AlH_2 (160 mg, 0.44 mmol) and [Pd(PCy_3)$_2$] (110 μL of a 0.05 M solution in C_6H_6, 0.022 mmol) were dissolved in benzene (~2 mL) in a 20 mL scintillation vial. 2-methylfuran (398 μL, 4.4 mmol, 10 equiv.) was added by micropipette and the solution transferred to an ampoule with an approximate head space volume of 60 mL. The ampoule was removed from the glove box and subject to a freeze-pump-thaw cycle to create a static vacuum in the reaction vessel. The reaction mixture was heated to 100 °C in an oil bath for 48 h before cooling to 25 °C where all volatiles were removed under reduced pressure on a vacuum line. The ampoule was transferred back to the glove box and the residue dissolved in toluene (~2 mL), filtered through a 0.45 μm syringe filter and evaporated to dryness. The crude product was then dissolved in a mixture of *n*-hexane (~2 mL) and toluene (~0.1 mL) and filtered through a 0.45 μm syringe filter. Pale-yellow crystalline material was obtained after storage of the mixture at -35 °C. The mother liquor was decanted from the vial and the solid material washed with cold *n*-hexane (2 x 1 mL) and dried *in vacuo*. NMR yield 95 % based on a ferrocene in C_6D_6 standard, inside a capillary tube. Isolated yield 113.3 mg (58.0 %, 0.256 mmol). **^{1}H NMR** (400 MHz, C_6D_6, 298 K): δ 7.12 (dd, $^3J_{HH}$ = 15.7 Hz, $^3J_{HH}$ = 6.4 Hz, 1H, H^b), 6.74 (s, 2H, Mes-Ar-H), 6.7 (s, 2H, Mes-Ar-H), 5.90 (d, $^3J_{HH}$ = 15.7 Hz, 1H, H^a), 4.94 (s, 1H, C-H), 4.83 (d, $^3J_{HH}$ = 6.4 Hz, 1H, H^c), 2.41 (s, 6H, Mes-CH_3), 2.21 (s, 6H, Mes-CH_3), 2.02 (s, 6H, Mes-CH_3), 1.93 (s, 3H, $CH^d{}_3$), 1.49 (s, 6H, CH_3); **^{13}C{^{1}H} NMR** (100.6 MHz, C_6D_6, 298K): δ 169.78, 157.09 (s, C-$H^d{}_3$), 148.69 (s, C-H^b), 140.02, 135.8, 134.00, 132.77, 129.96, 129.61, 125.12 (s, C-H^a), 102.42 (s, C-H^c), 97.29, 24.65 (s, C-$H^d{}_3$), 22.39, 20.91, 18.80, 18.56. The C–Al resonance was detected by the HSQC experiment. **Elemental analysis**: calc. for $C_{28}H_{35}AlN_2O$ – C 75.99%, H 7.97%, N 6.33%; found – C 75.90%, H 7.84%, N 6.24%.

2.4.3 Synthesis of Mes-*BDI*Al($C_4H_2Me_2O$) (6c)

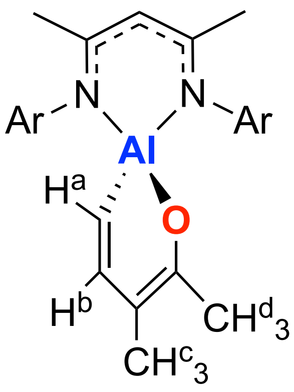

In a glovebox, Mes-*BDI*AlH_2 (160 mg, 0.44 mmol) and [Pd(PCy_3)$_2$] (110 µL of a 0.05 M solution in C_6H_6, 0.022 mmol) were dissolved in benzene (~2 mL) in a 20 mL scintillation vial. 2,3-dimethylfuran (466 µL, 4.4 mmol, 10 equiv.) was added by micropipette and the solution transferred to an ampoule with an approximate head space volume of 60 mL. The ampoule was removed from the glove box and subject to a freeze-pump-thaw cycle to create a static vacuum in the reaction vessel. The reaction mixture was heated to 100 °C in an oil bath for 48 h before cooling to 25 °C where all volatiles were removed under reduced pressure on a vacuum line. The ampoule was transferred back to the glove box and the residue dissolved in toluene (~2 mL), filtered through a 0.45 µm syringe filter and evaporated to dryness. The crude product was then dissolved in a mixture of *n*-hexane (~2 mL) and toluene (~0.1 mL) and filtered through a 0.45 µm syringe filter. Pale-yellow crystalline material was obtained after storage of the mixture at -35 °C. The mother liquor was decanted from the vial and the solid material washed with cold *n*-hexane (2 x 1 mL) and dried *in vacuo*. NMR yield 92 % against ferrocene capillary standard. Isolated yield 131.1 mg, 0.287 mmol, 67.1%. **^{1}H NMR** (400 MHz, C_6D_6, 298K): δ 7.09 (d, 1H, $^3J_{HH}$ = 15.7 Hz, H^b), 6.71 (s, 4H, Mes-Ar-H), 5.93 (d, 1H, $^3J_{HH}$ = 15.8 Hz, H^a), 4.94 (s, 1H, C-H), 2.43 (s, 6H, Mes-CH_3), 2.22 (s, 6H, Mes-CH_3), 2.01 (s, 3H, $CH^d{}_3$), 1.99 (s, 6H, Mes-CH_3), 1.49 (s, 6H, CH_3), 1.48 (s, 3H, $CH^c{}_3$). **^{13}C{^{1}H} NMR** (100.6 MHz, C_6D_6, 298K): δ 169.72, 155.10 (s, C-H^b), 151.67, 140.13, 135.80, 134.01, 132.72, 130.00, 129.59 , 126.17 (s, C-H^a), 105.59, 97.25, 22.41, 21.93 (s, C-$H^d{}_3$), 20.88, 19.51 (s, C-$H^c{}_3$), 18.80, 18.63. The C–Al resonance was detected by the HSQC experiment. **Elemental analysis**: calc. for $C_{29}H_{37}AlN_2O$ – C 76.28%, H 8.17%, N 6.14%; found – C 76.26%, H 8.28%, N 6.08%.

2.4.4 Synthesis of Mes-*BDI*Al(C_4H_3O(OMe)) (6d)

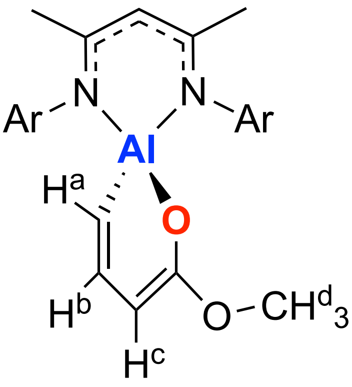

In a glovebox, Mes-*BDI*AlH_2 (160 mg, 0.44 mmol) and [Pd(PCy_3)$_2$] (110 µL of a 0.05 M solution in C_6H_6, 0.022 mmol) were dissolved in benzene (~2 mL) in a 20 mL scintillation vial. 2-methoxyfuran (407 µL, 4.4 mmol, 10 equiv.) was added by micropipette and the solution transferred to an ampoule with an approximate head space volume of 60 mL. The ampoule was removed from the glove box and subject to a freeze-pump-thaw cycle to create a static vacuum in the reaction vessel. The reaction mixture was heated to 100 °C in an oil bath for 48 h before cooling to 25 °C where all volatiles were removed under reduced pressure on a vacuum line. The ampoule was transferred back to the glove box and the residue dissolved in toluene (~2 mL), filtered through a 0.45 µm syringe filter and evaporated to dryness. The crude product was then dissolved in a mixture of *n*-hexane (~2 mL) and toluene (~0.1 mL) and filtered through a 0.45 µm syringe filter. A pale-orange solid was obtained after storage of the mixture at -35 °C. The mother liquor was decanted from the vial and the solid material washed with cold *n*-hexane (2 x 1 mL) and dried *in vacuo*. NMR yield 95 % against ferrocene capillary standard. Isolated yield 153.8 mg, 0.335 mmol, 76 %. **^{1}H NMR** (400 MHz, C_6D_6, 298K): δ 7.21 (dd, $^3J_{HH}$ = 15.4 Hz, $^3J_{HH}$ = 6.9 Hz, 1H, H^b), 6.72 –6.71 (overlapping m, 4H, Mes-Ar-H), 5.58 (d, $^3J_{HH}$ = 15.4 Hz, 1H, H^a), 4.94 (s, 1H, C-H), 4.17 (d, $^3J_{HH}$ = 6.6 Hz, 1H, H^c), 3.27 (s, 3H, CH^d_3), 2.41 (s, 6H, Mes-CH_3), 2.22 (s, 6H, Mes-CH_3), 2.00 (s, 6H, Mes-CH_3), 1.48 (s, 6H, CH_3) ppm; **^{13}C{^{1}H} NMR** (100.1 MHz, C_6D_6, 298K): δ 169.99, 162.49, 149.44 (s, C-H^b), 139.75, 135.84, 133.91, 132.78, 130.01, 129.59, 117.32 (s, C-H^a), 97.42, 76.90 (s, C-H^c), 53.07 (s, C-H^d_3), 22.34, 20.84, 18.78, 18.53. The C–Al resonance was detected by the HSQC experiment. **Elemental analysis**: calc. for $C_{28}H_{35}AlN_2O_2$ – C 73.34%, H 7.69%, N 6.11%; found – C 73.10%, H 7.84%, N 6.00%.

2.4.5 Synthesis of Mes-*BDI*Al($C_4H_3CH_2NC_5H_{10}$) (6g)

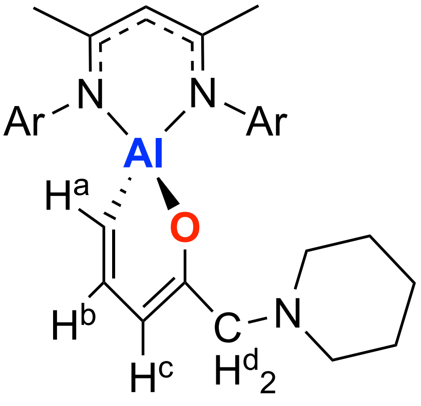

In a glovebox, Mes-*BDI*AlH_2 (160 mg, 0.44 mmol) and [Pd(PCy_3)$_2$] (110 µL of a 0.05 M solution in C_6H_6, 0.022 mmol) were dissolved in benzene (~2 mL) in a 20 mL scintillation vial. **3g** (100 µL, 0.63 mmol, 1.5 equiv.) was added by micropipette and the solution transferred to an ampoule with an approximate head space volume of 60 mL. The ampoule was removed from the glove box and subject to a freeze-pump-thaw cycle to create a static vacuum in the reaction vessel. The reaction mixture was heated to 100 °C in an oil bath for 72 h before cooling to 25 °C where all volatiles were removed under reduced pressure on a vacuum line.[5] The ampoule was transferred back to the glove box and the residue dissolved in toluene (~2 mL), filtered through a 0.45 µm syringe filter and evaporated to dryness. The crude product was then dissolved in a mixture of *n*-hexane (~2 mL) and toluene (~0.1 mL) and filtered through a 0.45 µm syringe filter. Pale-yellow crystalline material was obtained after storage of the mixture at -35 °C. The mother liquor was decanted from the vial and the solid material washed with cold *n*-hexane (2 x 1 mL) and dried *in vacuo*. NMR yield 72 % against ferrocene capillary standard. Isolated yield: 81.6 mg, 0.155 mmol, 35.2%. **^{1}H NMR** (400 MHz, C_6D_6, 298K): δ 7.10 (dd, $^3J_{HH}$ = 15.7 Hz, $^3J_{HH}$ = 6.4 Hz, 1H, H^b), 6.80 (s, 2H, Mes-Ar-H), 6.71 (s, 2H, Mes-Ar-H), 5.94 (d, $^3J_{HH}$ = 15.5 Hz, 1H, H^a), 5.08 (d, $^3J_{HH}$ = 6.0 Hz, 1H, H^c), 4.94 (s, 1H, C-H), 2.96 (s, 2H, CH^d_2), 2.46 (s, 6H, Mes-CH_3), 2.33 (m, 4H, $NC^{cyc}H_2$), 2.21 (s, 6H, Mes-CH_3), 2.03 (s, 6H, Mes-CH_3), 1.57 (m, 4H, $C^{cyc}H_2$), 1.50 (s, 6H, CH_3), 1.37 (m, 2H, $C^{cyc}H_2$). **^{13}C{^{1}H} NMR** (100.6 MHz, C_6D_6, 298K): δ 169.79, 157.08, 148.09 (s, C-H^b), 139.97, 135.79, 134.30, 132.82, 129.81, 129.61, 127.29 (s, C-H^a), 103.42 (s, C-H^c), 97.34, 66.58 (s, C-H^d_2), 55.22, 26.72, 25.16, 22.39, 20.93, 18.91, 18.81. The C–Al resonance was detected by the HSQC experiment. **Elemental analysis**: calc. for $C_{33}H_{44}AlN_3O$ – C 75.39%, H 8.44%, N 7.99%; found – C 75.31%, H 8.56%, N 7.82%.

[5] Shorter reaction times of 48 h were obtained upon using 5 molar equivalents of **3g**.

2.4.6 Synthesis of Mes-*BDI*Al($C_4H_3(OCH_2OSi^iPr_3)$) (6h)

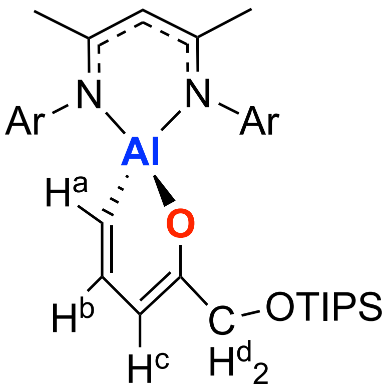

In a glovebox, Mes-*BDI*AlH_2 (160 mg, 0.44 mmol) and [$Pd(PCy_3)_2$] (110 µL of a 0.05 M solution in C_6H_6, 0.022 mmol) were dissolved in benzene (~2 mL) in a 20 mL scintillation vial. **3h** (562 µL, 2.2 mmol, 5 equiv.) was added by micropipette and the solution transferred to an ampoule with an approximate head space volume of 60 mL. The ampoule was removed from the glove box and subject to a freeze-pump-thaw cycle to create a static vacuum in the reaction vessel. The reaction mixture was heated to 100 °C in an oil bath for 96 h before cooling to 25 °C where all volatiles were removed under reduced pressure on a vacuum line. The ampoule was transferred back to the glove box and the residue dissolved in toluene (~2 mL), filtered through a 0.45 µm syringe filter and evaporated to dryness. The crude product was then dissolved in a mixture of *n*-hexane (~2 mL) and toluene (~0.1 mL) and filtered through a 0.45 µm syringe filter. Pale-yellow solid was obtained after storage at -35 °C. The mother liquor was decanted from the vial and the solid material washed with cold *n*-hexane (2 x 1 mL) and dried *in vacuo*. NMR Yield 80 % against ferrocene capillary standard. Isolated yield: 70.0 mg, 0.114 mmol, 26%. **^{1}H NMR** (500 MHz, C_6D_6, 298K): δ 7.17 (dd, $^3J_{HH}$ = 15.6 Hz, $^3J_{HH}$ = 6.4 Hz, 1H, H^b), 6.77 (s, 2H, Mes-Ar-H), 6.72 (s, 2H, Mes-Ar-H), 5.89 (d, $^3J_{HH}$ = 15.6 Hz, 1H, H^a), 5.38 (d, $^3J_{HH}$ = 6.3 Hz, 1H, H^c), 4.94 (s, 1H, C-H), 4.37 (s, 2H, CH^d_2), 2.40 (s, 6H, Mes-CH_3), 2.20 (s, 6H, Mes-CH_3), 2.05 (s, 6H, Mes-CH_3), 1.49 (s, 6H, CH_3), 1.13 (m, 21H, TIPS) ppm; **^{13}C{^{1}H} NMR** (100.6 MHz, C_6D_6, 298K): δ 169.48, 158.02, 147.96 (s, C-H^b), 139.54, 135.47, 133.57, 132.25, 129.58, 129.27, 126.22 (s, C-H^a), 99.31 (s, C-H^c), 96.97, 65.73 (s, C-H^d_2), 21.99, 20.56, 18.39, 18.29, 18.02, 12.14. The C–Al resonance was detected by the HSQC experiment.

2.4.7 Synthesis of Mes-*BDI*Al($C_4H_3O(CH_2OSiMe_2{}^tBu)$) (6i)

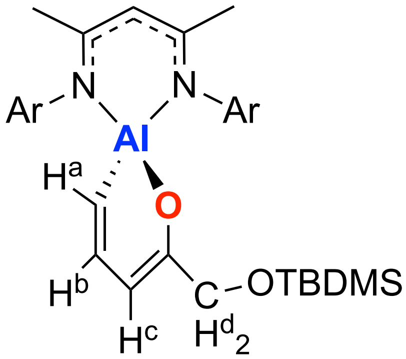

Ar = Mes

TBDMS = *tert*-butyldimethylsilyl

In a glovebox, Mes-*BDI*AlH_2 (160 mg, 0.44 mmol) and [Pd(PCy_3)$_2$] (110 μL of a 0.05 M solution in C_6H_6, 0.022 mmol) were dissolved in benzene (~2 mL) in a 20 mL scintillation vial. **3i** (100 μL, 0.48 mmol, 1.1 equiv.) was added by micropipette and the solution transferred to an ampoule with an approximate head space volume of 60 mL. The ampoule was removed from the glove box and subject to a freeze-pump-thaw cycle to create a static vacuum in the reaction vessel. The reaction mixture was heated to 100 °C in an oil bath for 260 h before cooling to 25 °C where all volatiles were removed under reduced pressure on a vacuum line.[6] The ampoule was transferred back to the glove box and the residue dissolved in toluene (~2 mL), filtered through a 0.45 μm syringe filter and evaporated to dryness to generate a sticky orange solid. The crude product was then dissolved in *n*-hexane (~1 mL) and stored at -35°C. NMR yield 86 % against ferrocene capillary standard. **^{1}H NMR** (500 MHz, C_6D_6, 298K): δ 7.12 (dd overlap with C_6D_6 peak, $^3J_{HH}$ = 15.6 Hz $^3J_{HH}$ = 6.4 Hz, 1H, H^b), 6.75 (s, 2H, Mes-Ar-H), 6.71 (s, 2H, Mes-Ar-H), 5.88 (d, $^3J_{HH}$ = 15.5 Hz, 1H, H^a), 5.24 (d, $^3J_{HH}$ = 7.1 Hz, 1H, H^c), 4.94 (s, 1H, C–H), 4.25 (s, 2H, $CH^d{}_2$), 2.39 (s, 6H, Mes-CH_3), 2.19 (s, 6H, Mes-CH_3) 2.03 (s, 6H, Mes-CH_3), 1.49 (s, 6H, CH_3), 0.98 (s, 6H, Si-CH_3), 0.12 – 0.09 (m, 9H, Si-*t*Bu) ppm; **^{13}C{^{1}H} NMR** (100.6 MHz, C_6D_6, 298K): δ 169.81, 158.30, 148.19 (s, C–H^b), 139.85, 135.81, 133.93, 132.66, 129.91, 129.57, 126.80 (s, C–H^a), 100.06 (s, C–H^c), 97.33, 66.02 (s, $CH^d{}_2$), 26.23, 22.32, 20.86, 18.71, 18.60, 2.03, -5.20. The C–Al resonance was detected by the HSQC experiment.

[6] Shorter reaction times of 72 h were obtained upon using 5 molar equivalents of **3h**, however this resulted in an intractable mixture of excess starting material and product.

2.5 Aluminated Products from Palladium Catalysed Insertion into the C–H Bond of Furan

2.5.1 Synthesis of Dipp-*BDI*AlH(C_4H_3O) (7a)

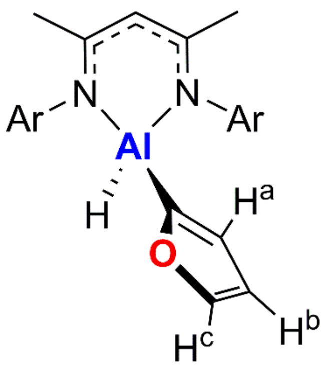

Ar = Dipp

In a glovebox, Dipp-*BDI*Al(I) (**1**, 5.0 mg, 0.0113 mmol) and [Pd(PCy_3)$_2$] (50 µL, 0.0113 M solution in cyclohexane, 0.00056 mmol, 5 mol%) were dissolved in cyclohexane (0.50 mL) and furan (1.6 µL, 0.225 mmol, 2 equiv.) was added by micropipette. The solution was mixed and transferred to a Young's NMR tube, sealed and removed from the glove box. The tube was allowed to stand at 25 °C for 18 hours. The reaction was monitored by NMR spectroscopy. Once the reaction was complete (in situ yield 95%) the NMR tube was returned to the glovebox and the solution was transferred to a 4 mL vial. The solvent was removed *in vacuo*. The oily product was dissolved in *n*-heptane (0.3 mL) and stored at –35 °C. Colourless crystals of the product formed, the mother liquor was decanted and the product **7a** dried *in vacuo*. An isolated yield was obtained from a scaled-up reaction using the same conditions and 15.0 mg (0.0338 mmol) of **1**. Isolated yield: 13 mg (75%). **^{1}H NMR** (400 MHz, C_6D_6, 298 K): δ 7.47 (d, $^3J_{HH}$ = 1.5 Hz, 1H, H^c), 7.13-7.03 (overlapping m, 6H, Dipp-Ar-H), 6.40 (d, $^3J_{HH}$ = 3.1 Hz, 1H, H^a), 6.01 (dd, $^3J_{HH}$ = 1.5 Hz, $^3J_{HH}$ = 3.1 Hz, 1H, H^b), 4.91 (s, 1H, C-H), 4.55 (br, FWHM ≈ 190 Hz, 1H, Al-H), 3.51 (sept, $^3J_{HH}$ = 6.8 Hz, 2H, -CHMe$_2$), 3.24 (sept, $^3J_{HH}$ = 6.8 Hz, 2H, -CHMe$_2$), 1.57 (s, 6H, CH$_3$), 1.45 (d, $^3J_{HH}$ = 6.8 Hz, 6H, Dipp-CH$_3$), 1.16 (overlapping d, $^3J_{HH}$ = 6.8 Hz, 6H, Dipp-CH$_3$), 1.15 (overlapping d, $^3J_{HH}$ = 6.8 Hz, 6H, Dipp-CH$_3$), 0.79 (d, $^3J_{HH}$ = 6.8 Hz, 6H, Dipp-CH$_3$) ppm; **^{13}C{^{1}H} NMR** (100.6 MHz, C_6D_6, 298 K): δ 170.31, 146.27 (s, C-H^c), 144.80, 143.80, 139.44, 127.27, 124.72 (s, C-H^a), 124.38, 124.25, 108.40 (s, C-H^b), 97.00, 28.56, 28.10, 24.77, 24.63, 24.45, 23.49, 22.90 ppm. The C–Al resonance could not be observed due to line-broadening associated with coupling to the quadrapolar I = 5/2 ^{27}Al nucleus.

3. X-ray Crystallographic Data

3.1 Tabulated X-ray Data

Compound	**4a**	**4b**	**4d**
CCDC No.	1973125	1973127	1973129
Formula	$C_{33}H_{45}AlN_2O$	$C_{34}H_{47}AlN_2O$	$2(C_{34}H_{47}AlN_2O_2)$, C_7H_{16}
M	512.69	526.71	1185.62
Crystal System	Monoclinic	Monoclinic	Triclinic
Space group	$P2_1/c$ (14)	$P2_1/c$ (14)	*P*-1 (2)
T [K]	173(2)	173(2)	173(2)
a [Å]	10.9108(2)	18.620(4)	13.6025(8)
b [Å]	12.4443(2)	8.9532(3)	17.2522(10)
c [Å]	22.7523(4)	32.557(7)	17.7358(9)
α [°]	90	90	108.504(5)
β [°]	99.2427(19)	144.73(5)	93.588(5)
γ [°]	90	90	112.988(6)
V [Å^3]	3049.16(11)	3134(2)	3549.6(4)
Z	4	4	2
Density [g cm^{-3}]	1.117	1.116	1.109
Radiation Used	Cu-Kα	Cu-Kα	Mo-Kα
μ (mm^{-1})	0.770	0.760	0.090
θ range [°]	$3.937 \leq \theta \leq 73.494$	$4.111 \leq \theta \leq 73.865$	$2.347 \leq \theta \leq 28.253$
Reflns collected	9780	9853	20817
R_{int}	0.0365	0.0436	0.0341
Completeness	0.987	0.990	0.986
No. of data/restr/param	5819/0/344	6043/0/374	14007/39/884
R_1 [$I > 2\sigma(I)$]	0.0450	0.0494	0.0705
wR_2 [all data]	0.1216	0.1196	0.1886
GoF	1.017	0.967	1.046
Largest diff. pk and hole [eÅ^{-3}]	0.24/-0.30	0.27/-0.27	0.96/-0.43

Table S3.1: Crystal Data, Data Collection and Refinement Parameters for the structures of **4a**, **4b**, **4d**.

Compound	**5b**	**5c**	**5e**
CCDC No.	1973126	1973124	1973123
Formula	$C_{34}H_{47}AlN_2O$	$C_{35}H_{49}AlN_2O$	$C_{33}H_{47}AlN_2O$
M	526.71	540.74	514.70
Crystal System	Orthorhombic	Orthorhombic	Monoclinic
Space group	$Pna2_1$ (33)	$Pna2_1$ (33)	$P2_1/c$ (14)
T [K]	173(2)	173(2)	173(2)
a [Å]	16.1355(5)	16.5281(6)	10.8627(3)
b [Å]	12.4185(4)	12.2177(5)	12.5842(4)
c [Å]	15.8598(5)	15.9821(6)	22.6057(7)
α [°]	90	90	90
β [°]	90	90	97.098(3)
γ [°]	90	90	90
V [Å^3]	3177.96(18)	3227.4(2)	3066.48(16)
Z	4	4	4
Density [g cm^{-3}]	1.101	1.113	1.115
Radiation Used	Mo-Kα	Mo-Kα	Mo-Kα
μ (mm^{-1})	0.091	0.091	0.093
θ range [°]	$2.525 \leq \theta \leq 28.213$	$2.433 \leq \theta \leq 28.198$	$2.432 \leq \theta \leq 28.263$
Reflns collected	7052	7175	10403
R_{int}	0.0300	0.0255	0.0307
Completeness	0.987	0.986	0.987
No. of data/restr/param	4588/1/354	4440/1/364	6108/12/390
R_1 [$I > 2\sigma(I)$]	0.0444	0.0416	0.0509
wR_2 [all data]	0.1056	0.1074	0.1259
GoF	1.029	1.032	1.029
Largest diff. pk and hole [eÅ^{-3}]	0.35/-0.28	0.21/-0.26	0.29/-0.35

Table S3.1: Crystal Data, Data Collection and Refinement Parameters for the structures of **5b**, **5c**, **5e**.

Compound	**5f**	**6b**	**6c**
CCDC No.	1973128	1973114	1973116
Formula	$C_{34}H_{49}AlN_2O$	$C_{28}H_{35}AlN_2O$	$C_{29}H_{37}AlN_2O$
M	528.73	442.56	456.58
Crystal System	Monoclinic	Monoclinic	Monoclinic
Space group	*P*2₁/*c* (14)	*P*2₁ (4)	*C*2/*c* (15)
T [K]	173(2)	173(2)	173(2)
a [Å]	14.3190(4)	8.4130(3)	35.5193(16)
b [Å]	9.8459(3)	16.8783(5)	16.6387(6)
c [Å]	23.1130(7)	18.5811(6)	18.3906(9)
α [°]	90	90	90
β [°]	98.845(3)	97.364(3)	99.240(5)
γ [°]	90	90	90
V [Å^3]	3219.79(16)	2616.70(14)	10727.8(8)
Z	4	4	16
Density [g cm^{-3}]	1.091	1.123	1.131
Radiation Used	Mo-Kα	Mo-Kα	Mo-Kα
μ (mm^{-1})	0.090	0.099	0.098
θ range [°]	$2.599 \leq \theta \leq 28.300$	$2.655 \leq \theta \leq 28.133$	$2.534 \leq \theta \leq 28.244$
Reflns collected	11110	9521	18700
R_{int}	0.0293	0.0197	0.0170
Completeness	0.988	0.985	0.989
No. of data/restr/param	6469/0/353	7755/1/596	10750/66/648
R_1 [$I > 2\sigma(I)$]	0.0466	0.0426	0.0536
wR_2 [all data]	0.1186	0.0960	0.1524
GoF	1.018	1.051	1.030
Largest diff. pk and hole [eÅ^{-3}]	0.32/-0.26	0.20/-0.22	0.40/-0.35

Table S3.1: Crystal Data, Data Collection and Refinement Parameters for the structures of **5f**, **6b**, **6c**.

Compound	**6g**	**7a**
CCDC No.	1973115	1973117
Formula	$C_{33}H_{44}AlN_3O$	$C_{33}H_{45}AlN_2O$
M	525.69	512.69
Crystal System	Triclinic	Orthorhombic
Space group	*P*-1 (2)	$Pna2_1$ (33)
T [K]	173(2)	173(2)
a [Å]	9.1792(4)	15.8366(4)
b [Å]	12.3312(10)	12.5839(4)
c [Å]	13.4571(8)	15.8013(4)
α [°]	100.221(6)	90
β [°]	91.945(4)	90
γ [°]	91.094(5)	90
V [$Å^3$]	1497.69(16)	3148.98(15)
Z	2	4
Density [g cm^{-3}]	1.166	1.081
Radiation Used	Mo-Kα	Mo-Kα
μ (mm^{-1})	0.097	0.090
θ range [deg]	$2.471 \leq \theta \leq 28.268$	$2.572 \leq \theta \leq 28.272$
Reflns collected	8737	17258
R_{int}	0.0305	0.0338
Completeness	0.990	0.986
No. of data/restr/param	5946/75/376	6549/1/348
R_1 [$I > 2\sigma(I)$]	0.0557	0.0411
wR_2 [all data]	0.1376	0.0949
GoF	1.027	1.037
Largest diff. pk and hole [e$Å^{-3}$]	0.27/-0.31	0.20/-0.20

Table S3.1 cont: Crystal Data, Data Collection and Refinement Parameters for the structures of **6g**, **7a**.

Table S3.1 provides a summary of the crystallographic data for the structures of **4a**, **4b**, **4d**, **5b**, **5c**, **5e**, **5f**, **6b**, **6c**, **6g**, **7a**,. Data were collected using Agilent Xcalibur 3 E (**4d**, **5b**, **5c**, **5e**, **5f**, **6b**, **6c**, **6g**, **7a**) and Xcalibur PX Ultra A (**4a**, **4b**) diffractometers, and the structures were refined using the SHELXTL and SHELX-2014 program systems.[7]

[7] SHELXTL v5.1, Bruker AXS, Madison, WI, 1998. SHELX-2014, G.M. Sheldrick, *Acta Cryst.*, 2015, **C71**, 3-8.

3.2 X-ray crystal structures

3.2.1 The X-ray crystal structure of 4a

No evidence of disorder of the oxygen atoms position could be detected.

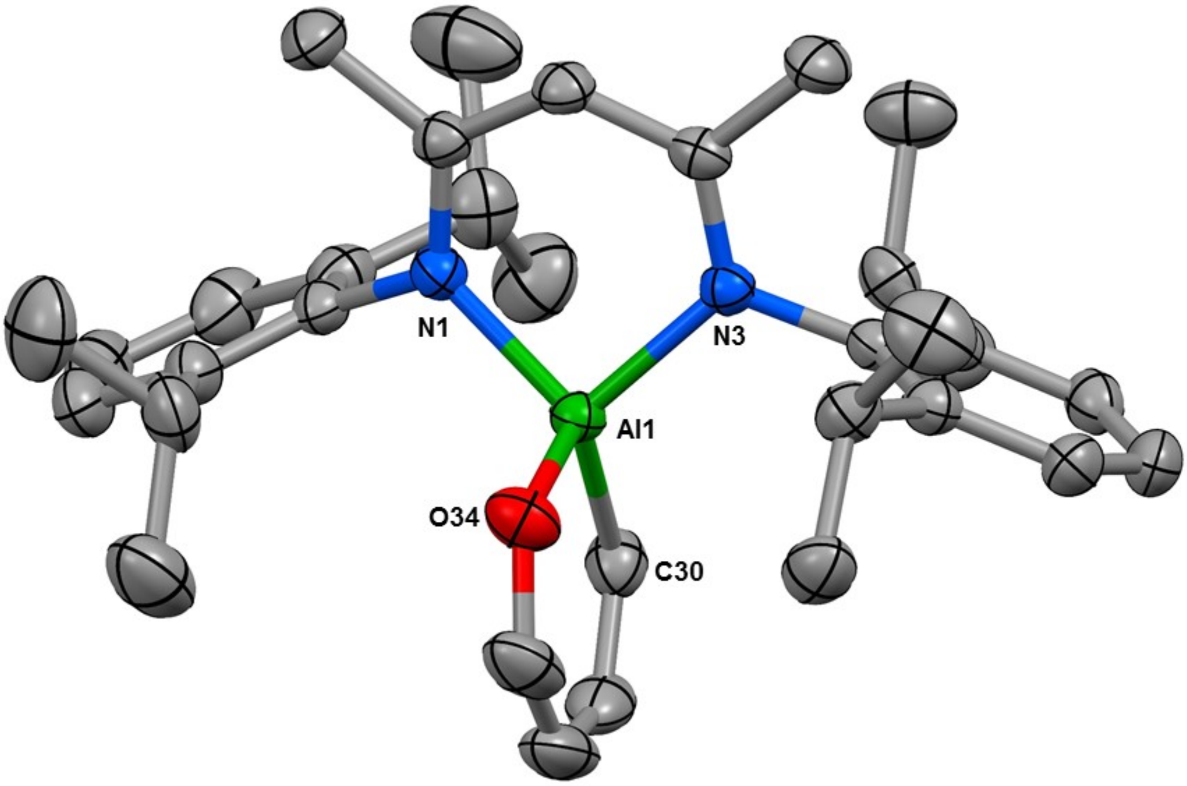

Figure S3.1: The crystal structure of **4a** (50% probability ellipsoids). Hydrogen atoms omitted for clarity.

3.2.2 The X-ray crystal structure of 4b

The C–O activated 2-methylfuran ring is disordered over 2 sites with all disordered atoms lying in the same plane. The occupancy of the disordered component was freely refined and found to be 64:36. Equivalent atoms of the methylfuran ring were described by the EADP command.

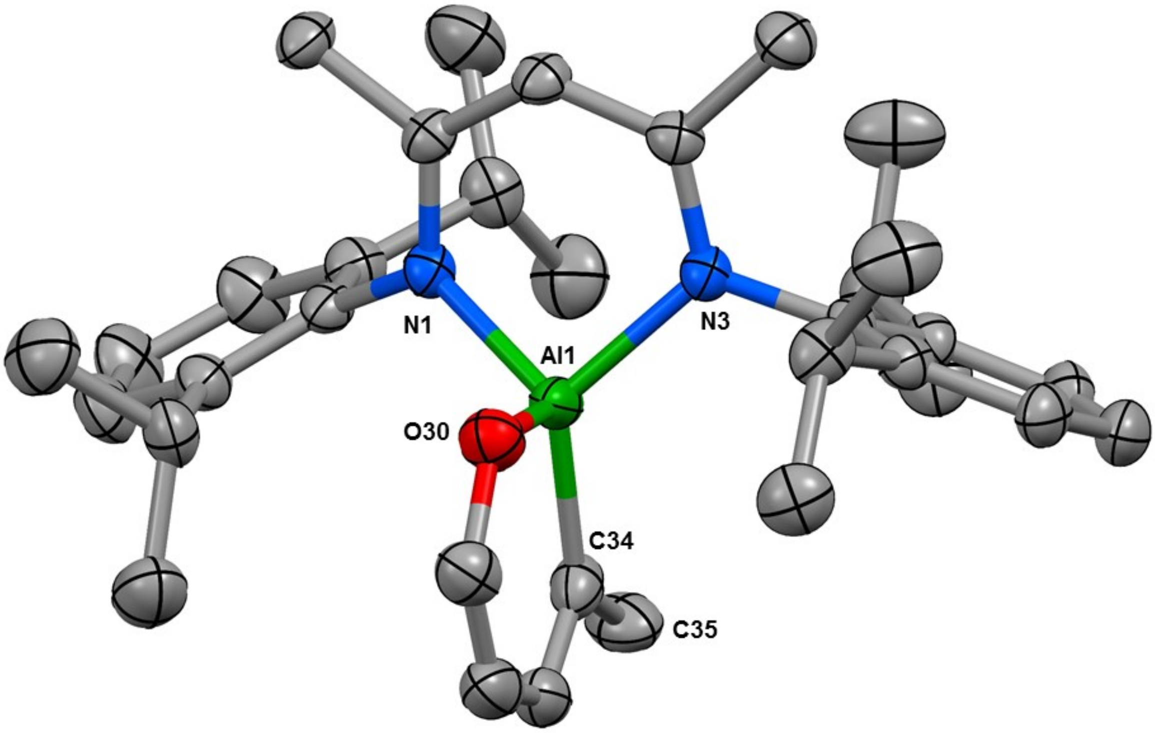

Figure S3.2: The crystal structure of **4b** (50% probability ellipsoids). Hydrogen atoms and minor component of disorder omitted for clarity.

3.2.3 The X-ray crystal structure of 4d

The crystal structure of **4d** contains 2 independent molecules in the asymmetric unit and one molecule of *n*-heptane, the solvent of crystallization. One of the independent molecules contains disorder of the C–O activated 2-methoxyfuran ring over 2 sites. All disordered atoms lie in the same plane. The occupancy of the disordered component was freely refined and found to be 61:39. Interatomic distances in the disordered component we described by the SADI command. 2 carbon atoms in the *n*-heptane molecule were also found to be disordered over 2 sites with the occupancy freely refined and found to be 59:41. The bond lengths of these atoms were described by the DFIX command and the ellipsoids by the ISOR command.

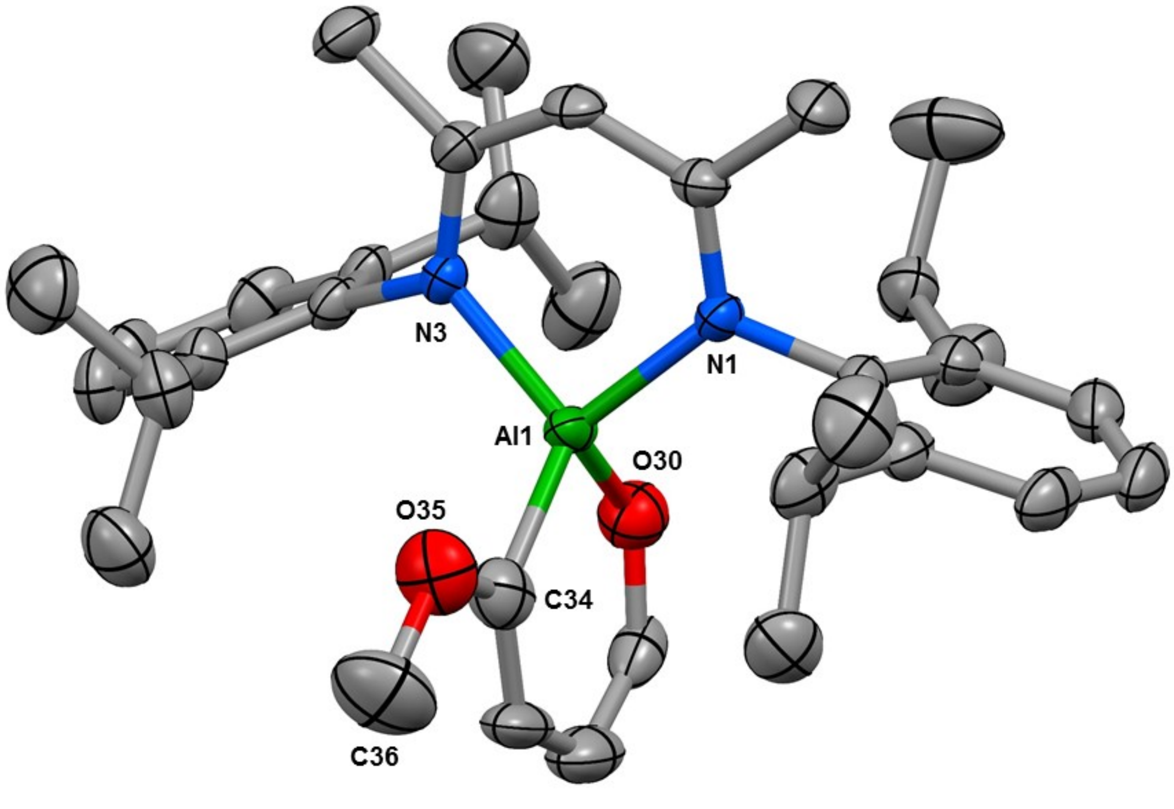

Figure S3.3: The crystal structure of **4d** (50% probability ellipsoids). Hydrogen atoms, second molecule in asymmetric unit and solvent molecule omitted for clarity.

3.2.4 The X-ray crystal structure of 5b

5b crystallised in the non-centrosymmetric space group $Pna2_1$ and the Flack parameter was calculated to be 0.01(14). We are not concerned with the absolute structure of this compound and it is likely that a racemic mixture spontaneously resolved on crystallisation. We did not collect data to accurately determine the absolute structure, hence the Flack parameter may not be accurate, but it is not relevant in this case.

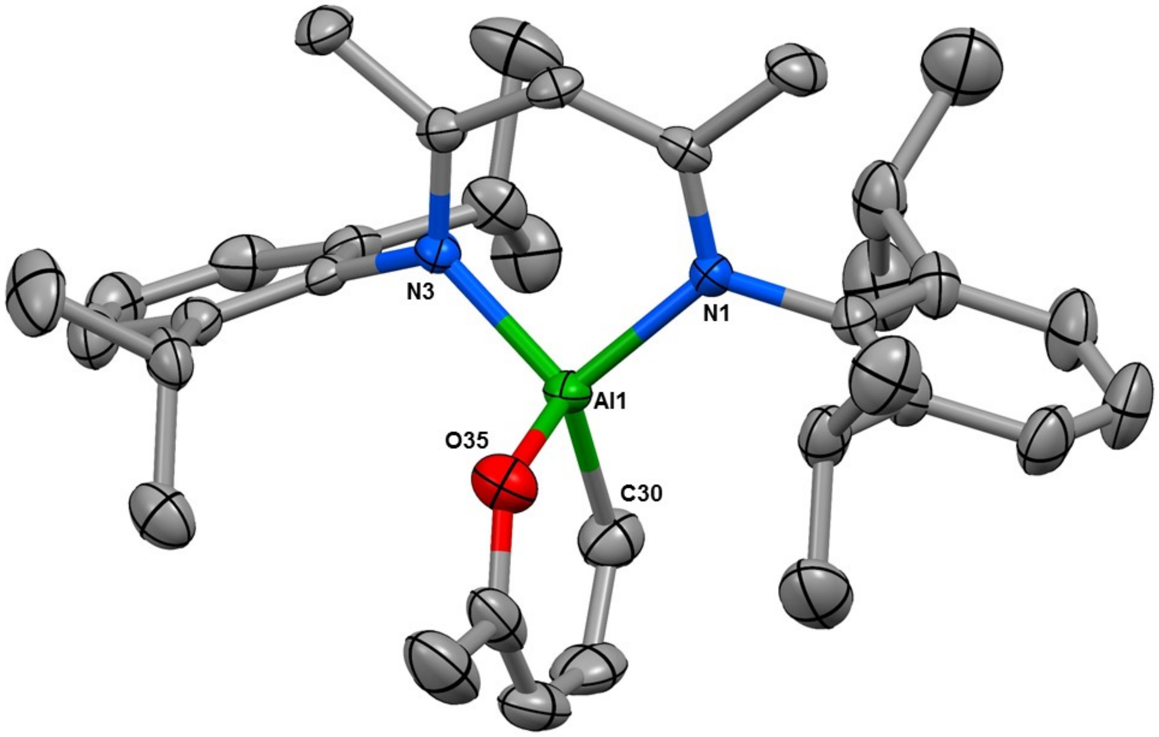

Figure S3.4: The crystal structure of **5b** (50% probability ellipsoids). Hydrogen atoms omitted for clarity.

3.2.5 The X-ray crystal structure of 5c

5c crystallised in the non-centrosymmetric space group $Pna2_1$ and the Flack parameter was calculated to be -0.06(13). We are not concerned with the absolute structure of this compound and it is likely that a racemic mixture spontaneously resolved on crystallisation. We did not collect data to accurately determine the absolute structure, hence the Flack parameter may not be accurate, but it is not relevant in this case.

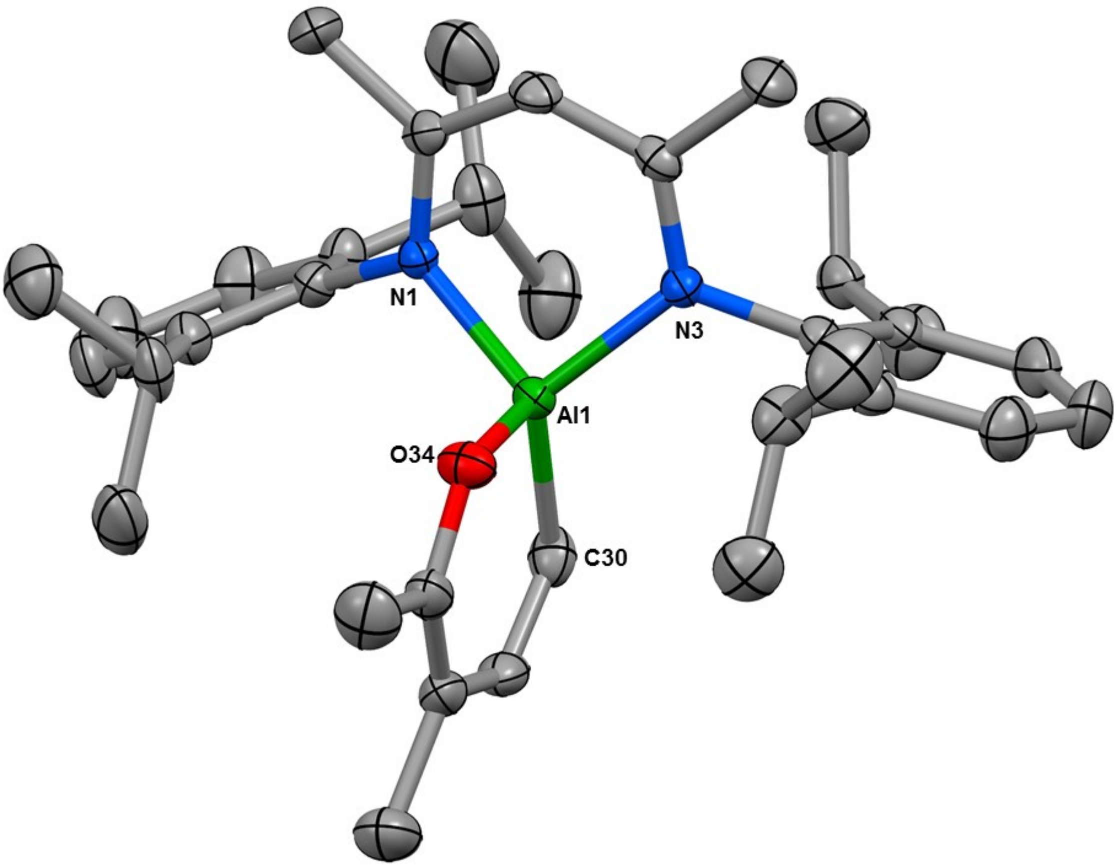

Figure S3.5: The crystal structure of **5c** (50% probability ellipsoids). Hydrogen atoms omitted for clarity.

3.2.6 The X-ray crystal structure of 5e

The C-O activated 2,3-dihydrofuran was found to be disordered over 2 positions. The occupancy of the disordered component was freely refined and found to be 57:43. Interatomic distances in the disordered component were described by the DFIX command.

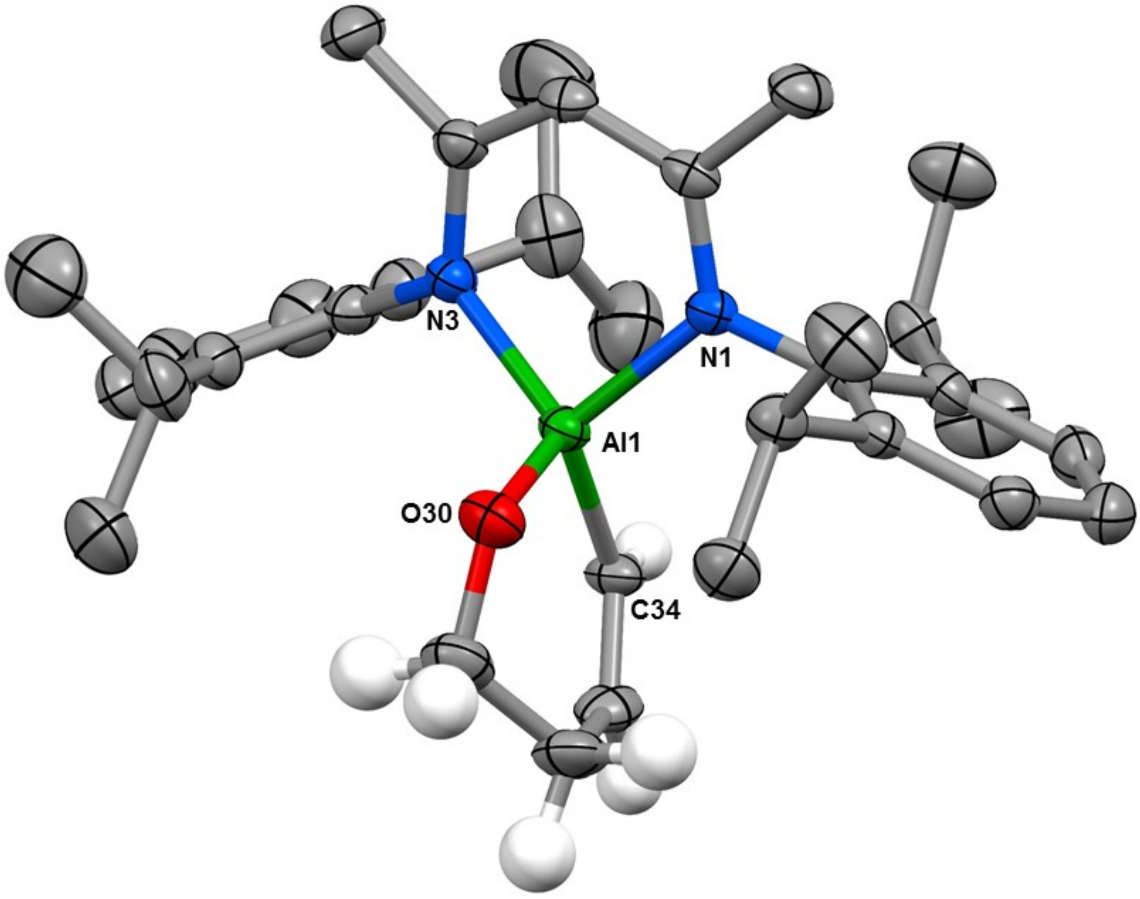

Figure S3.6: The crystal structure of **5e** (50% probability ellipsoids). Selected hydrogen atoms and disordered component omitted for clarity.

3.2.7 The X-ray crystal structure of 5f

The crystal structure of **5f** presented no significant issues.

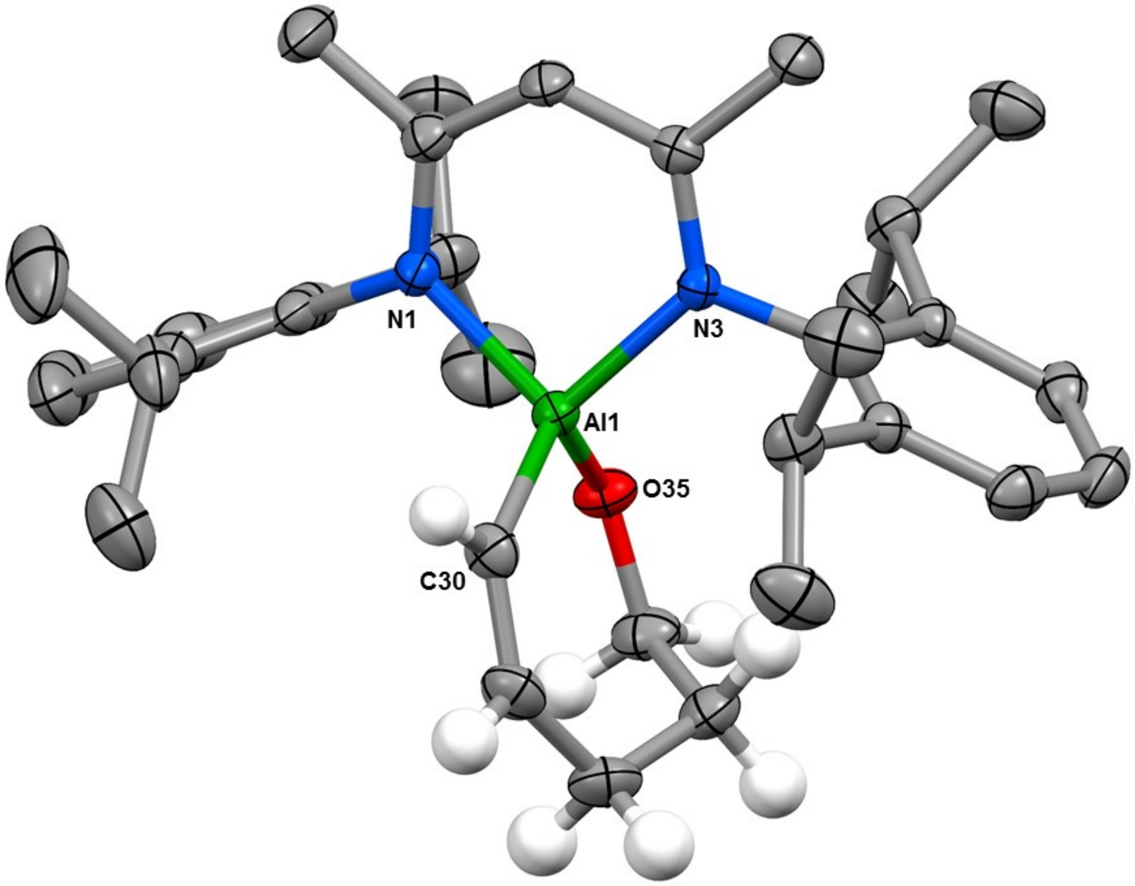

Figure S3.7: The crystal structure of **5f** (50% probability ellipsoids). Selected hydrogen atoms omitted for clarity.

3.2.8 The X-ray crystal structure of 6b

The structure of **6b** was found to contain two independent complexes. **6b** crystallised in the non-centrosymmetric space group $P2_1$ and the Flack parameter was calculated to be -0.13(9). We are not concerned with the absolute structure of this compound and it is likely that a racemic mixture spontaneously resolved on crystallisation. We did not collect data to accurately determine the absolute structure, hence the Flack parameter may not be accurate, but it is not relevant in this case.

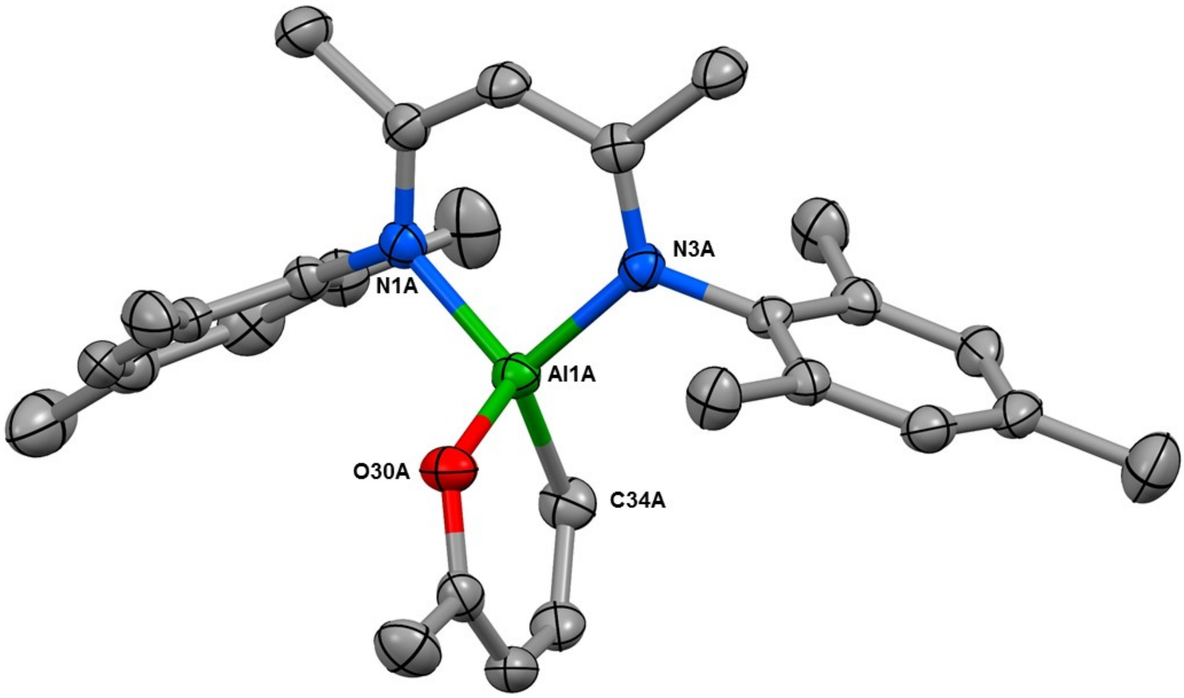

Figure S3.8: The crystal structure of **6b** (50% probability ellipsoids). Hydrogen atoms and second independent molecule omitted for clarity.

3.2.9 The X-ray crystal structure of 6c

The structure of **6c** was found to contain two independent complexes, **6c-A** and **6c-B**. Complex **6c-B** was found to be significantly disordered with two orientations identified for the C_6H_8O ligand and the aluminium centre of *ca*. 89 and 11% occupancy. Their geometries of the two orientations were optimised, the thermal parameters of adjacent atoms were restrained to be similar, and only the non-hydrogen atoms of the major occupancy orientation were refined anisotropically (those of the minor occupancy orientation were refined isotropically).

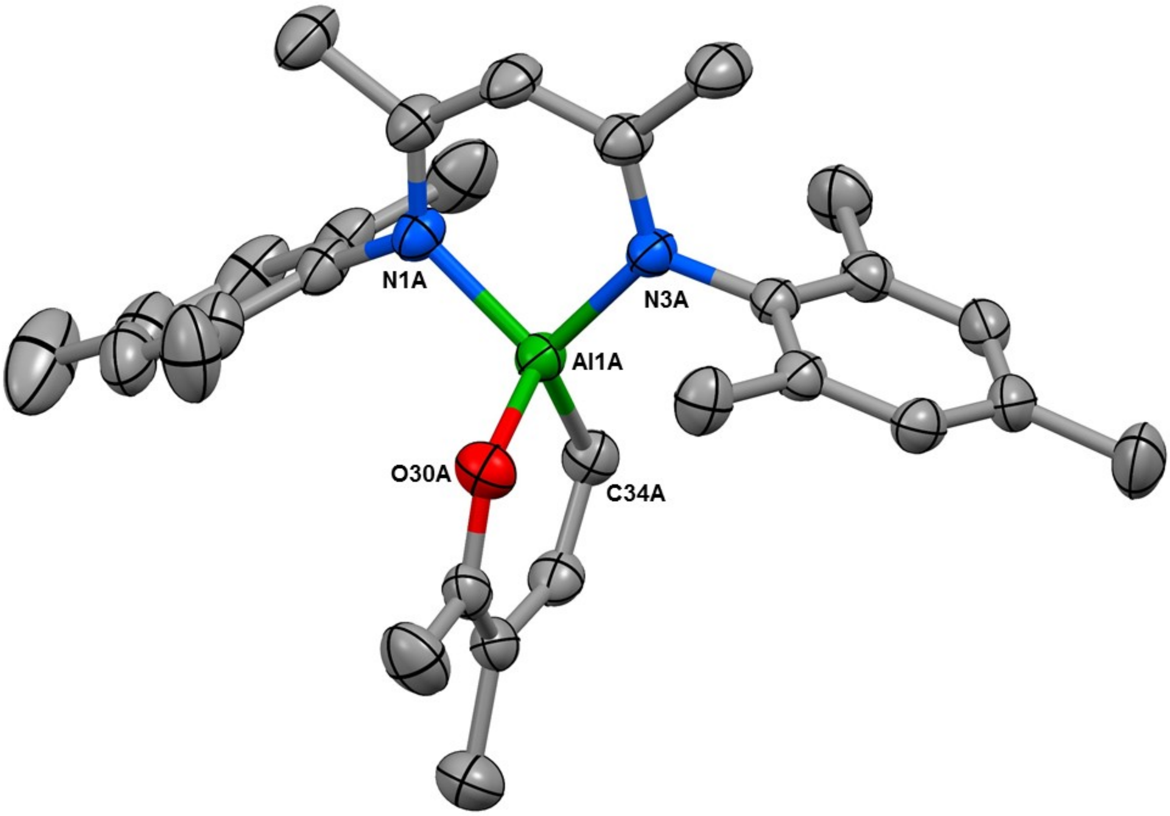

Figure S3.9: The crystal structure of **6c-A** (50% probability ellipsoids). Hydrogen atoms and second independent molecule omitted for clarity.

3.2.10 The X-ray crystal structure of 6g

The N37-based piperidine moiety in the structure of **6g** was found to be disordered. Two orientations were identified of *ca*. 55 and 45% occupancy, their geometries were optimised, the thermal parameters of adjacent atoms were restrained to be similar, and only the non-hydrogen atoms of the major occupancy orientation were refined anisotropically (those of the minor occupancy orientation were refined isotropically).

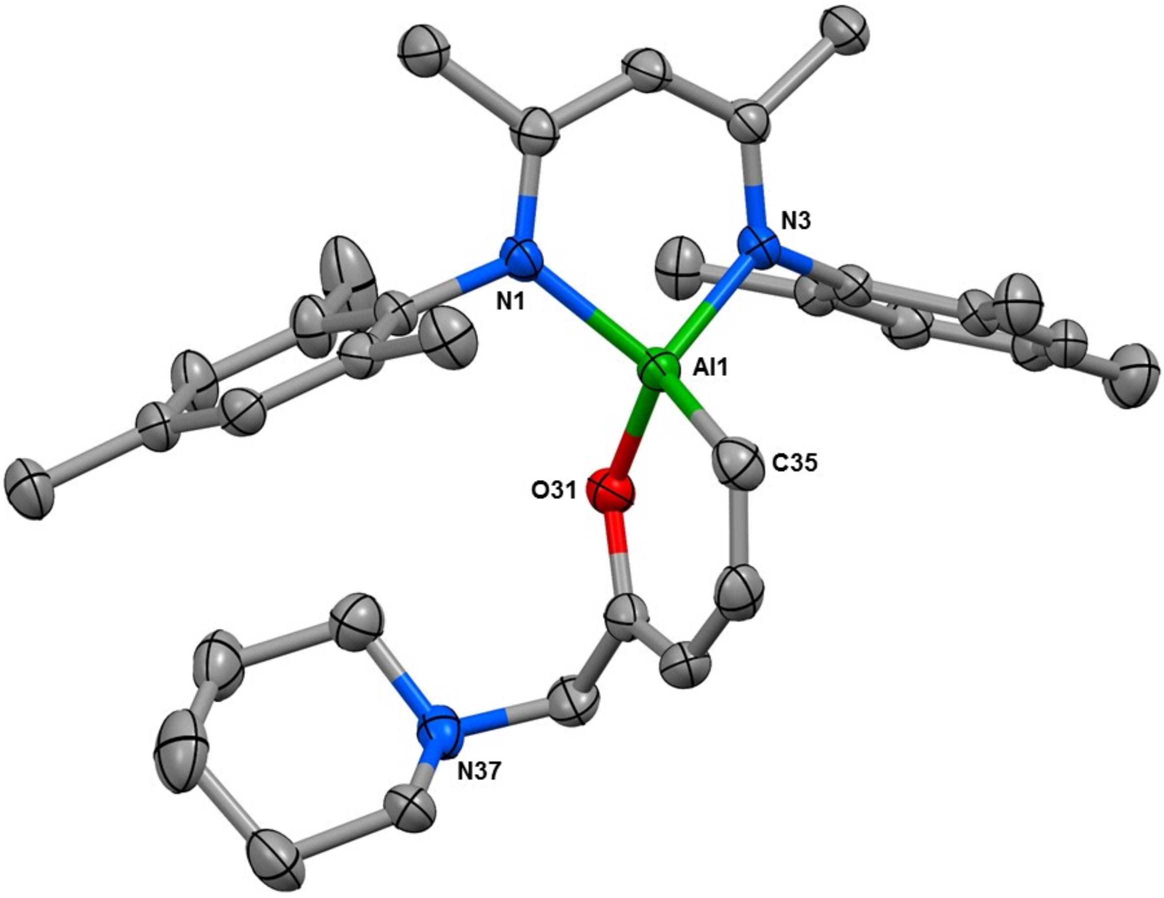

Figure S3.10: The crystal structure of **6g** (50% probability ellipsoids). Hydrogen atoms and minor component of disorder omitted for clarity.

3.2.11 The X-ray crystal structure of 7a

No evidence of disorder of the oxygen atoms position could be detected. The hydrogen atom H1 was located in the difference map and refined freely. **7a** crystallised in the non-centrosymmetric space group $Pna2_1$ and the Flack parameter was calculated to be -0.04(7). We are not concerned with the absolute structure of this compound and it is likely that a racemic mixture spontaneously resolved on crystallisation. We did not collect data to accurately determine the absolute structure, hence the Flack parameter may not be accurate, but it is not relevant in this case.

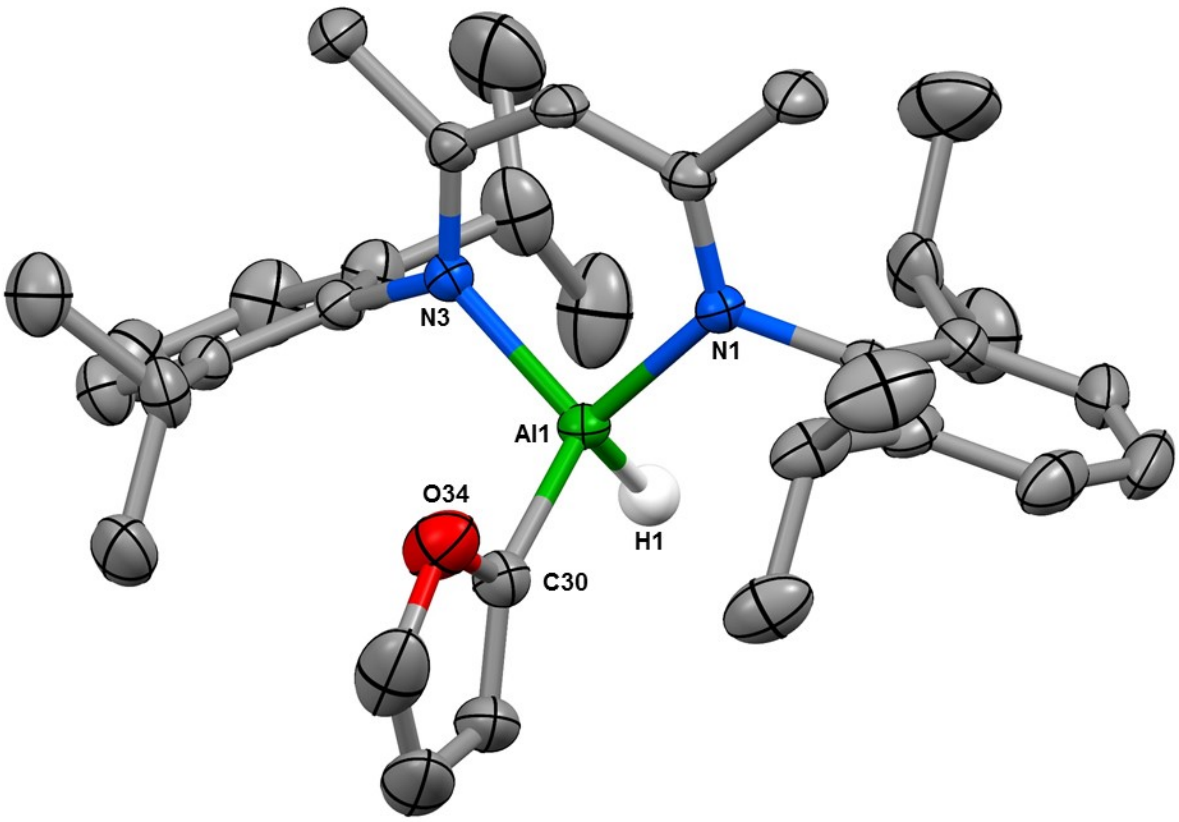

Figure S3.11: The crystal structure of **7a** (50% probability ellipsoids). Selected hydrogen atoms omitted for clarity.

4. Kinetic Studies by NMR Spectroscopy

4.1 Kinetic Studies by NMR Spectroscopy

4.1.1 Kinetic Isotope Effect Measurement for the Uncatalysed Alumination of Furan to form 4a

Experimental Procedure

1 (0.50 mL of 0.0180 M standard solution in C_6H_{12}, 0.0090 mmol) was added to a vial. A solution of furan or furan-D_4 (50.0 μL, 1.80 M standard solution in C_6H_{12}, 0.090 mmol, 10 equiv.) was added. The solution was mixed and transferred to a Young's NMR tube containing a capillary insert standard (ferrocene in C_6D_6). The tube was sealed, removed from the glove box and cooled in ice while transported directly to the NMR spectrometer. The sample was warmed to room temperature and loaded into the spectrometer pre-set at 353 K. The sample was locked to the C_6D_6 standard and shimmed and the first ^{1}H NMR spectrum recorded ~5 min after mixing.

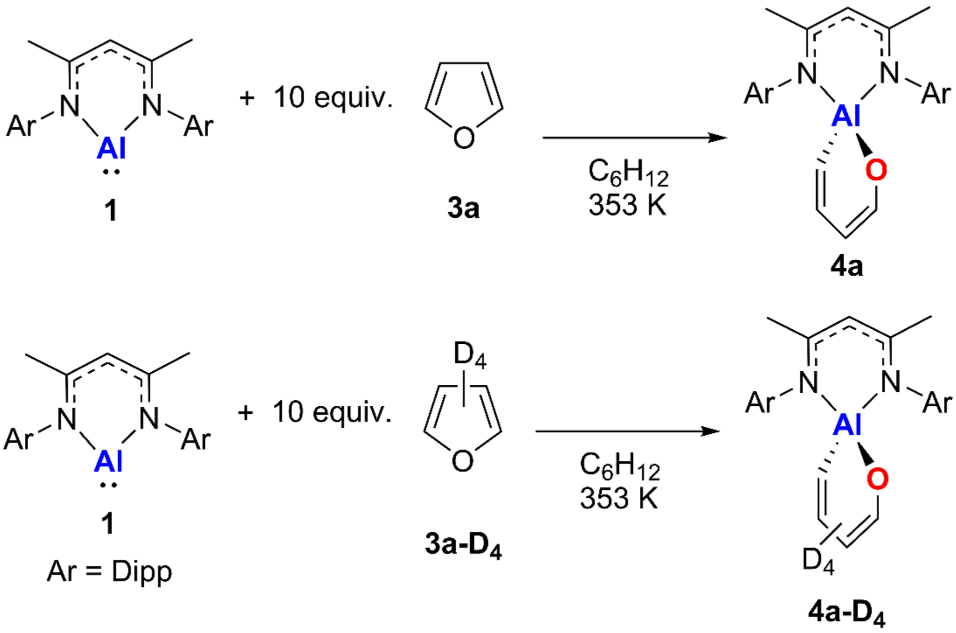

Scheme S4.1: Parallel reactions for KIE measurement for uncatalysed furan alumination by **1**.

A plot of ln [**1**] (determined from initial concentration and integration against internal standard) vs time for both reactions using furan or furan-D_4 indicated the reaction is (pseudo)-first order in [**1**] (figure S4.1). Standard errors were calculated by use of the regression analysis calculation in Microsoft Excel software. The rate constant for the furan reaction was found to be $k_{C\text{-}H}$ = 3.50 x 10^{-4} (± 1.11 x 10^{-5}) s^{-1} and $k_{C\text{-}D}$ = 3.56 x 10^{-4} (± 1.22 x 10^{-5}) s^{-1} for furan-D_4 at 353 K. This gave a KIE of 1.0 (± 0.1) for the reaction.

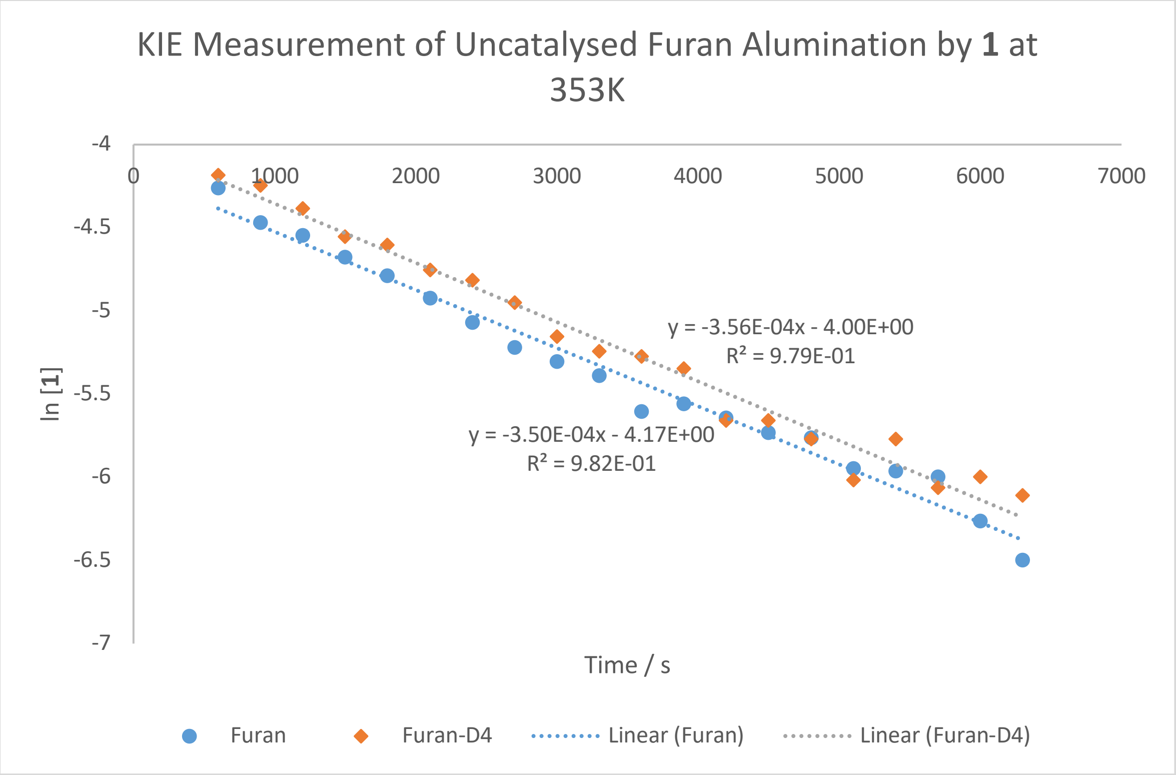

Figure S4.1: Kinetic study of the uncatalysed C–O alumination of furan to determine the kinetic isotope effect.

4.1.2 Eyring Analysis for the Uncatalysed Alumination of Furan to form 4a

Experimental Procedure

1 (0.50 mL of 0.0180 M standard solution in C_6H_{12}, 0.0090 mmol) was added to a vial. A solution of furan (50.0 µL, 1.80 M standard solution in C_6H_{12}, 0.090 mmol, 10 equiv.) was added. The solution was mixed and transferred to a Young's NMR tube containing a capillary insert standard (ferrocene in C_6D_6). The tube was sealed, removed from the glove box and cooled in ice while transported directly to the NMR spectrometer. The sample was warmed to room temperature and loaded into the spectrometer pre-set at the required temperature. The sample was locked to the C_6D_6 standard and shimmed and the first ^{1}H NMR spectrum recorded ~5 min after mixing.

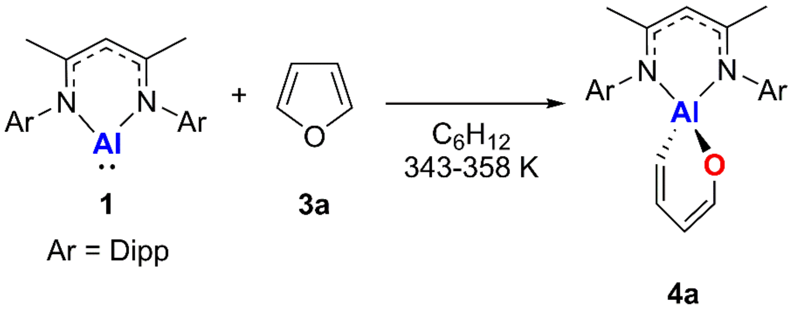

Scheme S4.2: Eyring analysis for uncatalysed furan alumination by **1**.

A plot of ln [**1**] (determined from initial concentration and integration against internal standard) vs time for the reactions at different temperatures showed a linear fits (figure S4.2) indicating the reaction is (pseudo)-first order in [**1**]. The rate constants were calculated (table S4.1) and a plot of ln k vs $1/T$ (figure S4.3) allowed calculation of the thermodynamic parameters using the Eyring equation. Standard errors were calculated by use of the regression analysis calculation in Microsoft Excel software. The enthalpy of activation was found to be $\Delta H^{\ddagger}$ = +19.7 (± 2.7) kcal mol^{-1} and the entropy of activation $\Delta S^{\ddagger}$ = –18.8 (± 7.8) cal K^{-1} mol^{-1}. This gave a value for the Gibbs free energy of activation of $\Delta G^{\ddagger}_{298\ K}$ = +25.3 (± 0.5) kcal mol^{-1}.

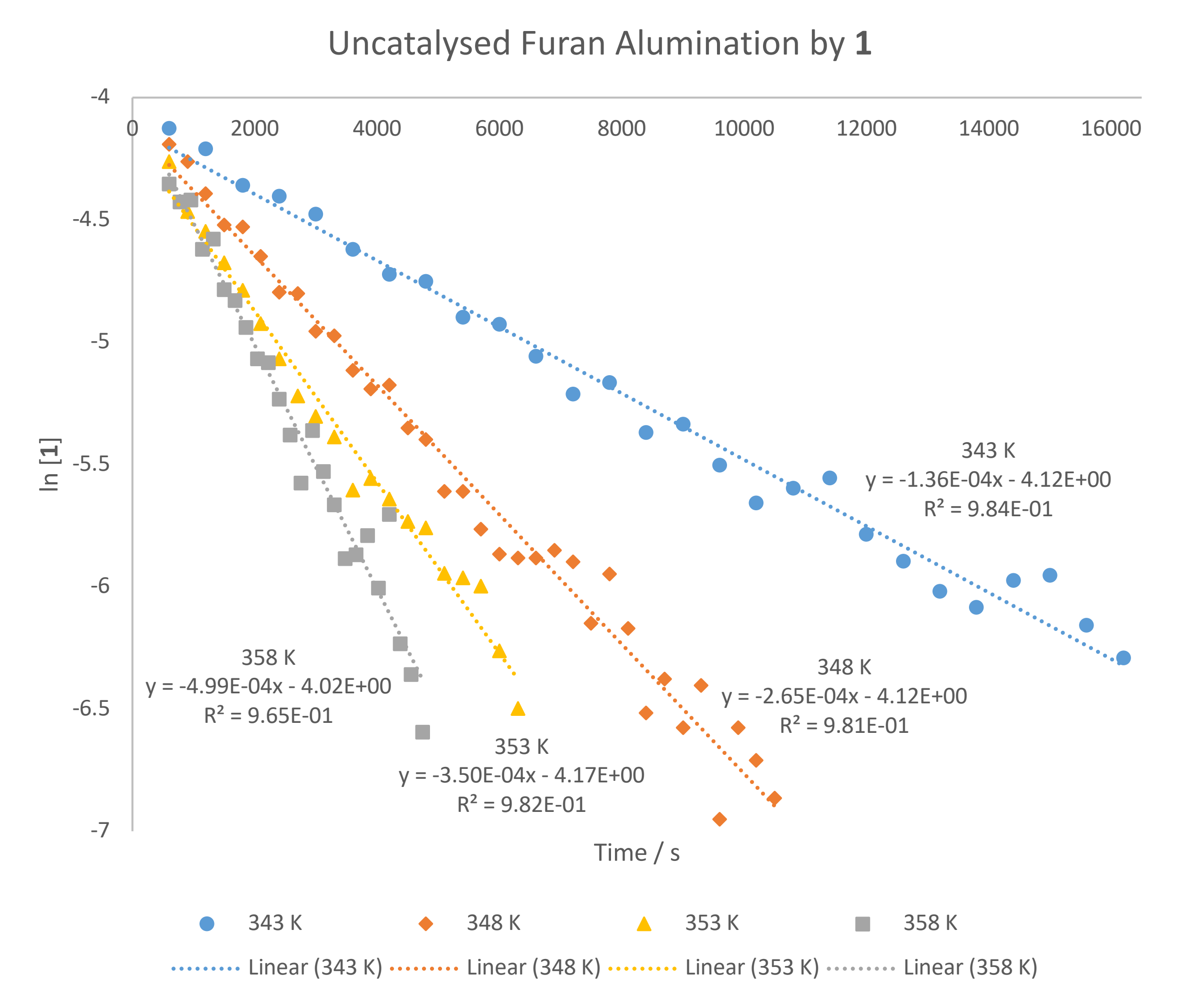

Figure S4.2: Kinetic study of the uncatalysed C–O alumination of furan.

Temperature (K)	k_{obs} (s^{-1})
**343**	1.36 x 10^{-4}
**348**	2.65 x 10^{-4}
**353**	3.50 x 10^{-4}
**358**	4.99 x 10^{-4}

Table S4.1: k_{obs} of the uncatalysed C–O alumination of furan.

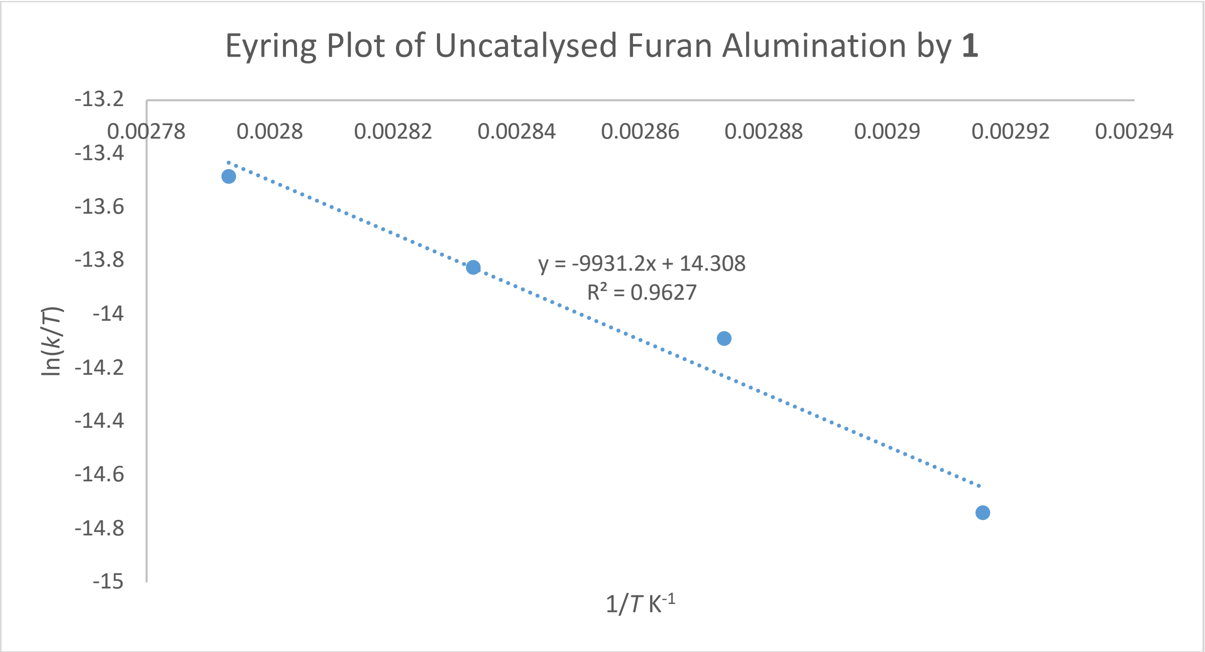

Figure S4.3: Eyring Plot of the uncatalysed C–O alumination of furan.

4.1.3 Kinetic Isotope Effect Measurement for the Palladium Catalysed Alumination of Furan to form 7a

Experimental Procedure

1 (0.40 mL of 0.0225 M standard solution in C_6H_{12}, 0.0090 mmol) was added to a vial. A solution of furan or furan-D_4 (50.0 µL, 1.80 M standard solution in C_6H_{12}, 0.090 mmol, 10 equiv.) was added followed by a solution of [Pd(PCy$_3$)$_2$] (180.0 µL, 0.0025 M standard solution in C_6H_{12}, 0.00045 mmol, 0.05 equiv.). The solution was mixed and transferred to a Young's NMR tube containing a capillary insert standard (ferrocene in C_6D_6). The tube was sealed, removed from the glove box and frozen in liquid nitrogen while transported directly to the NMR spectrometer. The sample was warmed to room temperature and loaded into the spectrometer pre-set at 303 K. The sample was locked to the C_6D_6 standard and shimmed and the first ^{1}H NMR spectrum recorded ~5 min after mixing.

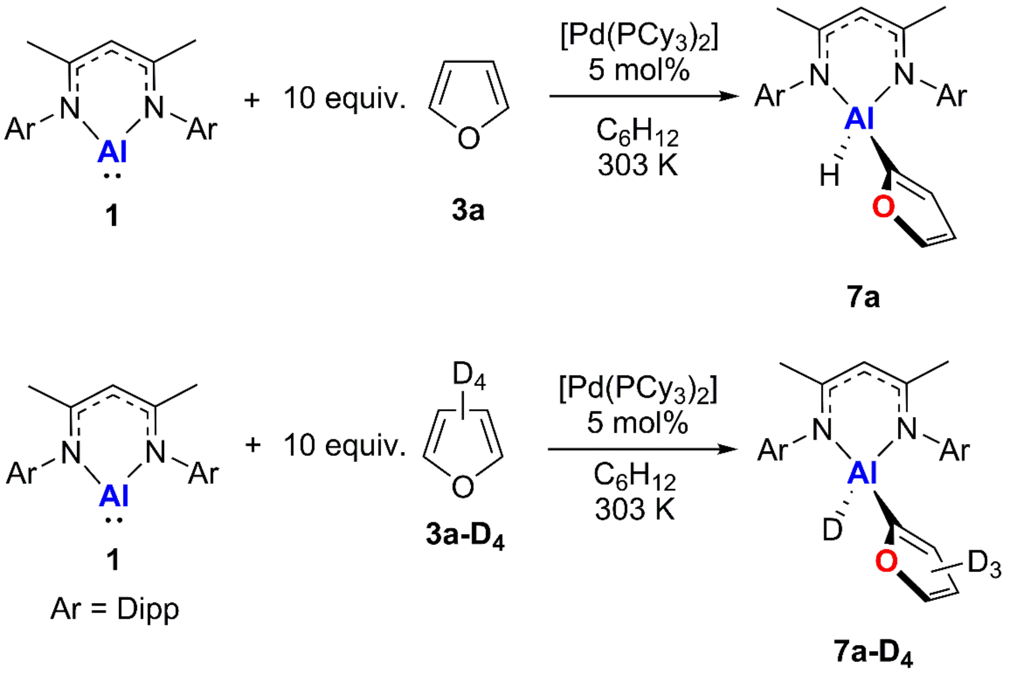

Scheme S4.3: Parallel reactions for KIE measurement for palladium catalysed C–H alumination of furan by **1**.

A plot of [**1**] (determined from initial concentration and integration against internal standard) vs time for both reactions using furan or furan-D_4 indicated the reaction is (pseudo)-zero order in [**1**] (figure S4.4). Standard errors were calculated by use of the regression analysis calculation in Microsoft Excel software. The rate constant for the furan reaction was found to be $k_{C\text{-}H}$ = 2.26 x 10^{-6} (± 6.5 x 10^{-8}) mol dm^{-3} s^{-1} and $k_{C\text{-}D}$ = 4.73 x 10^{-7} (± 1.2 x 10^{-8}) mol dm^{-3} s^{-1} for furan-D_4 at 303 K. This gave a KIE of 4.8 (± 0.3) for the reaction.

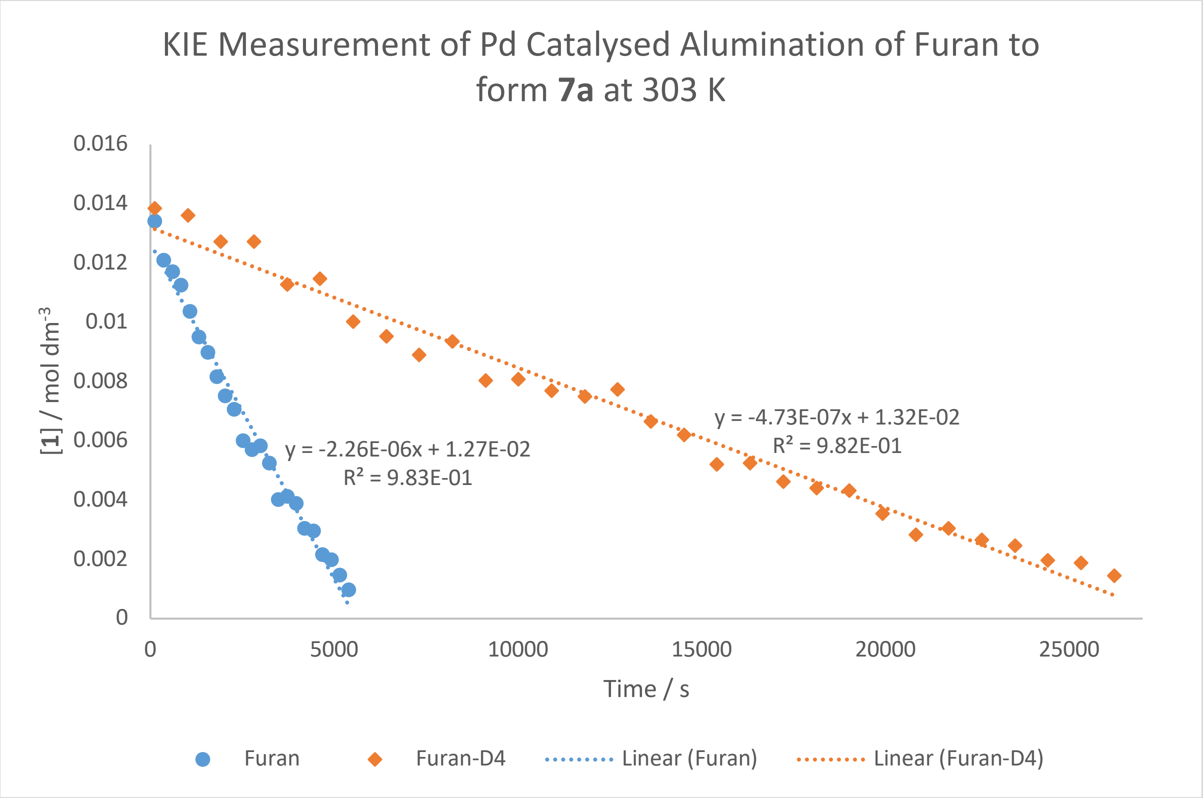

Figure S4.4: Kinetic study of the palladium catalysed C–H alumination of furan to determine the kinetic isotope effect.

4.1.4 Kinetic Isotope Effect Measurement for the Palladium Catalysed Alumination of 7a to form 4a

7a (5.1 mg, 0.010 mmol) or **7a-D$_4$** (5.2 mg, 0.010 mmol) was added to a vial and dissolved in C_6H_{12} (0.35 mL). A solution of [Pd(PCy$_3$)$_2$] (0.20 mL, 0.0025 M standard solution in C_6H_{12}, 0.00050 mmol, 0.05 equiv.) was added. The solution was mixed and transferred to a Young's NMR tube containing a capillary insert standard (ferrocene in C_6D_6). The tube was sealed, removed from the glove box and cooled in ice while transported directly to the NMR spectrometer. The sample was warmed to room temperature and loaded into the spectrometer pre-set at 353 K. The sample was locked to the C_6D_6 standard and shimmed and the first ^{1}H NMR spectrum recorded ~5 min after mixing.

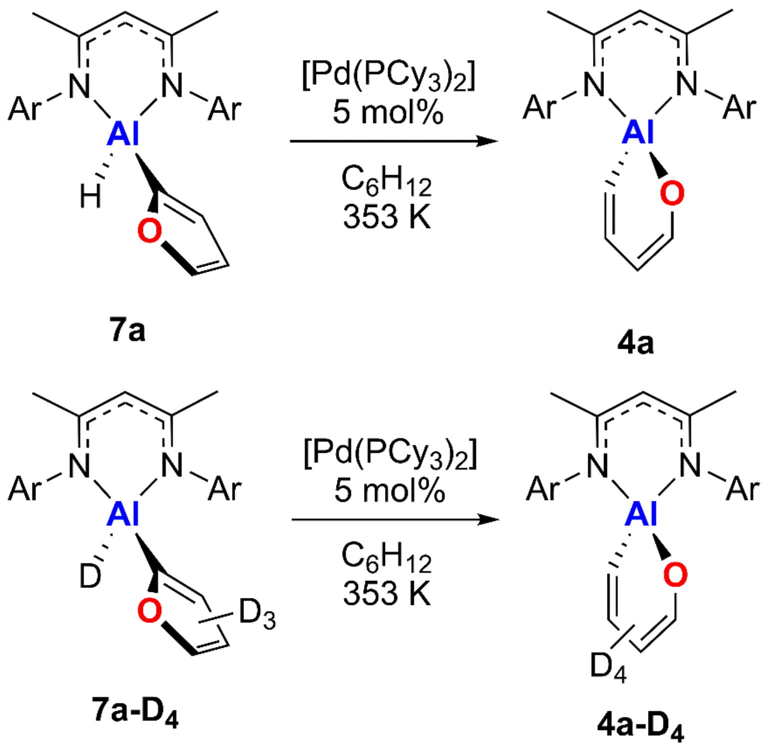

Scheme S4.4: KIE measurement for palladium catalysed conversion of **7a** to **4a**.

A plot of ln [**7a**] or ln [**7a-D$_4$**] (determined from initial concentration and integration against internal standard) vs time for both reactions indicated the reaction is (pseudo)-first order in [**7a**] or [**7a-D$_4$**] (figure S4.5). Standard errors were calculated by use of the regression analysis calculation in Microsoft Excel software. The rate constant for the **7a** reaction was found to be $k_{C\text{-}H}$ = 7.04 x 10^{-5} (± 1.8 x 10^{-6}) s^{-1} and $k_{C\text{-}D}$ = 6.97 x 10^{-5} (± 8.2 x 10^{-7}) s^{-1} for **7a-D$_4$** at 353 K. This gave a KIE of 1.01 (± 0.05) for the reaction.

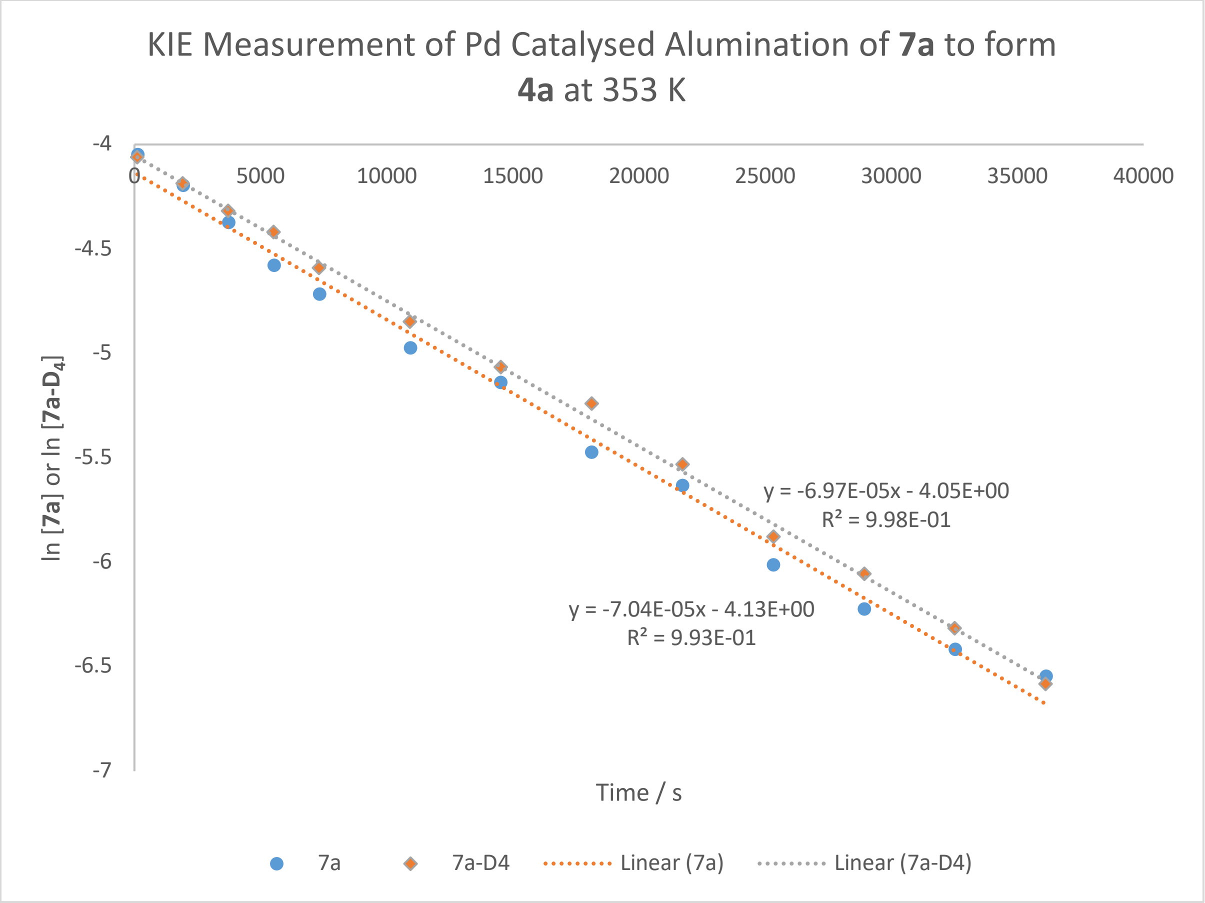

Figure S4.5: Kinetic study of the palladium catalysed C–O alumination of **7a** to determine the kinetic isotope effect.

5. Crossover experiments

5.1 Synthesis of C–H aluminated intermediates via non-catalytic route

Experimental Procedure

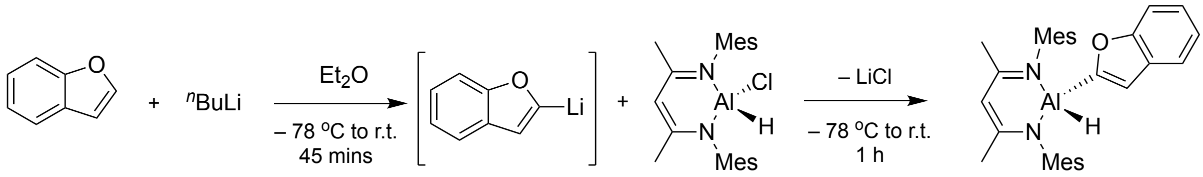

Scheme S5.1: Non-catalytic synthesis of C–H aluminated intermediate for benzofuran.

Synthesised using a previously described method.[8] Under Ar, a solution of ⁿBuLi (0.32 mL, 1.6 M in hexane, 0.50 mmol) was added dropwise to a stirred solution of benzofuran (65.0 mg 0.55 mmol) in Et_2O (10 mL) at -78 °C. The mixture was allowed to warm to 25 °C and then stirred for a further 45 mins. The reaction mixture was then cooled to -78 °C again. A solution of Mes-*BDI*AlHCl (200 mg, 0.50 mmol) in Et_2O (10 mL) was added dropwise. The reaction mixture was allowed to warm to 25 °C and stirred for a further 1 h at this temperature. The solvent was removed *in vacuo* to generate a white oily solid. The solid was then dissolved in *n*-hexane and stirred for 20 mins before filtration. The solvent volume was reduced to ~4 mL and the mixture stored at -35 °C to afford colourless crystalline solid. A further recrystallisation from *n*-hexane at -35 °C was performed followed by a cold *n*-hexane wash (1 mL) to purify the product.

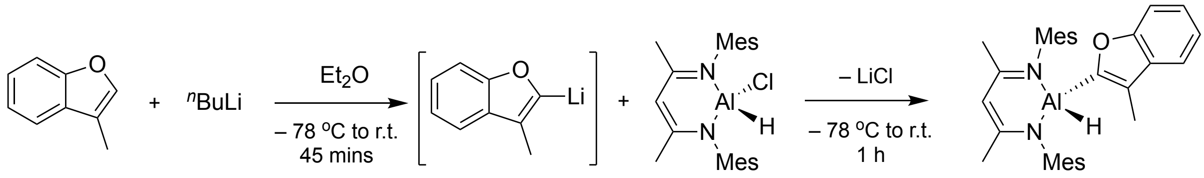

Scheme S5.2: Non-catalytic synthesis of C–H aluminated intermediate for 3-methylbenzofuran.

Synthesised as outlined in the previous procedure, using 3-methylbenzofuran (73.3 mg, 0.55 mmol). White crystalline solid was isolated by recrystallisation from n-hexane at -35 °C.

[8] Chen W., Hooper T. N., Ng J., White A. J. P., Crimmin M. R. *Angew. Chem. Int. Ed.*, 2017, **56**, 12687–12691

Complex S1

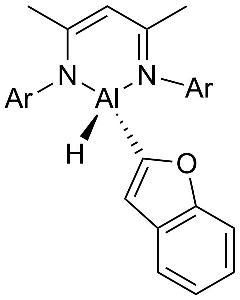

Ar = Mes

Isolated as white crystals. Yield: 108 mg, 0.226 mmol, 45%. Agreement with literature characterisation.[6]

Complex S2

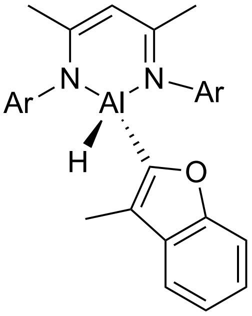

Ar = Mes

Isolated as white crystals. **^{1}H NMR** (400 MHz, C_6D_6, 298 K): δ 7.55 (d, $^3J_{HH}$ = , 1H), 7.34 (d, $^3J_{HH}$ = , 1H), 7.18 – 7.01 (m, 4H), 6.75 (s, 2H, Mes-Ar-H), 6.55 (s, 2H, Mes-Ar-H), 4.95 (s, 1H, C–H), 4.86 (br, Al–H), 2.39 (s, 6H, Mes-CH_3), 2.09 (s, 3H, CH_3), 2.02 (s, 6H, Mes-CH_3), 2.00 (s, 6H, Mes-CH_3), 1.52 (s, 6H, CH_3).

5.2 Crossover experiment from (C-H intermediate) to determine inter or intra-molecular rearrangement to C–O aluminated products.

Experimental Procedure

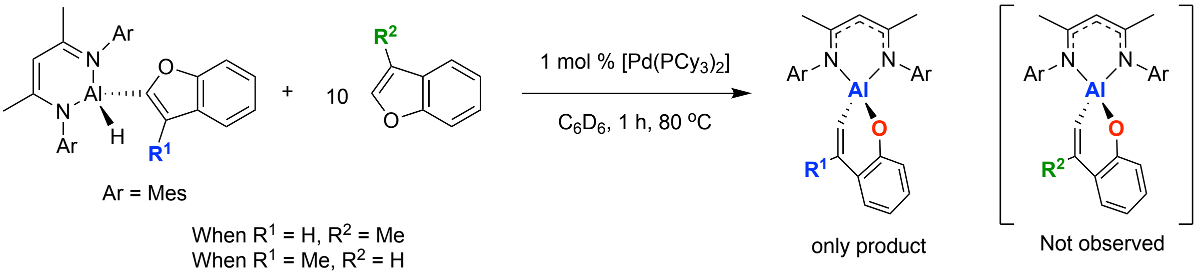

Scheme S5.3: Forward and reverse crossover experiments.

In a glovebox, the aluminated complex **(S1** or **S2**) (10 mg, 0.02 mmol) was dissolved in C_6D_6 (0.60 mL) in a 4 mL scintillation vial. 10 equiv. of the corresponding benzofuran for the cross-over experiment was added **(S1** with 3-methylbenzofuran**, S2** with benzofuran). The solution was transferred to a J Young's tube and a solution of [Pd(PCy_3)$_2$] (1 mol% corresponding to

4.05 μL of a 0.05 M solution in C_6D_6, 0.0002 mmol) was added using a micropipette. The NMR tube was removed from the glovebox and heated to 80 °C. After 1 h the major product resulting from intramolecular rearrangement was the only product observed. In both cases, no crossover product was observed.

6. Computational details

6.1 Methods

The geometries of products were optimised with the M06L DFT functional using the Gaussian09 program package.[9] Stationary points were characterised depending on their imaginary frequencies (0 for minima and 1 for TSs). NBO analysis was performed using the NBO 6.0 version program.[10] The ωB97x hybrid exchange-correlation DFT functional, the B3PW91 functional and the M062X Minnesota DFT functional were also employed to assess differences in performance arising from the level of theory.

The SDD effective core potential was used for Pd and Al (SDDAll). The split-valence 6-31G** basis set was used for C, H, N, P and O atoms. The default numerical integration grid was also improved using a pruned grid with 99 radial shells and 590 angular points per shell (int=ultrafine). Intrinsic Reaction Coordinate (IRC) calculations were used to connect transition states and minima located on the potential energy surface to give a full potential energy profile.

Uncatalysed: For the uncatalysed route the pathways presented herein were calculated with M06L and include dispersion and solvent corrections. ***Catalysed pathway:*** For the catalysed route the pathways were calculated with M06L but do not include dispersion and solvent corrections unless otherwise stated.

Dispersion effects were included via single point energy corrections and were modelled using ωB97xD functional for ωB97x, using Grimme's D3 correction for M06L and M062X (EmpiricalDispersion=GD3)[11] and Grimme's D3 correction with Becke-Johnson damping (EmpiricalDispersion=GD3BJ) for B3PW91.

Solvent effects were included via single point energy corrections (benzene, ε = 2.2706, cyclohexane, ε = 2.0165) and were modelled using the polarizable continuum model (PCM) to free energies for M06L.

---

[9] Frisch, M. J.; Trucks, G. W.; Schlegel, H. B.; Scuseria, G. E.; Robb, M. A.; Cheeseman, J. R.; Scalmani, G.; Barone, V.; Mennucci, B.; Petersson, G. A.; Nakatsuji, H.; Caricato, M.; Li, X.; Hratchian, H. P.; Izmaylov, A. F.; Bloino, J.; Zheng, G.; Sonnenberg, J. L.; Hada, M.; Ehara, M.; Toyota, K.; Fukuda, R.; Hasegawa, J.; Ishida, M.; Nakajima, T.; Honda, Y.; Kitao, O.; Nakai, H.; Vreven, T.; Montgomery, J. A., Jr.; Peralta, J. E.; Ogliaro, F.; Bearpark, M.; Heyd, J. J.; Brothers, E.; Kudin, K. N.; Staroverov, V. N.; Kobayashi, R.; Normand, J.; Raghavachari, K.; Rendell, A.; Burant, J. C.; Iyengar, S. S.; Tomasi, J.; Cossi, M.; Rega, N.; Millam, J. M.; Klene, M.; Knox, J. E.; Cross, J. B.; Bakken, V.; Adamo, C.; Jaramillo, J.; Gomperts, R.; Stratmann, R. E.; Yazyev, O.; Austin, A. J.; Cammi, R.; Pomelli, C.; Ochterski, J. W.; Martin, R. L.; Morokuma, K.; Zakrzewski, V. G.; Voth, G. A.; Salvador, P.; Dannenberg, J. J.; Dapprich, S.; Daniels, A. D.; Farkas, Ö.; Foresman, J. B.; Ortiz, J. V.; Cioslowski, J.; Fox, D. J. *Gaussian 09, Revision D.01*; Gaussian, Inc., Wallingford, CT, 2009.

[10] *NBO 6.0*. Glendening, E. D.; Badenhoop, J. K.; Reed, A. E.; Carpenter, J. E.; Bohmann, J. A.; Morales, C. M.; Landis, C. R.; Weinhold, F. Theoretical Chemistry Institute, University of Wisconsin, Madison (2013).

[11] Grimme, S.; Antony, J.; Ehrlich, S.; Krieg, H. *J Chem. Phys.* 2010, **132**, 154104.

6.2 Uncatalysed C–O bond alumination of furans: Mechanism

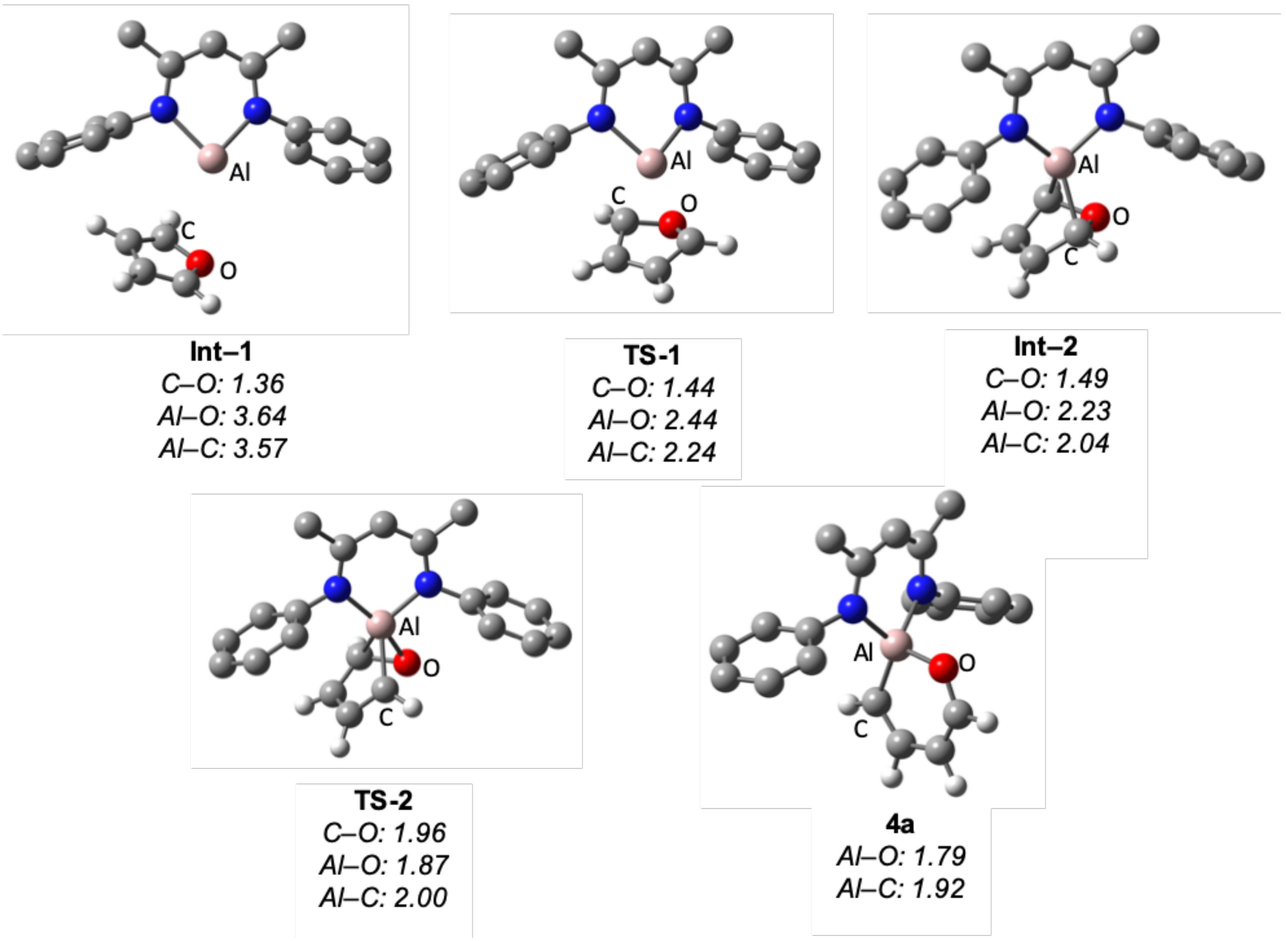

Figure S6.1: Selected bond lengths (in Å) for **Int-1** to **4a**. *i*-Pr groups and some hydrogens have been omitted for clarity.

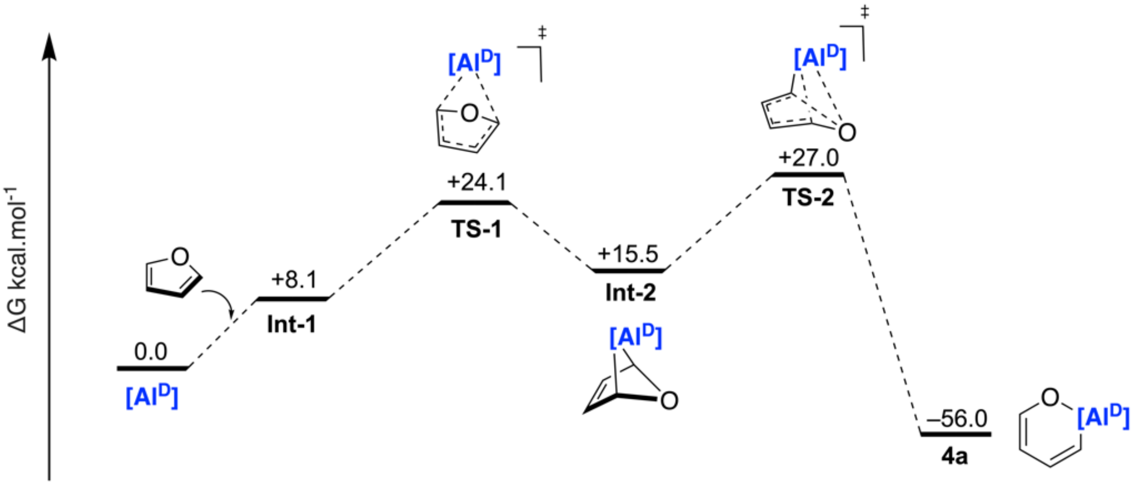

Figure S6.2: Calculated pathway for C–O alumination of furan from **1**. [Al^{D}] represents the Dipp-*BDI*Al fragment.

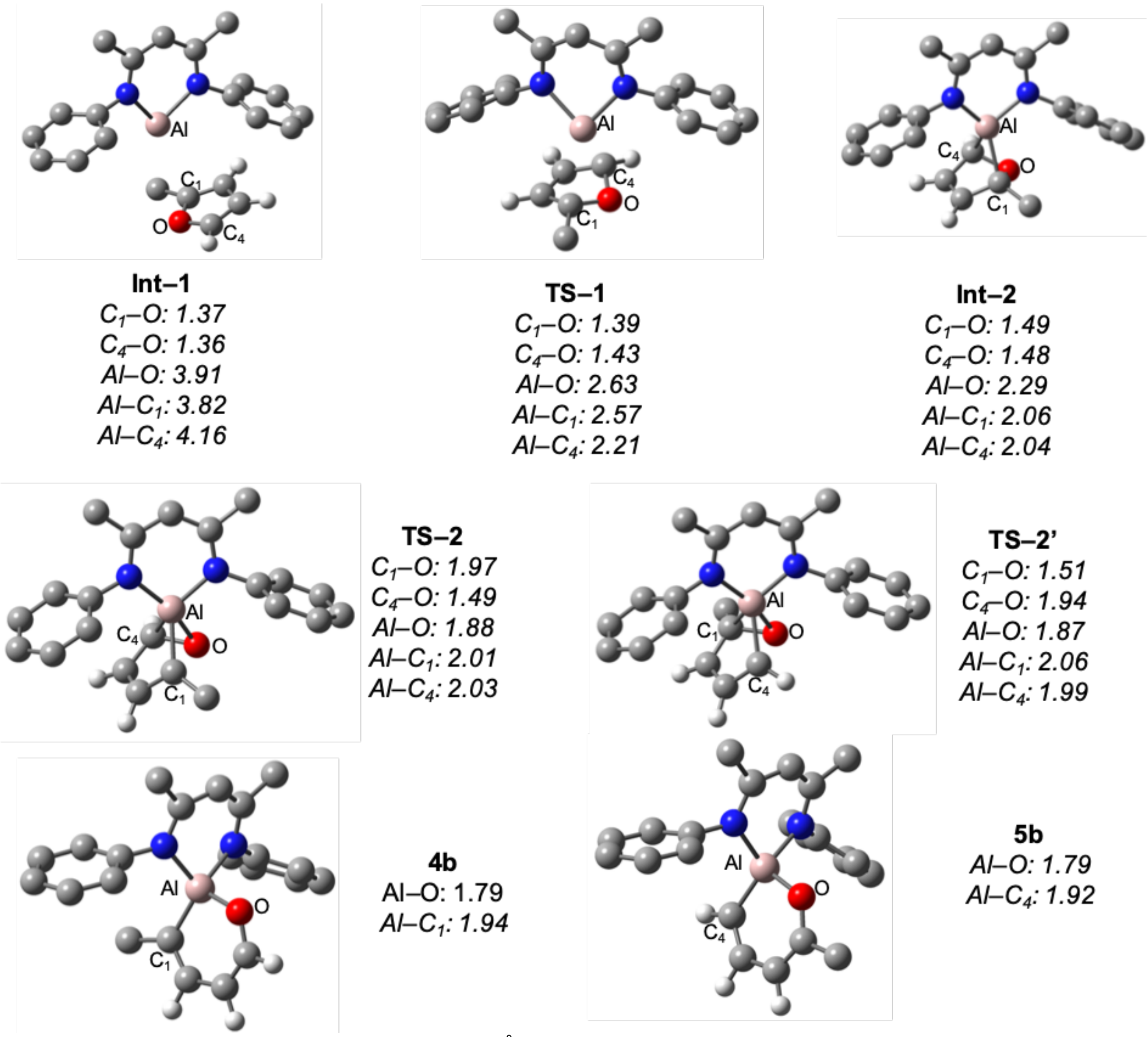

Figure S6.3: Selected bond lengths (in Å). *i*-Pr groups and some hydrogens have been omitted for clarity.

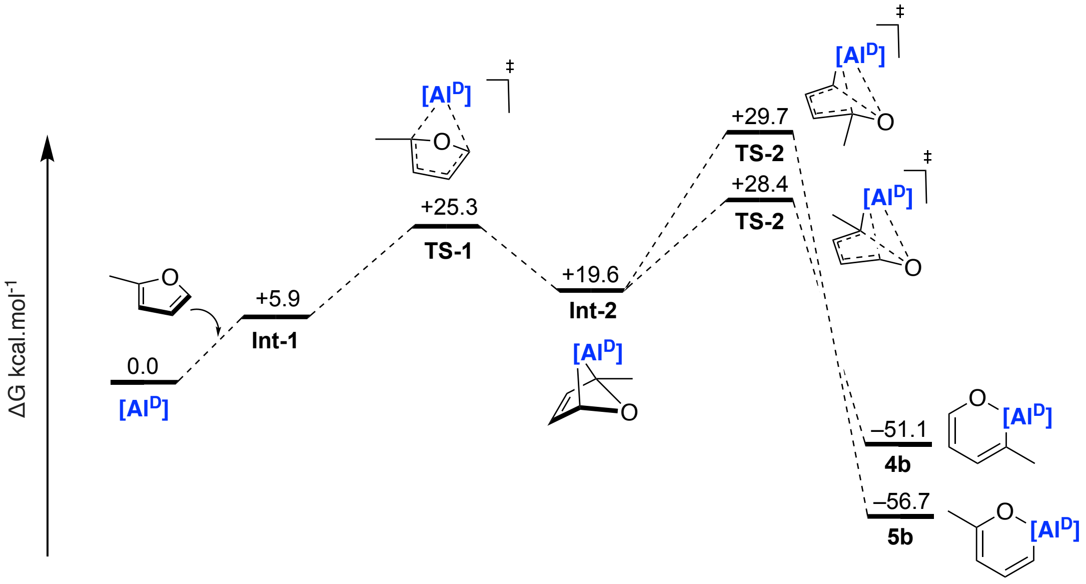

Figure S6.4: Calculated pathway for C–O alumination of 2-methylfuran from **1**. [Al^{D}] represents the Dipp-*BDI*Al fragment.

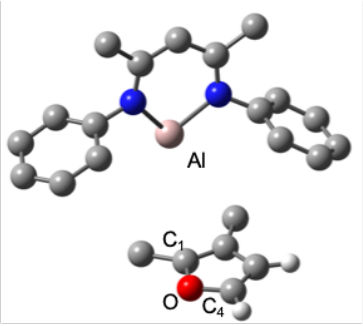

Int–1
C_1–O: 1.37
C_4–O: 1.36
Al–O: 4.90
Al–C_1: 4.01
Al–C_4: 5.37

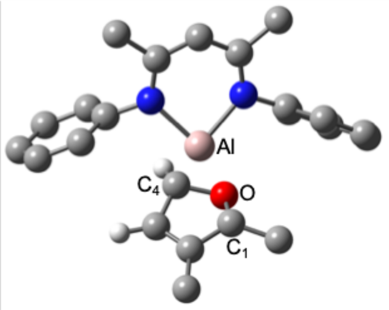

TS–1
C_1–O: 1.39
C_4–O: 1.44
Al–O: 2.48
Al–C_1: 2.55
Al–C_4: 2.20

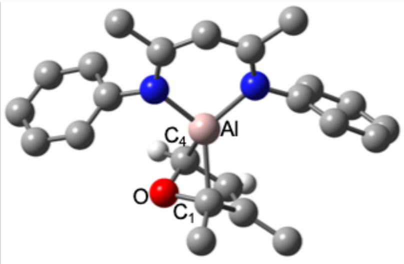

Int–2
C_1–O: 1.49
C_4–O: 1.49
Al–O: 2.25
Al–C_1: 2.07
Al–C_4: 2.03

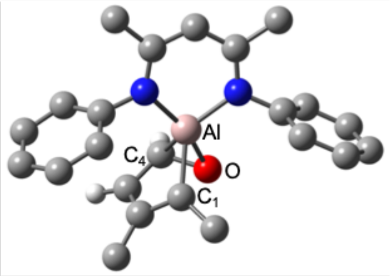

TS–2
C_1–O: 2.00
C_4–O: 1.49
Al–O: 1.87
Al–C_1: 2.01
Al–C_4: 2.03

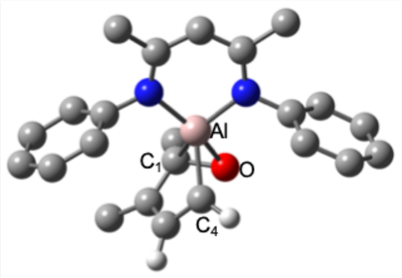

TS–2’
C_1–O: 1.51
C_4–O: 1.95
Al–O: 1.87
Al–C_1: 2.06
Al–C_4: 1.99

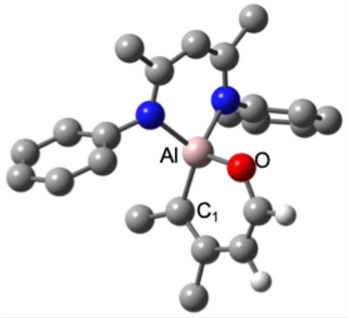

4c
Al–O: 1.79
Al–C_1: 1.93

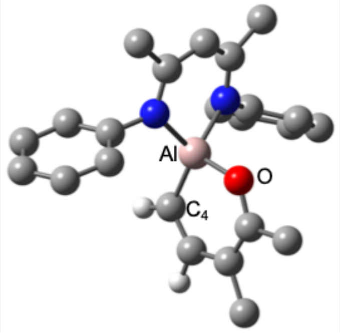

5c
Al–O: 1.78
Al–C_4: 1.91

Figure S6.5: Selected bond lengths (in Å). *i*-Pr groups and some hydrogens have been omitted for clarity.

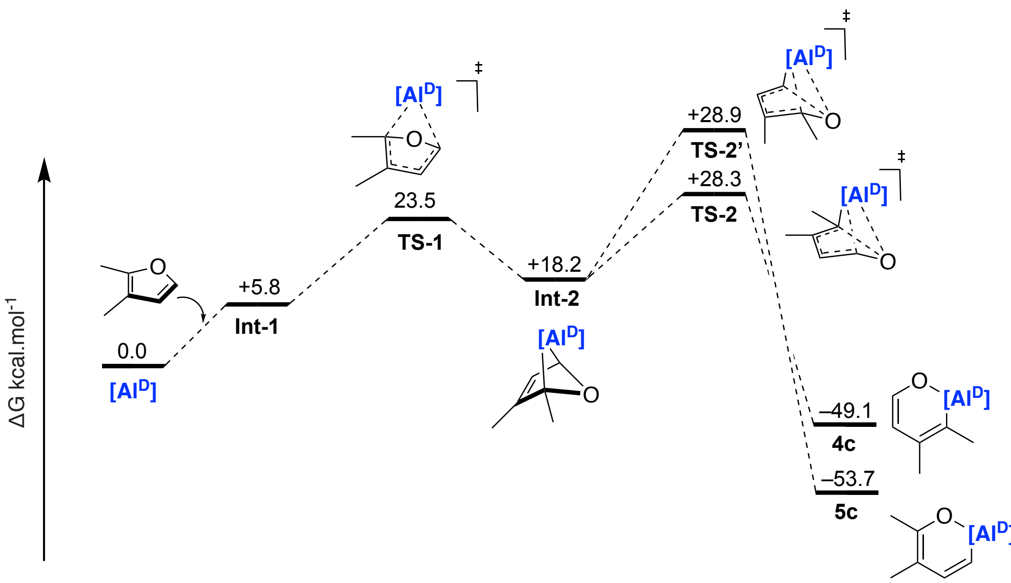

Figure S6.6: Calculated pathway for C–O alumination of 2,3-dimethylfuran from **1**. [Al^{D}] represents the Dipp-*BDI*Al fragment.

The calculations on 2,3-dimethylfuran (Figure 6.6) do not accurately predict the selectivity of the reaction. Experimentally **4c** and **5c** where observed in a 1:9 ratio. The energy difference between **TS-2** and **TS-2'** ($\Delta G^{\ddagger}$ = +0.6 kcal mol^{-1}) combined with the accuracy of the DFT method is small enough to consider these pathways to be competitive. To gain further insight into the role of sterics the reaction of **1** with 3-methylfuran was calculated. In this case there is a clear selectivity switch to favour the least hindered transition state (**TS-2**, $\Delta G^{\ddagger}$ = +2.4 kcal mol^{-1}, Figure 6.7). Based on this result it is likely that for 2,3-dimethylfuran, with substituents in both the 2 and 3 position, an interplay between steric and electronic effects is in operation that is challenging to accurately predict by DFT calculations.

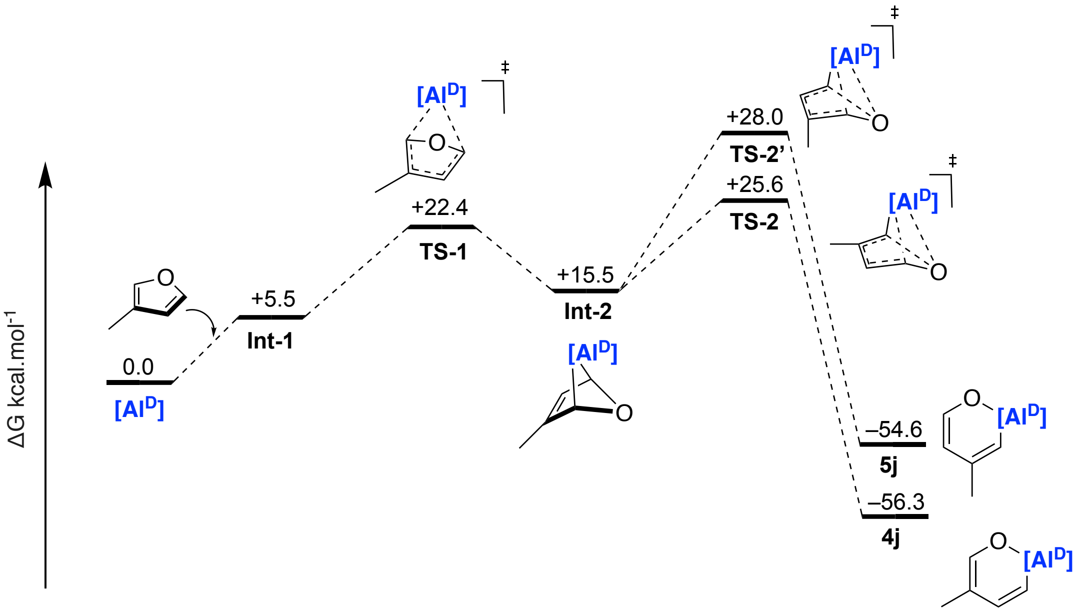

Figure S6.7: Calculated pathway for C–O alumination of 3-methylfuran from **1**. [Al^{D}] represents the Dipp-*BDI*Al fragment.

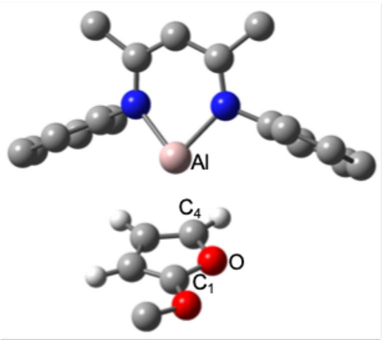

Int–1
C_1–O: 1.35
C_4–O: 1.38
Al–O: 3.59
Al–C_1: 3.61
Al–C_4: 3.66

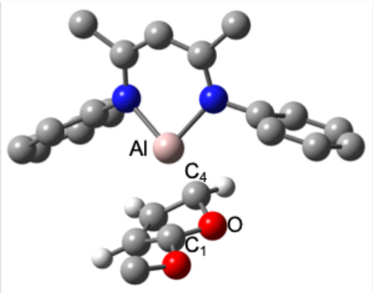

TS–1
C_1–O: 1.37
C_4–O: 1.45
Al–O: 2.61
Al–C_1: 2.58
Al–C_4: 2.23

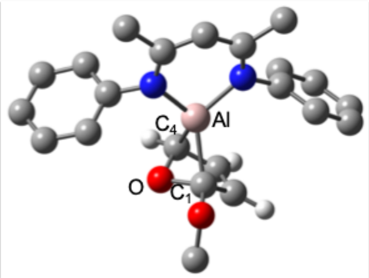

Int-2
C_1–O: 1.50
C_4–O: 1.48
Al–O: 2.28
Al–C_1: 2.04
Al–C_4: 2.04

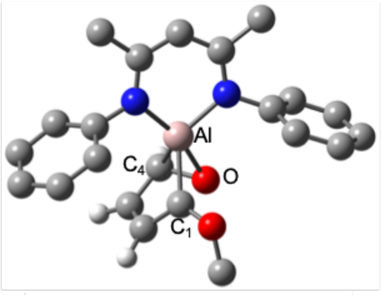

TS–2
C_1–O: 1.89
C_4–O: 1.45
Al–O: 1.92
Al–C_1: 2.02
Al–C_4: 2.01

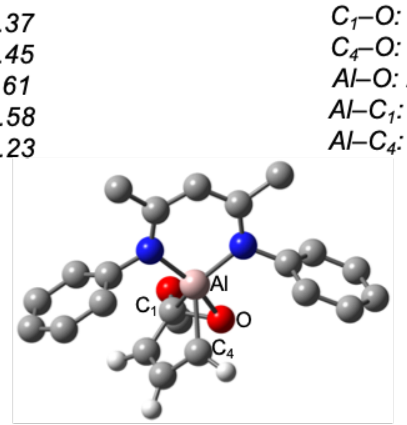

TS–2'
C_1–O: 1.50
C_4–O: 1.97
Al–O: 1.87
Al–C_1: 2.05
Al–C_4: 2.00

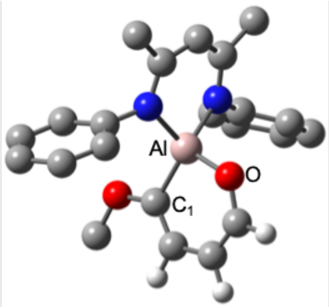

4d
Al–C_1: 1.95
Al–O: 1.77

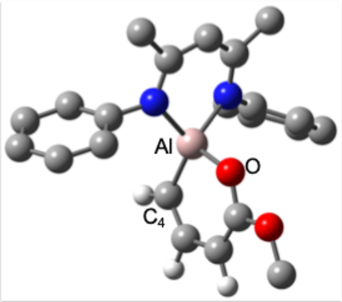

5d
Al–C_4: 1.91
Al–O: 1.80

Figure S6.8: Selected bond lengths (in Å). *i*-Pr groups and some hydrogens have been omitted for clarity.

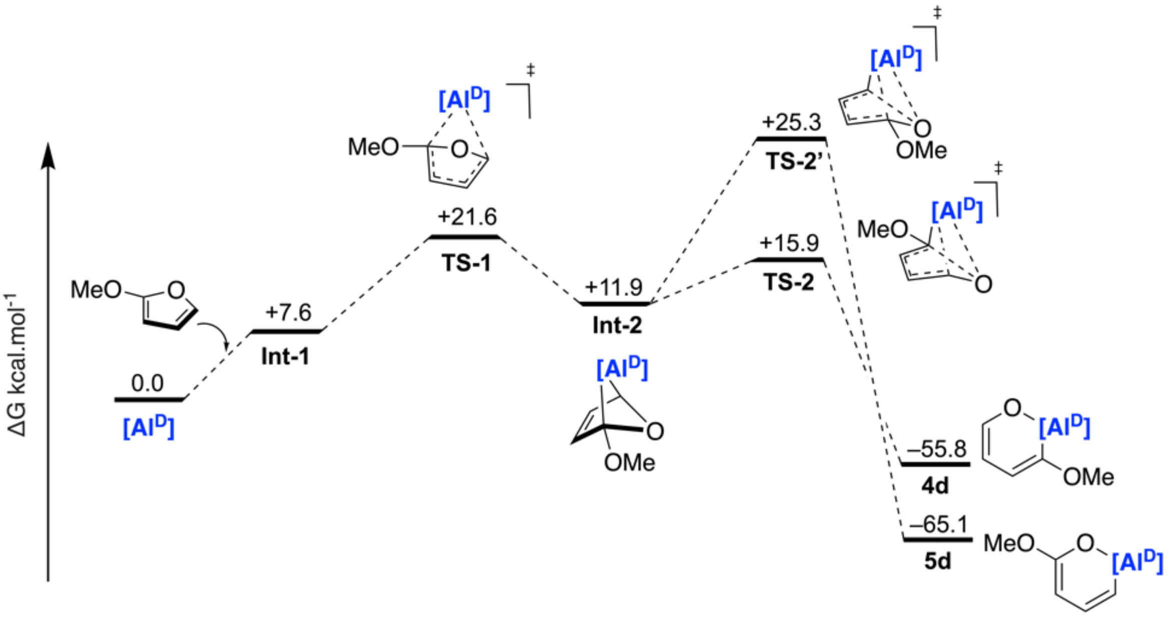

Figure S6.9: Calculated pathway for C–O alumination of 2-methoxyfuran from **1**. [Al^{D}] represents the Dipp-*BDI*Al fragment.

6.3 Nucleus-Independent Chemical Shift calculation (NICS)

A NICS(0) calculation was carried out on the optimised geometries of **4a**, **4b**, **4c**, **4d**, **5b**, **5c** and **5d** inspecting the 6 membered ring system containing the C–Al–O bond. In all cases a small NICS value was observed indicating that there is essentially no degree of aromaticity present within the ring and that the corresponding furans have been dearomatized.

	Isotropic shielding tensor (ppm)
4a	0.16
4b	0.50
4c	0.41
4d	1.53
5b	0.04
5c	0.75
5d	1.37

Table 6.1. Isotropic shielding tensor values for 'dummy atom' in NICS(0) calculation.

6.4 NBO Analysis

The NPA charges and Wiberg Bond Indices were inspected (M06L) for pathways both for furan and 2-methylfuran.

4a

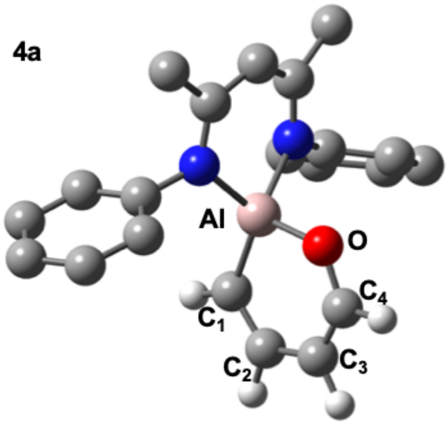

	Furan	Int-1	TS-1	Int-2	TS-2	4a
Al–C₁	-	0.02	0.35	0.45	0.43	0.56
Al–C₄	-	0.01	0.38	0.47	0.36	-
Al–O	-	-	-	-	0.20	0.33
C₁–O	1.05	1.04	0.89	0.86	0.53	-
C₄–O	1.05	1.05	0.96	0.87	0.91	1.13
C₁–C₂	1.65	1.63	1.26	1.09	1.27	1.78
C₂–C₃	1.26	1.27	1.56	1.79	1.59	1.19
C₃–C₄	1.65	1.64	1.32	1.09	1.24	1.65

Table 6.2. Wiberg Bond Indices on stationary points on pathway from **1** and furan

	Al	O	C_1	C_4
Furan	-	-0.46	0.09	0.09
1	0.78	-	-	-
Int-1	0.80	-0.45	0.08	0.12
TS-1	1.29	-0.53	-0.08	-0.08
Int-2	1.87	-0.61	-0.48	-0.48
TS-2	1.96	-0.75	-0.55	-0.41
4a	2.05	-0.90	-0.96	0.21

Table 6.3. *NPA* charges on stationary points on pathway from **1** and furan

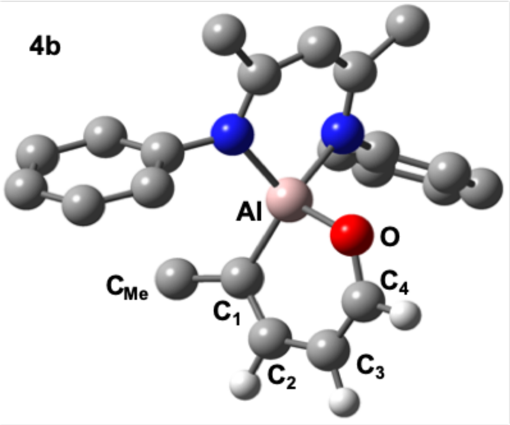

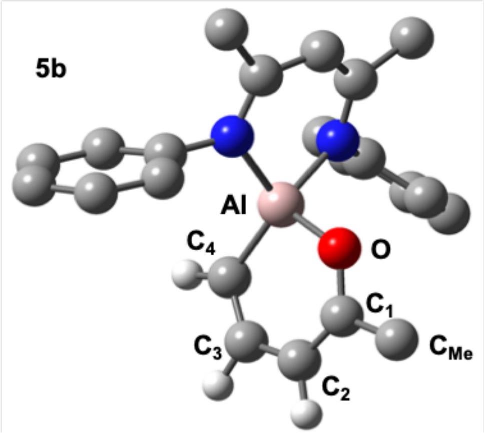

	2-methylfuran	Int-1	TS-1	Int-2	TS-2	4b	TS-2'	5b
Al–C₁	-	0.01	0.39	0.43	0.40	0.51	0.32	-
Al–C₄	-	0.01	0.37	0.43	0.36	-	0.42	0.57
Al–O	-	-	-	-	0.19	0.31	0.19	0.32
C₁–O	1.03	1.03	0.96	0.86	0.50	-	0.89	1.11
C₄–O	1.03	1.03	0.89	0.85	0.91	1.15	0.54	-
C₁–C₂	1.61	1.60	1.28	1.06	1.22	1.74	1.21	1.59
C₂–C₃	1.24	1.25	1.55	1.78	1.61	1.18	1.60	1.19
C₃–C₄	1.66	1.65	1.22	1.09	1.22	1.66	1.27	1.77
C₁–C_Me	1.06	1.05	1.05	1.03	1.07	1.05	1.03	1.03

Table 6.4. Wiberg Bond Indices on stationary points on pathway from **1** and 2-methylfuran.

	Al	O	C_1	C_4	C_{Me}
2-methylfuran	-	-0.47	0.09	0.32	-0.75
1	0.78	-	-	-	-
Int-1	0.80	0.46	0.34	0.09	-0.74
TS-1	1.35	-0.52	-0.35	0.14	-0.72
Int-2	1.90	-0.60	-0.27	-0.49	-0.70
TS-2	1.97	-0.75	-0.31	-0.43	-0.72
4b	2.09	-0.91	-0.71	0.20	-0.69
TS-2'	1.99	-0.75	-0.19	-0.56	-0.70
5b	2.05	-0.91	0.42	-0.97	-0.73

Table 6.5. *NPA* charges on stationary points on pathway from **1** and 2-methylfuran to **4b** and **5b.**

Functional		TS-1	TS-2
**wB97X (wB97XD)**	$\Delta G^{\ddagger}$	31.6	34.5
	$\Delta H^{\ddagger}$	17.4	18.6
**M06L (GD3)**	$\Delta G^{\ddagger}$	**24.1**	**27.0**
	$\Delta H^{\ddagger}$	**8.6**	**11.5**
**M062X (GD3)**	$\Delta G^{\ddagger}$	29.7	33.3
	$\Delta H^{\ddagger}$	14.1	16.8
**B3PW91 (GD3BJ)**	$\Delta G^{\ddagger}$	28.0	31.9
	$\Delta H^{\ddagger}$	14.5	16.6
**Experimental values**	$\Delta G^{\ddagger}_{298K}$		**+25.3 (± 0.5)**
	$\Delta H^{\ddagger}$		**+19.7 (± 2.7)**

Table 6.6. Relative free energies and enthalpies (kcal.mol^{-1}) of TS-1 and TS-2 using specified density functionals for furan pathway. Dispersion single point corrections used. Solvent correction used for entry-2.

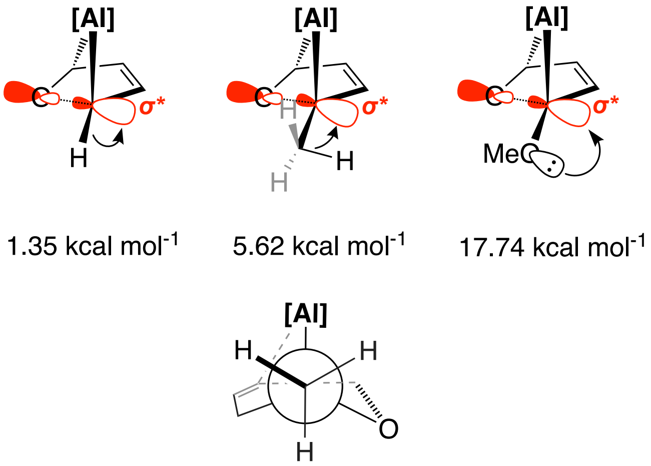

Figure 6.10: Diagram of **Int-1** for furan (left), 2-methylfuran (middle) and 2-methoxyfuran (right). Highlighting the hyperconjugation or anomeric effect that weakens the C–O σ* bond and contributes to the observed selectivity. Values derived from 2nd order perturbations from NBO analysis.

6.4 Alternative Mechanisms

Two alternative pathways for C–O bond activation were investigated computationally. These calculations did not lead to the identification of viable pathways for C–O alumination and either led to high energy intermediates or did not converge to suitable stationary points. These included:

1. (2+1) addition of furan to **1**, and subsequent isomerisation to C–O aluminated products.

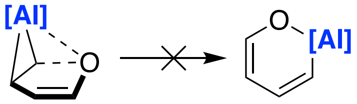

2. Direct oxidative addition pathway, analogous to a proposed literature mechanism.[12] We were unable to find a transition state that supports a mechanism for the direct C–O bond cleavage of furan by oxidative addition to **1** i.e. precluding the (4+1) or (2+1) addition steps. We were able to find a transition state that supports the direct oxidative addition of tetrahydrofuran to **1**. Using this as an input geometry (with modification of the tetrahydrofuran unit to a furan unit) in a transition state calculation, resulted in finding **TS-2**, further supporting the (4+1) pathway.

[12] Kim, Y.; Cho, H.; Hwang, S. Density Functional Theoretical Study on the C□F and C□O Oxidative Addition Reaction at an Al Center. *Bull. Korean Chem. Soc.* **2017**, *38*, 282–284.

6.5 Catalysed C–H and C–O alumination of Me-Furan: Mechanism

6.5.1 Key geometrical parameters of intermediates, Int-3 – Int-10.

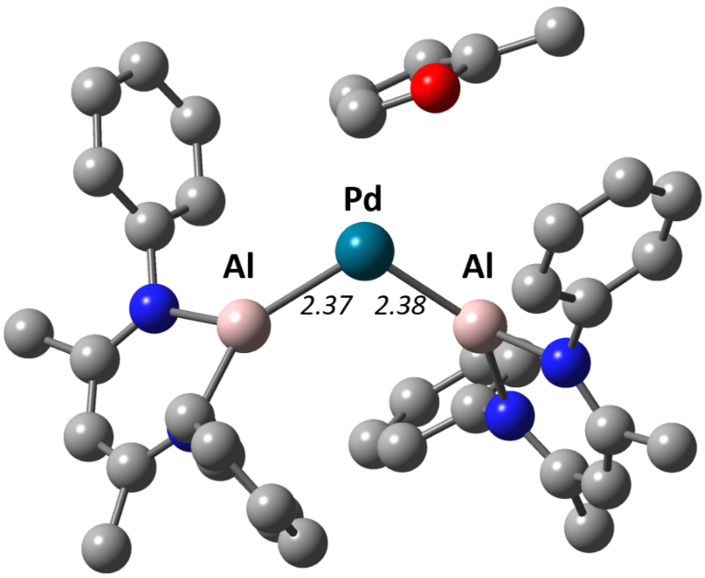

Figure S6.11: Selected bond lengths (in Å) for **Int-3**. *i*-Pr groups and some hydrogens have been omitted for clarity.

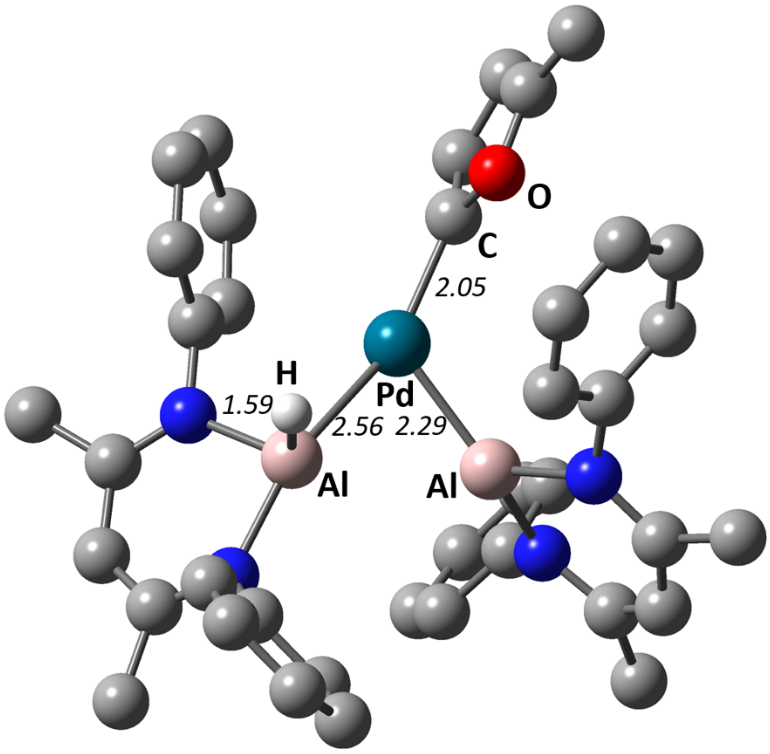

Figure S6.12: Selected bond lengths (in Å) for **Int-4**. *i*-Pr groups and some hydrogens have been omitted for clarity.

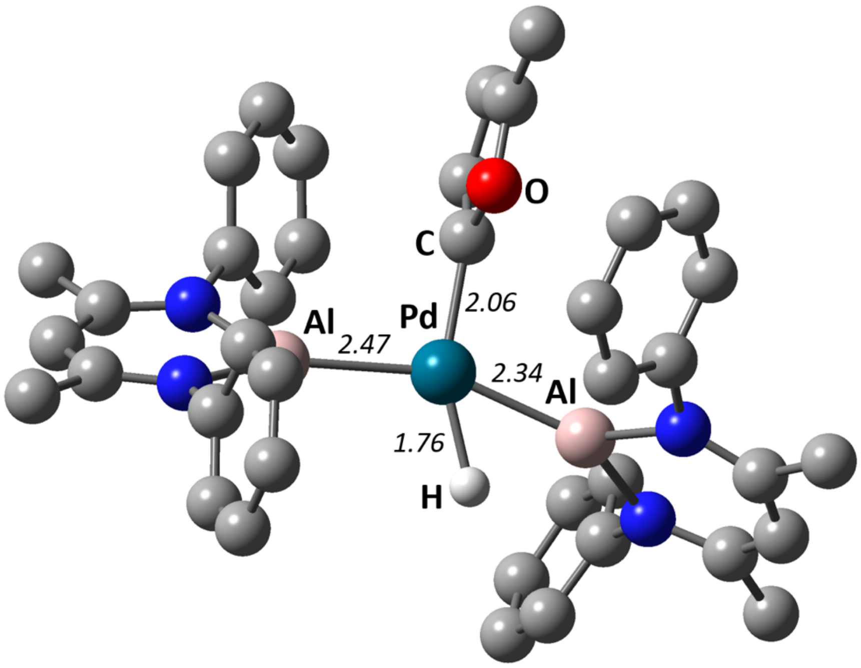

Figure S6.13: Selected bond lengths (in Å) for **Int-5**. *i*-Pr groups and some hydrogens have been omitted for clarity.

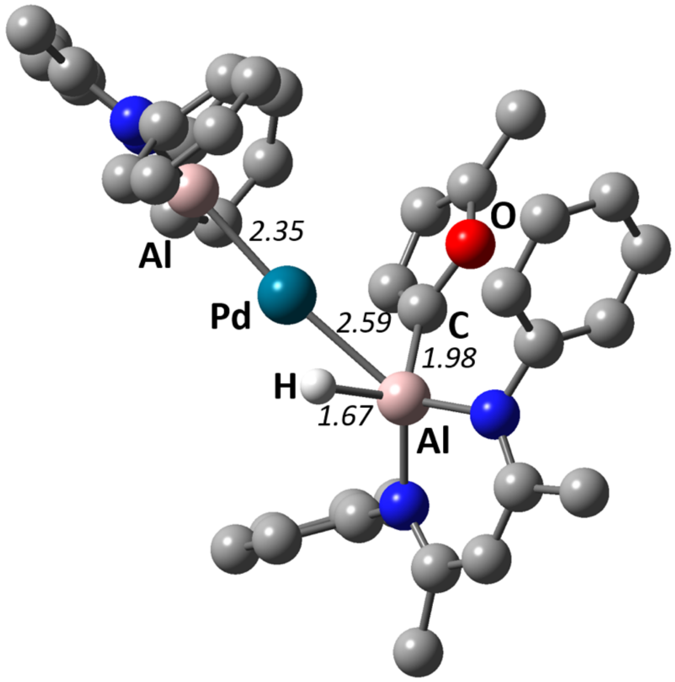

Figure S6.14: Selected bond lengths (in Å) for **Int-6**. *i*-Pr groups and some hydrogens have been omitted for clarity.

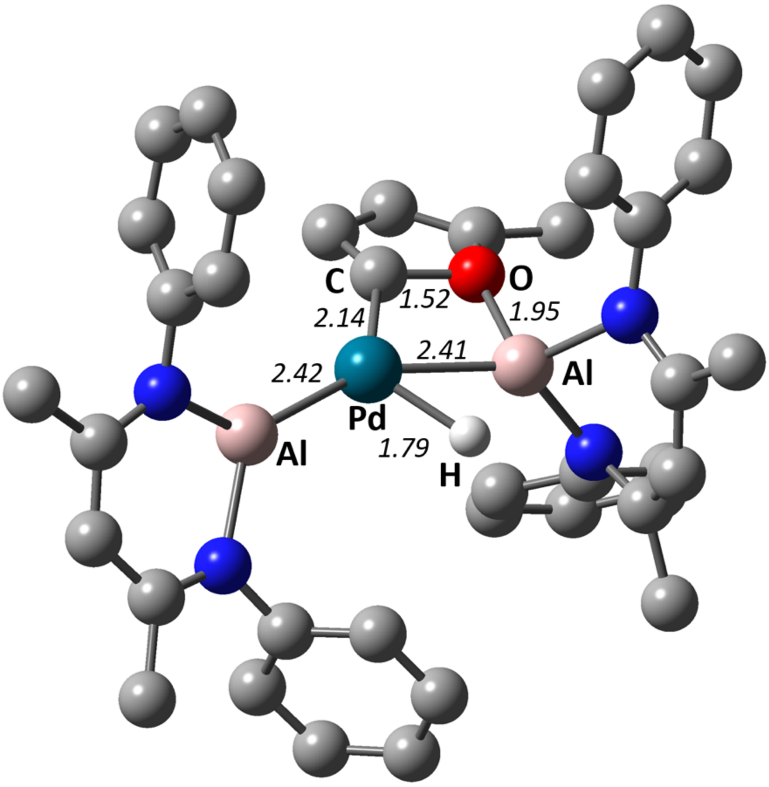

Figure S6.15: Selected bond lengths (in Å) for **Int-7**. *i*-Pr groups and some hydrogens have been omitted for clarity.

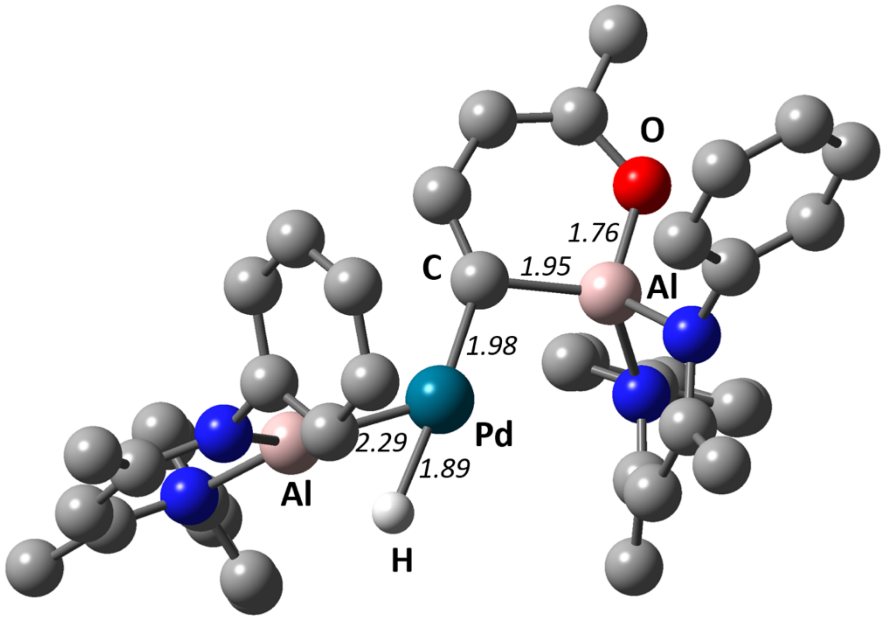

Figure S6.16: Selected bond lengths (in Å) for **Int-8**. *i*-Pr groups and some hydrogens have been omitted for clarity.

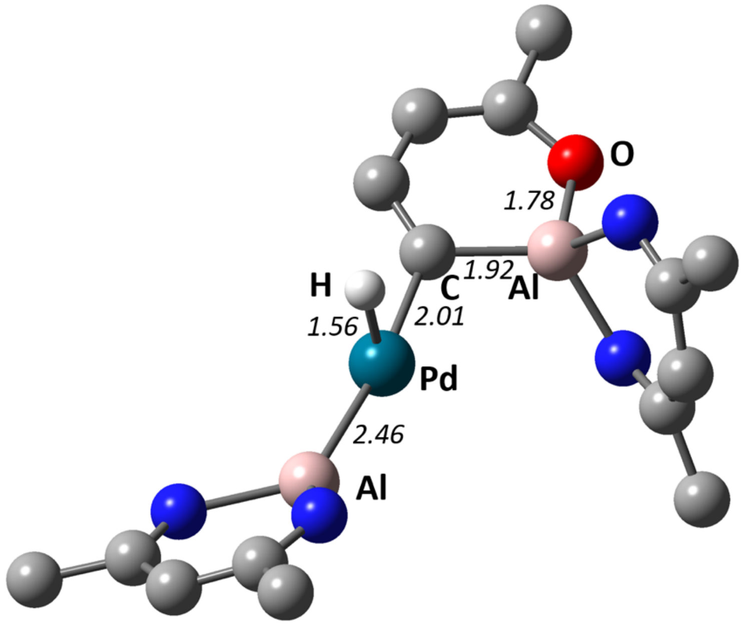

Figure S6.17: Selected bond lengths (in Å) for **Int-9**. 2,6-diisopropylphenyl groups and some hydrogens have been omitted for clarity.

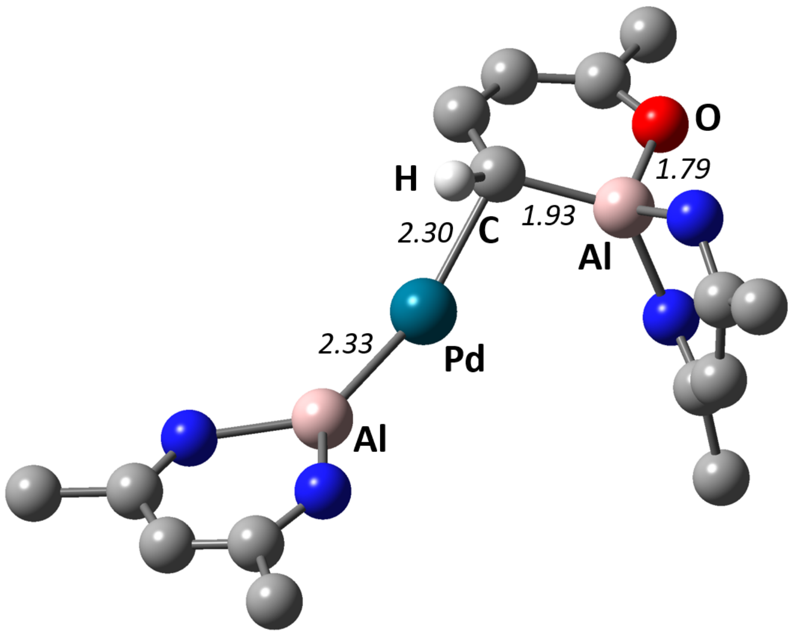

Figure S6.18: Selected bond lengths (in Å) for **Int-10**. 2,6-diisopropylphenyl groups and some hydrogens have been omitted for clarity.

6.5.2 Key geometrical parameters of transition states, TS-3 – TS-6

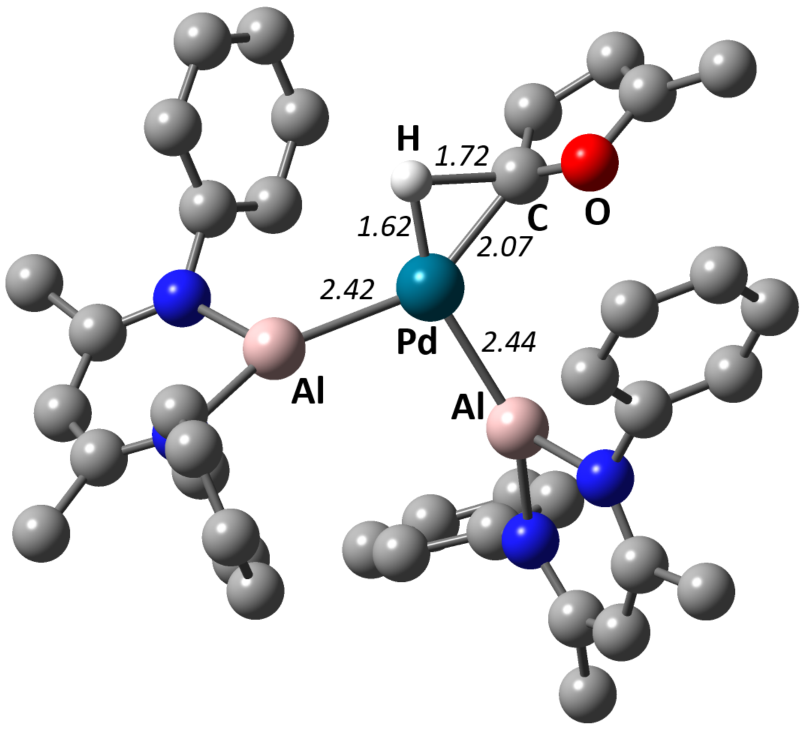

Figure S6.19: Selected bond lengths (in Å) for **TS-3**. *i*-Pr groups and some hydrogens have been omitted for clarity.

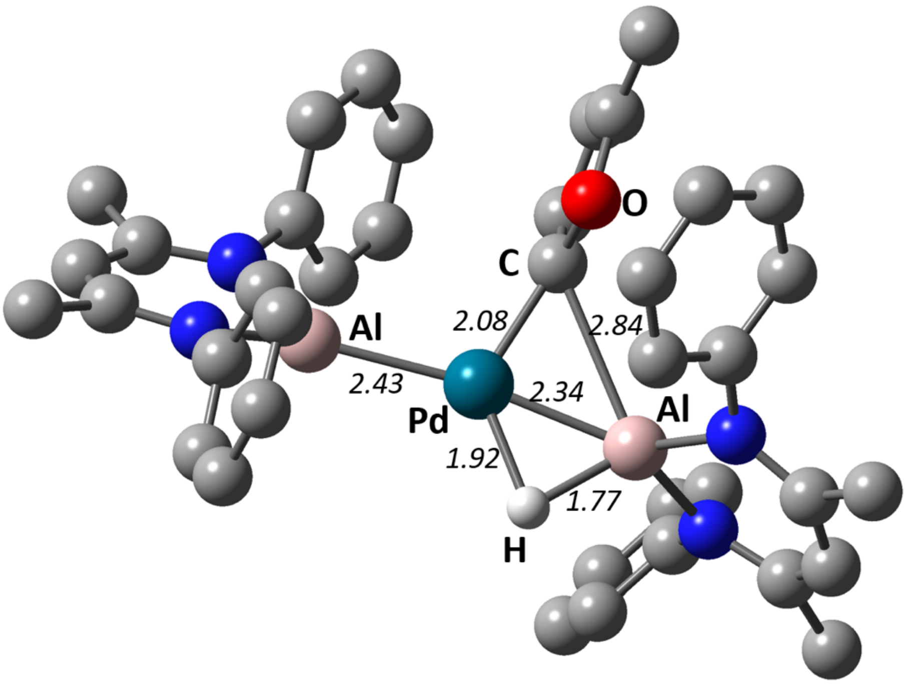

Figure S6.20: Selected bond lengths (in Å) for **TS-4**. *i*-Pr groups and some hydrogens have been omitted for clarity.

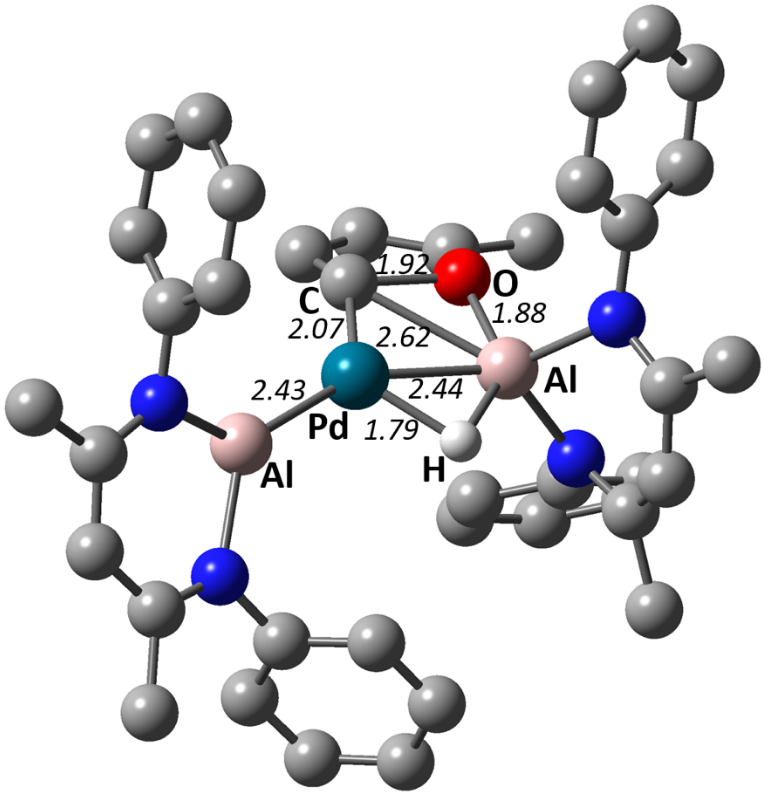

Figure S6.21: Selected bond lengths (in Å) for **TS-5**. *i*-Pr groups and some hydrogens have been omitted for clarity.

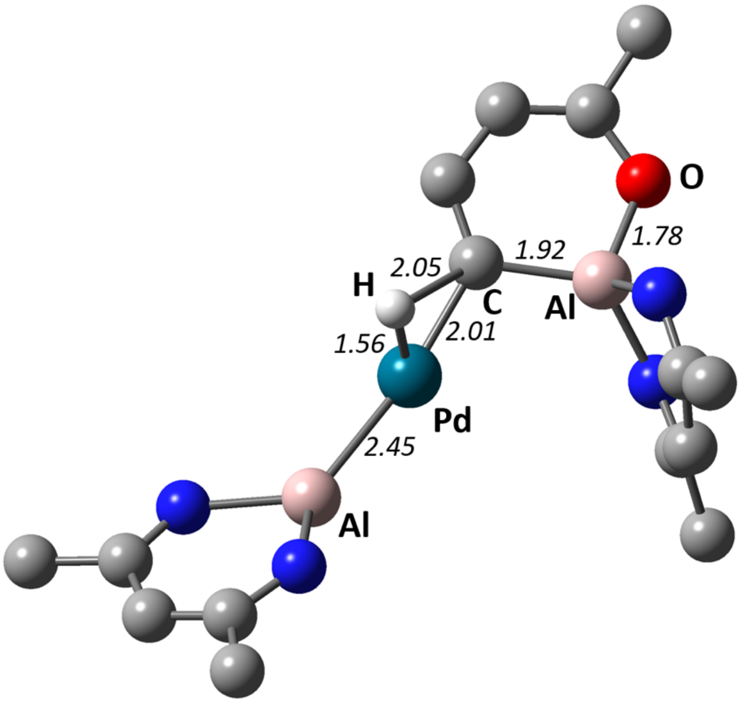

Figure S6.22: Selected bond lengths (in Å) for **TS-6**. 2,6-diisopropylphenyl groups and some hydrogens have been omitted for clarity.

6.5.3. Calculated KIE

The oxidative addition of 2-methylfuran and deuterated 2-methylfuran to [Pd(**1**)$_2$] was considered as a means to obtain a computational estimate of the KIE. The difference in $\Delta G^{\neq}$ allowed to derive an estimated KIE = 5.4. This value is virtually close to the experimental one (KIE = 4.8 ± 0.3) and consistent with a strong primary KIE, which gives additional support to the choice of M06L as the best functional for this system.

6.5.4 C–H and C–O alumination of Me-Furan from [Pd(1)$_2$]: General Pathway

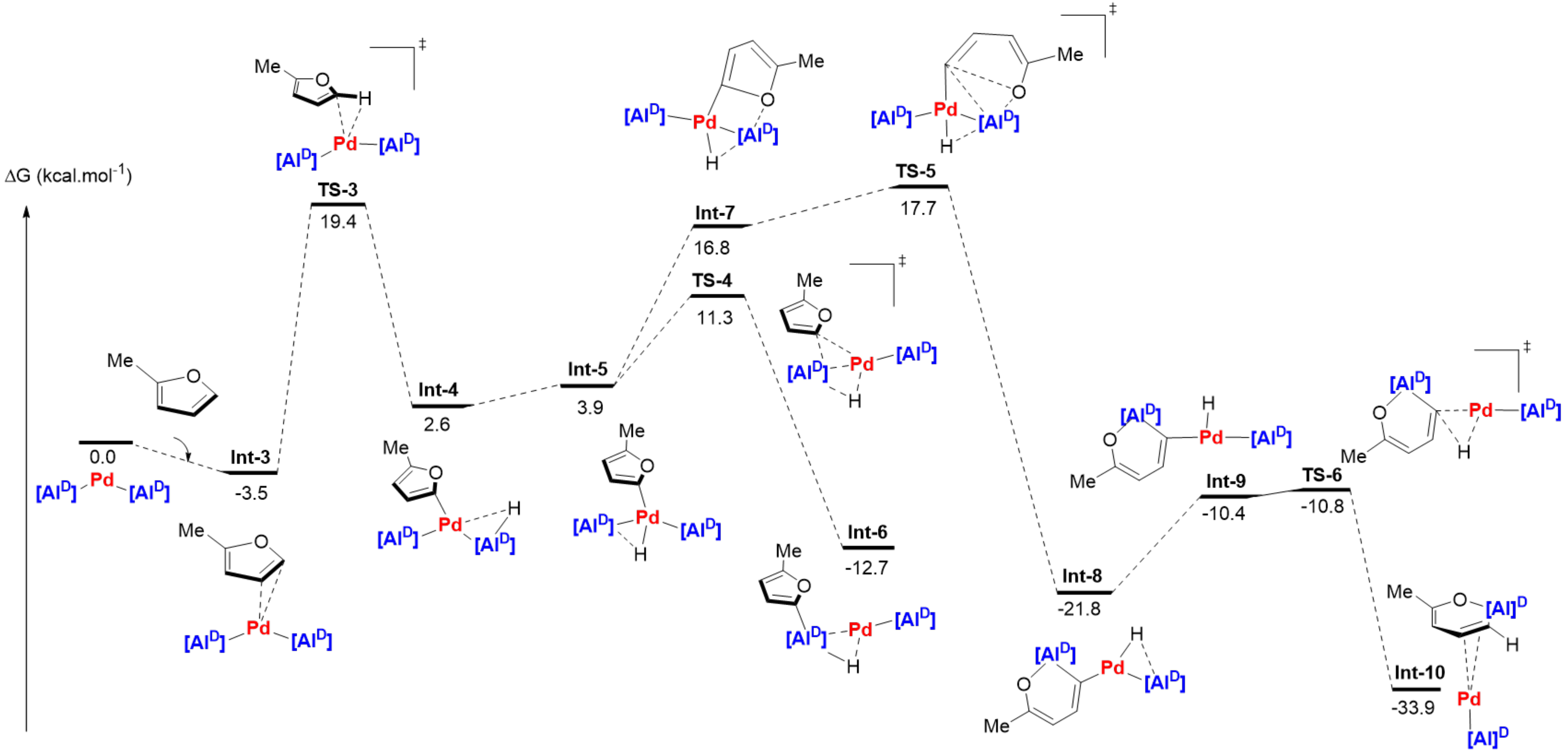

Figure S6.23: DFT calculated pathway for the palladium-catalyzed C–H and C-O alumination reactions of [Pd(**1**)$_2$] with 2-methyl-Furan.

Another possible route in the current pathway is the migration of the furan ligand through **TS-4b** ($\Delta G^{\ddagger}$ = +9.7 kcal mol^{-1}) from **Int-4** to form **Int-6b** (Figure S6.24). Both pathways going through either **TS-4** (from **Int-5**) or **TS-4b** (from **Int-4**) are feasible processes as the corresponding local Gibbs activation barriers are close in energy (8.7 kcal mol^{-1} *vs* 7.1 kcal mol^{-1}, respectively). However **TS-4b** is non-productive as it leads to migration of the hydride and furyl ligands to separate aluminium ligands and hence does not lead to the formation of **7b** bound to Pd.

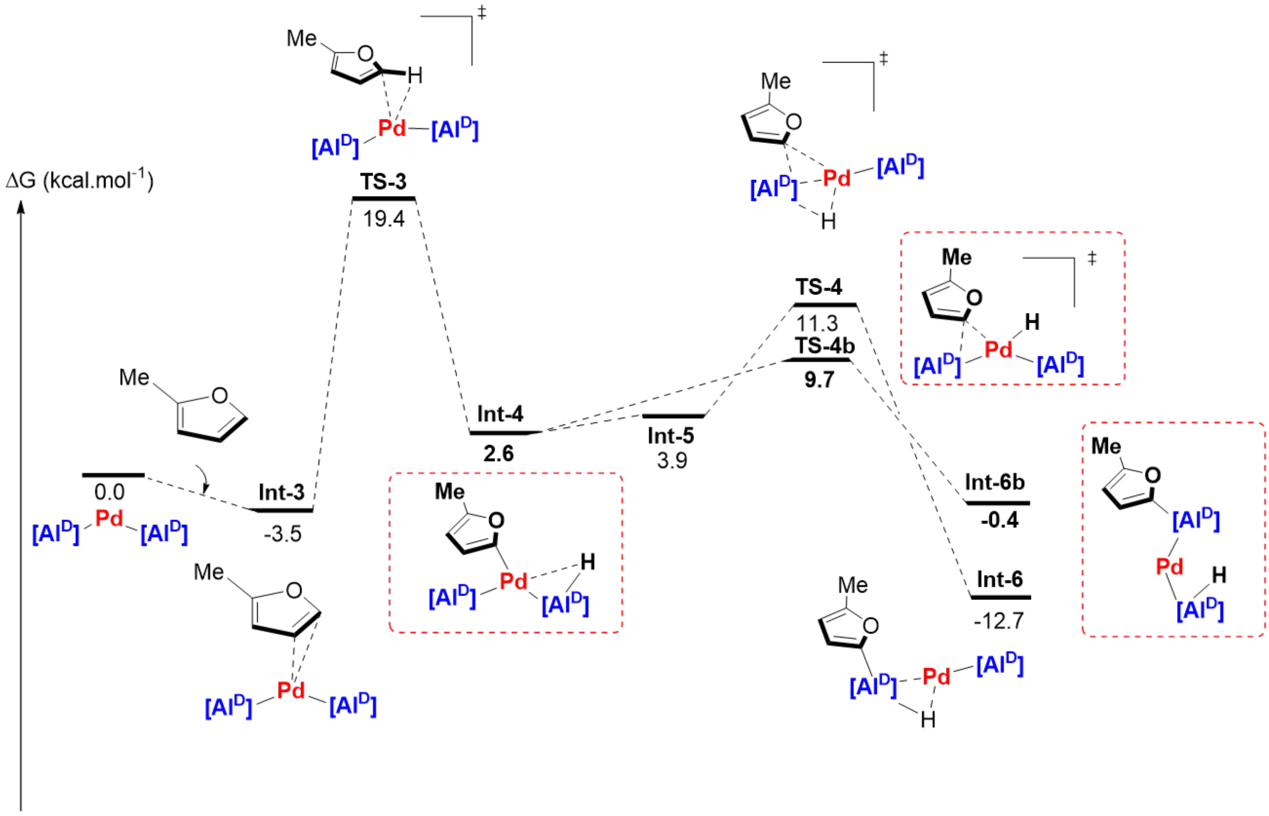

Figure S6.24: DFT calculated pathways for the palladium-catalyzed C–H alumination reaction of [Pd(**1**)$_2$] with 2-methyl-Furan.

When dispersion and solvent corrections were included into the calculations with M06L for the C–H activation of Me-Furan (**TS-3**) and the oxidative addition of the C–O bond to the Al centre (**TS-5**), no impact on the energies was observed (table S6.7). The performance of the three functionals was then inspected for the local barriers energies for both **TS-3** and **TS-5**. M06L appeared to be more robust.

Functional	Δ(Int-3 – TS-3)	Δ (Int-4 – TS-5)
**M06L**	22.9	15.1
**M06L (GD3)**	22.9	16.5
**M06L (pcm)**	21.5	15.3
**ωB97X**	21.4	10.1
**ωB97XD**	18.5	13.7
**B3PW91**	40.7	11.2
**B3PW91 (GD3)**	17.8	9.0

Table 6.7: Comparison of the calculated free-energy local barriers for **TS-3** and **TS-5** using M06L (including or not dispersion (GD3) and solvent (pcm) corrections), with ωB97X and B3PW91 (including or not dispersion corrections). All energies provided in kcal mol^{-1}.

6.5.5 C–H and C–O alumination of Me-Furan from [Pd(1)$_2$(PCy$_3$)]

Depending on the PdL_2 fragment that is formed under catalytic conditions, two pathways can be considered. The pathway from [Pd(**1**)$_2$] is provided in the main text, while the pathway from [Pd(**1**)$_2$(PCy$_3$)] is given below (figure S6.25).

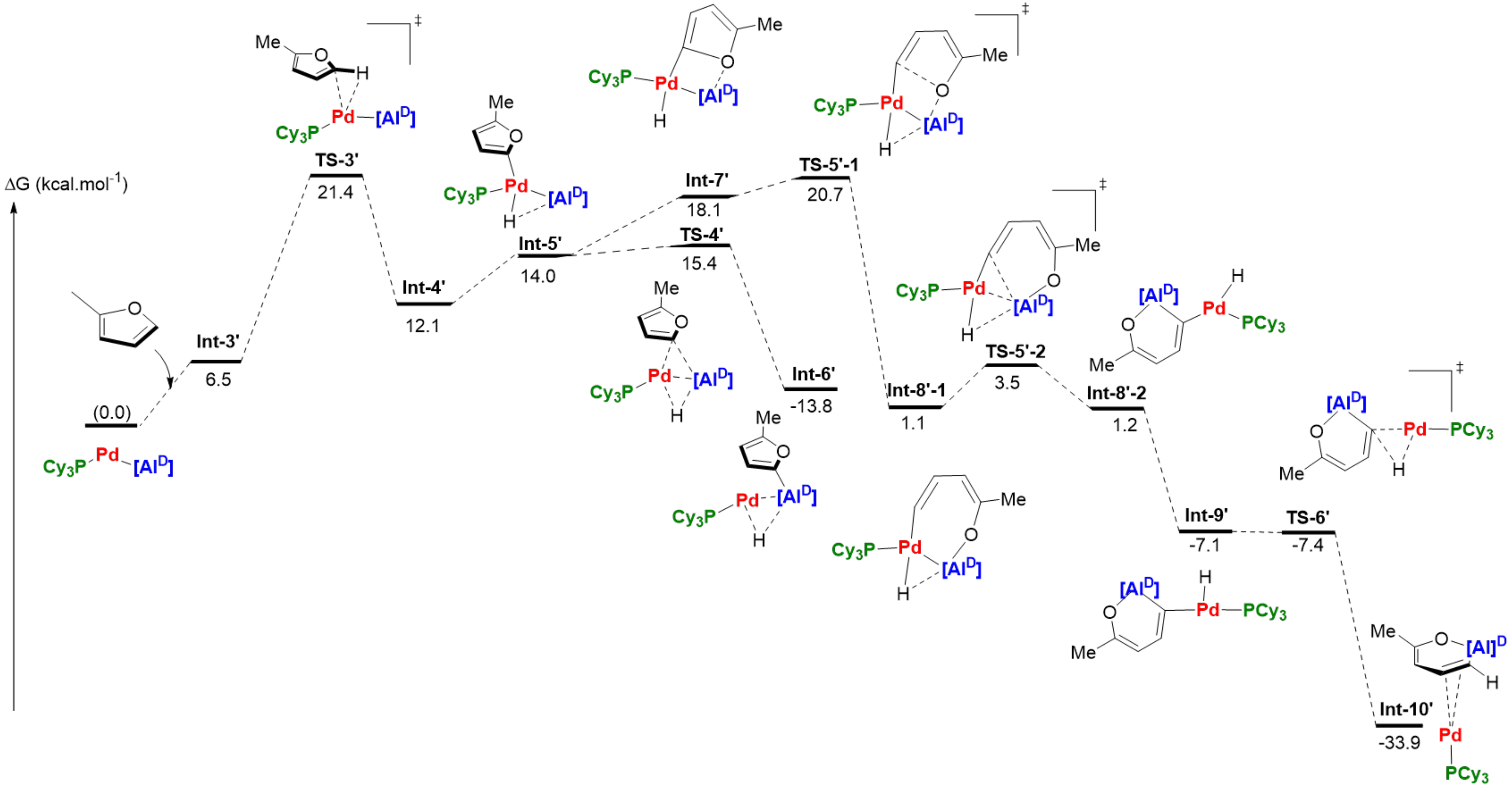

Figure S6.25: DFT calculated pathway for the palladium-catalyzed C–H and C-O alumination reactions of [Pd(1)PCy_3] with 2-methyl-Furan.

	**[Pd(1)$_2$]**	**[Pd(1)(PCy$_3$)]**
**INT-3 & INT-3'**	-3.5	6.5
**TS-3 & TS-3'**	**19.4**	**21.4**
**INT-4 & INT-4'**	2.6	12.1
**INT-5 & INT-5'**	3.9	14.0
**TS-4 & TS-4'**	**11.3**	**15.4**
**INT-6 & INT-6'**	-12.7	-13.8
**INT-7 & INT-7'**	16.8	18.1
**TS-5 & TS-5'a-TS-5'b**	**17.7**	**20.7** **3.5**
**INT-8 & INT-8'-2**	-21.8	1.2
**INT-9 & INT-9'**	-10.4	-7.1
**TS-6 & TS-6'**	**-10.8**	**-7.4**
**INT-10 & INT-10'**	-33.9	-33.9
**Δ(TS-3 – Int-3) & Δ(TS-3' – [Pd(1)(PCy$_3$)])**	**22.9**	**21.4**
**Δ(TS-4 – Int-4) & Δ(TS-4' – Int-4')**	**8.7**	**3.3**
**Δ(TS-5 – Int-4) & Δ(TS-5' – Int-4')**	**15.1**	**8.6**
**Δ(TS-6 – Int-8)**	**11.0**	**-**

Table 6.8: Comparison of the calculated free energy profile for C–H and C–O alumination reactions of [Pd(**1**)$_2$] and [Pd(**1**)$_2$(PCy$_3$)] with 2-methyl-Furan.

Both pathways, initiated from either [Pd(**1**)$_2$] or [Pd(**1**)$_2$(PCy$_3$)], have very similar trends. They both involve a rate-limiting oxidative addition of the C–H bond to palladium. When comparing both pathways, a difference in the mechanism can be observed in the addition of the C–O bond to the Al centre step (**TS-5**). The [Pd(**1**)$_2$] system involves a one step process with breaking of the C–O bond and insertion of the Al centre simultaneously (**TS-5**), while [Pd(**1**)$_2$(PCy$_3$)] involves a two steps process with breaking of the C–O bond (**TS-5'-1**) leading to a 7-membered ring intermediate (**Int8'-1**) prior to the insertion of the Al centre into the C–O bond (**TS-5'-2**).

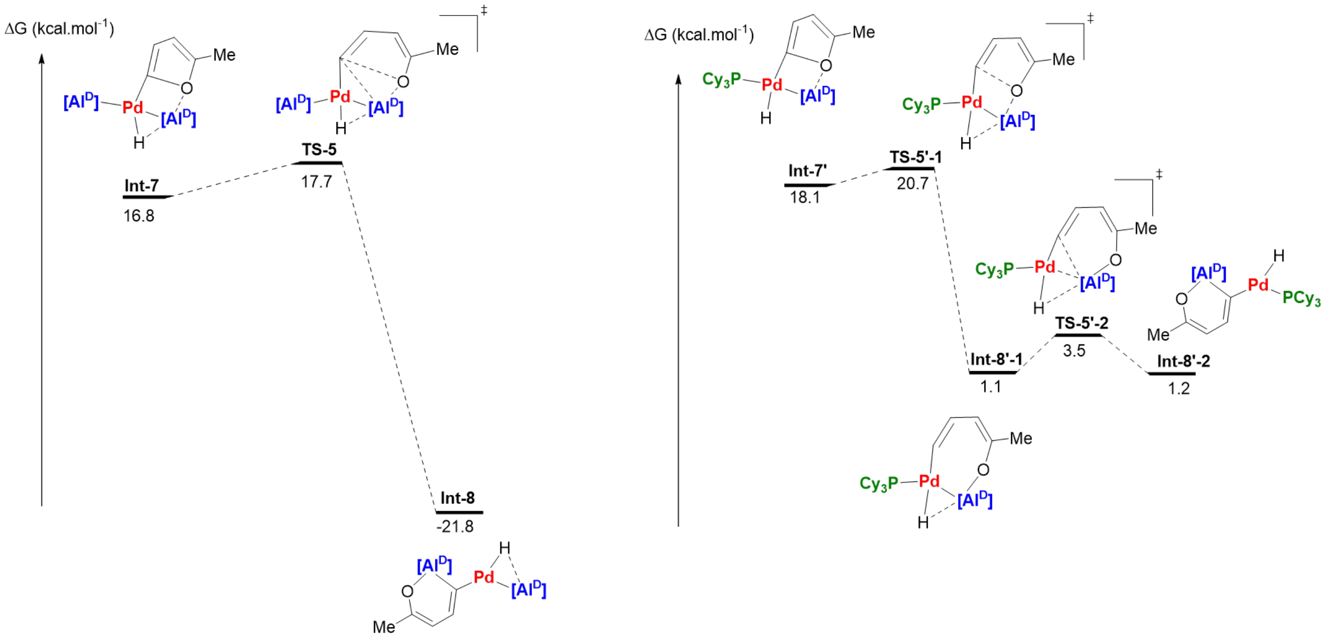

Figure S6.26: Comparison of the addition of the C–O bond to the Al centre process for both [Pd(**1**)$_2$] and [Pd(**1**)$_2$(PCy$_3$)] pathways.

6.5.6 Alternative Mechanisms

A number of alternative pathways for C–H and C–O bond activation were investigated computationally. These calculations did not lead to the identification of viable pathways for C–H and C–O alumination and either led to high energy intermediates or did not converge to suitable stationary points. These included:

(i) The ligand assisted oxidative addition of the C–O of 2-methylfuran to [Pd(**1**)$_2$] which proceeds by a high energy TS. Activation barrier = **32.5 kcal mol^{-1}**

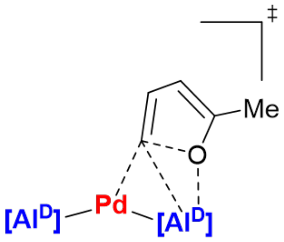

Figure S6.27: Transition state **TS-7** of the ligand assisted oxidative addition step of the C–O of 2-methylfuran to [Pd(**1**)$_2$].

(ii) The ligand assisted oxidative addition of the C–H bond of 2-methylfuran to [Pd(**1**)$_2$] or [Pd(**1**)(PCy$_3$)] for both of which a suitable TS could not be located.

6.6 NBO analysis

Wiberg Bond Indices (WBI) and NPA charges were inspected (M06L) for pathway from [Pd(**1**)$_2$].

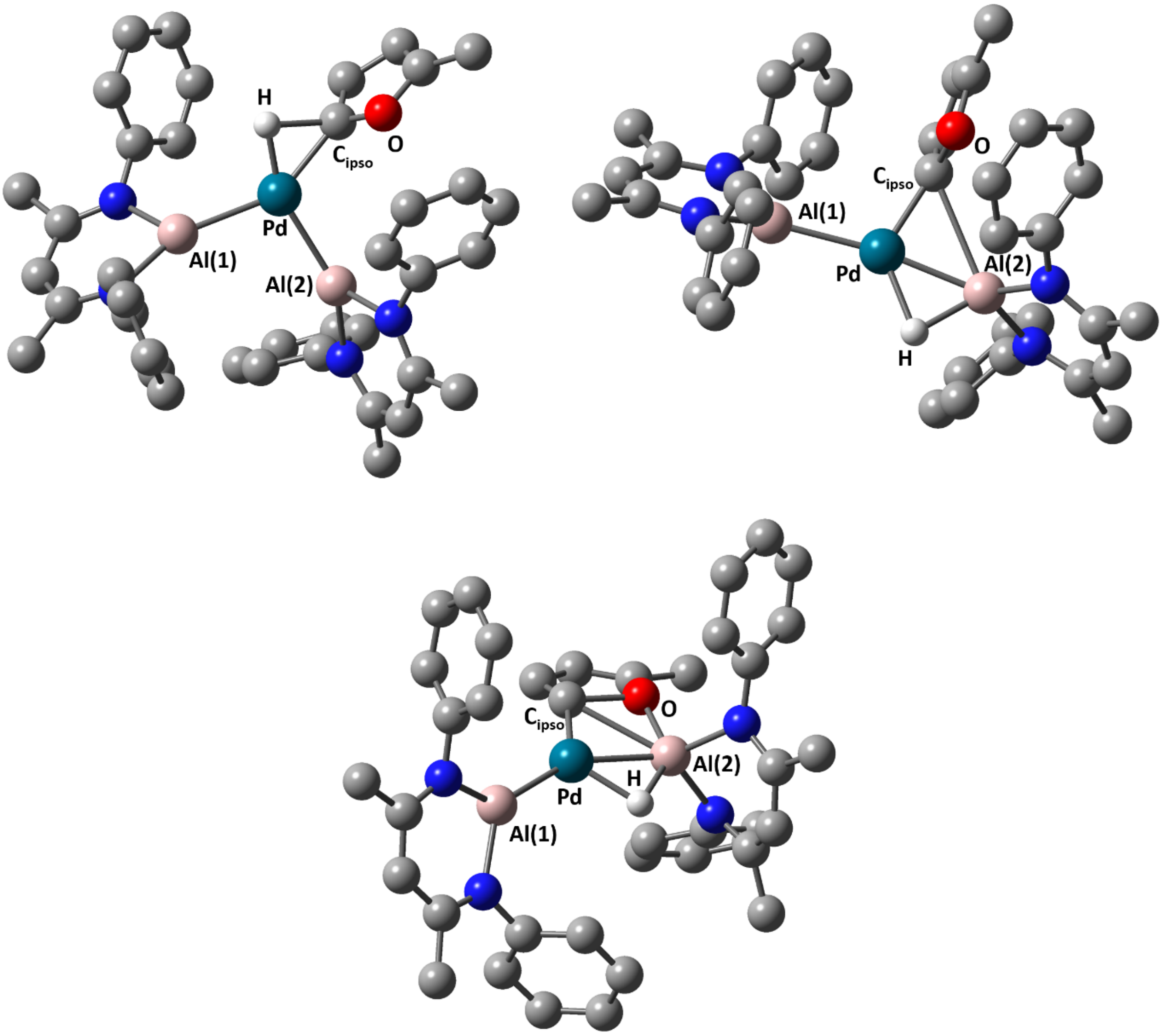

Figure S6.28: Models for **TS-3**, **TS-4** and **TS-5**, showcasing the atoms and bonds relevant to the NBO analysis.

	Int-3	TS-3	Int-4	Int-5	TS-4	Int-6	Int-7	TS-5	Int-8
Pd-Al(1)	0.86	1.10	0.38	0.82	0.88	0.90	0.96	0.95	0.68
Pd-Al(2)	0.85	0.84	0.68	0.66	0.51	0.17	0.39	0.36	0.07
Pd-H	0.01	0.42	0.26	0.40	0.27	0.13	0.34	0.35	0.22
Pd-Cipso	0.12	0.43	0.49	0.54	0.48	0.13	0.47	0.57	0.59
Al(1)-H	0.00	0.12	1.30	0.04	0.03	0.03	0.05	0.04	0.48
Al(2)-H	0.00	0.01	0.07	0.34	0.47	0.60	0.44	0.43	0.00
Al(2)-Cipso	0.01	0.09	-0.07	0.16	0.31	0.48	0.18	0.17	0.51
Al(2)-O	0.00	0.01	0.06	0.01	0.01	0.01	0.16	0.19	0.32
Cipso-H	0.89	0.35	0.01	0.10	0.05	0.02	0.03	0.03	0.13
Cipso-O	0.07	0.83	0.19	1.00	0.99	0.98	0.79	0.55	0.04

Table 6.9: Wiberg Bond Indices on stationary points on the pathway from [Pd(**1**)$_2$]

	Pd	H	Cipso	Al(1)	Al(2)	O
Int-3	-0.97	0.25	0.05	1.16	1.19	-0.46
TS-3	-1.15	0.07	0.12	1.40	1.39	-0.48
Int-4	-0.38	-0.47	-0.02	1.29	1.22	-0.48
Int-5	-0.96	-0.26	0.09	1.54	1.43	-0.49
TS-4	-0.83	-0.35	0.03	1.58	1.34	-0.49
Int-6	-0.58	-0.38	-0.36	1.72	1.18	-0.47
Int-7	-0.86	-0.28	-0.03	1.78	1.28	-0.68
TS-5	-0.87	-0.28	-0.04	1.83	1.30	-0.77
Int-8	0.02	-0.34	-0.67	1.63	0.94	-0.86

Table 6.10: NPA charges on stationary points on the pathway from [Pd(**1**)$_2$]

6-7 XYZ Coordinates

Dipp-*BDI*Al(I).log
SCF (M06L) = -1241.24018280
E(SCF)+ZPE(0 K)= -1240.602632
H(298 K)= -1240.566224
G(298 K)= -1240.669746
Lowest Frequency = 11.7804cm-1

Al	9.836661	1.454985	3.307972
N	8.896836	2.853496	4.439726
N	10.339142	2.953944	2.036225
C	8.738248	4.164690	4.223745
C	9.317739	4.830729	3.138075
H	9.151302	5.900694	3.082863
C	10.091303	4.266512	2.117625
C	7.914659	4.978450	5.180403
H	6.912136	4.551789	5.289522
H	7.823403	6.013839	4.849226
H	8.353752	4.971137	6.183959
C	10.649842	5.191858	1.074790
H	10.247866	4.957063	0.083151
H	11.735351	5.074608	0.992660
H	10.423417	6.234424	1.302449
C	8.292146	2.282863	5.608226
C	7.050699	1.630151	5.497468
C	6.526001	1.006949	6.633606
H	5.568337	0.494873	6.559347
C	7.203061	1.029398	7.844024
H	6.780770	0.537179	8.715680
C	8.425325	1.685998	7.938335
H	8.952477	1.701776	8.889101
C	8.991360	2.321803	6.832815
C	6.266766	1.585899	4.199398
H	6.815795	2.169697	3.448402
C	6.146168	0.155401	3.676382
H	7.132945	-0.292116	3.515017
H	5.600264	0.130894	2.727143
H	5.604894	-0.479331	4.387268
C	4.885750	2.220199	4.359034
H	4.947887	3.252590	4.718089
H	4.272240	1.662153	5.075151
H	4.348611	2.227584	3.405277
C	10.346890	2.996131	6.937863
H	10.404853	3.759703	6.151324
C	10.565241	3.700454	8.273073
H	9.749052	4.390153	8.511262
H	11.497312	4.273225	8.254004
H	10.647487	2.988752	9.101455
C	11.467518	1.990452	6.671380
H	11.347632	1.499096	5.696530
H	11.467666	1.200895	7.431137
H	12.448603	2.477822	6.687993
C	11.119350	2.480014	0.930176
C	12.495834	2.245654	1.104462
C	13.216022	1.702938	0.036164
H	14.280785	1.513551	0.159861
C	12.598417	1.401850	-1.168893
H	13.174002	0.977799	-1.986992
C	11.238356	1.645328	-1.326380
H	10.757819	1.407633	-2.272290
C	10.476078	2.185452	-0.290040
C	13.218820	2.565426	2.399293
H	12.495626	3.017079	3.091713
C	13.760212	1.297210	3.057168
H	12.956076	0.581620	3.260380
H	14.257704	1.530192	4.004684
H	14.492718	0.800031	2.411109
C	14.340852	3.578698	2.176780
H	13.972978	4.504548	1.722848
H	15.114702	3.175365	1.514047
H	14.824506	3.837712	3.124017
C	8.983950	2.403165	-0.460128
H	8.674328	3.181464	0.249320
C	8.603040	2.882429	-1.857284
H	9.180236	3.761798	-2.160858
H	7.541571	3.145373	-1.893921
H	8.763465	2.106133	-2.612987
C	8.212925	1.132956	-0.099049
H	8.444588	0.794017	0.919468
H	8.476667	0.314188	-0.777825
H	7.131356	1.294139	-0.165889

Furan_opt.log
SCF (M06L) = -229.995615843
E(SCF)+ZPE(0 K)= -229.925665
H(298 K)= -229.920987
G(298 K)= -229.951936
Lowest Frequency = 614.8317cm-1

C	-0.929044	-0.288462	0.008020
C	0.427497	-0.294834	-0.083410
C	0.832673	1.072638	-0.057325
C	-0.309339	1.802883	0.047984
O	-1.396916	0.986499	0.089264
H	1.060861	-1.166255	-0.160049
H	1.839212	1.460601	-0.109816
H	-0.520919	2.858685	0.103248
H	-1.682163	-1.059795	0.028373

Furan_Int-1.log
SCF (M06L) = -1471.24321042
E(SCF)+ZPE(0 K)= -1470.534384
H(298 K)= -1470.492823
G(298 K)= -1470.606980
Lowest Frequency = 17.5595cm-1

H	1.306890	0.074487	-3.355614
C	1.791977	1.050419	-3.277736
H	2.761626	0.992914	-3.786558
H	1.171355	1.764904	-3.829307
C	1.971262	1.502758	-1.826373
H	0.992185	1.410767	-1.331023
C	2.368375	2.976514	-1.804656
H	2.629644	3.324144	-0.800302
H	1.544204	3.595563	-2.175995
C	-1.640668	-1.343707	-1.626346
C	-2.890118	-1.486542	-2.449646
H	-3.659844	-2.053509	-1.915747
H	-2.686681	-1.989987	-3.395892
H	-3.328077	-0.504862	-2.658616
H	-1.481348	1.035779	-2.603549
H	-0.873279	2.690485	-2.682410
H	-2.612027	2.387757	-2.803267
C	-1.819238	2.213315	-0.786414
C	-1.859332	3.686104	-0.401807

H	-1.945231	3.823448	0.680229
H	-2.693722	4.211779	-0.879576
C	-4.159072	2.027991	0.178453
H	-4.255229	3.105676	0.083461
C	-5.210679	1.295347	0.715842
H	-6.121967	1.798893	1.026337
C	-5.081597	-0.076517	0.878452
H	-5.891689	-0.642293	1.333289
C	-3.921542	-0.747225	0.483663
H	-5.873279	-2.774105	1.050298
C	-5.023623	-3.032055	0.409145
H	-4.841126	-4.103412	0.536928
C	-2.880784	0.002080	-0.099541
C	-2.976330	1.406030	-0.225568
H	-0.899991	1.796746	-0.337469
H	-2.945269	-2.617710	0.172462
C	-3.777180	-2.229333	0.771809
C	-3.409088	-2.436320	2.241755
H	-2.487843	-1.901443	2.499249
H	-3.263384	-3.498356	2.466030
H	-4.203693	-2.059806	2.895981
C	0.836978	2.150760	3.567744
C	2.113180	1.808673	3.242145
H	2.817405	1.279415	3.867671
H	3.212552	2.157924	1.310009
H	0.225163	2.008856	4.443978
C	1.148854	2.851759	1.527462
H	0.818660	3.361201	0.634197
Al	-0.160624	-0.409369	0.883603
N	-1.662368	-0.637079	-0.492809
H	-5.335671	-2.864416	-0.627352
H	-0.943414	4.185763	-0.736425
C	-1.691003	2.066463	-2.302897
C	-0.475807	-1.958627	-2.108866
H	-0.587205	-2.586111	-2.986106
C	0.832855	-1.737067	-1.663397
C	1.947015	-2.406298	-2.419035
H	1.566970	-2.981348	-3.264853
H	2.497444	-3.084175	-1.756604
H	2.680303	-1.681894	-2.786356
H	1.871230	-2.799359	0.790094
C	2.885031	-2.557744	1.135404
C	2.810131	-2.280026	2.637097
H	2.125011	-1.452984	2.853002
H	2.460714	-3.163457	3.181802
H	3.797787	-2.012900	3.032001
C	3.776751	-3.766524	0.857253
H	3.865197	-3.977465	-0.213652
H	3.378318	-4.661515	1.345130
H	4.790903	-3.612352	1.242190
C	3.348840	-1.321767	0.391694
C	2.488678	-0.569820	-0.428898
C	2.932071	0.620965	-1.048368
C	2.319347	2.267370	1.909832
C	4.269708	0.990301	-0.895541
H	4.631297	1.895061	-1.377687
C	5.138926	0.240632	-0.109657
H	6.174349	0.549435	0.005020
C	4.670368	-0.890204	0.542923
H	5.341978	-1.462492	1.180113
N	1.122250	-0.954821	-0.614851
H	3.228462	3.174707	-2.454163
O	0.237944	2.802390	2.537531

Furan_TS-1.log
SCF (M06L) = -1471.22028599
E(SCF)+ZPE(0 K)= -1470.512400
H(298 K)= -1470.471959
G(298 K)= -1470.581715
Lowest Frequency = -171.0741cm-1

H	-1.960943	-1.950786	-2.714334
C	-2.169962	-2.787811	-2.043405
H	-3.190111	-3.135911	-2.243580
H	-1.482890	-3.597727	-2.311034
C	-2.005074	-2.406142	-0.570559
H	-1.003101	-1.965773	-0.464348
C	-2.056559	-3.662085	0.293046
H	-2.015177	-3.433169	1.362973
H	-1.213289	-4.318439	0.053927
C	1.226626	0.319206	-2.121994
C	2.437583	0.191600	-3.002245
H	3.100518	1.054578	-2.878113
H	2.156373	0.126669	-4.054107
H	3.031485	-0.687776	-2.736230
H	1.538550	-2.333190	-2.077256
H	1.091545	-3.881380	-1.358805
H	2.798364	-3.493262	-1.616267
C	1.893301	-2.464009	0.078270
C	2.044816	-3.552726	1.132734
H	2.123431	-3.124739	2.136121
H	2.928031	-4.177194	0.956489
C	4.192012	-1.633200	0.751735
H	4.423557	-2.636037	1.099637
C	5.136649	-0.623077	0.899350
H	6.101423	-0.841042	1.349166
C	4.835516	0.665562	0.485393
H	5.566915	1.459925	0.620824
C	3.603325	0.972132	-0.101687
H	5.363544	3.102451	-0.812639
C	4.430500	2.992685	-1.375894
H	4.153830	3.988058	-1.737115
C	2.665852	-0.066312	-0.258043
C	2.943267	-1.375792	0.187957
H	0.925439	-1.978358	0.255884
H	2.388822	2.425494	-1.086194
C	3.319282	2.407525	-0.504552
C	3.102830	3.284293	0.729332
H	2.253395	2.928840	1.321210
H	2.908633	4.323014	0.441408
H	3.992418	3.276827	1.370422
C	1.050817	0.743772	2.858379
C	-0.102639	0.932124	3.661831
H	-0.258323	1.756953	4.344994
H	-2.049194	-0.111752	3.575085
H	2.056293	1.118460	3.019877
C	-0.409326	-0.740612	2.190262
H	-0.640673	-1.780280	1.954919
Al	-0.041261	0.716977	0.530514
N	1.363100	0.204898	-0.793865
H	4.652091	2.366797	-2.246713
H	1.176655	-4.219748	1.114335
C	1.829777	-3.071590	-1.323273
C	-0.000647	0.558594	-2.762146
H	0.036792	0.690380	-3.837593
C	-1.273435	0.521787	-2.178510
C	-2.474788	0.665211	-3.067585

H	-2.205319	0.616346	-4.123816
H	-2.957677	1.632212	-2.880904
H	-3.229458	-0.097531	-2.856317
H	-2.376909	2.599585	-0.607050
C	-3.281997	2.497758	0.005884
C	-2.968745	3.069803	1.389197
H	-2.140180	2.533415	1.866628
H	-2.693162	4.127572	1.324354
H	-3.841236	2.990095	2.048423
C	-4.398129	3.291283	-0.669180
H	-4.649379	2.886022	-1.654853
H	-4.106792	4.338402	-0.797653
H	-5.315559	3.282268	-0.070384
C	-3.609526	1.022674	0.110554
C	-2.717404	0.029506	-0.328802
C	-2.999926	-1.342887	-0.141328
C	-1.010957	-0.025102	3.278203
C	-4.213875	-1.692644	0.451527
H	-4.451311	-2.742962	0.600343
C	-5.117196	-0.721136	0.870071
H	-6.055002	-1.013919	1.334126
C	-4.808666	0.621693	0.707144
H	-5.505972	1.381686	1.054630
N	-1.437742	0.374419	-0.862333
H	-2.969032	-4.242527	0.116112
O	1.005206	-0.543871	2.343767

Furan_Int-2.log
SCF (M06L) = -1471.23491524
E(SCF)+ZPE(0 K)= -1470.525851
H(298 K)= -1470.485452
G(298 K)= -1470.594567
Lowest Frequency = 30.6794cm-1

Al	-6.041831	-2.303450	-10.966356
O	-3.849031	-2.456502	-10.581422
N	-7.772586	-1.725153	-11.592835
N	-6.742371	-3.723743	-9.873006
C	-8.945779	-2.061358	-11.047683
C	-9.051614	-3.025713	-10.035700
H	-10.049941	-3.217910	-9.659259
C	-8.035985	-3.859256	-9.545636
C	-10.206568	-1.405718	-11.527525
H	-10.199771	-1.272174	-12.612941
H	-10.308836	-0.405526	-11.091565
H	-11.086209	-1.984652	-11.242584
C	-8.457006	-4.997693	-8.663373
H	-7.731455	-5.188429	-7.868606
H	-8.526027	-5.923726	-9.245749
H	-9.438131	-4.808674	-8.225073
C	-7.695721	-0.807620	-12.692296
C	-7.416010	-1.340118	-13.970141
C	-7.230318	-0.450085	-15.028020
H	-7.014280	-0.837427	-16.019703
C	-7.299961	0.924646	-14.831145
H	-7.141780	1.602064	-15.665684
C	-7.564202	1.428393	-13.566198
H	-7.605833	2.504857	-13.412498
C	-7.768267	0.580064	-12.474150
C	-7.337291	-2.840903	-14.182391
H	-6.869014	-3.272065	-13.285834
C	-6.467524	-3.242419	-15.365895
H	-6.916310	-2.955995	-16.323631
H	-6.338196	-4.328808	-15.386385
H	-5.473965	-2.786932	-15.309563
C	-8.729863	-3.461808	-14.300602
H	-9.326637	-3.301500	-13.397388
H	-8.660065	-4.543763	-14.459676
H	-9.279235	-3.034416	-15.147391
C	-8.017691	1.184751	-11.105776
H	-8.284874	0.376567	-10.414335
C	-9.172574	2.185623	-11.131256
H	-8.920213	3.069468	-11.727044
H	-9.405800	2.534230	-10.120352
H	-10.083278	1.756334	-11.561851
C	-6.753144	1.845488	-10.558044
H	-5.942473	1.120416	-10.450465
H	-6.942731	2.296300	-9.578373
H	-6.405259	2.639305	-11.229155
C	-5.829779	-4.777326	-9.519534
C	-4.908172	-4.572418	-8.476123
C	-4.024778	-5.607543	-8.166066
H	-3.312947	-5.470685	-7.355895
C	-4.031839	-6.799570	-8.879213
H	-3.331181	-7.590458	-8.626086
C	-4.928300	-6.971640	-9.925057
H	-4.917532	-7.897083	-10.497099
C	-5.843509	-5.972574	-10.265470
C	-4.887338	-3.285284	-7.677368
H	-5.377385	-2.511765	-8.280936
C	-3.472409	-2.798036	-7.385120
H	-2.946687	-3.461007	-6.688816
H	-3.503425	-1.808622	-6.917039
H	-2.886560	-2.727320	-8.305098
C	-5.690657	-3.434682	-6.384713
H	-6.738555	-3.685616	-6.579991
H	-5.675256	-2.505954	-5.804812
H	-5.271895	-4.227680	-5.754157
C	-6.765107	-6.184055	-11.454101
H	-7.535253	-5.401543	-11.447362
C	-7.479808	-7.533119	-11.410754
H	-6.777363	-8.365345	-11.526844
H	-8.206449	-7.609435	-12.225817
H	-8.013535	-7.689589	-10.467516
C	-5.985011	-6.038986	-12.761532
H	-5.510081	-5.055443	-12.833259
H	-6.643857	-6.167098	-13.628224
H	-5.194433	-6.795041	-12.828144
C	-4.316343	-2.662711	-11.980677
H	-3.879382	-3.579866	-12.379271
C	-3.958319	-1.368928	-12.620074
H	-3.747025	-1.224141	-13.674176
C	-4.055317	-0.422821	-11.663432
H	-3.936452	0.649186	-11.778605
C	-4.477637	-1.117075	-10.417242
H	-4.188623	-0.686621	-9.456269

Furan_TS-2.log
SCF (M06L) = -1471.21319590
E(SCF)+ZPE(0 K)= -1470.506606
H(298 K)= -1470.466359
G(298 K)= -1470.576079
Lowest Frequency = -527.2324cm-1

Al	-5.994453	-2.301125	-10.933096
O	-4.207009	-2.265460	-10.383842

N	-7.757517	-1.740997	-11.536771
N	-6.697269	-3.730854	-9.872147
C	-8.929967	-2.170926	-11.071357
C	-9.023669	-3.115646	-10.036829
H	-10.022176	-3.346525	-9.683012
C	-7.983419	-3.887839	-9.514165
C	-10.216005	-1.718269	-11.698641
H	-10.527440	-2.449166	-12.454079
H	-10.125763	-0.753874	-12.201232
H	-11.015050	-1.668052	-10.955530
C	-8.341699	-4.958302	-8.526838
H	-9.422340	-5.036207	-8.402853
H	-7.894295	-4.742770	-7.550945
H	-7.946914	-5.931964	-8.832427
C	-7.702783	-0.870259	-12.676243
C	-7.744866	-1.428229	-13.967552
C	-7.596123	-0.572072	-15.061350
H	-7.623680	-0.989430	-16.066354
C	-7.408189	0.791501	-14.885448
H	-7.291958	1.441902	-15.748027
C	-7.365419	1.323457	-13.602048
H	-7.213532	2.391628	-13.471178
C	-7.503251	0.511327	-12.474763
C	-7.934369	-2.913869	-14.210890
H	-8.057963	-3.411743	-13.239718
C	-6.711790	-3.529121	-14.890710
H	-6.531103	-3.067669	-15.868160
H	-6.859416	-4.602353	-15.055757
H	-5.807980	-3.395609	-14.287797
C	-9.187944	-3.187951	-15.042319
H	-10.082425	-2.735457	-14.602913
H	-9.363742	-4.264580	-15.137969
H	-9.085189	-2.782209	-16.055062
C	-7.474722	1.107102	-11.079792
H	-7.073277	0.336062	-10.410828
C	-8.880825	1.462612	-10.593665
H	-9.364140	2.171937	-11.275841
H	-8.841070	1.928453	-9.603490
H	-9.525551	0.583000	-10.515756
C	-6.560351	2.321017	-10.965647
H	-5.564809	2.114880	-11.369203
H	-6.446569	2.610252	-9.916493
H	-6.962820	3.192343	-11.494500
C	-5.744754	-4.728845	-9.461403
C	-5.125633	-4.656474	-8.201912
C	-4.209056	-5.653911	-7.860138
H	-3.718603	-5.608900	-6.889727
C	-3.905558	-6.684510	-8.737385
H	-3.191654	-7.452375	-8.452364
C	-4.501488	-6.720629	-9.992592
H	-4.239788	-7.516653	-10.684071
C	-5.420500	-5.745298	-10.385487
C	-5.376390	-3.518971	-7.232172
H	-6.190795	-2.902750	-7.635003
C	-4.133455	-2.634636	-7.115368
H	-3.309258	-3.193769	-6.656942
H	-4.334388	-1.765214	-6.479912
H	-3.800027	-2.293186	-8.098557
C	-5.795607	-4.015845	-5.848731
H	-6.667653	-4.677393	-5.881795
H	-6.037821	-3.173474	-5.193201
H	-4.985412	-4.575396	-5.368317
C	-6.051417	-5.787449	-11.768627
H	-6.183333	-4.747680	-12.102586
C	-7.440770	-6.427967	-11.754843
H	-7.395828	-7.450878	-11.363033
H	-7.850877	-6.478455	-12.770251
H	-8.152211	-5.863046	-11.145808
C	-5.170562	-6.478325	-12.803926
H	-4.148548	-6.087460	-12.797811
H	-5.581972	-6.333899	-13.807444
H	-5.114056	-7.559597	-12.635944
C	-4.546208	-2.566119	-12.286097
H	-4.050734	-3.498504	-12.576237
C	-4.002901	-1.332693	-12.763506
H	-3.430251	-1.234430	-13.680936
C	-4.142618	-0.337986	-11.832739
H	-3.862083	0.701940	-11.991249
C	-4.611952	-0.834080	-10.562638
H	-4.384214	-0.236781	-9.678522

4a.log

SCF (M06L) = -1471.34967794
E(SCF)+ZPE(0 K)= -1470.639496
H(298 K)= -1470.598877
G(298 K)= -1470.708940
Lowest Frequency = 24.9019cm-1

Al	-6.044170	-2.231908	-10.901297
O	-5.577858	-0.980686	-9.706776
N	-7.740738	-1.726747	-11.638106
N	-6.730366	-3.725236	-9.921564
C	-8.901258	-2.014359	-11.034533
C	-8.998381	-2.917445	-9.964988
H	-9.983068	-3.040075	-9.529325
C	-8.001331	-3.783379	-9.498796
C	-10.168856	-1.387072	-11.530966
H	-10.277542	-1.526179	-12.611285
H	-10.158007	-0.305312	-11.364321
H	-11.041309	-1.806209	-11.029012
C	-8.395643	-4.844761	-8.517197
H	-7.754449	-4.808075	-7.630269
H	-8.258636	-5.843941	-8.943372
H	-9.436107	-4.736742	-8.210005
C	-7.753058	-0.900703	-12.815396
C	-7.620448	-1.529365	-14.071510
C	-7.664198	-0.730145	-15.215273
H	-7.572051	-1.193405	-16.193229
C	-7.810244	0.649202	-15.125858
H	-7.843929	1.252192	-16.029041
C	-7.883590	1.253331	-13.880088
H	-7.956350	2.336636	-13.808113
C	-7.848595	0.498641	-12.704157
C	-7.438899	-3.032497	-14.180502
H	-6.812354	-3.346895	-13.332406
C	-6.710052	-3.449189	-15.452415
H	-7.325332	-3.294069	-16.345563
H	-6.464082	-4.514280	-15.417808
H	-5.778215	-2.891281	-15.584630
C	-8.767790	-3.780234	-14.058621
H	-9.244245	-3.621052	-13.086121
H	-8.615542	-4.859589	-14.175740
H	-9.469524	-3.458112	-14.836819
C	-7.826048	1.215003	-11.368817
H	-7.900345	0.466503	-10.571012
C	-8.975378	2.208888	-11.209151

H	-8.898843	3.025810	-11.935208
H	-8.956461	2.660783	-10.212711
H	-9.958095	1.746259	-11.347607
C	-6.487549	1.936101	-11.198751
H	-5.639588	1.262479	-11.349571
H	-6.401260	2.366034	-10.195179
H	-6.396760	2.754026	-11.923269
C	-5.854128	-4.822289	-9.612134
C	-5.016869	-4.769951	-8.483387
C	-4.172750	-5.855879	-8.236062
H	-3.518193	-5.824568	-7.367117
C	-4.153208	-6.962220	-9.074107
H	-3.491255	-7.797241	-8.862129
C	-4.979657	-6.991600	-10.189902
H	-4.959524	-7.853364	-10.854631
C	-5.837584	-5.929885	-10.482807
C	-4.984966	-3.576602	-7.551335
H	-5.737728	-2.855806	-7.890165
C	-3.623806	-2.885404	-7.618246
H	-2.832454	-3.543312	-7.239763
H	-3.620304	-1.975595	-7.008747
H	-3.362168	-2.601336	-8.641695
C	-5.312690	-3.969143	-6.111498
H	-6.286986	-4.462013	-6.026595
H	-5.327944	-3.086346	-5.464771
H	-4.563682	-4.658318	-5.705206
C	-6.704427	-6.000563	-11.725430
H	-7.294554	-5.077489	-11.788107
C	-7.682996	-7.172674	-11.667923
H	-7.152719	-8.130704	-11.624683
H	-8.321820	-7.190976	-12.557150
H	-8.335785	-7.120075	-10.790755
C	-5.844505	-6.083743	-12.985029
H	-5.173374	-5.222695	-13.070464
H	-6.474062	-6.118166	-13.881343
H	-5.225111	-6.987543	-12.985361
C	-4.498207	-2.356987	-12.034923
H	-4.342779	-3.000329	-12.902903
C	-3.465669	-1.512650	-11.736779
H	-2.565695	-1.529164	-12.360028
C	-3.416020	-0.561908	-10.652966
H	-2.520093	0.040873	-10.537487
C	-4.409174	-0.357048	-9.744793
H	-4.277168	0.384076	-8.950897

2-MeFuran.log
SCF (M06L) = -269.247824773
E(SCF)+ZPE(0 K)= -269.149616
H(298 K)= -269.143330
G(298 K)= -269.178255
Lowest Frequency = 122.3728cm-1

C	-0.938256	-0.295919	0.008314
C	0.422345	-0.294013	-0.083057
C	0.833210	1.077490	-0.057161
C	-0.308372	1.806645	0.048010
O	-1.393209	0.987181	0.088959
H	1.056203	-1.166394	-0.159717
H	1.841566	1.463012	-0.109887
H	-0.515410	2.864442	0.103197
C	-1.962379	-1.370848	0.035122
H	-2.547170	-1.345760	0.961679
H	-2.665675	-1.276762	-0.800126
H	-1.476923	-2.346631	-0.035755

2-MeFuran_Int-1.log
SCF (M06L) = -1510.56475699
E(SCF)+ZPE(0 K)= -1509.828515
H(298 K)= -1509.785042
G(298 K)= -1509.904087
Lowest Frequency = 18.9378cm-1

H	-1.317495	2.850187	1.657135
C	-1.634843	3.404928	0.769803
H	-2.598874	3.877848	0.991627
H	-0.904001	4.205047	0.605444
C	-1.741436	2.511431	-0.466818
H	-0.766715	2.009372	-0.592092
C	-1.960548	3.360545	-1.711780
H	-2.037907	2.741560	-2.610624
H	-1.122468	4.050424	-1.849282
C	1.412481	0.414748	2.454228
C	2.590739	0.730712	3.329662
H	3.140772	1.594843	2.942411
H	3.305720	-0.099269	3.340415
H	2.283951	0.942085	4.354985
H	3.275353	3.773475	1.457681
H	2.215452	4.718185	0.400747
H	3.807375	4.213431	-0.166742
C	2.322278	2.615708	-0.132336
C	1.855212	2.821832	-1.573391
H	1.334303	1.934730	-1.956841
H	2.703827	3.020324	-2.237958
C	4.537403	1.530083	-0.631398
H	4.812276	2.446286	-1.150873
C	5.434922	0.472375	-0.579056
H	6.409047	0.557946	-1.052490
C	5.078487	-0.698749	0.078016
H	5.778741	-1.530788	0.111290
C	3.831711	-0.835516	0.691495
H	5.455813	-2.932941	1.746843
C	4.547920	-2.658130	2.294524
H	4.209857	-3.553835	2.824778
C	2.932480	0.246590	0.629115
C	3.277357	1.440499	-0.034824
H	1.435786	2.385587	0.473917
H	2.556286	-1.970964	1.959076
C	3.457391	-2.144459	1.357797
C	3.110066	-3.195535	0.303181
H	2.297461	-2.851975	-0.347836
H	2.801873	-4.136992	0.770220
H	3.976411	-3.404412	-0.334827
C	-1.355379	-1.917296	-3.380847
C	-2.517774	-1.277497	-3.064178
H	-3.360773	-1.704480	-2.537882
H	-3.128416	0.847281	-3.477895
C	-1.161910	0.139947	-4.117949
H	-0.615274	0.926388	-4.614878
Al	0.262326	-0.443006	-0.251340
N	1.609298	0.119360	1.165253
H	4.832327	-1.910568	3.042538
H	1.170895	3.675107	-1.643327
C	2.939876	3.897496	0.423315
C	0.138517	0.469443	3.032116
H	0.098553	0.682187	4.094503
C	-1.090226	0.401442	2.363046

C	-2.329802	0.668721	3.170973
H	-2.082123	0.958407	4.193220
H	-2.973166	-0.217088	3.210148
H	-2.933139	1.460490	2.715223
H	-2.189206	-1.932055	1.875468
C	-3.045125	-2.115896	1.214593
C	-2.590327	-3.123051	0.157394
H	-1.768987	-2.716049	-0.445721
H	-2.253931	-4.058939	0.616473
H	-3.414083	-3.361047	-0.527270
C	-4.172484	-2.691099	2.065889
H	-4.543867	-1.967595	2.799459
H	-3.829505	-3.576588	2.609606
H	-5.025660	-3.003400	1.454232
C	-3.409113	-0.806607	0.542696
C	-2.483591	0.249602	0.444772
C	-2.777614	1.413814	-0.296972
C	-2.392986	0.058474	-3.547503
C	-4.045555	1.525062	-0.871781
H	-4.297264	2.417199	-1.439061
C	-4.983894	0.506815	-0.753002
H	-5.963212	0.612799	-1.211738
C	-4.657698	-0.654215	-0.064669
H	-5.382975	-1.462370	0.000667
N	-1.190271	0.141267	1.053401
H	-2.865981	3.973304	-1.637662
O	-0.519468	-1.057927	-4.032585
C	-0.840007	-3.281556	-3.147952
H	-0.485913	-3.745469	-4.074314
H	0.000637	-3.270920	-2.442612
H	-1.626092	-3.912596	-2.727969

2-MeFuran_TS-1.log

SCF (M06L) = -1510.53870894
E(SCF)+ZPE(0 K)= -1509.802572
H(298 K)= -1509.760806
G(298 K)= -1509.872828
Lowest Frequency = -155.0927cm-1

H	-1.184795	2.821879	1.420343
C	-1.579626	3.393183	0.575918
H	-2.504141	3.887192	0.896762
H	-0.849536	4.178300	0.346563
C	-1.825134	2.523147	-0.658454
H	-0.889556	1.979440	-0.861451
C	-2.099147	3.418940	-1.861143
H	-2.316370	2.847530	-2.769280
H	-1.230081	4.051553	-2.064769
C	1.368206	0.244154	2.231680
C	2.593048	0.403075	3.084894
H	3.296609	1.121156	2.654080
H	3.131251	-0.550891	3.140602
H	2.341468	0.716718	4.099065
H	2.165813	2.910178	1.960027
H	1.530797	4.289008	1.061784
H	3.253867	3.894172	0.968983
C	1.978059	2.568092	-0.193269
C	1.874593	3.412436	-1.458440
H	1.681316	2.800459	-2.345467
H	2.784732	3.992707	-1.645299
C	4.217113	1.656328	-0.954546
H	4.424786	2.624234	-1.404771
C	5.159217	0.638161	-1.051904
H	6.096958	0.812200	-1.572323
C	4.890917	-0.604916	-0.495809
H	5.619393	-1.407522	-0.592099
C	3.694216	-0.852716	0.182220
H	5.455083	-2.924878	1.006592
C	4.540112	-2.775571	1.590320
H	4.271413	-3.745142	2.021141
C	2.762589	0.193862	0.291818
C	3.003306	1.454449	-0.296441
H	1.001071	2.092734	-0.033048
H	2.504744	-2.172017	1.348574
C	3.404982	-2.237044	0.723519
C	3.096242	-3.193272	-0.429352
H	2.297654	-2.800568	-1.068444
H	2.790568	-4.176160	-0.054009
H	3.981391	-3.335395	-1.060622
C	-0.411133	-2.244300	-2.280916
C	-1.533722	-1.413598	-2.579602
H	-2.568300	-1.738498	-2.585687
H	-1.637739	0.774073	-2.814756
C	0.345528	-0.199446	-2.341325
H	1.086717	0.516791	-2.694964
Al	0.058086	-0.773772	-0.228846
N	1.497871	-0.007137	0.928305
H	4.786543	-2.096397	2.413366
H	1.054421	4.132723	-1.362017
C	2.247675	3.461886	1.018868
C	0.110667	0.362141	2.841687
H	0.105716	0.545624	3.910058
C	-1.133811	0.419867	2.195998
C	-2.315909	0.819742	3.032328
H	-2.006914	1.128006	4.031779
H	-3.025007	-0.008764	3.130870
H	-2.870091	1.639157	2.564118
H	-2.377112	-1.848884	1.829427
C	-3.319427	-1.971441	1.280668
C	-3.142034	-3.160667	0.336820
H	-2.292418	-3.000913	-0.333758
H	-2.964368	-4.083453	0.899317
H	-4.039126	-3.309008	-0.276546
C	-4.422024	-2.265405	2.297636
H	-4.614655	-1.416583	2.962185
H	-4.153116	-3.125458	2.918542
H	-5.368615	-2.508892	1.803148
C	-3.585843	-0.698610	0.498156
C	-2.621114	0.313940	0.334931
C	-2.894131	1.466458	-0.436513
C	-1.054559	-0.130445	-2.700718
C	-4.155987	1.588173	-1.019082
H	-4.387041	2.465575	-1.615926
C	-5.120732	0.597792	-0.865215
H	-6.094478	0.709011	-1.334082
C	-4.830089	-0.533582	-0.119222
H	-5.579913	-1.314656	-0.010547
N	-1.308405	0.159469	0.891254
H	-2.948089	4.088512	-1.682331
O	0.726396	-1.544231	-2.652959
C	-0.328006	-3.721658	-2.419882
H	-0.229236	-4.013530	-3.474145
H	0.527689	-4.133162	-1.879699
H	-1.234408	-4.191107	-2.029105

2-MeFuran_Int-2.log

SCF (M06L) = -1510.55141665
E(SCF)+ZPE(0 K)= -1509.814062
H(298 K)= -1509.772405
G(298 K)= -1509.884051
Lowest Frequency = 21.5045cm-1

Al	-6.015725	-2.370988	-10.975638
O	-3.779130	-2.363919	-10.505479
N	-7.725230	-1.760519	-11.643627
N	-6.777865	-3.737082	-9.840989
C	-8.922595	-2.174219	-11.215885
C	-9.077783	-3.078436	-10.155022
H	-10.096213	-3.278650	-9.841045
C	-8.080124	-3.845179	-9.538383
C	-10.163535	-1.707987	-11.918226
H	-10.294565	-2.280503	-12.844516
H	-10.106318	-0.656771	-12.210252
H	-11.050428	-1.865706	-11.302469
C	-8.529639	-4.877077	-8.542798
H	-7.793801	-5.019111	-7.748139
H	-8.662343	-5.854442	-9.020039
H	-9.488179	-4.594782	-8.103626
C	-7.638440	-0.822110	-12.727818
C	-7.656520	-1.283678	-14.055388
C	-7.508304	-0.347474	-15.082509
H	-7.512114	-0.690942	-16.115202
C	-7.345608	1.001610	-14.805424
H	-7.232455	1.715489	-15.616611
C	-7.311623	1.437524	-13.485420
H	-7.163523	2.493297	-13.276290
C	-7.445840	0.542779	-12.422721
C	-7.763342	-2.755514	-14.403259
H	-7.976050	-3.314309	-13.481636
C	-6.429113	-3.261417	-14.953076
H	-6.157035	-2.722495	-15.867878
H	-6.477591	-4.328759	-15.193930
H	-5.622950	-3.112092	-14.227682
C	-8.892060	-3.038752	-15.392234
H	-9.855921	-2.657576	-15.039381
H	-8.995656	-4.115507	-15.561322
H	-8.697008	-2.575400	-16.365573
C	-7.387038	1.025034	-10.984092
H	-6.886446	0.238312	-10.403208
C	-8.782545	1.217989	-10.386840
H	-9.373076	1.917321	-10.990477
H	-8.713329	1.630125	-9.374870
H	-9.338961	0.279754	-10.317363
C	-6.572225	2.301492	-10.810064
H	-5.588059	2.227097	-11.281663
H	-6.420700	2.507486	-9.746426
H	-7.081385	3.174349	-11.234301
C	-5.876314	-4.773275	-9.417484
C	-4.918844	-4.499179	-8.423262
C	-4.010872	-5.507474	-8.091345
H	-3.265029	-5.316079	-7.324468
C	-4.041710	-6.741924	-8.724766
H	-3.322147	-7.510334	-8.455876
C	-4.993815	-6.994361	-9.704970
H	-5.009000	-7.962251	-10.198789
C	-5.928076	-6.023705	-10.072242
C	-4.897612	-3.178072	-7.680460
H	-5.363840	-2.423644	-8.325665
C	-3.487588	-2.692300	-7.365852
H	-2.993647	-3.324776	-6.619563
H	-3.524666	-1.681673	-6.946378
H	-2.872668	-2.668056	-8.268527
C	-5.734803	-3.265373	-6.402928
H	-6.783634	-3.498482	-6.612758
H	-5.710878	-2.316715	-5.856524
H	-5.348244	-4.043832	-5.734827
C	-6.920617	-6.303274	-11.189740
H	-7.824557	-5.709387	-11.011277
C	-7.354377	-7.763913	-11.257634
H	-6.540650	-8.416859	-11.590291
H	-8.170392	-7.884327	-11.976445
H	-7.699374	-8.135156	-10.287128
C	-6.368956	-5.843283	-12.538117
H	-6.104481	-4.779403	-12.520543
H	-7.106986	-5.997876	-13.334226
H	-5.461763	-6.398396	-12.803155
C	-4.199429	-2.745917	-11.878700
C	-3.959555	-1.473176	-12.621631
H	-3.766451	-1.402345	-13.688074
C	-4.157410	-0.461599	-11.750561
H	-4.158483	0.602103	-11.965299
C	-4.525111	-1.085617	-10.452229
C	-3.485809	-3.994058	-12.301683
H	-3.700345	-4.828389	-11.624391
H	-3.796154	-4.297492	-13.308028
H	-2.398846	-3.842008	-12.316218
H	-4.294430	-0.548715	-9.529858

2-MeFuran_TS-2.log
SCF (M06L) = -1510.53354628
E(SCF)+ZPE(0 K)= -1509.798331
H(298 K)= -1509.756942
G(298 K)= -1509.867143
Lowest Frequency = -446.8142cm-1

Al	-5.939967	-2.355042	-10.952849
O	-4.149579	-2.303291	-10.384523
N	-7.699959	-1.802414	-11.603090
N	-6.655554	-3.785314	-9.900600
C	-8.862281	-2.119558	-11.033107
C	-8.953867	-3.053656	-9.987011
H	-9.942946	-3.214638	-9.573103
C	-7.933926	-3.867987	-9.490137
C	-10.141853	-1.482459	-11.490469
H	-10.982048	-2.168051	-11.363272
H	-10.092328	-1.164249	-12.534340
H	-10.357181	-0.591663	-10.889919
C	-8.307463	-4.901379	-8.469886
H	-7.796522	-4.704946	-7.522140
H	-7.991732	-5.901360	-8.782366
H	-9.383059	-4.909640	-8.290414
C	-7.638750	-0.864526	-12.687621
C	-7.374230	-1.371472	-13.979035
C	-7.201336	-0.465194	-15.024694
H	-6.997873	-0.836773	-16.025001
C	-7.269628	0.906095	-14.806004
H	-7.120362	1.597488	-15.630728
C	-7.530705	1.386288	-13.532150
H	-7.585110	2.459836	-13.361325
C	-7.725350	0.520561	-12.451202
C	-7.307494	-2.866712	-14.224063
H	-6.819439	-3.313811	-13.347433

C	-6.471818	-3.245500	-15.440045
H	-6.959017	-2.960268	-16.379272
H	-6.325110	-4.330128	-15.475135
H	-5.485473	-2.770452	-15.416878
C	-8.703880	-3.482219	-14.320444
H	-9.276133	-3.343505	-13.397895
H	-8.639604	-4.559676	-14.509438
H	-9.274560	-3.032671	-15.141262
C	-8.025106	1.118582	-11.089387
H	-8.200056	0.299684	-10.380815
C	-9.281564	1.991298	-11.136498
H	-9.112280	2.888400	-11.742188
H	-9.559791	2.327080	-10.132315
H	-10.138448	1.467657	-11.571287
C	-6.849873	1.937551	-10.557986
H	-5.959448	1.318101	-10.435546
H	-7.095424	2.381279	-9.587504
H	-6.601896	2.757037	-11.242450
C	-5.742829	-4.824276	-9.501559
C	-5.039736	-4.741067	-8.287107
C	-4.174305	-5.787416	-7.954767
H	-3.622259	-5.735105	-7.018291
C	-3.999746	-6.875484	-8.796125
H	-3.323940	-7.679344	-8.517498
C	-4.676856	-6.925324	-10.009656
H	-4.515861	-7.768097	-10.675940
C	-5.550060	-5.904700	-10.391755
C	-5.157698	-3.560070	-7.343459
H	-5.905851	-2.869601	-7.754740
C	-3.828685	-2.809088	-7.244676
H	-3.063545	-3.442568	-6.780242
H	-3.938106	-1.917194	-6.617959
H	-3.473729	-2.510122	-8.233671
C	-5.611408	-3.984875	-5.945899
H	-6.552888	-4.543493	-5.951579
H	-5.745420	-3.109941	-5.302025
H	-4.860888	-4.625228	-5.468960
C	-6.256738	-5.950235	-11.738143
H	-6.258566	-4.920060	-12.129293
C	-7.718085	-6.395485	-11.632781
H	-7.792114	-7.361020	-11.118669
H	-8.150020	-6.521299	-12.631328
H	-8.344152	-5.675359	-11.100996
C	-5.545046	-6.825927	-12.764488
H	-4.474603	-6.611132	-12.827823
H	-5.981244	-6.667018	-13.756387
H	-5.659337	-7.891232	-12.534110
C	-4.468150	-2.539059	-12.310718
C	-4.005254	-1.233796	-12.700185
H	-3.475968	-1.052332	-13.632899
C	-4.177303	-0.303879	-11.714903
H	-3.972888	0.759644	-11.825989
C	-4.617503	-0.889665	-10.467758
C	-3.784170	-3.761938	-12.815089
H	-3.732191	-4.517659	-12.023925
H	-4.375074	-4.210997	-13.626559
H	-2.768429	-3.583221	-13.188238
H	-4.405275	-0.336054	-9.551703

2-MeFuran_TS-2'.log
SCF (M06L) = -1510.52963883
E(SCF)+ZPE(0 K)= -1509.795258
H(298 K)= -1509.753567
G(298 K)= -1509.865060
Lowest Frequency = -534.8813cm-1

Al	-5.977435	-2.267056	-10.917518
O	-4.191786	-2.203230	-10.352154
N	-7.745393	-1.718206	-11.526979
N	-6.671673	-3.705705	-9.860048
C	-8.914313	-2.153606	-11.057000
C	-9.000456	-3.093014	-10.016940
H	-9.997084	-3.326478	-9.659550
C	-7.957488	-3.866586	-9.501241
C	-10.204321	-1.716168	-11.687320
H	-10.502949	-2.449022	-12.446099
H	-10.125676	-0.749173	-12.186928
H	-11.006494	-1.678852	-10.946836
C	-8.315173	-4.949017	-8.526669
H	-9.393782	-5.004632	-8.376159
H	-7.835584	-4.768199	-7.559706
H	-7.953096	-5.924935	-8.864366
C	-7.701805	-0.862241	-12.678345
C	-7.748236	-1.438160	-13.961624
C	-7.610112	-0.597348	-15.068737
H	-7.639150	-1.029719	-16.067286
C	-7.431308	0.769568	-14.913686
H	-7.323411	1.408459	-15.785879
C	-7.387191	1.320218	-13.638284
H	-7.242500	2.391081	-13.525100
C	-7.512950	0.524125	-12.498160
C	-7.928987	-2.927901	-14.183830
H	-8.056532	-3.411650	-13.206277
C	-6.697839	-3.545333	-14.845873
H	-6.516040	-3.099921	-15.830541
H	-6.836202	-4.622619	-14.991904
H	-5.798327	-3.393473	-14.240520
C	-9.175533	-3.222110	-15.018822
H	-10.075250	-2.767294	-14.592586
H	-9.345104	-4.301070	-15.097665
H	-9.068799	-2.833020	-16.037629
C	-7.477844	1.141121	-11.112125
H	-7.016084	0.401138	-10.445644
C	-8.884791	1.428731	-10.585019
H	-9.432371	2.090160	-11.266812
H	-8.836918	1.923402	-9.609325
H	-9.473417	0.516309	-10.459267
C	-6.629854	2.405322	-11.043154
H	-5.635829	2.249701	-11.474465
H	-6.503042	2.718558	-10.002270
H	-7.096182	3.243426	-11.573345
C	-5.729317	-4.724285	-9.474266
C	-5.080126	-4.672768	-8.228770
C	-4.205670	-5.711238	-7.897690
H	-3.697619	-5.684452	-6.935597
C	-3.965174	-6.758827	-8.773715
H	-3.285171	-7.559355	-8.495716
C	-4.577042	-6.767295	-10.021574
H	-4.359025	-7.573386	-10.716503
C	-5.457575	-5.752715	-10.403110
C	-5.247623	-3.519854	-7.259130
H	-5.992874	-2.829614	-7.676894
C	-3.927831	-2.760338	-7.112095
H	-3.168898	-3.398998	-6.645028
H	-4.055172	-1.880712	-6.471341
H	-3.550856	-2.437854	-8.086008

C	-5.734640	-3.978693	-5.884448
H	-6.674102	-4.539012	-5.927991
H	-5.888806	-3.119730	-5.223799
H	-4.994633	-4.627855	-5.403175
C	-6.096813	-5.765819	-11.783161
H	-6.205214	-4.719370	-12.104288
C	-7.499200	-6.377433	-11.774601
H	-7.477005	-7.401149	-11.382773
H	-7.905000	-6.420714	-12.792189
H	-8.202222	-5.797584	-11.169826
C	-5.232995	-6.464893	-12.827791
H	-4.203044	-6.095593	-12.820730
H	-5.643830	-6.301858	-13.828620
H	-5.199150	-7.548749	-12.671161
C	-4.510995	-2.550552	-12.238257
H	-4.001248	-3.481914	-12.505114
C	-3.985944	-1.316635	-12.736077
H	-3.430319	-1.221549	-13.663971
C	-4.150694	-0.306721	-11.828259
H	-3.902474	0.739089	-12.010001
C	-4.611513	-0.768760	-10.538405
C	-4.310901	0.063535	-9.328097
H	-4.904064	-0.222558	-8.452741
H	-4.516046	1.120768	-9.531754
H	-3.252313	-0.023049	-9.043854

4b.log
SCF (M06L) = -1510.66213070
E(SCF)+ZPE(0 K)= -1509.923921
H(298 K)= -1509.881926
G(298 K)= -1509.994286
Lowest Frequency = 20.1831cm-1

Al	-1.365364	3.921919	5.422811
N	-1.815934	5.779212	5.479590
N	-0.745812	3.966273	3.598320
C	-1.086006	6.682844	4.809949
C	-0.270335	6.330545	3.727629
H	0.279610	7.137910	3.258174
C	-0.194246	5.079202	3.093393
C	-1.142958	8.124180	5.218380
H	-2.173079	8.476376	5.321591
H	-0.616937	8.763386	4.508340
H	-0.678911	8.245684	6.204345
C	0.511257	5.022429	1.771629
H	1.423244	4.421685	1.838697
H	0.779928	6.020598	1.424897
H	-0.115932	4.539766	1.015372
C	-2.857147	6.228284	6.362316
C	-4.161845	6.350281	5.836190
C	-5.181638	6.767887	6.692942
H	-6.192783	6.869203	6.307506
C	-4.927620	7.045174	8.031479
H	-5.735781	7.365322	8.683321
C	-3.643034	6.898645	8.533972
H	-3.448237	7.097629	9.585872
C	-2.585972	6.489244	7.715637
C	-4.448057	6.054954	4.375462
H	-3.731867	5.285450	4.056026
C	-4.210230	7.284484	3.494569
H	-4.808919	8.133677	3.844224
H	-4.497939	7.079230	2.457831
H	-3.160915	7.591651	3.484272
C	-5.850982	5.510321	4.130075
H	-6.080889	4.656063	4.773982
H	-5.948977	5.185143	3.088479
H	-6.620558	6.272547	4.294717
C	-1.209477	6.290898	8.314476
H	-0.504922	6.089540	7.498229
C	-1.210797	5.065219	9.227345
H	-1.861238	5.229689	10.094685
H	-0.202804	4.851567	9.597686
H	-1.570423	4.173354	8.705067
C	-0.722936	7.525274	9.070561
H	-0.734923	8.425532	8.446857
H	0.299989	7.378112	9.430406
H	-1.347538	7.730387	9.947129
C	-0.697877	2.756769	2.819372
C	0.479565	1.987383	2.759490
C	0.477449	0.839003	1.962014
H	1.381128	0.235163	1.909772
C	-0.650652	0.451082	1.255823
H	-0.629121	-0.442513	0.638243
C	-1.818925	1.196423	1.362783
H	-2.710016	0.871512	0.833518
C	-1.870355	2.349037	2.147780
C	1.712276	2.304553	3.582384
H	1.571344	3.280427	4.061964
C	1.864777	1.264381	4.693474
H	2.688298	1.529997	5.364790
H	2.083591	0.277501	4.268692
H	0.957027	1.174786	5.296317
C	2.989406	2.352234	2.744374
H	3.222265	1.370098	2.318186
H	3.842779	2.644826	3.363679
H	2.925623	3.057599	1.909509
C	-3.153525	3.145191	2.278616
H	-3.208624	3.479531	3.324522
C	-3.151560	4.400198	1.404375
H	-4.112394	4.922507	1.482844
H	-3.001055	4.140072	0.350212
H	-2.371453	5.110199	1.695884
C	-4.406506	2.321259	2.008364
H	-4.509214	2.071164	0.946432
H	-5.298525	2.889139	2.291290
H	-4.409463	1.385275	2.575114
O	0.116119	3.647165	6.384932
C	0.221246	2.655062	7.260955
H	1.205034	2.580399	7.731923
C	-0.762999	1.777130	7.590032
H	-0.515709	1.020628	8.328771
C	-2.114443	1.816040	7.086530
H	-2.793921	1.091335	7.554907
C	-2.643703	2.659132	6.147955
C	-4.118907	2.588414	5.880902
H	-4.615991	3.531472	6.153485
H	-4.605530	1.789450	6.456970
H	-4.360116	2.417690	4.822803

5b.log
SCF (M06L) = -1510.67026577
E(SCF)+ZPE(0 K)= -1509.932247
H(298 K)= -1509.890127
G(298 K)= -1510.003421
Lowest Frequency = 19.7982cm-1

Al	-6.034390	-2.213505	-10.917995
O	-5.579001	-0.995292	-9.693110
N	-7.765837	-1.717842	-11.569733
N	-6.652580	-3.765685	-9.976214
C	-8.890893	-2.036690	-10.913808
C	-8.929143	-2.986587	-9.885154
H	-9.890451	-3.139302	-9.408678
C	-7.903137	-3.866263	-9.507598
C	-10.180254	-1.388290	-11.318046
H	-10.444530	-1.648000	-12.348267
H	-10.086337	-0.297422	-11.300291
H	-10.998918	-1.686519	-10.662682
C	-8.254990	-4.982051	-8.570520
H	-7.723351	-4.873495	-7.619906
H	-7.947470	-5.949177	-8.980975
H	-9.326062	-5.006568	-8.367980
C	-7.875950	-0.961083	-12.786885
C	-8.101606	-1.659228	-13.990022
C	-8.206099	-0.926942	-15.173978
H	-8.381859	-1.456675	-16.108601
C	-8.084493	0.456511	-15.177499
H	-8.169127	1.010548	-16.108218
C	-7.844684	1.125302	-13.985319
H	-7.735993	2.208168	-13.986397
C	-7.734803	0.438176	-12.773280
C	-8.216203	-3.170498	-14.043564
H	-8.102859	-3.561756	-13.024693
C	-7.097413	-3.768656	-14.894255
H	-7.151382	-3.413311	-15.929314
H	-7.172693	-4.861623	-14.919090
H	-6.109609	-3.501829	-14.504214
C	-9.581016	-3.619787	-14.562938
H	-10.401597	-3.231669	-13.951165
H	-9.656263	-4.712296	-14.565323
H	-9.747830	-3.277100	-15.590369
C	-7.447227	1.216820	-11.505977
H	-7.432843	0.514340	-10.664355
C	-8.518079	2.271463	-11.230745
H	-8.544176	3.030095	-12.021233
H	-8.315988	2.789830	-10.288125
H	-9.521192	1.837177	-11.164317
C	-6.067027	1.868181	-11.588215
H	-5.277804	1.129104	-11.756211
H	-5.833597	2.404395	-10.662194
H	-6.025649	2.594735	-12.408184
C	-5.701470	-4.794279	-9.652108
C	-5.051053	-4.797936	-8.404737
C	-4.157007	-5.836498	-8.129203
H	-3.646728	-5.850292	-7.168218
C	-3.899199	-6.831253	-9.059718
H	-3.204939	-7.633347	-8.825025
C	-4.510556	-6.782270	-10.306941
H	-4.276896	-7.544496	-11.044396
C	-5.408363	-5.764605	-10.633836
C	-5.215350	-3.682623	-7.391510
H	-6.020022	-3.018105	-7.729308
C	-3.929850	-2.855817	-7.332260
H	-3.094384	-3.468692	-6.974254
H	-4.041488	-2.012710	-6.642063
H	-3.654034	-2.458549	-8.313782
C	-5.564893	-4.198110	-5.996159
H	-6.460058	-4.828546	-5.989640
H	-5.739507	-3.363117	-5.310738
H	-4.747903	-4.794897	-5.575744
C	-6.050358	-5.707126	-12.008012
H	-6.136967	-4.644822	-12.281560
C	-7.465763	-6.287195	-12.004407
H	-7.458268	-7.331577	-11.671201
H	-7.894986	-6.263883	-13.013005
H	-8.140749	-5.727658	-11.349156
C	-5.204952	-6.378876	-13.083544
H	-4.173979	-6.012808	-13.073973
H	-5.623863	-6.182313	-14.074369
H	-5.177316	-7.467193	-12.960627
C	-4.480023	-2.310740	-12.035823
H	-4.301513	-2.933602	-12.913736
C	-3.437721	-1.508645	-11.660666
H	-2.513426	-1.532529	-12.247426
C	-3.394115	-0.591119	-10.549825
H	-2.484466	-0.014611	-10.407618
C	-4.406805	-0.366869	-9.658834
C	-4.292995	0.624166	-8.548372
H	-5.088881	1.374223	-8.626328
H	-3.327996	1.134965	-8.548967
H	-4.430359	0.131486	-7.579026

2,3-DiMeFuran.log
SCF (M06L) = -308.629585380
E(SCF)+ZPE(0 K)= -308.504148
H(298 K)= -308.496116
G(298 K)= -308.535238
Lowest Frequency = 90.1213cm-1

C	-2.962423	0.394959	-0.091644
C	-1.606235	0.390125	-0.130146
C	-1.188805	1.757684	-0.041952
C	-2.337489	2.493517	0.044262
O	-3.430176	1.668660	0.014758
H	-3.716278	-0.375448	-0.126450
H	-0.968209	-0.479011	-0.211706
C	-2.616781	3.939928	0.157700
H	-3.216050	4.307577	-0.682325
H	-3.166977	4.179299	1.074173
H	-1.681973	4.504228	0.172728
C	0.210900	2.263991	-0.044420
H	0.742188	1.986477	-0.961335
H	0.247102	3.354062	0.034115
H	0.791543	1.858479	0.791309

2,3-DiMeFuran_Int-1.log
SCF (M06L) = -1549.88219844
E(SCF)+ZPE(0 K)= -1549.117532
H(298 K)= -1549.072776
G(298 K)= -1549.193140
Lowest Frequency = 21.4707cm-1

H	-1.316227	2.679326	1.876239
C	-1.671900	3.231338	1.003985
H	-2.614069	3.724099	1.271826
H	-0.936325	4.016909	0.797023
C	-1.857638	2.345749	-0.230861
H	-0.904095	1.820358	-0.402749
C	-2.111813	3.231824	-1.443005
H	-2.226759	2.650098	-2.361799
H	-1.269449	3.917340	-1.582712
C	1.372210	0.374773	2.471116

C	2.616356	0.828905	3.177532
H	3.095109	1.642822	2.622441
H	3.358226	0.024836	3.231449
H	2.400793	1.175433	4.189150
H	3.195015	3.598109	0.534647
H	1.961772	4.292318	-0.530326
H	3.457506	3.654867	-1.210808
C	2.029107	2.126429	-0.584832
C	1.360805	1.997040	-1.954368
H	0.769109	1.075169	-2.027823
H	2.105082	1.967748	-2.758801
C	4.186414	0.933800	-1.087799
H	4.401252	1.731301	-1.796428
C	5.087553	-0.110387	-0.932240
H	6.005783	-0.131336	-1.512598
C	4.805915	-1.131717	-0.033557
H	5.507326	-1.955444	0.081755
C	3.630159	-1.131708	0.719426
H	5.364379	-3.017953	1.976265
C	4.510882	-2.641039	2.549998
H	4.232844	-3.423382	3.262968
C	2.725052	-0.064602	0.549980
C	2.996618	0.979980	-0.356492
H	1.243685	2.063273	0.180982
H	2.490721	-2.008623	2.289336
C	3.331168	-2.294564	1.645046
C	2.893106	-3.514997	0.835055
H	2.020584	-3.286673	0.211803
H	2.635988	-4.353369	1.491029
H	3.697370	-3.845015	0.167540
C	-0.411759	-0.568102	-3.926304
C	-1.729506	-0.586236	-3.555624
H	-3.320322	0.954908	-3.957384
C	-1.291835	1.292017	-4.695604
H	-1.229101	2.232656	-5.220552
Al	-0.014417	-0.853016	0.053649
N	1.457582	-0.085844	1.219193
H	4.859522	-1.773572	3.120404
H	0.687067	2.837913	-2.153295
C	2.700919	3.489565	-0.435953
C	0.154952	0.476022	3.158567
H	0.208338	0.792356	4.194270
C	-1.127596	0.356225	2.607171
C	-2.301192	0.684087	3.485673
H	-1.980788	1.022505	4.472161
H	-2.945480	-0.193036	3.612478
H	-2.928501	1.458696	3.033027
H	-2.371343	-2.010115	2.257929
C	-3.296587	-2.164377	1.688566
C	-3.044980	-3.311723	0.709187
H	-2.210821	-3.079913	0.036515
H	-2.809979	-4.240254	1.240103
H	-3.930200	-3.493127	0.087936
C	-4.399587	-2.530005	2.678841
H	-4.622685	-1.708940	3.368483
H	-4.110566	-3.401693	3.273998
H	-5.333075	-2.785935	2.166189
C	-3.598610	-0.889514	0.926333
C	-2.644852	0.133539	0.772654
C	-2.911367	1.269230	-0.028645
C	-2.293787	0.628288	-4.063007
C	-4.164200	1.364572	-0.637677
H	-4.389245	2.224823	-1.261426
C	-5.124052	0.371093	-0.477335
H	-6.090506	0.464674	-0.965001
C	-4.836700	-0.745964	0.292994
H	-5.578896	-1.534670	0.397653
N	-1.332764	0.001498	1.333470
H	-3.004289	3.854139	-1.308984
O	-0.135523	0.579532	-4.626038
C	0.719207	-1.499815	-3.739874
H	1.109460	-1.864734	-4.697128
H	1.551580	-1.026255	-3.203324
H	0.402002	-2.356479	-3.142146
C	-2.453369	-1.643816	-2.799220
H	-1.791672	-2.473027	-2.534067
H	-2.869614	-1.255313	-1.860821
H	-3.291539	-2.050974	-3.377245

2,3-DiMeFuran_TS-1.log
SCF (M06L) = -1549.85781380
E(SCF)+ZPE(0 K)= -1549.093858
H(298 K)= -1549.050565
G(298 K)= -1549.164986
Lowest Frequency = -153.5702cm-1

H	1.996896	-3.220866	1.518325
C	2.220937	-3.606184	0.520503
H	3.244653	-3.998599	0.529801
H	1.543721	-4.447554	0.338517
C	2.057413	-2.539588	-0.564850
H	1.052288	-2.109914	-0.445276
C	2.123202	-3.196027	-1.940333
H	2.096254	-2.462317	-2.752383
H	1.277876	-3.879457	-2.072629
C	-1.174220	-1.047724	2.122242
C	-2.365392	-1.650507	2.812958
H	-3.034487	-0.871170	3.193517
H	-2.058959	-2.272980	3.654751
H	-2.961928	-2.252670	2.121443
H	-1.524026	-3.278894	0.639536
H	-1.086117	-4.212558	-0.792742
H	-2.790742	-4.005331	-0.366966
C	-1.870917	-2.239822	-1.254009
C	-2.031906	-2.595435	-2.725906
H	-2.102586	-1.696623	-3.344748
H	-2.921424	-3.209732	-2.906702
C	-4.166633	-1.167021	-1.360704
H	-4.398100	-1.808412	-2.206379
C	-5.117197	-0.256650	-0.912222
H	-6.085572	-0.195092	-1.401184
C	-4.819619	0.582235	0.152187
H	-5.557126	1.308537	0.487365
C	-3.581175	0.523820	0.799735
H	-5.324177	1.995406	2.530305
C	-4.411944	1.561065	2.953211
H	-4.123019	2.187367	3.802848
C	-2.636462	-0.415816	0.341861
C	-2.913774	-1.259110	-0.754656
H	-0.897406	-1.744487	-1.149858
H	-2.375632	1.176655	2.436992
C	-3.286766	1.503538	1.921276
C	-3.005878	2.897468	1.359829
H	-2.138032	2.882345	0.690917
H	-2.805010	3.610143	2.166964
H	-3.865883	3.268926	0.789612

C	-0.886857	1.918117	-2.012703
C	0.363919	2.453779	-2.473317
H	2.284639	1.422487	-2.789155
C	0.502970	0.225082	-2.128070
H	0.713879	-0.779557	-2.497976
Al	0.045547	0.639310	-0.012549
N	-1.327879	-0.467588	0.925065
H	-4.674340	0.569590	3.337550
H	-1.169841	-3.176833	-3.069008
C	-1.817442	-3.501439	-0.391567
C	0.057386	-1.128945	2.792210
H	0.036863	-1.569132	3.782844
C	1.319121	-0.815301	2.271390
C	2.531636	-1.109741	3.107106
H	2.272144	-1.646516	4.020960
H	3.027629	-0.172062	3.385097
H	3.273360	-1.691802	2.552481
H	2.405461	1.790596	1.956845
C	3.278272	2.030208	1.335400
C	2.866329	3.151068	0.379383
H	2.031235	2.841128	-0.260192
H	2.558151	4.047836	0.927661
H	3.700186	3.426435	-0.278170
C	4.402140	2.486235	2.261347
H	4.726426	1.686384	2.934973
H	4.078297	3.333684	2.873419
H	5.282007	2.815254	1.697771
C	3.637856	0.789963	0.544096
C	2.750796	-0.294016	0.429740
C	3.046282	-1.398666	-0.401222
C	1.216867	1.386707	-2.598790
C	4.270024	-1.410340	-1.072370
H	4.518356	-2.252459	-1.713390
C	5.169969	-0.357202	-0.947787
H	6.116168	-0.384015	-1.481263
C	4.847588	0.734706	-0.154209
H	5.541975	1.568922	-0.077135
N	1.468717	-0.268625	1.062228
H	3.034273	-3.792117	-2.065104
O	-0.886950	0.557479	-2.305095
C	-2.217495	2.575235	-2.102730
H	-2.178862	3.577597	-1.667158
H	-2.543167	2.679097	-3.147954
H	-2.982426	2.003156	-1.569304
C	0.657503	3.906273	-2.600191
H	0.020159	4.398879	-3.344678
H	0.485349	4.427092	-1.648177
H	1.699033	4.080471	-2.883158

2,3-DiMeFuran_Int-2.log
SCF (M06L) = -1549.86727072
E(SCF)+ZPE(0 K)= -1549.101666
H(298 K)= -1549.058549
G(298 K)= -1549.172395
Lowest Frequency = 21.2834cm-1

Al	-6.003862	-2.308051	-10.915295
O	-3.800846	-2.434617	-10.484796
N	-7.743135	-1.721907	-11.560252
N	-6.737357	-3.692867	-9.780697
C	-8.923970	-2.070593	-11.035564
C	-9.044310	-3.016901	-10.008734
H	-10.051304	-3.216821	-9.659556
C	-8.036172	-3.837472	-9.484087
C	-10.191627	-1.457324	-11.557459
H	-10.128876	-1.250889	-12.629037
H	-10.393438	-0.500825	-11.062837
H	-11.045516	-2.109882	-11.367998
C	-8.477564	-4.978298	-8.614010
H	-7.737764	-5.224019	-7.848887
H	-8.616060	-5.882802	-9.217024
H	-9.434320	-4.753830	-8.138827
C	-7.663636	-0.770465	-12.631026
C	-7.307081	-1.246414	-13.912301
C	-7.107734	-0.314958	-14.931313
H	-6.827662	-0.660841	-15.922227
C	-7.254233	1.048144	-14.698359
H	-7.086227	1.758877	-15.502714
C	-7.615233	1.496938	-13.436681
H	-7.724736	2.564287	-13.255111
C	-7.826925	0.605385	-12.380474
C	-7.187231	-2.736316	-14.173094
H	-6.712849	-3.187138	-13.289261
C	-6.310300	-3.082127	-15.368125
H	-6.770278	-2.780711	-16.316112
H	-6.153215	-4.163890	-15.418606
H	-5.329236	-2.602318	-15.301761
C	-8.567814	-3.378885	-14.319467
H	-9.176088	-3.253025	-13.418728
H	-8.477688	-4.454371	-14.508131
H	-9.116298	-2.937437	-15.159648
C	-8.184772	1.156003	-11.012764
H	-8.463287	0.318665	-10.363086
C	-9.370993	2.116908	-11.077582
H	-9.115240	3.031072	-11.623907
H	-9.679727	2.418963	-10.071920
H	-10.236091	1.672175	-11.580333
C	-6.979662	1.833138	-10.364928
H	-6.153601	1.125882	-10.250794
H	-7.233826	2.224917	-9.374434
H	-6.623256	2.671507	-10.975200
C	-5.836954	-4.770057	-9.470207
C	-4.887160	-4.602750	-8.444788
C	-3.998945	-5.648819	-8.191888
H	-3.262472	-5.540246	-7.400604
C	-4.029648	-6.817916	-8.942179
H	-3.324494	-7.617749	-8.733543
C	-4.954055	-6.953566	-9.967837
H	-4.961534	-7.859689	-10.570430
C	-5.873215	-5.940931	-10.254318
C	-4.848752	-3.334871	-7.617615
H	-5.217928	-2.522839	-8.256927
C	-3.440311	-2.954635	-7.180282
H	-3.029341	-3.665799	-6.455026
H	-3.447068	-1.974898	-6.691813
H	-2.766023	-2.902053	-8.039388
C	-5.786994	-3.430872	-6.414369
H	-6.827540	-3.581446	-6.718827
H	-5.747742	-2.514325	-5.816166
H	-5.504939	-4.266218	-5.762822
C	-6.820387	-6.125256	-11.428022
H	-7.557529	-5.311863	-11.421039
C	-7.585287	-7.446331	-11.354179
H	-6.913566	-8.303604	-11.470892
H	-8.328220	-7.506960	-12.155767
H	-8.106919	-7.574059	-10.400138

C	-6.058390	-6.037033	-12.751297
H	-5.550967	-5.073390	-12.854013
H	-6.736749	-6.162879	-13.602950
H	-5.296720	-6.822613	-12.814107
C	-4.278334	-2.872785	-11.821683
H	-3.861521	-3.855570	-12.048848
C	-3.902910	-1.716110	-12.671793
H	-3.706496	-1.750678	-13.739554
C	-3.988735	-0.608722	-11.903993
C	-4.393523	-1.067847	-10.533496
C	-3.924808	-0.248585	-9.365787
H	-4.353927	-0.567128	-8.411009
H	-2.830957	-0.298685	-9.273069
H	-4.191298	0.804292	-9.505418
C	-3.848492	0.814375	-12.306198
H	-3.070894	1.335785	-11.732378
H	-3.601156	0.905583	-13.367678
H	-4.780701	1.373335	-12.143504

2,3-DiMeFuran_TS-2.log
SCF (M06L) = -1549.84868004
E(SCF)+ZPE(0 K)= -1549.085556
H(298 K)= -1549.042542
G(298 K)= -1549.156169
Lowest Frequency = -429.8755cm-1

Al	-5.926889	-2.371555	-10.937197
O	-4.140954	-2.280445	-10.378525
N	-7.688616	-1.816408	-11.591031
N	-6.644746	-3.807269	-9.898911
C	-8.848319	-2.115367	-11.009651
C	-8.940551	-3.059171	-9.971106
H	-9.928143	-3.215043	-9.551475
C	-7.924692	-3.886930	-9.489071
C	-10.123156	-1.451270	-11.441163
H	-10.961146	-2.149602	-11.384940
H	-10.056506	-1.051201	-12.455321
H	-10.356438	-0.614598	-10.772957
C	-8.304127	-4.936664	-8.488004
H	-7.774267	-4.779519	-7.543567
H	-8.015405	-5.934127	-8.834097
H	-9.376906	-4.927566	-8.292714
C	-7.604491	-0.885387	-12.678260
C	-7.341395	-1.408929	-13.964097
C	-7.103144	-0.516208	-15.008394
H	-6.896285	-0.900375	-16.003554
C	-7.110584	0.858119	-14.793522
H	-6.907616	1.538970	-15.615562
C	-7.388534	1.354474	-13.529514
H	-7.406741	2.430332	-13.365297
C	-7.646028	0.502708	-12.450038
C	-7.364270	-2.906470	-14.202995
H	-6.905043	-3.381133	-13.324427
C	-6.565522	-3.344151	-15.423653
H	-7.045291	-3.036484	-16.359621
H	-6.485270	-4.435913	-15.451343
H	-5.551080	-2.931529	-15.418431
C	-8.799303	-3.428152	-14.295773
H	-9.358712	-3.252382	-13.372197
H	-8.808945	-4.506740	-14.486966
H	-9.339491	-2.938927	-15.114446
C	-7.980970	1.116653	-11.103728
H	-8.169603	0.305194	-10.390188
C	-9.243835	1.976503	-11.201788
H	-9.065556	2.860773	-11.823757
H	-9.551011	2.332076	-10.212817
H	-10.084132	1.434675	-11.646182
C	-6.831621	1.953854	-10.546008
H	-5.941681	1.344519	-10.379550
H	-7.116355	2.411599	-9.592948
H	-6.564265	2.764938	-11.233131
C	-5.731530	-4.841285	-9.489412
C	-5.055993	-4.758852	-8.259142
C	-4.189052	-5.799380	-7.913009
H	-3.657676	-5.747104	-6.964669
C	-3.986840	-6.881219	-8.756108
H	-3.310898	-7.681209	-8.466860
C	-4.634914	-6.929005	-9.985494
H	-4.450194	-7.766097	-10.652547
C	-5.507919	-5.913653	-10.381973
C	-5.200370	-3.580174	-7.316069
H	-5.960207	-2.904204	-7.729765
C	-3.885518	-2.804407	-7.217550
H	-3.108716	-3.424613	-6.754484
H	-4.010905	-1.914946	-6.590349
H	-3.536336	-2.498253	-8.206728
C	-5.650193	-4.010322	-5.919139
H	-6.581740	-4.585530	-5.926669
H	-5.802407	-3.136267	-5.278186
H	-4.890042	-4.635405	-5.437349
C	-6.176490	-5.950344	-11.748012
H	-6.146950	-4.920781	-12.139045
C	-7.649193	-6.364405	-11.691902
H	-7.762797	-7.331466	-11.188055
H	-8.049564	-6.473512	-12.705786
H	-8.276203	-5.632939	-11.176650
C	-5.448798	-6.838698	-12.751576
H	-4.375529	-6.632012	-12.792828
H	-5.861085	-6.684882	-13.754031
H	-5.575626	-7.901310	-12.515378
C	-4.490280	-2.511872	-12.335780
C	-4.068987	-1.187213	-12.732743
C	-4.248394	-0.283631	-11.720467
H	-4.074994	0.786721	-11.834256
C	-4.638872	-0.878115	-10.462489
C	-3.779952	-3.710750	-12.859049
H	-3.747310	-4.493974	-12.094592
H	-4.340633	-4.134561	-13.705725
H	-2.754748	-3.516869	-13.197568
H	-4.422970	-0.312285	-9.554639
C	-3.442411	-0.892555	-14.057267
H	-3.264930	0.180212	-14.172216
H	-2.478469	-1.401817	-14.188098
H	-4.078433	-1.205841	-14.896957

2,3-DiMeFuran_TS-2'.log
SCF (M06L) = -1549.84665657
E(SCF)+ZPE(0 K)= -1549.084157
H(298 K)= -1549.040982
G(298 K)= -1549.155378
Lowest Frequency = -502.9222cm-1

Al	-5.981011	-2.279287	-10.913429
O	-4.216617	-2.206071	-10.297712
N	-7.747494	-1.716248	-11.518398
N	-6.702821	-3.702629	-9.841496

C	-8.924812	-2.183874	-11.099276
C	-9.032785	-3.129986	-10.069412
H	-10.036992	-3.381407	-9.747242
C	-7.992505	-3.880139	-9.513409
C	-10.197154	-1.764083	-11.776221
H	-10.432890	-2.478086	-12.574268
H	-10.125680	-0.777058	-12.236608
H	-11.035265	-1.777579	-11.075999
C	-8.359123	-4.950242	-8.528165
H	-9.441162	-5.039158	-8.424784
H	-7.933329	-4.722736	-7.545487
H	-7.948483	-5.921860	-8.818101
C	-7.679741	-0.820193	-12.636786
C	-7.669733	-1.350891	-13.939865
C	-7.480225	-0.471447	-15.009308
H	-7.461171	-0.868175	-16.022771
C	-7.311191	0.888539	-14.797439
H	-7.159977	1.557508	-15.640124
C	-7.339528	1.397416	-13.503003
H	-7.208752	2.464921	-13.349243
C	-7.518211	0.562906	-12.397931
C	-7.858675	-2.830149	-14.222396
H	-8.037455	-3.345354	-13.269008
C	-6.613909	-3.452707	-14.853032
H	-6.378236	-2.975769	-15.811294
H	-6.773887	-4.519636	-15.045934
H	-5.738367	-3.347829	-14.204819
C	-9.072655	-3.071010	-15.120949
H	-9.979860	-2.603177	-14.725956
H	-9.264363	-4.143028	-15.235410
H	-8.908361	-2.660401	-16.123438
C	-7.559085	1.124489	-10.986631
H	-6.981437	0.431858	-10.358149
C	-8.982632	1.184142	-10.426276
H	-9.641727	1.757343	-11.088971
H	-8.985476	1.678725	-9.449478
H	-9.421119	0.194221	-10.286939
C	-6.923054	2.503693	-10.858828
H	-5.917437	2.543332	-11.288062
H	-6.844555	2.782142	-9.803657
H	-7.525534	3.276493	-11.350200
C	-5.757229	-4.712026	-9.442171
C	-5.104968	-4.639383	-8.199687
C	-4.214900	-5.662329	-7.862067
H	-3.703076	-5.619236	-6.902529
C	-3.965907	-6.716460	-8.728073
H	-3.273400	-7.504559	-8.445368
C	-4.587540	-6.749392	-9.970890
H	-4.365443	-7.562456	-10.656556
C	-5.482665	-5.750425	-10.359764
C	-5.298624	-3.486111	-7.235438
H	-6.052214	-2.810826	-7.662604
C	-3.996793	-2.699873	-7.072043
H	-3.229636	-3.322597	-6.596887
H	-4.151096	-1.825259	-6.430266
H	-3.616161	-2.367210	-8.041016
C	-5.794928	-3.954943	-5.867246
H	-6.720526	-4.536739	-5.925382
H	-5.977661	-3.099981	-5.208610
H	-5.047651	-4.588060	-5.375808
C	-6.130973	-5.789566	-11.735831
H	-6.268692	-4.748900	-12.062103
C	-7.516854	-6.437055	-11.720279
H	-7.467905	-7.462064	-11.334251
H	-7.926538	-6.482941	-12.736253
H	-8.230938	-5.878008	-11.109337
C	-5.254844	-6.472429	-12.781465
H	-4.234341	-6.077170	-12.782747
H	-5.674729	-6.325567	-13.781072
H	-5.191833	-7.554204	-12.619136
C	-4.490725	-2.735125	-12.151453
H	-4.017721	-3.710972	-12.304799
C	-3.885944	-1.587635	-12.744107
H	-3.266860	-1.617459	-13.637082
C	-4.032084	-0.466688	-11.966687
C	-4.587737	-0.787708	-10.658840
C	-3.615428	0.906634	-12.360868
H	-2.886542	1.339306	-11.664346
H	-3.185318	0.918917	-13.365318
H	-4.476237	1.587680	-12.373326
C	-4.318104	0.150053	-9.519356
H	-4.890178	-0.108392	-8.621907
H	-3.254232	0.150740	-9.236103
H	-4.589330	1.177120	-9.790966

4c.log

SCF (M06L) = -1549.97380031
E(SCF)+ZPE(0 K)= -1549.207829
H(298 K)= -1549.164131
G(298 K)= -1549.280139
Lowest Frequency = 24.4169cm-1

Al	-6.019674	-2.207935	-10.902808
O	-5.725331	-0.937092	-9.680912
N	-7.720742	-1.731375	-11.679401
N	-6.688518	-3.769899	-10.019643
C	-8.866358	-2.032656	-11.053103
C	-8.934283	-2.917959	-9.964118
H	-9.903713	-3.018445	-9.489973
C	-7.931539	-3.785642	-9.514830
C	-10.155482	-1.439488	-11.537410
H	-10.267803	-1.579825	-12.617174
H	-10.179276	-0.358942	-11.366992
H	-11.010653	-1.887391	-11.030572
C	-8.275609	-4.767935	-8.435369
H	-7.726639	-4.516036	-7.520265
H	-7.974786	-5.785461	-8.699625
H	-9.342915	-4.758975	-8.211259
C	-7.768971	-0.893929	-12.848309
C	-7.626010	-1.506216	-14.111746
C	-7.702665	-0.704758	-15.251413
H	-7.600861	-1.161892	-16.231658
C	-7.895261	0.668203	-15.151914
H	-7.954677	1.276677	-16.050073
C	-7.984555	1.259597	-13.901059
H	-8.096856	2.338984	-13.821634
C	-7.916955	0.500757	-12.728631
C	-7.393214	-2.999222	-14.221510
H	-6.731686	-3.271895	-13.388058
C	-6.681344	-3.409379	-15.504250
H	-7.331076	-3.312328	-16.381379
H	-6.375614	-4.458928	-15.445866
H	-5.784715	-2.808312	-15.680936
C	-8.684240	-3.801413	-14.051839
H	-9.131325	-3.664541	-13.062119
H	-8.488626	-4.873241	-14.175339

H	-9.425825	-3.510512	-14.804835
C	-7.921660	1.215154	-11.391795
H	-7.963435	0.463651	-10.594380
C	-9.108552	2.164403	-11.230793
H	-9.057806	2.989579	-11.949776
H	-9.110907	2.608626	-10.230763
H	-10.073633	1.668947	-11.378418
C	-6.614931	1.992054	-11.223034
H	-5.738791	1.352966	-11.362667
H	-6.551492	2.434400	-10.223143
H	-6.555200	2.806960	-11.954257
C	-5.807174	-4.870039	-9.740604
C	-4.970834	-4.848633	-8.611852
C	-4.119181	-5.936556	-8.398837
H	-3.462033	-5.928844	-7.531545
C	-4.093861	-7.013063	-9.273102
H	-3.426463	-7.850615	-9.089670
C	-4.916901	-7.011130	-10.393584
H	-4.882735	-7.850006	-11.083717
C	-5.780707	-5.946083	-10.653940
C	-4.928220	-3.670636	-7.661539
H	-5.743774	-2.985882	-7.923948
C	-3.616822	-2.906884	-7.842090
H	-2.763592	-3.527859	-7.543793
H	-3.603807	-1.999178	-7.229960
H	-3.465435	-2.608784	-8.884354
C	-5.110134	-4.092906	-6.205354
H	-6.032278	-4.663452	-6.051074
H	-5.143172	-3.216165	-5.551337
H	-4.279880	-4.720764	-5.863099
C	-6.677101	-5.959117	-11.878364
H	-6.829766	-4.912553	-12.176383
C	-8.059168	-6.540061	-11.568360
H	-7.970825	-7.545499	-11.140359
H	-8.658328	-6.618733	-12.482038
H	-8.622177	-5.920402	-10.865221
C	-6.064359	-6.697183	-13.064347
H	-5.043672	-6.363811	-13.275732
H	-6.669162	-6.532746	-13.962866
H	-6.034766	-7.779947	-12.899353
C	-4.359850	-2.337765	-11.876864
C	-3.398017	-1.409090	-11.543595
C	-3.547871	-0.390397	-10.522335
C	-4.605259	-0.226848	-9.683741
H	-2.713831	0.290863	-10.373024
H	-4.573980	0.556124	-8.920941
C	-4.005093	-3.386182	-12.899028
H	-3.753898	-2.955466	-13.880500
H	-3.127875	-3.978809	-12.597555
H	-4.814187	-4.103469	-13.072433
C	-2.055226	-1.439299	-12.230520
H	-1.522598	-2.375398	-12.020701
H	-2.158875	-1.392010	-13.320908
H	-1.409926	-0.614356	-11.918153

5c.log

SCF (M06L) = -1549.97954172
E(SCF)+ZPE(0 K)= -1549.214292
H(298 K)= -1549.170300
G(298 K)= -1549.287809
Lowest Frequency = 23.8827cm-1

Al	-6.050009	-2.233322	-10.919528
O	-5.587671	-0.989389	-9.729964
N	-7.749686	-1.730183	-11.649510
N	-6.734944	-3.724511	-9.932751
C	-8.908206	-2.012875	-11.040338
C	-9.003271	-2.917198	-9.971746
H	-9.986746	-3.039327	-9.533107
C	-8.004938	-3.783908	-9.508548
C	-10.174983	-1.378378	-11.529808
H	-10.292657	-1.521438	-12.608736
H	-10.154348	-0.295913	-11.368357
H	-11.046996	-1.788633	-11.019756
C	-8.398734	-4.848985	-8.530600
H	-7.745170	-4.830527	-7.652353
H	-8.281196	-5.845332	-8.969452
H	-9.433760	-4.731392	-8.208725
C	-7.761673	-0.905360	-12.826840
C	-7.639514	-1.536847	-14.082603
C	-7.681744	-0.739167	-15.227399
H	-7.597730	-1.204610	-16.205066
C	-7.814698	0.641672	-15.139353
H	-7.847377	1.243670	-16.043238
C	-7.875009	1.248291	-13.894086
H	-7.936283	2.332447	-13.823442
C	-7.841373	0.494899	-12.717223
C	-7.465250	-3.041092	-14.188113
H	-6.833456	-3.354326	-13.343265
C	-6.747309	-3.464840	-15.463785
H	-7.367801	-3.308472	-16.353139
H	-6.507067	-4.531207	-15.428238
H	-5.813224	-2.912583	-15.603586
C	-8.795474	-3.784336	-14.053946
H	-9.265630	-3.618695	-13.079574
H	-8.646879	-4.864774	-14.166132
H	-9.501004	-3.464577	-14.829724
C	-7.805626	1.213205	-11.383099
H	-7.880516	0.466833	-10.583183
C	-8.947432	2.215298	-11.219945
H	-8.868051	3.031171	-11.946903
H	-8.922500	2.667690	-10.223818
H	-9.933400	1.758741	-11.355070
C	-6.461934	1.926516	-11.219944
H	-5.618630	1.241638	-11.351188
H	-6.381605	2.377944	-10.225247
H	-6.359342	2.728210	-11.960766
C	-5.853804	-4.815932	-9.619582
C	-5.002470	-4.748047	-8.501907
C	-4.152938	-5.829348	-8.252195
H	-3.489483	-5.787278	-7.390434
C	-4.138582	-6.944487	-9.078440
H	-3.471250	-7.774838	-8.864988
C	-4.976774	-6.988041	-10.184906
H	-4.959053	-7.855404	-10.842390
C	-5.842342	-5.932858	-10.478531
C	-4.964793	-3.548016	-7.577742
H	-5.708937	-2.821941	-7.924690
C	-3.596979	-2.868237	-7.635966
H	-2.810143	-3.537438	-7.268441
H	-3.586198	-1.970260	-7.008763
H	-3.334511	-2.570461	-8.656011
C	-5.305289	-3.931729	-6.138208
H	-6.285379	-4.413600	-6.057888
H	-5.315180	-3.046650	-5.494340
H	-4.566234	-4.627946	-5.725609

C	-6.715760	-6.016054	-11.715772
H	-7.327779	-5.106730	-11.769738
C	-7.665757	-7.211479	-11.663714
H	-7.113861	-8.157842	-11.639929
H	-8.313486	-7.232321	-12.546366
H	-8.309850	-7.186441	-10.778900
C	-5.858188	-6.069253	-12.978898
H	-5.207103	-5.192351	-13.059172
H	-6.489175	-6.111526	-13.873902
H	-5.217982	-6.958482	-12.986501
C	-4.499862	-2.334117	-12.034679
H	-4.319659	-2.954588	-12.913777
C	-3.479308	-1.487174	-11.700047
H	-2.572012	-1.493390	-12.316473
C	-3.406185	-0.542503	-10.607356
C	-4.422050	-0.348402	-9.702833
C	-4.287447	0.632734	-8.578366
H	-3.413052	0.415764	-7.952601
H	-4.149748	1.656781	-8.947768
H	-5.181723	0.606860	-7.953063
C	-2.140298	0.253380	-10.439871
H	-1.435443	0.037846	-11.247818
H	-2.314115	1.337729	-10.445584
H	-1.622060	0.032284	-9.495782

2-MeOFuran.log
SCF (M06L) = -344.505550704
E(SCF)+ZPE(0 K)= -344.403180
H(298 K)= -344.395855
G(298 K)= -344.433762
Lowest Frequency = 59.8982cm-1

C	-2.892702	0.428402	0.174527
C	-1.651265	0.393146	-0.370792
C	-1.089990	1.699169	-0.233452
C	-2.051249	2.434251	0.394444
O	-3.154311	1.696217	0.655709
H	-3.688377	-0.284091	0.309959
H	-1.181543	-0.468104	-0.823508
O	-2.048832	3.718429	0.767311
C	-3.231257	4.188143	1.404002
H	-3.047329	5.237377	1.630227
H	-4.101200	4.095839	0.745843
H	-3.431222	3.637189	2.328587
H	-0.121343	2.052111	-0.548540

2-MeOFuran_Int-1.log
SCF (M06L) = -1585.75603858
E(SCF)+ZPE(0 K)= -1585.014234
H(298 K)= -1584.970341
G(298 K)= -1585.089040
Lowest Frequency = 20.7914cm-1

H	1.261459	-2.719601	-2.413452
C	1.737448	-2.016864	-3.101487
H	2.701796	-2.438762	-3.409000
H	1.103371	-1.958054	-3.992831
C	1.928199	-0.628125	-2.486415
H	0.956457	-0.310299	-2.078643
C	2.312902	0.358461	-3.585414
H	2.575921	1.343272	-3.187999
H	1.479613	0.485931	-4.285308
C	-1.668534	-2.325516	-0.185561
C	-2.928936	-3.027653	-0.606391
H	-3.683171	-3.006117	0.187319
H	-2.735167	-4.069056	-0.867462
H	-3.381308	-2.524635	-1.467387
H	-1.511281	-1.485806	-2.605105
H	-0.906790	-0.461604	-3.908452
H	-2.645467	-0.749037	-3.752154
C	-1.844900	0.659986	-2.302661
C	-1.897499	1.913076	-3.165898
H	-1.976033	2.821618	-2.561387
H	-2.740945	1.893919	-3.865319
C	-4.172977	1.275352	-1.501799
H	-4.274290	1.916283	-2.372649
C	-5.215171	1.203630	-0.585256
H	-6.124001	1.775979	-0.749158
C	-5.079130	0.421169	0.552210
H	-5.880744	0.396951	1.287120
C	-3.921831	-0.326455	0.782808
H	-5.859124	-1.228223	2.698264
C	-5.013151	-1.883242	2.464130
H	-4.824839	-2.491787	3.353941
C	-2.891748	-0.278777	-0.176975
C	-2.992748	0.553647	-1.314176
H	-0.918745	0.733591	-1.704611
H	-2.938853	-1.794885	1.977812
C	-3.767787	-1.085539	2.086853
C	-3.387051	-0.115366	3.206294
H	-2.468471	0.430489	2.962094
H	-3.230974	-0.645595	4.151877
H	-4.179686	0.625343	3.361967
C	0.798474	3.633531	0.648000
C	2.082925	3.165878	0.643965
H	3.185939	2.191780	-1.052509
C	1.128390	2.855746	-1.357472
H	0.808271	2.696512	-2.374966
Al	-0.162593	0.157258	0.751860
N	-1.676877	-1.007103	0.030606
H	-5.333734	-2.553245	1.659071
H	-0.988912	1.985393	-3.773652
C	-1.721928	-0.582652	-3.185174
C	-0.508925	-3.103730	-0.053235
H	-0.630440	-4.175860	-0.160287
C	0.804835	-2.633953	0.063918
C	1.911269	-3.651692	0.060211
H	1.523990	-4.662731	-0.075321
H	2.459881	-3.614285	1.008465
H	2.647733	-3.447817	-0.722824
H	1.872947	-1.486513	2.473069
C	2.889755	-1.074222	2.509194
C	2.827948	0.239973	3.288276
H	2.149360	0.949802	2.801246
H	2.475674	0.073960	4.311981
H	3.819661	0.704953	3.346098
C	3.781432	-2.084216	3.229026
H	3.858049	-3.029770	2.682178
H	3.390880	-2.303336	4.227694
H	4.799540	-1.699985	3.356467
C	3.341903	-0.823105	1.084880
C	2.471333	-0.944676	-0.013221
C	2.902484	-0.627644	-1.321699
C	2.287174	2.659610	-0.674454
C	4.239357	-0.273614	-1.511712
H	4.591827	-0.041709	-2.513663

C	5.118899	-0.176533	-0.438348
H	6.153357	0.111036	-0.605239
C	4.662029	-0.427713	0.847113
H	5.342006	-0.325421	1.690854
N	1.104843	-1.332489	0.167313
H	3.166429	0.001129	-4.172782
O	0.203707	3.480058	-0.548454
H	2.773572	3.149307	1.473438
C	0.543507	4.098554	2.879798
H	1.481647	4.662376	2.963555
H	-0.201391	4.529999	3.546885
H	0.725709	3.049260	3.147428
O	0.008736	4.187953	1.569424

2-MeOFuran_TS-1.log
SCF (M06L) = -1585.73392648
E(SCF)+ZPE(0 K)= -1584.993612
H(298 K)= -1584.950604
G(298 K)= -1585.066157
Lowest Frequency = -157.9448cm-1

H	-1.836538	3.207570	1.544757
C	-2.043185	3.644693	0.563575
H	-3.051187	4.074845	0.589933
H	-1.334083	4.466196	0.416868
C	-1.919957	2.613502	-0.559848
H	-0.932270	2.142922	-0.456248
C	-1.957287	3.303436	-1.918305
H	-1.917403	2.586273	-2.744610
H	-1.102305	3.980355	-2.018637
C	1.294548	0.726305	2.193461
C	2.514553	1.091631	2.989077
H	3.150815	0.212696	3.140438
H	2.246743	1.496295	3.965946
H	3.131607	1.822588	2.457513
H	1.606777	3.139799	1.279236
H	1.278863	4.393267	0.082594
H	2.949347	4.032586	0.538916
C	2.045285	2.558079	-0.786121
C	2.272778	3.261076	-2.119338
H	2.390142	2.547827	-2.941067
H	3.165621	3.896201	-2.099999
C	4.326935	1.493891	-1.093215
H	4.598178	2.325996	-1.736937
C	5.244711	0.472970	-0.874763
H	6.226828	0.515681	-1.337411
C	4.891942	-0.611184	-0.085137
H	5.597839	-1.426914	0.057452
C	3.637165	-0.686756	0.525435
H	5.272877	-2.565219	1.904968
C	4.321438	-2.283287	2.368991
H	3.991139	-3.136735	2.969348
C	2.733163	0.373399	0.324928
C	3.057363	1.460806	-0.514305
H	1.064126	2.068117	-0.836468
H	2.325046	-1.734284	1.838794
C	3.272069	-1.926223	1.318274
C	3.046619	-3.103126	0.367474
H	2.310140	-2.863790	-0.406005
H	2.698014	-3.987118	0.913741
H	3.980464	-3.370962	-0.140735
C	0.088752	-2.113087	-2.209833
C	-1.278483	-1.849199	-2.552876
H	-2.279644	0.098368	-2.750445
C	-0.078081	0.041202	-2.277215
H	0.295241	0.984266	-2.675740
Al	0.007757	-0.579772	-0.139233
N	1.428518	0.339062	0.920241
H	4.528033	-1.452202	3.051655
H	1.425755	3.913264	-2.354431
C	1.968127	3.582973	0.346302
C	0.051734	0.818925	2.839505
H	0.072667	1.116760	3.881611
C	-1.214878	0.687264	2.254194
C	-2.414367	1.001785	3.101311
H	-2.128406	1.488134	4.034960
H	-2.953451	0.079482	3.348033
H	-3.127766	1.639253	2.571077
H	-2.413073	-1.728838	1.912729
C	-3.336701	-1.903058	1.345942
C	-3.101210	-3.126873	0.461897
H	-2.244336	-2.968348	-0.200761
H	-2.903646	-4.016787	1.069445
H	-3.979080	-3.334826	-0.161793
C	-4.462717	-2.172083	2.343266
H	-4.676831	-1.299498	2.969180
H	-4.205462	-3.007201	3.002275
H	-5.393963	-2.438842	1.831573
C	-3.609484	-0.669971	0.507205
C	-2.679868	0.377900	0.385660
C	-2.949321	1.508595	-0.416979
C	-1.371239	-0.484428	-2.651983
C	-4.177746	1.577573	-1.074402
H	-4.404800	2.442689	-1.692408
C	-5.106151	0.546435	-0.969495
H	-6.052819	0.610202	-1.498870
C	-4.816792	-0.565084	-0.190803
H	-5.541441	-1.373732	-0.115730
N	-1.386096	0.290620	0.989383
H	-2.859279	3.911963	-2.047734
O	0.844781	-1.035701	-2.571351
H	-2.078112	-2.577917	-2.579311
C	0.094855	-4.362887	-1.691889
H	-0.861019	-4.592511	-2.182032
H	0.755954	-5.226037	-1.774705
H	-0.095766	-4.132737	-0.631447
O	0.760877	-3.281922	-2.309499

2-MeOFuran_Int-2.log
SCF (M06L) = -1585.75064279
E(SCF)+ZPE(0 K)= -1585.009393
H(298 K)= -1584.966303
G(298 K)= -1585.081507
Lowest Frequency = 27.2440cm-1

Al	-6.017652	-2.359867	-10.910848
O	-3.759636	-2.421742	-10.582774
N	-7.729937	-1.746483	-11.538623
N	-6.742717	-3.776865	-9.837812
C	-8.911667	-2.070873	-11.004808
C	-9.041216	-3.053840	-10.013593
H	-10.045745	-3.238781	-9.650119
C	-8.041258	-3.909056	-9.529339
C	-10.157082	-1.379090	-11.473002
H	-10.125337	-1.177011	-12.547147
H	-10.263998	-0.409084	-10.974356

H	-11.046083	-1.968667	-11.243939
C	-8.483862	-5.063344	-8.678810
H	-7.757404	-5.298600	-7.897185
H	-8.586710	-5.967312	-9.290141
H	-9.455441	-4.860760	-8.225174
C	-7.622282	-0.806913	-12.616901
C	-7.341305	-1.320506	-13.902600
C	-7.108982	-0.414688	-14.937356
H	-6.890503	-0.787062	-15.934230
C	-7.133604	0.957147	-14.709823
H	-6.938546	1.647152	-15.526074
C	-7.402194	1.441160	-13.438338
H	-7.411712	2.514827	-13.260852
C	-7.652150	0.576989	-12.367902
C	-7.308228	-2.818510	-14.146127
H	-6.849903	-3.282739	-13.260773
C	-6.457104	-3.223109	-15.342002
H	-6.900221	-2.901287	-16.291035
H	-6.364100	-4.312451	-15.386226
H	-5.448288	-2.802924	-15.281711
C	-8.718782	-3.396300	-14.270891
H	-9.307589	-3.238124	-13.362375
H	-8.680934	-4.476099	-14.453327
H	-9.258299	-2.935248	-15.106216
C	-7.920186	1.165154	-10.995619
H	-8.194744	0.348972	-10.316472
C	-9.082635	2.158351	-11.037135
H	-8.820158	3.050360	-11.616435
H	-9.342271	2.493629	-10.028148
H	-9.979697	1.729530	-11.495877
C	-6.674780	1.836705	-10.416125
H	-5.867633	1.121516	-10.237695
H	-6.908795	2.317205	-9.460479
H	-6.300802	2.613298	-11.093793
C	-5.836030	-4.841448	-9.504507
C	-4.898069	-4.643020	-8.473807
C	-4.010146	-5.681211	-8.187470
H	-3.282055	-5.550656	-7.391578
C	-4.032384	-6.868714	-8.908662
H	-3.329089	-7.662501	-8.672188
C	-4.943705	-7.031938	-9.942814
H	-4.940708	-7.951981	-10.523628
C	-5.860199	-6.027611	-10.264016
C	-4.874140	-3.355615	-7.676555
H	-5.217617	-2.546367	-8.331048
C	-3.476291	-2.964161	-7.218745
H	-3.087702	-3.639663	-6.447611
H	-3.496073	-1.957897	-6.790390
H	-2.779209	-2.959184	-8.061199
C	-5.838474	-3.426917	-6.492213
H	-6.873208	-3.578631	-6.816398
H	-5.808473	-2.499181	-5.911728
H	-5.574919	-4.251627	-5.819261
C	-6.784573	-6.219733	-11.453700
H	-7.550508	-5.433093	-11.438613
C	-7.505363	-7.565891	-11.427565
H	-6.806034	-8.399362	-11.553039
H	-8.231566	-7.629586	-12.244063
H	-8.040151	-7.731669	-10.486568
C	-6.002269	-6.061822	-12.758574
H	-5.517370	-5.081899	-12.813827
H	-6.661530	-6.169957	-13.627632
H	-5.218836	-6.824181	-12.836430
C	-4.291568	-2.712732	-11.935662
H	-3.844474	-3.628599	-12.323703
C	-4.041073	-1.435659	-12.651433
H	-3.955076	-1.326149	-13.728254
C	-4.115137	-0.450247	-11.734404
C	-4.471586	-1.108704	-10.439069
C	-2.821193	-0.029275	-9.170265
H	-2.626983	0.757502	-9.911545
H	-2.681287	0.377547	-8.166488
H	-2.113237	-0.848511	-9.338470
O	-4.166045	-0.490012	-9.244868
H	-4.110968	0.620988	-11.908375

2-MeOFuran_TS-2.log
SCF (M06L) = -1585.74307695
E(SCF)+ZPE(0 K)= -1585.003308
H(298 K)= -1584.960498
G(298 K)= -1585.075090
Lowest Frequency = -267.4650cm-1

Al	-5.947773	-2.357649	-10.956457
O	-4.070113	-2.438070	-10.568290
N	-7.711802	-1.793100	-11.567072
N	-6.643824	-3.793333	-9.901402
C	-8.872083	-2.121523	-11.002469
C	-8.956275	-3.088336	-9.987031
H	-9.943808	-3.272872	-9.579601
C	-7.927191	-3.906152	-9.511904
C	-10.146248	-1.454450	-11.429951
H	-11.002323	-2.116742	-11.287450
H	-10.109947	-1.131772	-12.473204
H	-10.322178	-0.558970	-10.822657
C	-8.300307	-4.983057	-8.536759
H	-7.783430	-4.840477	-7.582527
H	-7.991786	-5.967809	-8.901986
H	-9.375313	-4.994390	-8.354302
C	-7.643149	-0.871446	-12.662583
C	-7.412003	-1.406599	-13.948675
C	-7.209568	-0.520705	-15.006644
H	-7.031199	-0.913955	-16.003937
C	-7.220248	0.855045	-14.803518
H	-7.049127	1.530050	-15.637612
C	-7.454924	1.362095	-13.534108
H	-7.467382	2.439109	-13.377021
C	-7.674370	0.517400	-12.441569
C	-7.429505	-2.905806	-14.177641
H	-7.031501	-3.380717	-13.271058
C	-6.550532	-3.353701	-15.337964
H	-6.960657	-3.047975	-16.307220
H	-6.474851	-4.445669	-15.351570
H	-5.536780	-2.950769	-15.253879
C	-8.862014	-3.411769	-14.355834
H	-9.470500	-3.231615	-13.463794
H	-8.874070	-4.489401	-14.550402
H	-9.350761	-2.913788	-15.201262
C	-7.944907	1.132078	-11.081680
H	-8.150857	0.321649	-10.371719
C	-9.168361	2.049369	-11.128217
H	-8.975271	2.931029	-11.749334
H	-9.424010	2.408669	-10.126172
H	-10.047197	1.547893	-11.545593
C	-6.734327	1.900021	-10.554489
H	-5.875581	1.238080	-10.423930

H	-6.960292	2.363647	-9.588561
H	-6.444914	2.699958	-11.245909
C	-5.720847	-4.813084	-9.473827
C	-5.099364	-4.732907	-8.216451
C	-4.245637	-5.771094	-7.834984
H	-3.756279	-5.721651	-6.864135
C	-4.002511	-6.847786	-8.674968
H	-3.340653	-7.649438	-8.358615
C	-4.586187	-6.884026	-9.936155
H	-4.363170	-7.711423	-10.603963
C	-5.442118	-5.868185	-10.366787
C	-5.266443	-3.537309	-7.300285
H	-6.040178	-2.885957	-7.726907
C	-3.961340	-2.740378	-7.244342
H	-3.177153	-3.326713	-6.750951
H	-4.092767	-1.816329	-6.670696
H	-3.609413	-2.491398	-8.248960
C	-5.703076	-3.930796	-5.890061
H	-6.632334	-4.510091	-5.881157
H	-5.858631	-3.040933	-5.271989
H	-4.938340	-4.538672	-5.393989
C	-6.034675	-5.887093	-11.764884
H	-6.030564	-4.848727	-12.123130
C	-7.486111	-6.369358	-11.797226
H	-7.581404	-7.363678	-11.344840
H	-7.837102	-6.443538	-12.832773
H	-8.166062	-5.689493	-11.275287
C	-5.197637	-6.689765	-12.752909
H	-4.141128	-6.415943	-12.697737
H	-5.540914	-6.501677	-13.774788
H	-5.280457	-7.768805	-12.577707
C	-4.532705	-2.513853	-12.393664
C	-3.965479	-1.205823	-12.729949
C	-4.071297	-0.348397	-11.688715
H	-3.878839	0.720375	-11.744794
C	-4.532656	-1.017296	-10.474681
H	-4.254964	-0.565194	-9.520445
H	-3.648096	-0.953384	-13.737032
C	-2.693822	-3.635383	-13.354515
H	-2.453253	-4.661271	-13.636498
H	-2.532073	-2.973846	-14.212404
H	-2.067767	-3.310543	-12.519248
O	-4.071965	-3.637509	-12.957135

2-MeOFuran_TS-2'.log
SCF (M06L) = -1585.72686410
E(SCF)+ZPE(0 K)= -1584.988241
H(298 K)= -1584.945338
G(298 K)= -1585.060089
Lowest Frequency = -528.7853cm-1

Al	-5.998882	-2.321722	-10.902139
O	-4.218633	-2.221957	-10.329709
N	-7.753392	-1.760494	-11.505471
N	-6.694550	-3.768353	-9.867462
C	-8.927974	-2.196888	-11.052648
C	-9.023893	-3.163194	-10.038359
H	-10.023120	-3.402741	-9.692460
C	-7.982828	-3.937319	-9.520587
C	-10.210501	-1.726352	-11.672859
H	-10.527444	-2.443669	-12.439015
H	-10.112176	-0.755124	-12.160578
H	-11.009182	-1.680813	-10.929057
C	-8.340704	-5.022865	-8.550101
H	-9.421585	-5.109009	-8.434149
H	-7.900778	-4.816430	-7.569008
H	-7.937745	-5.990152	-8.864960
C	-7.684151	-0.860260	-12.620839
C	-7.710566	-1.388252	-13.925118
C	-7.539487	-0.508424	-14.996634
H	-7.555905	-0.901869	-16.011517
C	-7.342761	0.849022	-14.785190
H	-7.208981	1.518662	-15.630408
C	-7.313784	1.350398	-13.489038
H	-7.153914	2.413638	-13.330083
C	-7.474563	0.514234	-12.382247
C	-7.907161	-2.866911	-14.203834
H	-8.045462	-3.385539	-13.245603
C	-6.681015	-3.476890	-14.882052
H	-6.484721	-2.996620	-15.847399
H	-6.834138	-4.545463	-15.070742
H	-5.783785	-3.362526	-14.264998
C	-9.153192	-3.112773	-15.054995
H	-10.048625	-2.662927	-14.614889
H	-9.336275	-4.185541	-15.176802
H	-9.036265	-2.685126	-16.057094
C	-7.460717	1.079945	-10.974216
H	-7.089399	0.291199	-10.309583
C	-8.870369	1.455362	-10.513184
H	-9.319796	2.194814	-11.186655
H	-8.843727	1.892120	-9.509593
H	-9.537851	0.589834	-10.476357
C	-6.524200	2.271458	-10.814034
H	-5.523048	2.050442	-11.194157
H	-6.424314	2.528359	-9.755863
H	-6.897740	3.161814	-11.332913
C	-5.739390	-4.767000	-9.464508
C	-5.115186	-4.699249	-8.207034
C	-4.204645	-5.704307	-7.870368
H	-3.711817	-5.664682	-6.900856
C	-3.909871	-6.734758	-8.750690
H	-3.201332	-7.508698	-8.468578
C	-4.505585	-6.761968	-10.006382
H	-4.247793	-7.556000	-10.701664
C	-5.419383	-5.779917	-10.394602
C	-5.352401	-3.562031	-7.233110
H	-6.136962	-2.915820	-7.647904
C	-4.085360	-2.718357	-7.080806
H	-3.290447	-3.305196	-6.604974
H	-4.277061	-1.843914	-6.449460
H	-3.727226	-2.374798	-8.053624
C	-5.814277	-4.058251	-5.862801
H	-6.708626	-4.687827	-5.915999
H	-6.037548	-3.214186	-5.202852
H	-5.032388	-4.651341	-5.375362
C	-6.045083	-5.808368	-11.780838
H	-6.174647	-4.765238	-12.104762
C	-7.435061	-6.447452	-11.783747
H	-7.394313	-7.475113	-11.404113
H	-7.837493	-6.484702	-12.802799
H	-8.150442	-5.888902	-11.173667
C	-5.158853	-6.489014	-12.818627
H	-4.136484	-6.099218	-12.802968
H	-5.564626	-6.334154	-13.822879
H	-5.103742	-7.571971	-12.661509
C	-4.532048	-2.587994	-12.244409

H	-4.023002	-3.519846	-12.512508
C	-3.986552	-1.358587	-12.724799
H	-3.457934	-1.264116	-13.668670
C	-4.135096	-0.343798	-11.821020
C	-4.663432	-0.806532	-10.555014
H	-3.889129	0.699884	-12.007039
C	-3.164721	0.345071	-9.159744
H	-2.508862	-0.534087	-9.154445
H	-2.790635	1.061924	-9.904252
H	-3.148439	0.815482	-8.174246
O	-4.512866	-0.028469	-9.422221

4d.log

SCF (M06L) = -1585.86136093
E(SCF)+ZPE(0 K)= -1585.118256
H(298 K)= -1585.075317
G(298 K)= -1585.189886
Lowest Frequency = 22.7247cm-1

Al	-6.088254	-2.224570	-11.006991
O	-5.544697	-0.960805	-9.890349
N	-7.804338	-1.714618	-11.677745
N	-6.709433	-3.735433	-10.025453
C	-8.938110	-2.039021	-11.039832
C	-8.985415	-2.960386	-9.982410
H	-9.953034	-3.100503	-9.514905
C	-7.957703	-3.805369	-9.542692
C	-10.231613	-1.421156	-11.477479
H	-10.372600	-1.531796	-12.557276
H	-10.234449	-0.344622	-11.280002
H	-11.079606	-1.870559	-10.960105
C	-8.286477	-4.831122	-8.500440
H	-7.766948	-4.593476	-7.565232
H	-7.943507	-5.827311	-8.794338
H	-9.357857	-4.867212	-8.300469
C	-7.873833	-0.840398	-12.819521
C	-7.789055	-1.417080	-14.104248
C	-7.892045	-0.576115	-15.213474
H	-7.836257	-1.003136	-16.210987
C	-8.051508	0.796577	-15.063248
H	-8.132428	1.434578	-15.939051
C	-8.078487	1.350379	-13.792119
H	-8.162337	2.428907	-13.674389
C	-7.983191	0.552041	-12.648420
C	-7.585704	-2.909447	-14.271833
H	-6.920036	-3.232809	-13.463111
C	-6.880030	-3.275143	-15.571002
H	-7.526446	-3.140748	-16.446024
H	-6.579871	-4.327630	-15.549271
H	-5.978337	-2.673872	-15.718586
C	-8.891639	-3.691495	-14.129202
H	-9.322514	-3.596999	-13.127063
H	-8.720702	-4.758839	-14.310165
H	-9.638350	-3.347555	-14.854656
C	-7.916830	1.218376	-11.288829
H	-7.933119	0.438695	-10.518047
C	-9.083444	2.173260	-11.039478
H	-9.062716	3.016916	-11.738290
H	-9.028613	2.590198	-10.029194
H	-10.059648	1.689496	-11.148262
C	-6.592659	1.972195	-11.152097
H	-5.734334	1.329443	-11.365141
H	-6.472728	2.366495	-10.137480
H	-6.558967	2.819210	-11.847640
C	-5.776629	-4.787818	-9.722843
C	-5.002083	-4.747322	-8.551070
C	-4.111143	-5.798561	-8.314223
H	-3.502755	-5.779521	-7.412146
C	-3.983911	-6.850245	-9.209565
H	-3.287917	-7.659391	-9.005683
C	-4.736253	-6.857050	-10.378730
H	-4.617104	-7.671779	-11.088029
C	-5.636322	-5.829474	-10.664157
C	-5.058432	-3.586114	-7.580361
H	-5.871818	-2.918184	-7.888833
C	-3.757742	-2.785246	-7.648227
H	-2.914447	-3.387103	-7.289066
H	-3.820146	-1.888412	-7.022880
H	-3.529817	-2.463903	-8.668425
C	-5.325448	-4.040057	-6.146270
H	-6.237580	-4.639989	-6.060169
H	-5.426632	-3.176965	-5.481078
H	-4.500678	-4.650165	-5.761360
C	-6.454751	-5.846449	-11.940956
H	-6.624438	-4.801198	-12.231282
C	-7.825038	-6.491425	-11.721506
H	-7.717696	-7.504739	-11.316649
H	-8.373766	-6.568188	-12.666176
H	-8.448573	-5.916412	-11.030643
C	-5.735279	-6.520173	-13.102757
H	-4.721385	-6.130829	-13.222893
H	-6.281174	-6.341636	-14.035417
H	-5.673133	-7.606815	-12.973542
C	-4.544060	-2.445655	-12.177357
C	-3.475921	-1.624310	-11.949605
C	-3.405471	-0.617927	-10.913808
C	-4.361797	-0.349050	-9.990152
H	-2.496619	-0.026409	-10.855105
H	-4.198847	0.424263	-9.236684
C	-3.395978	-3.530606	-13.967844
H	-2.511417	-3.790728	-13.369034
H	-3.595300	-4.328974	-14.686140
H	-3.182102	-2.599258	-14.511810
O	-4.550158	-3.412541	-13.167336
H	-2.581054	-1.709161	-12.567443

5d.log

SCF (M06L) = -1585.87577714
E(SCF)+ZPE(0 K)= -1585.132474
H(298 K)= -1585.089524
G(298 K)= -1585.204344
Lowest Frequency = 25.4217cm-1

Al	-6.040640	-2.321915	-10.883709
O	-5.588493	-1.107941	-9.638729
N	-7.721401	-1.761918	-11.606220
N	-6.757415	-3.813083	-9.926862
C	-8.889208	-2.033251	-11.009615
C	-9.009648	-2.960546	-9.963794
H	-9.998106	-3.072638	-9.533891
C	-8.031494	-3.855741	-9.512302
C	-10.138409	-1.359159	-11.490579
H	-10.256741	-1.481441	-12.572059
H	-10.090289	-0.280301	-11.311630
H	-11.021592	-1.755811	-10.989124
C	-8.451251	-4.931718	-8.557740

H	-7.810225	-4.934312	-7.670013
H	-8.337896	-5.921993	-9.011239
H	-9.489267	-4.807290	-8.248491
C	-7.709944	-0.908398	-12.763227
C	-7.598825	-1.512245	-14.033196
C	-7.625633	-0.686596	-15.158455
H	-7.550050	-1.129834	-16.147145
C	-7.732020	0.694151	-15.036519
H	-7.753856	1.318284	-15.925628
C	-7.777353	1.272035	-13.776875
H	-7.814291	2.355259	-13.679918
C	-7.757691	0.490013	-12.618521
C	-7.447130	-3.016333	-14.172060
H	-6.820633	-3.356933	-13.333607
C	-6.732101	-3.422365	-15.454985
H	-7.346890	-3.235574	-16.342422
H	-6.509567	-4.493175	-15.442989
H	-5.788484	-2.882728	-15.578882
C	-8.786945	-3.744896	-14.055430
H	-9.257662	-3.591466	-13.079376
H	-8.651275	-4.824860	-14.187260
H	-9.486259	-3.401943	-14.826902
C	-7.692821	1.172520	-11.266774
H	-7.780555	0.408662	-10.485272
C	-8.805553	2.199440	-11.065387
H	-8.718067	3.026828	-11.778399
H	-8.751723	2.631355	-10.061431
H	-9.805014	1.770031	-11.190906
C	-6.327324	1.843754	-11.099582
H	-5.506051	1.148961	-11.300007
H	-6.203476	2.227239	-10.081608
H	-6.225621	2.685098	-11.795468
C	-5.893989	-4.919454	-9.614770
C	-5.068125	-4.878404	-8.476939
C	-4.229330	-5.968741	-8.231058
H	-3.583072	-5.946725	-7.355628
C	-4.203203	-7.067050	-9.079626
H	-3.543927	-7.904696	-8.869517
C	-5.019093	-7.085194	-10.203458
H	-4.992122	-7.939765	-10.877175
C	-5.873247	-6.019745	-10.493907
C	-5.041776	-3.691943	-7.535575
H	-5.793294	-2.968740	-7.871238
C	-3.681323	-2.997067	-7.590179
H	-2.892224	-3.652187	-7.202067
H	-3.689780	-2.084349	-6.985148
H	-3.409205	-2.718612	-8.613133
C	-5.376902	-4.095021	-6.100512
H	-6.351838	-4.588270	-6.024971
H	-5.396187	-3.216367	-5.448386
H	-4.630317	-4.787078	-5.694516
C	-6.723190	-6.071769	-11.748903
H	-7.342254	-5.166221	-11.785491
C	-7.664003	-7.275338	-11.751813
H	-7.105652	-8.218146	-11.752541
H	-8.298569	-7.270427	-12.644190
H	-8.320535	-7.285174	-10.875919
C	-5.841607	-6.077198	-12.996584
H	-5.196009	-5.193351	-13.035600
H	-6.454932	-6.095037	-13.904660
H	-5.194026	-6.960877	-13.019488
C	-4.472408	-2.387659	-11.974179
H	-4.278617	-2.985413	-12.865180
C	-3.463262	-1.538533	-11.605743
H	-2.549310	-1.518814	-12.208304
C	-3.420892	-0.625994	-10.493825
C	-4.449764	-0.471972	-9.597523
H	-2.520860	-0.039343	-10.361506
C	-3.259884	1.149557	-8.360757
H	-3.083514	1.804401	-9.224310
H	-3.426812	1.757543	-7.471250
H	-2.374056	0.518205	-8.211828
O	-4.425092	0.371657	-8.534193

3-MeFuran.log

SCF (M06L) = -269.310389012
E(SCF)+ZPE(0 K)= -269.212624
H(298 K)= -269.206295
G(298 K)= -269.241305
Lowest Frequency = 120.0364cm-1

C	-2.965099	0.398976	-0.091229
C	-1.607052	0.387866	-0.130286
C	-1.183163	1.752993	-0.042532
C	-2.332645	2.479435	0.043000
O	-3.429802	1.671530	0.014945
H	-3.720884	-0.369645	-0.125851
H	-0.974340	-0.485000	-0.211930
C	0.213114	2.268018	-0.044209
H	0.745099	1.992319	-0.960861
H	0.237728	3.357616	0.034745
H	0.794372	1.864191	0.791463
H	-2.534961	3.536206	0.125950

3-MeFuran_Int-1.log

SCF (M06L) = -1510.56106958
E(SCF)+ZPE(0 K)= -1509.824550
H(298 K)= -1509.781067
G(298 K)= -1509.900756
Lowest Frequency = 14.2247cm-1

H	-1.263677	2.865525	1.520989
C	-1.533798	3.378917	0.594254
H	-2.521847	3.833269	0.733633
H	-0.813338	4.192211	0.449044
C	-1.535182	2.443086	-0.615769
H	-0.539882	1.966973	-0.659129
C	-1.693111	3.249570	-1.897757
H	-1.697413	2.604413	-2.779551
H	-0.866041	3.959014	-1.997980
C	1.469266	0.482701	2.600002
C	2.599247	0.815690	3.530652
H	3.172301	1.670530	3.156467
H	3.309910	-0.015446	3.596870
H	2.237493	1.048869	4.532980
H	3.315733	3.793772	1.752817
H	2.328113	4.745792	0.634564
H	3.964007	4.265105	0.180142
C	2.497177	2.654124	0.076424
C	2.132653	2.881790	-1.389990
H	1.668345	1.990955	-1.830413
H	3.021131	3.119849	-1.985830
C	4.747906	1.591793	-0.300178
H	5.041827	2.511095	-0.803517
C	5.652856	0.544213	-0.195147
H	6.651280	0.640657	-0.612562

C	5.274250	-0.629640	0.444419
H	5.982137	-1.452190	0.521806
C	3.996383	-0.780197	0.986828
H	5.586719	-2.848892	2.144842
C	4.648707	-2.579241	2.641937
H	4.293361	-3.473674	3.162881
C	3.088936	0.289504	0.866489
C	3.456929	1.487922	0.223404
H	1.571806	2.404713	0.612526
H	2.669745	-1.918386	2.196747
C	3.602738	-2.087539	1.644637
C	3.322885	-3.152461	0.584127
H	2.540899	-2.826828	-0.111422
H	3.002896	-4.093394	1.044051
H	4.223617	-3.356245	-0.006146
C	-2.125531	-0.907939	-3.257870
C	-3.310363	-1.454914	-3.650771
H	-4.896341	-0.535633	-4.963063
C	-3.090034	0.601004	-4.505711
H	-3.117704	1.565636	-4.989259
Al	0.469672	-0.438659	-0.128516
N	1.738781	0.151695	1.331053
H	4.885535	-1.821274	3.396020
H	1.429837	3.716474	-1.491981
C	3.057766	3.931879	0.698074
C	0.166475	0.561039	3.103589
H	0.068549	0.808142	4.154689
C	-1.023832	0.473431	2.369413
C	-2.306445	0.769520	3.094094
H	-2.116150	1.136343	4.103796
H	-2.931384	-0.127131	3.166827
H	-2.902115	1.510766	2.552171
H	-2.059195	-1.878176	1.937610
C	-2.907866	-2.093867	1.277546
C	-2.477847	-3.219291	0.336138
H	-1.627446	-2.914831	-0.286324
H	-2.189772	-4.116492	0.894093
H	-3.300110	-3.490564	-0.336118
C	-4.075175	-2.543956	2.152107
H	-4.430870	-1.743590	2.809613
H	-3.782732	-3.391198	2.780057
H	-4.928745	-2.871391	1.548924
C	-3.217630	-0.844399	0.476278
C	-2.302156	0.219638	0.372863
C	-2.551798	1.322429	-0.474530
C	-3.937484	-0.460864	-4.467993
C	-3.757350	1.350950	-1.177715
H	-3.965979	2.179179	-1.848568
C	-4.682893	0.320783	-1.064935
H	-5.606004	0.355996	-1.637545
C	-4.408581	-0.767413	-0.249389
H	-5.122575	-1.586358	-0.184370
N	-1.047757	0.167075	1.067456
H	-2.617389	3.838775	-1.895103
O	-1.973562	0.344467	-3.774346
C	-3.849046	-2.791621	-3.283150
H	-4.174113	-3.356925	-4.162830
H	-3.099287	-3.392303	-2.761824
H	-4.718795	-2.705377	-2.619361
H	-1.313194	-1.249243	-2.628363

3-MeFuran_TS-1.log

SCF (M06L) = -1510.53654361

E(SCF)+ZPE(0 K)= -1509.801378

H(298 K)= -1509.759019

G(298 K)= -1509.873461

Lowest Frequency = -168.7526cm-1

H	-1.913390	3.018680	1.850847
C	-2.130887	3.514793	0.901729
H	-3.148410	3.919458	0.955949
H	-1.440584	4.360421	0.812055
C	-1.983956	2.570956	-0.293918
H	-0.985927	2.114612	-0.222250
C	-2.037377	3.374321	-1.589445
H	-2.000482	2.734562	-2.477333
H	-1.191986	4.068731	-1.637217
C	1.258745	0.684549	2.161293
C	2.479477	1.155142	2.899667
H	3.138044	0.312028	3.134977
H	2.211226	1.648803	3.834618
H	3.072786	1.842580	2.289544
H	1.568196	3.083308	1.034320
H	1.096995	4.206728	-0.242112
H	2.811040	3.951880	0.114365
C	1.877885	2.324607	-0.994659
C	2.003130	2.888797	-2.403927
H	2.061514	2.088815	-3.147413
H	2.887427	3.526454	-2.515893
C	4.167292	1.283556	-1.317347
H	4.385628	2.055039	-2.050507
C	5.114493	0.298591	-1.057865
H	6.068820	0.310333	-1.577308
C	4.828480	-0.707258	-0.147387
H	5.560931	-1.490585	0.038017
C	3.609951	-0.743321	0.538838
H	5.395156	-2.385492	2.036757
C	4.464573	-2.063985	2.516959
H	4.201634	-2.829192	3.253965
C	2.671195	0.273339	0.278778
C	2.931928	1.283749	-0.671239
H	0.909294	1.811177	-0.940069
H	2.414842	-1.676834	2.041925
C	3.341467	-1.891482	1.494369
C	3.126534	-3.196733	0.726854
H	2.272160	-3.118330	0.046493
H	2.940932	-4.028144	1.415267
H	4.013975	-3.447192	0.133395
C	1.045071	-1.715651	-2.239298
C	-0.119296	-2.273055	-2.830641
H	-2.101566	-1.341599	-3.105787
C	-0.459134	-0.133366	-2.188214
H	-0.727235	0.904248	-2.391855
Al	-0.031565	-0.781215	-0.070610
N	1.379790	0.248000	0.901074
H	4.684648	-1.136871	3.056508
H	1.133019	3.509944	-2.640533
C	1.839124	3.450352	0.039121
C	0.037827	0.727724	2.855846
H	0.085732	1.043960	3.891793
C	-1.240595	0.527515	2.318705
C	-2.434198	0.761246	3.199370
H	-2.154545	1.224931	4.146734
H	-2.924006	-0.195840	3.415944
H	-3.186830	1.383265	2.706881
H	-2.332396	-2.049373	1.641393

C	-3.242726	-2.185270	1.042772
C	-2.928681	-3.212706	-0.046776
H	-2.118990	-2.866359	-0.700447
H	-2.623921	-4.170064	0.389224
H	-3.809390	-3.393730	-0.674901
C	-4.343068	-2.694538	1.970223
H	-4.591046	-1.965613	2.748507
H	-4.036754	-3.623298	2.461554
H	-5.265514	-2.909761	1.419627
C	-3.588211	-0.852097	0.412390
C	-2.699220	0.236461	0.439268
C	-2.987927	1.432715	-0.256681
C	-1.051049	-1.256728	-2.850203
C	-4.210552	1.532408	-0.922160
H	-4.452413	2.446768	-1.458150
C	-5.115282	0.475968	-0.928109
H	-6.060435	0.571471	-1.455477
C	-4.796019	-0.706662	-0.276535
H	-5.491191	-1.543550	-0.306136
N	-1.417214	0.124292	1.058790
H	-2.948037	3.979938	-1.657923
O	0.954573	-0.337753	-2.287180
C	-0.316057	-3.712132	-3.152753
H	0.440602	-4.094679	-3.846194
H	-0.254506	-4.326935	-2.245354
H	-1.298717	-3.887535	-3.597841
H	2.061779	-2.096217	-2.257798

3-MeFuran_Int-2.log
SCF (M06L) = -1510.55160754
E(SCF)+ZPE(0 K)= -1509.813813
H(298 K)= -1509.772179
G(298 K)= -1509.883368
Lowest Frequency = 27.8652cm-1

Al	-6.043658	-2.401170	-11.019676
O	-3.858645	-2.523447	-10.562985
N	-7.769330	-1.770966	-11.627797
N	-6.781636	-3.750189	-9.859034
C	-8.954586	-2.106790	-11.106389
C	-9.087119	-3.072107	-10.099225
H	-10.095631	-3.266008	-9.751603
C	-8.080900	-3.885274	-9.559059
C	-10.205913	-1.445428	-11.605463
H	-10.165076	-1.272161	-12.684318
H	-10.339818	-0.464332	-11.136542
H	-11.086171	-2.046003	-11.371091
C	-8.517675	-4.998372	-8.651984
H	-7.799240	-5.174612	-7.847645
H	-8.593751	-5.938514	-9.210395
H	-9.499716	-4.788851	-8.224806
C	-7.670019	-0.770928	-12.650420
C	-7.334931	-1.193430	-13.955432
C	-7.130912	-0.218801	-14.932390
H	-6.869004	-0.522678	-15.941961
C	-7.248059	1.134093	-14.632989
H	-7.079482	1.878749	-15.405921
C	-7.572294	1.530691	-13.343644
H	-7.647559	2.590672	-13.109219
C	-7.784246	0.594858	-12.326556
C	-7.228828	-2.671869	-14.280971
H	-6.758007	-3.163639	-13.416860
C	-6.348989	-2.971674	-15.486352
H	-6.806032	-2.634489	-16.423691
H	-6.191880	-4.050853	-15.576893
H	-5.368315	-2.494706	-15.398014
C	-8.612194	-3.300548	-14.454791
H	-9.216435	-3.217217	-13.546413
H	-8.527003	-4.365401	-14.696623
H	-9.161865	-2.816075	-15.270155
C	-8.080007	1.084899	-10.920744
H	-8.378014	0.225343	-10.309172
C	-9.224920	2.096821	-10.897375
H	-8.944002	3.029762	-11.397634
H	-9.493703	2.352731	-9.867830
H	-10.121953	1.719376	-11.399350
C	-6.831160	1.680758	-10.271311
H	-6.025092	0.943601	-10.210827
H	-7.050532	2.031759	-9.257464
H	-6.460906	2.537114	-10.847249
C	-5.868227	-4.789379	-9.469639
C	-4.940710	-4.538824	-8.441364
C	-4.035425	-5.548409	-8.112319
H	-3.315950	-5.376537	-7.316051
C	-4.027621	-6.758722	-8.794429
H	-3.308554	-7.528868	-8.529177
C	-4.932017	-6.976534	-9.824716
H	-4.909262	-7.917126	-10.370954
C	-5.869859	-6.004667	-10.182956
C	-4.938601	-3.225148	-7.686170
H	-5.384495	-2.466524	-8.341627
C	-3.534881	-2.743609	-7.339680
H	-3.055329	-3.381273	-6.588521
H	-3.577030	-1.734500	-6.917093
H	-2.902112	-2.718000	-8.230391
C	-5.810882	-3.316493	-6.433589
H	-6.852000	-3.553157	-6.676636
H	-5.806548	-2.369562	-5.883648
H	-5.442144	-4.096121	-5.756818
C	-6.804565	-6.270256	-11.351577
H	-7.597055	-5.510761	-11.350613
C	-7.479338	-7.637437	-11.256404
H	-6.756778	-8.452418	-11.371094
H	-8.224635	-7.755320	-12.049108
H	-7.982210	-7.784468	-10.294739
C	-6.060462	-6.135101	-12.681048
H	-5.635501	-5.133152	-12.796174
H	-6.731854	-6.324286	-13.526271
H	-5.235972	-6.854731	-12.741033
C	-4.294878	-2.920853	-11.928308
H	-3.865945	-3.893538	-12.176736
C	-3.894883	-1.734934	-12.728608
H	-3.639830	-1.738338	-13.784142
C	-4.013944	-0.651324	-11.929631
C	-4.472346	-1.170884	-10.605857
C	-3.867155	0.783650	-12.284250
H	-3.218125	1.321994	-11.581787
H	-3.452947	0.903090	-13.289549
H	-4.835579	1.302227	-12.274698
H	-4.192297	-0.607847	-9.711390

3-MeFuran_TS-2.log
SCF (M06L) = -1510.52898571
E(SCF)+ZPE(0 K)= -1509.793948
H(298 K)= -1509.752308
G(298 K)= -1509.863242

Lowest Frequency = -517.4352cm-1

Al	-5.956420	-2.382979	-10.948020
O	-4.142668	-2.395238	-10.506770
N	-7.719862	-1.761217	-11.497428
N	-6.667656	-3.822899	-9.909599
C	-8.881197	-2.090747	-10.936326
C	-8.972413	-3.092859	-9.956320
H	-9.958959	-3.277515	-9.546488
C	-7.949473	-3.935861	-9.514048
C	-10.145961	-1.386767	-11.329163
H	-11.013661	-2.037725	-11.206259
H	-10.110349	-1.023188	-12.358957
H	-10.300030	-0.512552	-10.685806
C	-8.325194	-5.034353	-8.564464
H	-7.841723	-4.890690	-7.592778
H	-7.982684	-6.007063	-8.931096
H	-9.404346	-5.071739	-8.412451
C	-7.645493	-0.857078	-12.607621
C	-7.456331	-1.425776	-13.886176
C	-7.235815	-0.570185	-14.965111
H	-7.092312	-0.989843	-15.958012
C	-7.185832	0.808600	-14.788391
H	-6.997602	1.459955	-15.637396
C	-7.388872	1.349003	-13.527198
H	-7.362972	2.428861	-13.392777
C	-7.627717	0.535250	-12.415038
C	-7.555342	-2.925634	-14.090286
H	-7.297210	-3.407103	-13.138367
C	-6.589123	-3.461302	-15.138736
H	-6.830081	-3.106269	-16.146823
H	-6.631507	-4.555241	-15.168716
H	-5.556681	-3.169901	-14.917230
C	-8.994503	-3.328490	-14.415878
H	-9.684424	-3.043409	-13.614903
H	-9.076952	-4.411788	-14.554551
H	-9.338312	-2.844931	-15.337487
C	-7.881105	1.184645	-11.068551
H	-8.071646	0.390236	-10.336053
C	-9.113336	2.090185	-11.127851
H	-8.934466	2.950941	-11.781858
H	-9.358263	2.481613	-10.135126
H	-9.993800	1.568796	-11.515963
C	-6.674432	1.981127	-10.576565
H	-5.802843	1.337251	-10.443813
H	-6.894186	2.462928	-9.618199
H	-6.406915	2.770785	-11.288185
C	-5.734455	-4.834394	-9.485958
C	-5.143422	-4.770036	-8.211936
C	-4.257039	-5.785214	-7.843898
H	-3.788517	-5.746002	-6.862576
C	-3.954548	-6.825696	-8.709661
H	-3.265841	-7.608489	-8.403983
C	-4.515688	-6.850450	-9.980996
H	-4.249951	-7.651452	-10.664841
C	-5.402694	-5.856455	-10.399787
C	-5.379014	-3.612985	-7.261261
H	-6.192824	-2.997692	-7.665910
C	-4.126259	-2.737639	-7.183614
H	-3.303631	-3.290843	-6.715359
H	-4.311801	-1.844670	-6.576768
H	-3.796811	-2.432513	-8.180349
C	-5.787030	-4.073095	-5.862532
H	-6.668117	-4.723503	-5.871304
H	-6.012464	-3.212630	-5.224870
H	-4.978956	-4.630648	-5.376516
C	-5.983396	-5.872155	-11.803835
H	-6.018426	-4.828765	-12.147518
C	-7.419299	-6.398463	-11.845951
H	-7.481358	-7.406624	-11.419774
H	-7.775027	-6.454166	-12.881160
H	-8.114289	-5.753396	-11.299905
C	-5.116711	-6.634439	-12.799521
H	-4.071258	-6.314486	-12.757812
H	-5.480188	-6.472141	-13.818771
H	-5.142716	-7.715347	-12.621153
C	-4.623685	-2.555548	-12.427438
C	-4.111423	-1.285117	-12.855687
C	-4.177895	-0.374362	-11.831797
H	-3.914342	0.677946	-11.939055
C	-4.545818	-0.954660	-10.563372
H	-4.240284	-0.415535	-9.665436
C	-3.510862	-1.044834	-14.203243
H	-3.113053	-0.029684	-14.286191
H	-2.690934	-1.744005	-14.411922
H	-4.245222	-1.169968	-15.010755
H	-4.153815	-3.456142	-12.842270

3-MeFuran_TS-2’.log

SCF (M06L) = -1510.53228965
E(SCF)+ZPE(0 K)= -1509.797414
H(298 K)= -1509.755742
G(298 K)= -1509.867181
Lowest Frequency = -504.6466cm-1

Al	-5.984146	-2.375795	-10.995151
O	-4.176746	-2.347541	-10.499063
N	-7.760147	-1.787381	-11.538847
N	-6.678222	-3.805717	-9.923521
C	-8.919495	-2.142172	-10.986639
C	-8.997596	-3.134030	-9.996679
H	-9.983289	-3.336331	-9.593172
C	-7.956899	-3.941824	-9.529848
C	-10.197481	-1.474113	-11.400213
H	-11.048541	-2.147012	-11.279263
H	-10.160805	-1.121335	-12.433646
H	-10.383103	-0.597388	-10.768854
C	-8.309501	-5.025125	-8.554272
H	-9.387707	-5.082950	-8.401569
H	-7.830000	-4.843985	-7.586861
H	-7.944842	-5.999256	-8.894317
C	-7.698308	-0.856886	-12.627536
C	-7.474666	-1.385258	-13.916418
C	-7.266473	-0.494567	-14.969454
H	-7.093758	-0.884055	-15.969669
C	-7.265577	0.879335	-14.757224
H	-7.087653	1.558524	-15.586398
C	-7.504072	1.380992	-13.485042
H	-7.514998	2.457597	-13.322797
C	-7.729150	0.530954	-12.398024
C	-7.521082	-2.881270	-14.162289
H	-7.273194	-3.381189	-13.217068
C	-6.510697	-3.356206	-15.198474
H	-6.752319	-3.000008	-16.206105
H	-6.499836	-4.450478	-15.241672
H	-5.500099	-3.014589	-14.952631

C	-8.938563	-3.316664	-14.537431
H	-9.656741	-3.081261	-13.745055
H	-8.983289	-4.396184	-14.716488
H	-9.274650	-2.811876	-15.450516
C	-8.021582	1.132580	-11.036747
H	-8.221699	0.312297	-10.336525
C	-9.260616	2.028392	-11.090886
H	-9.076514	2.915996	-11.706236
H	-9.532431	2.378489	-10.089785
H	-10.126190	1.513492	-11.519243
C	-6.832878	1.917449	-10.486813
H	-5.956631	1.275088	-10.369695
H	-7.073359	2.350320	-9.510192
H	-6.563172	2.743981	-11.154968
C	-5.727148	-4.798987	-9.496236
C	-5.111572	-4.703851	-8.236008
C	-4.203683	-5.700335	-7.868791
H	-3.715848	-5.637099	-6.898101
C	-3.905930	-6.753024	-8.721251
H	-3.199822	-7.520448	-8.416267
C	-4.496246	-6.810988	-9.978272
H	-4.236745	-7.622960	-10.651592
C	-5.405245	-5.836822	-10.396562
C	-5.353134	-3.541115	-7.293731
H	-6.170076	-2.933565	-7.704124
C	-4.107283	-2.656599	-7.213141
H	-3.280084	-3.204899	-6.747083
H	-4.300442	-1.768940	-6.600858
H	-3.782073	-2.343331	-8.208486
C	-5.761031	-3.998692	-5.893623
H	-6.636575	-4.656429	-5.900440
H	-5.993863	-3.137544	-5.259467
H	-4.948976	-4.548221	-5.404960
C	-6.019569	-5.892106	-11.785781
H	-6.086272	-4.856706	-12.147288
C	-7.443480	-6.451304	-11.785279
H	-7.474297	-7.450828	-11.335835
H	-7.818032	-6.538422	-12.811571
H	-8.142267	-5.809960	-11.240336
C	-5.161448	-6.656112	-12.787963
H	-4.120916	-6.317897	-12.777545
H	-5.552180	-6.518856	-13.800776
H	-5.163391	-7.734004	-12.590247
C	-4.568695	-2.675787	-12.373531
H	-4.094720	-3.617085	-12.672693
C	-4.019538	-1.457283	-12.877956
H	-3.452604	-1.382532	-13.802609
C	-4.132286	-0.428090	-11.975613
C	-4.587080	-0.918578	-10.687151
C	-3.807198	1.000060	-12.241877
H	-3.296094	1.475541	-11.396716
H	-3.181487	1.103498	-13.132398
H	-4.720090	1.580528	-12.429598
H	-4.316419	-0.313418	-9.818664

4j.log
SCF (M06L) = -1510.66465826
E(SCF)+ZPE(0 K)= -1509.926856
H(298 K)= -1509.884522
G(298 K)= -1509.998468
Lowest Frequency = 24.5836cm-1

Al	-6.054954	-2.228027	-10.914587
O	-5.578192	-0.983906	-9.717442
N	-7.755984	-1.714860	-11.638089
N	-6.741165	-3.724646	-9.938230
C	-8.914444	-2.004961	-11.032112
C	-9.008612	-2.914843	-9.968076
H	-9.991810	-3.039416	-9.529589
C	-8.010419	-3.784212	-9.510861
C	-10.183237	-1.372833	-11.519482
H	-10.296249	-1.504517	-12.600296
H	-10.170454	-0.292231	-11.345632
H	-11.054298	-1.794273	-11.017013
C	-8.401882	-4.850781	-8.533645
H	-7.758278	-4.818563	-7.648279
H	-8.265907	-5.847758	-8.965277
H	-9.441487	-4.744461	-8.222937
C	-7.771612	-0.880581	-12.809267
C	-7.644932	-1.500373	-14.070374
C	-7.692004	-0.692936	-15.208226
H	-7.604633	-1.149498	-16.189807
C	-7.835384	0.686029	-15.108392
H	-7.871921	1.295519	-16.007108
C	-7.902482	1.281329	-13.858009
H	-7.973120	2.364228	-13.777938
C	-7.864054	0.518114	-12.687679
C	-7.465820	-3.002912	-14.190421
H	-6.837783	-3.323928	-13.346034
C	-6.739711	-3.411499	-15.466490
H	-7.355947	-3.249183	-16.357715
H	-6.495089	-4.477168	-15.439710
H	-5.807204	-2.853924	-15.595615
C	-8.795419	-3.749669	-14.070824
H	-9.269322	-3.596399	-13.096112
H	-8.644884	-4.828410	-14.195558
H	-9.498608	-3.421376	-14.845136
C	-7.834635	1.224868	-11.347337
H	-7.905703	0.470755	-10.554584
C	-8.982270	2.218647	-11.175258
H	-8.908602	3.040272	-11.896323
H	-8.957971	2.663913	-10.175945
H	-9.966091	1.757829	-11.311850
C	-6.494807	1.943749	-11.178255
H	-5.648191	1.270663	-11.338726
H	-6.403073	2.365393	-10.171658
H	-6.407259	2.767527	-11.896564
C	-5.864515	-4.823615	-9.637598
C	-5.023608	-4.777580	-8.511296
C	-4.179263	-5.865160	-8.272137
H	-3.522122	-5.838703	-7.404955
C	-4.162819	-6.967154	-9.115973
H	-3.500755	-7.803684	-8.910321
C	-4.992623	-6.990199	-10.229468
H	-4.974994	-7.848496	-10.898757
C	-5.850929	-5.926561	-10.514199
C	-4.987988	-3.588862	-7.573455
H	-5.741117	-2.865817	-7.906501
C	-3.626702	-2.898046	-7.641379
H	-2.834344	-3.558161	-7.268818
H	-3.620819	-1.991165	-7.027527
H	-3.368874	-2.608789	-8.664400
C	-5.311280	-3.988155	-6.134486
H	-6.285627	-4.480790	-6.048853
H	-5.323881	-3.108510	-5.483407
H	-4.561458	-4.679823	-5.733934

C	-6.721758	-5.990151	-11.754455
H	-7.310764	-5.065950	-11.810771
C	-7.701821	-7.161139	-11.699144
H	-7.172678	-8.120057	-11.661626
H	-8.343319	-7.174696	-12.586532
H	-8.351929	-7.111476	-10.819807
C	-5.866299	-6.068473	-13.017411
H	-5.193582	-5.208397	-13.100270
H	-6.499066	-6.096629	-13.911713
H	-5.249079	-6.973762	-13.024800
C	-4.522333	-2.355814	-12.059644
C	-3.462270	-1.529054	-11.797231
C	-3.422238	-0.583154	-10.696182
C	-4.402966	-0.374002	-9.777707
H	-2.523518	0.018700	-10.582137
H	-4.249900	0.366740	-8.987089
C	-2.224285	-1.539251	-12.653480
H	-1.334934	-1.784439	-12.060383
H	-2.300485	-2.263410	-13.468153
H	-2.037069	-0.551036	-13.090539
H	-4.393954	-3.004698	-12.930066

5j.log
SCF (M06L) = -1510.66205658
E(SCF)+ZPE(0 K)= -1509.924243
H(298 K)= -1509.881953
G(298 K)= -1509.995801
Lowest Frequency = 23.6684cm-1

Al	-6.049931	-2.222583	-10.900419
O	-5.588255	-0.968438	-9.717881
N	-7.743847	-1.727088	-11.647273
N	-6.733516	-3.718135	-9.922796
C	-8.904830	-2.012674	-11.043589
C	-9.002277	-2.912493	-9.971252
H	-9.987136	-3.033919	-9.535610
C	-8.005030	-3.776452	-9.501447
C	-10.171757	-1.386145	-11.542596
H	-10.280320	-1.529023	-12.622450
H	-10.159753	-0.303755	-11.379848
H	-11.044769	-1.802588	-11.039371
C	-8.399916	-4.836148	-8.518224
H	-7.752737	-4.805294	-7.635492
H	-8.272893	-5.835711	-8.946713
H	-9.437785	-4.721669	-8.204590
C	-7.754019	-0.905203	-12.827282
C	-7.619887	-1.538748	-14.080749
C	-7.661352	-0.744026	-15.227709
H	-7.568191	-1.211101	-16.203757
C	-7.806336	0.635787	-15.143717
H	-7.838375	1.235359	-16.049239
C	-7.880440	1.244800	-13.900335
H	-7.951952	2.328467	-13.832632
C	-7.847694	0.494669	-12.721398
C	-7.438443	-3.042333	-14.183133
H	-6.814409	-3.353460	-13.331863
C	-6.706674	-3.464299	-15.451627
H	-7.319711	-3.312111	-16.346810
H	-6.461426	-4.529382	-15.412529
H	-5.774219	-2.907397	-15.583861
C	-8.767925	-3.789092	-14.061544
H	-9.247084	-3.625053	-13.091200
H	-8.615827	-4.869067	-14.173143
H	-9.467244	-3.470280	-14.843267
C	-7.824871	1.215824	-11.388674
H	-7.901450	0.470477	-10.588185
C	-8.972164	2.212779	-11.233939
H	-8.893120	3.027126	-11.962630
H	-8.953555	2.667970	-10.239003
H	-9.955620	1.751570	-11.372057
C	-6.484619	1.933972	-11.219725
H	-5.638774	1.255985	-11.363063
H	-6.399312	2.369266	-10.218429
H	-6.389380	2.747153	-11.949024
C	-5.857149	-4.814353	-9.611160
C	-5.010928	-4.754996	-8.489343
C	-4.169168	-5.842030	-8.238500
H	-3.508767	-5.805615	-7.374169
C	-4.159062	-6.955548	-9.067041
H	-3.498675	-7.791125	-8.852367
C	-4.992542	-6.990974	-10.177412
H	-4.978705	-7.857595	-10.835967
C	-5.849195	-5.929028	-10.473081
C	-4.966901	-3.554213	-7.567242
H	-5.712354	-2.828541	-7.911475
C	-3.598545	-2.877414	-7.639547
H	-2.813739	-3.539616	-7.254938
H	-3.585988	-1.961891	-7.038844
H	-3.335026	-2.604943	-8.665779
C	-5.298567	-3.932380	-6.124462
H	-6.279508	-4.411168	-6.035733
H	-5.301443	-3.045075	-5.483755
H	-4.558634	-4.629068	-5.714267
C	-6.720064	-6.005252	-11.712595
H	-7.314682	-5.085041	-11.774944
C	-7.692559	-7.182192	-11.653033
H	-7.157892	-8.137926	-11.613952
H	-8.334894	-7.201846	-12.539681
H	-8.342007	-7.134467	-10.773132
C	-5.861577	-6.085076	-12.973542
H	-5.194695	-5.220831	-13.060552
H	-6.491910	-6.123427	-13.869153
H	-5.237619	-6.985749	-12.973389
C	-4.494117	-2.343241	-12.019498
H	-4.328112	-2.983156	-12.888067
C	-3.463981	-1.502686	-11.708804
H	-2.557705	-1.523305	-12.326394
C	-3.397902	-0.542610	-10.624473
C	-4.415788	-0.347731	-9.737570
C	-2.139000	0.258604	-10.469706
H	-1.914199	0.851726	-11.365164
H	-2.207470	0.954223	-9.627792
H	-1.263694	-0.379924	-10.293537
H	-4.293463	0.392270	-8.939056

[Pd(1)$_2$].log

SCF (M06L) = -2610.58136399
E(SCF)+ZPE(0 K)= -2609.302627
H(298 K)= -2609.228276
G(298 K)= -2609.409952
Lowest Frequency = 16.2727cm-1

Pd	0.004121000	-0.065181000	-1.394658000
Al	2.149727000	-0.207636000	-0.377820000
N	3.392991000	1.323921000	-0.175072000

N	2.874528000	-1.038878000	1.281248000
C	4.061002000	1.678612000	0.926832000
C	4.151536000	0.851651000	2.056292000
H	4.740979000	1.232170000	2.882836000
C	3.679530000	-0.459255000	2.183656000
C	4.792716000	2.987883000	0.969405000
H	5.453947000	3.099514000	0.104165000
H	5.381933000	3.085446000	1.882304000
H	4.086010000	3.823445000	0.922345000
C	4.129424000	-1.239795000	3.384487000
H	3.279127000	-1.531263000	4.010193000
H	4.825910000	-0.663393000	3.994510000
H	4.614776000	-2.173467000	3.082097000
C	3.539710000	2.130970000	-1.358216000
C	4.422756000	1.671187000	-2.361682000
C	4.592887000	2.456188000	-3.503242000
H	5.271887000	2.124468000	-4.283238000
C	3.897502000	3.648458000	-3.668757000
H	4.045565000	4.245121000	-4.564733000
C	2.994894000	4.055850000	-2.698736000
H	2.419203000	4.967730000	-2.844623000
C	2.791258000	3.309386000	-1.534654000
C	5.158760000	0.351791000	-2.209161000
H	4.446602000	-0.355556000	-1.750987000
C	5.584399000	-0.259540000	-3.537691000
H	4.743288000	-0.352264000	-4.231201000
H	6.000797000	-1.258591000	-3.374491000
H	6.366230000	0.330657000	-4.028495000
C	6.363530000	0.456174000	-1.272924000
H	7.083227000	1.192897000	-1.648878000
H	6.881185000	-0.507461000	-1.204355000
H	6.081095000	0.746993000	-0.257440000
C	1.726113000	3.753166000	-0.554874000
H	1.803535000	3.120611000	0.335693000
C	0.336866000	3.531722000	-1.154116000
H	-0.443812000	3.806097000	-0.435849000
H	0.182270000	2.477753000	-1.429139000
H	0.199770000	4.144884000	-2.053769000
C	1.882787000	5.207976000	-0.116128000
H	2.874848000	5.423025000	0.296497000
H	1.136299000	5.452436000	0.647737000
H	1.724019000	5.897788000	-0.952691000
C	2.572920000	-2.435317000	1.436406000
C	1.543478000	-2.860400000	2.301077000
C	1.259720000	-4.226025000	2.375544000
H	0.460705000	-4.561438000	3.034165000
C	1.964893000	-5.157648000	1.624612000
H	1.721606000	-6.214309000	1.694932000
C	2.978242000	-4.725069000	0.781615000
H	3.533582000	-5.449048000	0.188414000
C	3.301346000	-3.370017000	0.670752000
C	0.721312000	-1.897229000	3.135358000
H	1.205834000	-0.914633000	3.096321000
C	-0.681573000	-1.740471000	2.550060000
H	-1.172416000	-2.715285000	2.436074000
H	-0.636043000	-1.268728000	1.558420000
H	-1.318940000	-1.127223000	3.197971000
C	0.639939000	-2.321460000	4.601216000
H	0.060597000	-3.242944000	4.723620000
H	0.141775000	-1.548435000	5.195593000
H	1.628055000	-2.499467000	5.038758000
C	4.421323000	-2.964334000	-0.268418000
H	4.576407000	-1.881513000	-0.170778000
C	4.044881000	-3.256653000	-1.720975000
H	3.843524000	-4.324154000	-1.868299000
H	4.857528000	-2.974770000	-2.399279000
H	3.145701000	-2.704309000	-2.019604000
C	5.740182000	-3.642642000	0.098771000
H	6.038914000	-3.422177000	1.128545000
H	6.544436000	-3.306771000	-0.564238000
H	5.672223000	-4.731884000	0.001667000

Al	-2.129338000	0.166050000	-0.362975000
N	-3.394498000	-1.339659000	-0.085919000
N	-2.845512000	1.090910000	1.246682000
C	-4.169324000	-1.572692000	0.975232000
C	-4.216871000	-0.710423000	2.080798000
H	-4.850212000	-1.021068000	2.904291000
C	-3.658801000	0.568780000	2.178743000
C	-5.096726000	-2.753248000	0.987068000
H	-5.994458000	-2.531022000	0.397918000
H	-5.418223000	-2.990611000	2.002830000
H	-4.642482000	-3.636668000	0.531854000
C	-4.032424000	1.385234000	3.382943000
H	-3.161276000	1.557516000	4.023560000
H	-4.798617000	0.884857000	3.976534000
H	-4.398732000	2.375419000	3.093724000
C	-3.565838000	-2.157081000	-1.256114000
C	-4.649562000	-1.893305000	-2.117751000
C	-4.791674000	-2.674488000	-3.266191000
H	-5.621053000	-2.476156000	-3.942289000
C	-3.886098000	-3.683639000	-3.564846000
H	-4.013920000	-4.284961000	-4.460836000
C	-2.806544000	-3.909236000	-2.720959000
H	-2.088201000	-4.688523000	-2.963703000
C	-2.615223000	-3.151015000	-1.562958000
C	-5.606315000	-0.738460000	-1.886366000
H	-5.445965000	-0.346706000	-0.873363000
C	-5.295395000	0.391060000	-2.869980000
H	-5.450950000	0.058851000	-3.902684000
H	-4.252642000	0.721081000	-2.787256000
H	-5.946148000	1.255977000	-2.695631000
C	-7.072698000	-1.149158000	-1.994273000
H	-7.727379000	-0.308879000	-1.740450000
H	-7.318960000	-1.980989000	-1.326182000
H	-7.329850000	-1.462801000	-3.011846000
C	-1.441971000	-3.442959000	-0.650255000
H	-1.262458000	-2.545268000	-0.046613000
C	-1.771709000	-4.595635000	0.298537000
H	-2.627046000	-4.365641000	0.943753000
H	-0.915321000	-4.819069000	0.943869000
H	-2.015556000	-5.506438000	-0.261928000
C	-0.144736000	-3.714394000	-1.403942000
H	0.686874000	-3.793995000	-0.693876000
H	0.088338000	-2.891370000	-2.090026000
H	-0.179187000	-4.651361000	-1.973375000
C	-2.495010000	2.480869000	1.355528000
C	-1.481668000	2.913973000	2.232665000
C	-1.198577000	4.281107000	2.301102000
H	-0.412717000	4.622993000	2.972421000
C	-1.891283000	5.202214000	1.528653000
H	-1.660347000	6.261677000	1.600916000
C	-2.865345000	4.757932000	0.642880000
H	-3.387623000	5.478224000	0.019542000
C	-3.175827000	3.401339000	0.527034000
C	-0.653443000	1.954076000	3.062249000
H	-1.118920000	0.964006000	3.001224000
C	0.753758000	1.838219000	2.478729000
H	1.251131000	2.817145000	2.466925000
H	0.720616000	1.462476000	1.446131000
H	1.380819000	1.164981000	3.073854000
C	-0.575238000	2.354932000	4.534523000
H	-0.061318000	1.581112000	5.114665000
H	-1.562701000	2.510962000	4.981065000
H	-0.010173000	3.283915000	4.668100000
C	-4.235647000	2.939202000	-0.455557000
H	-3.939717000	1.938611000	-0.809125000
C	-4.325994000	3.823691000	-1.692228000
H	-4.737030000	4.813172000	-1.463822000
H	-4.986487000	3.368355000	-2.436059000
H	-3.342837000	3.962556000	-2.153722000
C	-5.600807000	2.786757000	0.215200000
H	-5.937163000	3.740957000	0.637527000

H	-5.578235000	2.049348000	1.024215000
H	-6.355295000	2.457402000	-0.508714000

[Pd(1)PCy$_3$].log

SCF (M06L) = -2416.41103039
E(SCF)+ZPE(0 K)= -2415.286339
H(298 K)= -2415.227133
G(298 K)= -2415.380701
Lowest Frequency = 13.8384 cm-1

Pd	0.561963000	-0.353021000	-0.963757000
P	2.767456000	-0.368323000	-0.256873000
C	3.474321000	1.356343000	-0.206068000
H	4.523674000	1.306295000	0.127453000
C	2.721021000	2.275312000	0.758733000
H	1.652571000	2.282945000	0.483561000
H	2.774571000	1.889817000	1.785731000
C	3.280328000	3.693220000	0.719817000
H	2.728640000	4.330492000	1.420827000
H	4.324616000	3.680490000	1.068308000
C	3.228457000	4.270676000	-0.688159000
H	3.639534000	5.287247000	-0.706880000
H	2.175026000	4.351869000	-0.996461000
C	3.970648000	3.371396000	-1.669018000
H	5.042376000	3.366440000	-1.417021000
H	3.896752000	3.766812000	-2.689219000
C	3.436371000	1.944595000	-1.622316000
H	3.995480000	1.307284000	-2.320498000
H	2.388439000	1.925191000	-1.964780000
C	4.091712000	-1.291363000	-1.216362000
H	3.823974000	-1.060730000	-2.261352000
C	5.556902000	-0.882991000	-1.030298000
H	5.869729000	-1.076574000	0.005196000
H	5.687502000	0.192912000	-1.194877000
C	6.463599000	-1.654709000	-1.987232000
H	6.213493000	-1.368325000	-3.019826000
H	7.509493000	-1.363883000	-1.833535000
C	6.296181000	-3.161318000	-1.834287000
H	6.632577000	-3.460677000	-0.830149000
H	6.938216000	-3.694251000	-2.545067000
C	4.838712000	-3.569441000	-2.011933000
H	4.719205000	-4.649707000	-1.869233000
H	4.524373000	-3.356908000	-3.044243000
C	3.937151000	-2.805741000	-1.048689000
H	2.886071000	-3.095759000	-1.177096000
H	4.212434000	-3.079504000	-0.018736000
C	2.859765000	-0.896761000	1.527876000
H	2.260315000	-0.099207000	1.997409000
C	2.095232000	-2.193588000	1.816404000
H	1.124011000	-2.165885000	1.302559000
H	2.640546000	-3.053020000	1.402779000
C	1.925263000	-2.388271000	3.319250000
H	1.392402000	-3.324210000	3.526241000
H	1.290921000	-1.578947000	3.713812000
C	3.270060000	-2.369426000	4.038579000
H	3.132369000	-2.478843000	5.120627000
H	3.861495000	-3.238881000	3.714352000
C	4.050762000	-1.097066000	3.726401000
H	5.026167000	-1.109709000	4.226941000
H	3.505747000	-0.229627000	4.131026000
C	4.222366000	-0.906495000	2.220890000
H	4.830083000	-1.731830000	1.818965000
H	4.777698000	0.017587000	2.012477000
Al	-1.747191000	0.127060000	-0.651298000
N	-2.581626000	1.783699000	0.000072000
N	-3.421354000	-0.853921000	-0.362030000
C	-3.893392000	2.031739000	0.115719000
C	-4.867401000	1.034926000	-0.028410000
H	-5.900132000	1.349103000	0.070926000
C	-4.642270000	-0.339786000	-0.174603000
C	-4.359839000	3.423199000	0.430684000
H	-3.893330000	3.798182000	1.347584000
H	-5.443922000	3.462919000	0.543976000
H	-4.065253000	4.118374000	-0.363350000
C	-5.822481000	-1.259999000	-0.081617000
H	-5.830798000	-1.976959000	-0.908467000
H	-6.761532000	-0.705030000	-0.076931000
H	-5.772006000	-1.858084000	0.835306000
C	-1.662287000	2.830699000	0.351269000
C	-1.086374000	2.806152000	1.639200000
C	-0.254465000	3.865204000	2.008451000
H	0.194517000	3.871431000	2.998465000
C	0.031751000	4.897603000	1.122673000
H	0.678045000	5.716273000	1.428917000
C	-0.473779000	4.856733000	-0.169141000
H	-0.201182000	5.635567000	-0.878375000
C	-1.318810000	3.824021000	-0.582805000
C	-1.348151000	1.653085000	2.590257000
H	-1.465008000	0.750494000	1.971068000
C	-0.178615000	1.385394000	3.529781000
H	0.761625000	1.283419000	2.975703000
H	-0.344268000	0.457192000	4.085997000
H	-0.050632000	2.182840000	4.270334000
C	-2.646457000	1.831031000	3.377566000
H	-2.617278000	2.747595000	3.978619000
H	-2.803263000	0.988885000	4.061767000
H	-3.520678000	1.885944000	2.721223000
C	-1.759715000	3.748916000	-2.029788000
H	-2.572373000	3.014643000	-2.101184000
C	-0.594687000	3.236544000	-2.881509000
H	-0.893126000	3.117062000	-3.928512000
H	-0.227717000	2.268779000	-2.513131000
H	0.244182000	3.943650000	-2.849443000
C	-2.285273000	5.076892000	-2.567439000
H	-3.097175000	5.479532000	-1.952126000
H	-2.664636000	4.956036000	-3.586662000
H	-1.497944000	5.837312000	-2.607023000
C	-3.258118000	-2.281258000	-0.339055000
C	-3.163285000	-2.996288000	-1.546739000
C	-2.943142000	-4.374792000	-1.482966000
H	-2.868147000	-4.938866000	-2.410639000
C	-2.810558000	-5.029576000	-0.266602000
H	-2.638556000	-6.102001000	-0.238982000
C	-2.887753000	-4.304199000	0.916049000
H	-2.762761000	-4.813354000	1.869398000
C	-3.107936000	-2.925909000	0.905192000
C	-3.272326000	-2.328025000	-2.902390000
H	-3.470279000	-1.260834000	-2.740379000
C	-1.956936000	-2.445349000	-3.672173000
H	-1.713843000	-3.494900000	-3.874392000
H	-1.126463000	-2.008500000	-3.104585000
H	-2.023308000	-1.928642000	-4.635390000
C	-4.433010000	-2.895501000	-3.718279000
H	-4.282226000	-3.958525000	-3.937388000
H	-4.524940000	-2.375073000	-4.676832000
H	-5.389031000	-2.802915000	-3.192533000
C	-3.099084000	-2.154034000	2.211062000
H	-3.488192000	-1.145369000	2.020408000
C	-1.661590000	-2.005716000	2.712833000
H	-1.217920000	-2.988361000	2.912229000
H	-1.625784000	-1.427545000	3.643957000
H	-1.022159000	-1.506219000	1.971665000
C	-3.980902000	-2.789337000	3.282581000
H	-5.009408000	-2.932808000	2.935625000
H	-4.012569000	-2.158497000	4.176878000
H	-3.600628000	-3.768488000	3.593122000

Int-3_M06L.log

SCF (M06L) = -2879.92452594
E(SCF)+ZPE(0 K)= -2878.546762
H(298 K)= -2878.465871
G(298 K)= -2878.659799
Lowest Frequency = 21.2400 cm-1

Pd	0.010129000	-0.073170000	1.215736000
Al	-1.965290000	-0.152428000	-0.120663000
N	-3.141775000	1.448708000	-0.400414000
N	-2.624025000	-0.936917000	-1.833375000
C	-3.850796000	1.764531000	-1.488662000
C	-3.946404000	0.922888000	-2.605873000
H	-4.573863000	1.275328000	-3.417501000
C	-3.474225000	-0.390547000	-2.714163000
C	-4.681558000	3.017867000	-1.516788000
H	-5.652122000	2.841139000	-1.037663000
H	-4.876197000	3.331921000	-2.544395000
H	-4.210898000	3.837187000	-0.968990000
C	-4.039246000	-1.220789000	-3.834038000
H	-3.301831000	-1.887783000	-4.285803000
H	-4.476775000	-0.588479000	-4.608734000
H	-4.835024000	-1.866830000	-3.443661000
C	-3.353039000	2.218940000	0.792922000
C	-4.546599000	2.028251000	1.521822000
C	-4.730766000	2.758497000	2.697836000
H	-5.644677000	2.620045000	3.271874000
C	-3.758576000	3.638769000	3.156767000
H	-3.915705000	4.193199000	4.078179000
C	-2.580085000	3.795711000	2.440666000
H	-1.815809000	4.474471000	2.809298000
C	-2.349523000	3.095106000	1.253747000
C	-5.580531000	0.994642000	1.111614000
H	-5.437641000	0.755514000	0.051086000
C	-5.356562000	-0.300722000	1.893197000
H	-4.325914000	-0.665412000	1.781903000
H	-6.038288000	-1.089635000	1.553418000
H	-5.532745000	-0.142110000	2.963930000
C	-7.016792000	1.482619000	1.276635000
H	-7.281084000	1.625870000	2.329815000
H	-7.720305000	0.750040000	0.868971000
H	-7.187065000	2.435703000	0.764836000
C	-1.088971000	3.342875000	0.451043000
H	-0.839897000	2.409595000	-0.072848000
C	0.126094000	3.682711000	1.303920000
H	1.021899000	3.700519000	0.672888000
H	0.282393000	2.928146000	2.083083000
H	0.042320000	4.667889000	1.779615000
C	-1.330411000	4.427798000	-0.598486000
H	-2.105106000	4.135903000	-1.314979000
H	-0.413499000	4.628576000	-1.162956000
H	-1.648282000	5.365106000	-0.124908000
C	-2.287130000	-2.320368000	-2.018164000
C	-1.116113000	-2.656622000	-2.730216000
C	-0.864600000	-4.007428000	-2.981532000
H	0.016443000	-4.284757000	-3.555151000
C	-1.710799000	-5.003478000	-2.507617000
H	-1.493601000	-6.047753000	-2.715392000
C	-2.815516000	-4.656656000	-1.743417000
H	-3.454395000	-5.436183000	-1.332875000
C	-3.122809000	-3.318709000	-1.480200000
C	-0.175628000	-1.589365000	-3.259629000
H	-0.285335000	-0.708426000	-2.610396000
C	1.292259000	-1.999637000	-3.210218000
H	1.512600000	-2.860011000	-3.852483000
H	1.614885000	-2.248209000	-2.194580000
H	1.925479000	-1.177502000	-3.562016000
C	-0.546896000	-1.176850000	-4.684804000
H	-0.552723000	-2.045912000	-5.353804000
H	0.183154000	-0.461086000	-5.078983000
H	-1.532522000	-0.705280000	-4.739780000
C	-4.297467000	-3.001679000	-0.573930000
H	-4.454926000	-1.913890000	-0.575270000
C	-3.965096000	-3.421515000	0.860205000
H	-3.733648000	-4.492360000	0.907037000
H	-4.807883000	-3.229335000	1.532621000
H	-3.093572000	-2.873950000	1.239834000
C	-5.594199000	-3.658815000	-1.040962000
H	-5.842958000	-3.393442000	-2.073718000
H	-6.431518000	-3.353955000	-0.404727000
H	-5.532093000	-4.751543000	-0.990968000
Al	2.060032000	0.140374000	0.035574000
N	3.301787000	-1.439427000	-0.001262000
N	3.046064000	0.960130000	-1.477909000
C	4.236853000	-1.718740000	-0.910634000
C	4.456656000	-0.913649000	-2.040663000
H	5.185278000	-1.279297000	-2.755811000
C	3.946384000	0.366409000	-2.278396000
C	5.156148000	-2.891031000	-0.718843000
H	4.645543000	-3.748216000	-0.273394000
H	5.967011000	-2.620699000	-0.031960000
H	5.612811000	-3.190649000	-1.664124000
C	4.469101000	1.103439000	-3.477882000
H	4.850838000	2.092387000	-3.204372000
H	3.666026000	1.275715000	-4.203168000
H	5.263394000	0.543291000	-3.973213000
C	3.271669000	-2.200716000	1.217014000
C	4.201263000	-1.911373000	2.235927000
C	4.135814000	-2.639428000	3.426813000
H	4.845762000	-2.417429000	4.221182000
C	3.177279000	-3.624758000	3.615690000
H	3.143896000	-4.184918000	4.546098000
C	2.246921000	-3.875571000	2.614716000
H	1.480641000	-4.630031000	2.772152000
C	2.261668000	-3.166105000	1.410861000
C	5.211889000	-0.786255000	2.122330000
H	5.210853000	-0.414479000	1.088931000
C	4.794632000	0.372259000	3.029237000
H	4.764976000	0.053159000	4.077332000
H	3.795734000	0.743760000	2.767425000
H	5.503559000	1.204616000	2.953563000
C	6.633736000	-1.233923000	2.454577000
H	7.345391000	-0.420225000	2.280657000
H	6.949286000	-2.091619000	1.851691000
H	6.727052000	-1.525486000	3.506404000
C	1.242526000	-3.474209000	0.334382000
H	1.071281000	-2.546194000	-0.230613000
C	1.785222000	-4.534675000	-0.624384000
H	2.677850000	-4.187727000	-1.156525000
H	1.033263000	-4.798422000	-1.374582000
H	2.054518000	-5.447844000	-0.079633000
C	-0.117307000	-3.882441000	0.887186000
H	-0.844335000	-3.939498000	0.070347000
H	-0.486908000	-3.141562000	1.606624000
H	-0.096156000	-4.866907000	1.370967000
C	2.797486000	2.365166000	-1.652581000
C	1.880545000	2.839000000	-2.610224000
C	1.700110000	4.220376000	-2.731684000
H	0.989300000	4.595715000	-3.465454000
C	2.392569000	5.115361000	-1.930641000
H	2.239900000	6.184975000	-2.045308000
C	3.263039000	4.633019000	-0.961570000
H	3.780105000	5.334962000	-0.313840000
C	3.471682000	3.262937000	-0.791244000
C	1.048462000	1.924118000	-3.487674000
H	1.413534000	0.896435000	-3.356564000
C	-0.416981000	1.956840000	-3.050051000
H	-0.818954000	2.975619000	-3.107090000
H	-0.529944000	1.611800000	-2.013874000
H	-1.038525000	1.320043000	-3.688981000

C	1.161237000	2.292286000	-4.967448000
H	0.610424000	1.579441000	-5.589486000
H	2.199262000	2.312867000	-5.315336000
H	0.735005000	3.281661000	-5.164988000
C	4.402528000	2.762930000	0.299124000
H	3.985545000	1.812171000	0.669852000
C	4.473302000	3.705252000	1.494780000
H	4.984232000	4.643215000	1.251292000
H	5.036477000	3.241373000	2.309384000
H	3.475736000	3.951282000	1.873127000
C	5.803999000	2.461868000	-0.232964000
H	6.255743000	3.359382000	-0.671821000
H	5.795910000	1.678967000	-0.997590000
H	6.459548000	2.122024000	0.577460000
H	1.015133000	1.110549000	3.552867000
C	0.191860000	0.423329000	3.674023000
C	0.083633000	-0.946219000	3.598388000
O	-0.953085000	0.953567000	4.205518000
C	-1.215251000	-1.264468000	4.103289000
H	0.868344000	-1.631525000	3.299351000
C	-1.805168000	-0.087546000	4.452382000
H	-1.666225000	-2.245384000	4.167408000
C	-3.141703000	0.269229000	4.963927000
H	-3.725434000	-0.635415000	5.146981000
H	-3.083281000	0.836607000	5.899426000
H	-3.686805000	0.891306000	4.243642000

Int-3'_M06L.log

SCF (M06L) = -2685.73904432
E(SCF)+ZPE(0 K)= -2684.51646
H(298 K)= -2684.451095
G(298 K)= -2684.614578
Lowest Frequency = 15.9359 cm-1

Pd	0.957700000	-0.146985000	-1.195540000
Al	-1.355317000	0.162888000	-0.499482000
N	-2.531425000	1.760803000	-0.425930000
N	-2.927264000	-0.981099000	-0.903088000
C	-3.713463000	1.880793000	-1.044014000
C	-4.380941000	0.794116000	-1.625713000
H	-5.307945000	1.018189000	-2.141197000
C	-4.063961000	-0.562535000	-1.475381000
C	-4.398565000	3.215627000	-1.089749000
H	-5.214690000	3.213798000	-1.813857000
H	-3.695257000	4.015932000	-1.337773000
H	-4.816457000	3.471387000	-0.109692000
C	-5.070413000	-1.568129000	-1.953747000
H	-4.636011000	-2.222477000	-2.715985000
H	-5.951868000	-1.080707000	-2.372103000
H	-5.385713000	-2.226358000	-1.137269000
C	-2.072949000	2.872204000	0.359680000
C	-2.652287000	3.095233000	1.627647000
C	-2.236661000	4.202429000	2.369019000
H	-2.677031000	4.378497000	3.348619000
C	-1.266359000	5.070307000	1.884119000
H	-0.962705000	5.934536000	2.468585000
C	-0.666403000	4.804746000	0.662081000
H	0.118985000	5.461444000	0.292644000
C	-1.038691000	3.701451000	-0.113006000
C	-3.635887000	2.121343000	2.247496000
H	-3.959353000	1.409437000	1.477546000
C	-2.927977000	1.323240000	3.344025000
H	-2.053722000	0.786308000	2.953447000
H	-3.605484000	0.590234000	3.796646000
H	-2.574865000	1.987462000	4.141496000
C	-4.883786000	2.800888000	2.805193000
H	-4.643080000	3.464682000	3.642706000
H	-5.594746000	2.056343000	3.177880000
H	-5.397385000	3.403485000	2.049209000
C	-0.290538000	3.432307000	-1.401206000
H	-0.653745000	2.492458000	-1.834323000
C	1.194288000	3.235516000	-1.095689000
H	1.746401000	2.923214000	-1.986747000
H	1.324115000	2.446360000	-0.344555000
H	1.654384000	4.155408000	-0.712301000
C	-0.512609000	4.532752000	-2.434724000
H	-1.576342000	4.677919000	-2.650726000
H	-0.016841000	4.278062000	-3.376717000
H	-0.111934000	5.494025000	-2.090652000
C	-2.792413000	-2.380956000	-0.611414000
C	-2.311481000	-3.281757000	-1.579532000
C	-2.260770000	-4.641422000	-1.255419000
H	-1.894846000	-5.345443000	-2.000333000
C	-2.661736000	-5.102756000	-0.011767000
H	-2.628607000	-6.164437000	0.216794000
C	-3.069535000	-4.192744000	0.956920000
H	-3.334161000	-4.553358000	1.947178000
C	-3.118856000	-2.823328000	0.690635000
C	-1.783720000	-2.838631000	-2.929012000
H	-1.933196000	-1.753617000	-3.014803000
C	-0.280738000	-3.115041000	-3.003545000
H	-0.078257000	-4.187753000	-2.904981000
H	0.254942000	-2.588761000	-2.201533000
H	0.134906000	-2.784048000	-3.961691000
C	-2.499126000	-3.516644000	-4.096606000
H	-2.331971000	-4.599552000	-4.088087000
H	-2.121387000	-3.137412000	-5.051250000
H	-3.581761000	-3.355782000	-4.079230000
C	-3.490946000	-1.838893000	1.785246000
H	-2.888818000	-0.931056000	1.615133000
C	-3.147807000	-2.353181000	3.179145000
H	-3.804210000	-3.175693000	3.483911000
H	-3.272540000	-1.557872000	3.919411000
H	-2.114364000	-2.709916000	3.238634000
C	-4.960709000	-1.419051000	1.726663000
H	-5.205652000	-0.889208000	0.801418000
H	-5.203480000	-0.749784000	2.560527000
H	-5.617715000	-2.293613000	1.802705000
H	-0.202873000	-0.504270000	-3.718819000
C	0.652031000	0.054359000	-3.352146000
C	1.983366000	-0.419221000	-3.162183000
O	0.719579000	1.330187000	-3.948866000
C	2.839164000	0.635855000	-3.648935000
H	2.268600000	-1.466317000	-3.133124000
C	2.046550000	1.644066000	-4.090883000
H	3.921685000	0.639966000	-3.662102000
C	2.348063000	2.967889000	-4.676636000
H	3.415238000	3.047305000	-4.893532000
H	1.795234000	3.124536000	-5.609455000
H	2.082399000	3.795984000	-4.006922000
P	2.459008000	-0.604918000	0.598433000
C	4.203576000	-0.862076000	-0.030964000
C	4.757133000	0.459777000	-0.574586000
C	5.247233000	-1.590975000	0.821627000
H	4.019891000	-1.502885000	-0.910977000
C	6.043692000	0.235805000	-1.360051000
H	4.965352000	1.144532000	0.261689000
H	3.995361000	0.949430000	-1.197910000
C	6.537775000	-1.799019000	0.030370000
H	5.471797000	-1.017811000	1.731442000
H	4.859308000	-2.560663000	1.158026000
C	7.085581000	-0.483271000	-0.510744000
H	6.436578000	1.189642000	-1.731892000
H	5.818574000	-0.374843000	-2.247887000
H	7.286590000	-2.302660000	0.652896000
H	6.333067000	-2.475214000	-0.813038000
H	8.002232000	-0.657211000	-1.085937000
H	7.369100000	0.163628000	0.333226000
C	2.125431000	-2.176530000	1.562171000
C	2.663827000	-2.260681000	2.994209000

C	0.639992000	-2.560287000	1.538737000
H	2.656040000	-2.936798000	0.961027000
C	2.444145000	-3.648419000	3.590184000
H	2.137529000	-1.519213000	3.614209000
H	3.725542000	-1.994811000	3.032759000
C	0.411210000	-3.938925000	2.147072000
H	0.062579000	-1.802538000	2.096571000
H	0.256062000	-2.517945000	0.510773000
C	0.971285000	-4.035416000	3.560340000
H	2.836156000	-3.687111000	4.613417000
H	3.024698000	-4.381722000	3.009658000
H	-0.657769000	-4.182093000	2.133198000
H	0.899722000	-4.693029000	1.510942000
H	0.827522000	-5.042950000	3.967770000
H	0.410035000	-3.353798000	4.218032000
C	2.588304000	0.847670000	1.779010000
C	1.235541000	1.166462000	2.428348000
C	3.708682000	0.919295000	2.824056000
H	2.784802000	1.665340000	1.063043000
C	1.258163000	2.522405000	3.123914000
H	0.986605000	0.379231000	3.159575000
H	0.445582000	1.138857000	1.665474000
C	3.729444000	2.292036000	3.493928000
H	3.561136000	0.151706000	3.593351000
H	4.682851000	0.717188000	2.366478000
C	2.388303000	2.615063000	4.141086000
H	0.287430000	2.723294000	3.594169000
H	1.388554000	3.305116000	2.360487000
H	4.538300000	2.338725000	4.232746000
H	3.958663000	3.056195000	2.735476000
H	2.412961000	3.608076000	4.605000000
H	2.202561000	1.898410000	4.955518000

Int-4_M06L.log

SCF (M06L) = -2879.91339088
E(SCF)+ZPE(0 K)= -2878.538295
H(298 K)= -2878.458098
G(298 K)= -2878.649952
Lowest Frequency = 15.0024 cm-1

Pd	-0.672845000	-1.302482000	-0.207910000
Al	-1.034148000	0.939460000	0.130963000
N	-1.777871000	1.693228000	1.754267000
N	-1.707244000	2.339553000	-1.003592000
C	-2.259269000	2.934803000	1.871531000
C	-2.311505000	3.833651000	0.794575000
H	-2.646998000	4.838566000	1.025384000
C	-2.139183000	3.535061000	-0.558780000
C	-2.836735000	3.407548000	3.172412000
H	-3.915479000	3.217385000	3.192059000
H	-2.697676000	4.485491000	3.280968000
H	-2.403378000	2.894212000	4.032510000
C	-2.483519000	4.605793000	-1.551126000
H	-1.609569000	4.864574000	-2.158646000
H	-2.840617000	5.506300000	-1.050519000
H	-3.250549000	4.264841000	-2.253932000
C	-2.006234000	0.753123000	2.824132000
C	-3.291882000	0.205097000	2.994853000
C	-3.472102000	-0.740067000	4.007050000
H	-4.459538000	-1.176270000	4.146329000
C	-2.419863000	-1.146267000	4.811142000
H	-2.579385000	-1.895318000	5.582259000
C	-1.156140000	-0.600760000	4.620348000
H	-0.335290000	-0.926838000	5.251084000
C	-0.920766000	0.357262000	3.632337000
C	-4.477956000	0.550450000	2.113575000
H	-4.190688000	1.361044000	1.429593000
C	-4.883128000	-0.652242000	1.261303000
H	-4.065574000	-0.996818000	0.619424000
H	-5.746625000	-0.403209000	0.633078000
H	-5.171900000	-1.499503000	1.894047000
C	-5.677882000	1.031933000	2.930736000
H	-6.089682000	0.220101000	3.540213000
H	-6.480011000	1.377157000	2.269877000
H	-5.425918000	1.849816000	3.613660000
C	0.451274000	0.985042000	3.472239000
H	0.668718000	1.045605000	2.391255000
C	1.567124000	0.163853000	4.102149000
H	2.539384000	0.572290000	3.810771000
H	1.531120000	-0.880229000	3.777609000
H	1.514175000	0.190375000	5.197320000
C	0.488960000	2.411253000	4.025720000
H	-0.194245000	3.083900000	3.499762000
H	1.497928000	2.828118000	3.931122000
H	0.218415000	2.418662000	5.088418000
C	-1.860802000	2.070921000	-2.413224000
C	-0.869561000	2.455703000	-3.333616000
C	-1.122342000	2.270591000	-4.696731000
H	-0.361806000	2.561899000	-5.418609000
C	-2.326553000	1.743408000	-5.139502000
H	-2.516946000	1.632307000	-6.203425000
C	-3.273398000	1.321172000	-4.213418000
H	-4.192513000	0.858737000	-4.562113000
C	-3.051274000	1.441520000	-2.840232000
C	0.460308000	3.041281000	-2.904976000
H	0.405552000	3.245073000	-1.827872000
C	1.586548000	2.032966000	-3.134958000
H	1.646370000	1.742531000	-4.190667000
H	1.437372000	1.118329000	-2.548791000
H	2.558023000	2.456192000	-2.852798000
C	0.783962000	4.353469000	-3.617882000
H	0.906482000	4.204366000	-4.696070000
H	1.724102000	4.771518000	-3.243000000
H	0.002806000	5.108625000	-3.481916000
C	-4.065531000	0.897152000	-1.849834000
H	-3.504485000	0.556351000	-0.965165000
C	-4.810236000	-0.321411000	-2.385341000
H	-5.527385000	-0.050838000	-3.168942000
H	-5.373078000	-0.801677000	-1.580312000
H	-4.121814000	-1.067486000	-2.792976000
C	-5.059073000	1.965606000	-1.389452000
H	-4.574033000	2.790498000	-0.857716000
H	-5.800973000	1.530999000	-0.709947000
H	-5.600546000	2.386765000	-2.245285000
Al	1.712460000	-0.401958000	0.028859000
N	2.305616000	-1.627999000	-1.397687000
N	3.252699000	0.789175000	-0.247985000
C	3.356044000	-1.475057000	-2.198986000
C	4.134646000	-0.300798000	-2.215921000
H	4.906275000	-0.248911000	-2.975904000
C	4.129410000	0.719893000	-1.265743000
C	3.818552000	-2.609271000	-3.069509000
H	2.996789000	-3.185425000	-3.497414000
H	4.403307000	-3.305808000	-2.456779000
H	4.465436000	-2.252214000	-3.872912000
C	5.191135000	1.776597000	-1.380361000

H	5.805847000	1.839853000	-0.477454000
H	4.726611000	2.762436000	-1.499857000
H	5.844056000	1.594661000	-2.235018000
C	1.510739000	-2.825118000	-1.523303000
C	1.708493000	-3.901328000	-0.638918000
C	0.957559000	-5.063614000	-0.836641000
H	1.091829000	-5.899306000	-0.152797000
C	0.046587000	-5.164232000	-1.876322000
H	-0.526102000	-6.076810000	-2.015163000
C	-0.165052000	-4.076472000	-2.715184000
H	-0.917913000	-4.144671000	-3.495805000
C	0.534501000	-2.881204000	-2.546939000
C	2.650923000	-3.826825000	0.544869000
H	3.147684000	-2.848390000	0.531896000
C	1.857763000	-3.922038000	1.849387000
H	1.377120000	-4.902516000	1.945250000
H	1.070453000	-3.160521000	1.891640000
H	2.515222000	-3.785435000	2.715434000
C	3.735930000	-4.899883000	0.483459000
H	4.418490000	-4.811776000	1.335541000
H	4.331243000	-4.828525000	-0.432866000
H	3.304560000	-5.906792000	0.515379000
C	0.203073000	-1.670685000	-3.405910000
H	0.475858000	-0.782861000	-2.817461000
C	0.976734000	-1.610183000	-4.723799000
H	2.047206000	-1.439019000	-4.578597000
H	0.598444000	-0.787886000	-5.342008000
H	0.857506000	-2.537795000	-5.296913000
C	-1.294288000	-1.565406000	-3.674230000
H	-1.514586000	-0.620318000	-4.176290000
H	-1.856527000	-1.592077000	-2.732157000
H	-1.660574000	-2.370392000	-4.322707000
C	3.679992000	1.497128000	0.925393000
C	3.277021000	2.821156000	1.169885000
C	3.718030000	3.452446000	2.337253000
H	3.406891000	4.477514000	2.533504000
C	4.552624000	2.801274000	3.235082000
H	4.894690000	3.309676000	4.132338000
C	4.949532000	1.493948000	2.977224000
H	5.605037000	0.982449000	3.679478000
C	4.517641000	0.813023000	1.837329000
C	2.408182000	3.582803000	0.193644000
H	2.223623000	2.920157000	-0.661360000
C	1.060111000	3.942461000	0.808733000
H	1.178279000	4.591683000	1.685083000
H	0.517474000	3.050749000	1.147958000
H	0.426352000	4.474246000	0.089762000
C	3.093732000	4.848655000	-0.319315000
H	2.465149000	5.355713000	-1.059702000
H	4.061692000	4.638122000	-0.784814000
H	3.273896000	5.559603000	0.494968000
C	4.989604000	-0.611211000	1.599745000
H	4.417960000	-1.023664000	0.760063000
C	4.723109000	-1.510685000	2.803244000
H	5.276024000	-1.183456000	3.691260000
H	5.036324000	-2.538029000	2.586430000
H	3.657244000	-1.525543000	3.047611000
C	6.469437000	-0.648765000	1.216447000
H	7.095679000	-0.232627000	2.014379000
H	6.673796000	-0.079720000	0.303934000
H	6.795607000	-1.679738000	1.042937000
H	2.042895000	-0.959202000	1.481758000
C	-2.190809000	-2.646737000	0.123854000
C	-3.026345000	-3.409493000	-0.666559000
O	-2.533216000	-2.931489000	1.448780000
C	-3.880537000	-4.175163000	0.178528000
H	-3.007015000	-3.421499000	-1.750471000
C	-3.539444000	-3.849854000	1.459440000
H	-4.645779000	-4.883909000	-0.112041000
C	-4.033195000	-4.282097000	2.784372000
H	-4.763490000	-5.087294000	2.675474000
H	-3.218992000	-4.643404000	3.423460000
H	-4.517255000	-3.461955000	3.332179000

Int-4'_M06L.log

SCF (M06L) = -2685.72499275
E(SCF)+ZPE(0 K)= -2684.506467
H(298 K)= -2684.440897
G(298 K)= -2684.605624
Lowest Frequency = 14.3991 cm-1

Pd	0.884915000	-0.366279000	-0.943108000
Al	-1.456629000	0.015438000	-0.711817000
N	-2.554901000	1.538596000	-1.182880000
N	-2.918759000	-1.239108000	-0.913437000
C	-3.685297000	1.458626000	-1.899779000
C	-4.308304000	0.241874000	-2.204214000
H	-5.192628000	0.296964000	-2.828393000
C	-4.001847000	-1.017667000	-1.669292000
C	-4.350911000	2.718889000	-2.365129000
H	-5.137638000	2.505121000	-3.089496000
H	-3.626242000	3.407873000	-2.809487000
H	-4.799607000	3.255036000	-1.521570000
C	-4.955057000	-2.142396000	-1.940703000
H	-4.460555000	-2.940140000	-2.504291000
H	-5.821883000	-1.800762000	-2.507127000
H	-5.298005000	-2.599807000	-1.006936000
C	-2.146079000	2.830576000	-0.701352000
C	-2.777102000	3.352337000	0.447783000
C	-2.395729000	4.616883000	0.899097000
H	-2.873123000	5.028270000	1.786686000
C	-1.412794000	5.348004000	0.243295000
H	-1.132318000	6.333412000	0.605243000
C	-0.775451000	4.801523000	-0.861636000
H	0.009431000	5.362883000	-1.364762000
C	-1.118124000	3.537214000	-1.352210000
C	-3.805713000	2.562264000	1.235778000
H	-4.068414000	1.662785000	0.664283000
C	-3.203975000	2.101116000	2.563940000
H	-2.302540000	1.493815000	2.412909000
H	-3.924908000	1.504084000	3.134273000
H	-2.920970000	2.960014000	3.183974000
C	-5.092213000	3.347182000	1.480746000
H	-4.915164000	4.223481000	2.113756000
H	-5.833587000	2.723414000	1.990708000
H	-5.540965000	3.704190000	0.548313000
C	-0.376113000	2.987124000	-2.553034000
H	-0.762653000	1.983822000	-2.769233000
C	1.115122000	2.844119000	-2.251642000
H	1.643448000	2.376541000	-3.087180000
H	1.277115000	2.199327000	-1.376965000
H	1.576707000	3.819135000	-2.051224000
C	-0.599837000	3.851277000	-3.793187000

H	-1.661478000	3.939079000	-4.048003000
H	-0.081984000	3.425013000	-4.657550000
H	-0.213369000	4.866366000	-3.645410000
C	-2.772927000	-2.517860000	-0.272149000
C	-2.232115000	-3.611555000	-0.972079000
C	-2.147934000	-4.840325000	-0.309395000
H	-1.728718000	-5.694005000	-0.837873000
C	-2.580574000	-4.985123000	0.999636000
H	-2.518548000	-5.951203000	1.493054000
C	-3.063079000	-3.879950000	1.692364000
H	-3.360488000	-3.990794000	2.731547000
C	-3.145828000	-2.625721000	1.085651000
C	-1.669692000	-3.490173000	-2.373751000
H	-1.904933000	-2.488573000	-2.755767000
C	-0.146570000	-3.619452000	-2.327401000
H	0.146492000	-4.625174000	-2.001574000
H	0.297494000	-2.891486000	-1.637846000
H	0.298033000	-3.435970000	-3.310231000
C	-2.257864000	-4.520355000	-3.336593000
H	-1.990193000	-5.540555000	-3.040015000
H	-1.867288000	-4.365582000	-4.346680000
H	-3.350979000	-4.475219000	-3.389653000
C	-3.603101000	-1.409550000	1.871692000
H	-2.992587000	-0.558330000	1.524621000
C	-3.363312000	-1.546696000	3.370842000
H	-4.034814000	-2.283607000	3.825151000
H	-3.550467000	-0.593293000	3.873611000
H	-2.334625000	-1.848942000	3.593405000
C	-5.066180000	-1.051245000	1.604800000
H	-5.247586000	-0.787652000	0.558743000
H	-5.370126000	-0.192850000	2.215486000
H	-5.723494000	-1.889875000	1.862655000
H	-0.271454000	-0.386105000	-2.197337000
C	2.432448000	-0.898453000	-2.253994000
C	3.140080000	-2.054033000	-2.489423000
O	2.915457000	0.055909000	-3.142619000
C	4.077223000	-1.804184000	-3.540955000
H	2.994910000	-2.988617000	-1.959415000
C	3.907282000	-0.503872000	-3.905930000
H	4.789056000	-2.495262000	-3.974621000
C	4.563787000	0.382589000	-4.889610000
H	5.329328000	-0.167539000	-5.441573000
H	3.853861000	0.791791000	-5.618802000
H	5.051081000	1.240539000	-4.408058000
P	2.177845000	-0.197079000	0.983127000
C	3.979189000	-0.409891000	0.578605000
C	4.490623000	0.797675000	-0.217500000
C	4.979873000	-0.836557000	1.656115000
H	3.925716000	-1.238612000	-0.145320000
C	5.846311000	0.491374000	-0.843294000
H	4.586025000	1.671030000	0.446420000
H	3.764446000	1.064368000	-0.995472000
C	6.336793000	-1.129935000	1.016435000
H	5.101677000	-0.054739000	2.419040000
H	4.623030000	-1.729992000	2.184268000
C	6.853637000	0.060661000	0.216803000
H	6.214826000	1.365147000	-1.393921000
H	5.715090000	-0.311373000	-1.583908000
H	7.060366000	-1.423915000	1.786020000
H	6.229745000	-1.993088000	0.343265000
H	7.820230000	-0.179228000	-0.241056000
H	7.038334000	0.901385000	0.903464000
C	1.836802000	-1.530592000	2.245003000
C	2.242255000	-1.215222000	3.689283000
C	0.390196000	-2.039842000	2.203218000
H	2.475875000	-2.357564000	1.888008000
C	2.064866000	-2.430183000	4.594308000
H	1.608585000	-0.397555000	4.064005000
H	3.273882000	-0.852243000	3.738785000
C	0.200848000	-3.240504000	3.124329000
H	-0.289246000	-1.222392000	2.501573000
H	0.108254000	-2.292984000	1.172283000
C	0.630279000	-2.938368000	4.554029000
H	2.362915000	-2.182719000	5.619771000
H	2.744358000	-3.228420000	4.259209000
H	-0.842909000	-3.572943000	3.093360000
H	0.796313000	-4.080820000	2.736558000
H	0.514690000	-3.824556000	5.188563000
H	-0.033848000	-2.167938000	4.975673000
C	2.035924000	1.496210000	1.767383000
C	0.621537000	1.812865000	2.262371000
C	3.067249000	1.913351000	2.825400000
H	2.210388000	2.140360000	0.887014000
C	0.481567000	3.289449000	2.612582000
H	0.395859000	1.198147000	3.149761000
H	-0.109701000	1.529945000	1.493633000
C	2.928340000	3.398593000	3.151002000
H	2.918709000	1.334389000	3.744918000
H	4.085500000	1.701924000	2.485922000
C	1.522205000	3.729692000	3.633720000
H	-0.533743000	3.498290000	2.972135000
H	0.599728000	3.878350000	1.690687000
H	3.674893000	3.689780000	3.899053000
H	3.150495000	3.985251000	2.246871000
H	1.428033000	4.801238000	3.844797000
H	1.338964000	3.209934000	4.586356000

Int-5_M06L.log

SCF (M06L) = -2879.90235353
E(SCF)+ZPE(0 K)= -2878.530988
H(298 K)= -2878.449569
G(298 K)= -2878.647894
Lowest Frequency = 14.5038 cm-1

Pd	-0.134906000	0.064891000	-0.017611000
Al	-2.392030000	-0.418220000	0.357318000
N	-3.272124000	-2.075924000	-0.042243000
N	-3.933376000	0.362884000	1.186403000
C	-4.505967000	-2.436372000	0.323241000
C	-5.361741000	-1.575008000	1.024107000
H	-6.343591000	-1.964343000	1.267593000
C	-5.114705000	-0.245696000	1.384765000
C	-5.021282000	-3.791608000	-0.056894000
H	-5.212636000	-3.844053000	-1.134493000
H	-5.949970000	-4.023725000	0.466023000
H	-4.280607000	-4.567779000	0.156532000
C	-6.240840000	0.530957000	1.997667000
H	-5.949482000	0.966475000	2.958410000
H	-7.121056000	-0.095539000	2.143987000
H	-6.515131000	1.377173000	1.358448000
C	-2.493732000	-2.935667000	-0.894937000
C	-2.754676000	-2.938921000	-2.280232000
C	-1.935380000	-3.718855000	-3.095778000
H	-2.118245000	-3.738697000	-4.167122000
C	-0.879529000	-4.453050000	-2.568266000
H	-0.242576000	-5.038961000	-3.226370000

C	-0.634062000	-4.425458000	-1.203775000
H	0.194379000	-4.997357000	-0.790738000
C	-1.428599000	-3.665619000	-0.340578000
C	-3.829629000	-2.056678000	-2.887840000
H	-4.600305000	-1.876172000	-2.126277000
C	-3.244638000	-0.695344000	-3.269355000
H	-2.735111000	-0.205589000	-2.429436000
H	-4.030099000	-0.022987000	-3.634936000
H	-2.486723000	-0.795678000	-4.055244000
C	-4.523444000	-2.695114000	-4.086924000
H	-3.848279000	-2.783063000	-4.944913000
H	-5.367107000	-2.078400000	-4.411171000
H	-4.902885000	-3.697238000	-3.860174000
C	-1.144451000	-3.682792000	1.148277000
H	-1.745210000	-2.895838000	1.622792000
C	0.318433000	-3.387046000	1.455278000
H	0.487351000	-3.347995000	2.537067000
H	0.627373000	-2.421707000	1.032165000
H	0.981919000	-4.160821000	1.054345000
C	-1.570195000	-5.016897000	1.763297000
H	-2.634800000	-5.221939000	1.606621000
H	-1.382573000	-5.027912000	2.841978000
H	-1.007569000	-5.846522000	1.319711000
C	-3.815725000	1.753466000	1.544543000
C	-3.399002000	2.104526000	2.842346000
C	-3.342282000	3.461232000	3.171902000
H	-3.030988000	3.746473000	4.175202000
C	-3.658650000	4.442908000	2.242693000
H	-3.607207000	5.492785000	2.518135000
C	-4.013250000	4.077243000	0.950624000
H	-4.223465000	4.846848000	0.210791000
C	-4.097654000	2.734871000	0.573647000
C	-2.980784000	1.075475000	3.873989000
H	-3.083040000	0.079681000	3.425140000
C	-1.510240000	1.260640000	4.250911000
H	-1.346618000	2.231662000	4.733963000
H	-0.861908000	1.201762000	3.370273000
H	-1.193485000	0.483459000	4.954619000
C	-3.862620000	1.124031000	5.120969000
H	-3.784941000	2.095130000	5.622913000
H	-3.558028000	0.358541000	5.841583000
H	-4.920088000	0.960376000	4.888704000
C	-4.419715000	2.383096000	-0.866546000
H	-4.623414000	1.304424000	-0.922176000
C	-3.203666000	2.669954000	-1.750051000
H	-2.932956000	3.732556000	-1.706171000
H	-3.403318000	2.415582000	-2.796347000
H	-2.326083000	2.090802000	-1.436706000
C	-5.658408000	3.106176000	-1.388667000
H	-6.536259000	2.929836000	-0.758364000
H	-5.897198000	2.767797000	-2.401822000
H	-5.502823000	4.189259000	-1.440216000
Al	2.247935000	0.478380000	0.482249000
N	3.254570000	2.063348000	-0.087172000
N	3.862979000	-0.317347000	1.276901000
C	4.477426000	2.431438000	0.315875000
C	5.303228000	1.602320000	1.085365000
H	6.277780000	2.000299000	1.344447000
C	5.047546000	0.283345000	1.472480000
C	5.029872000	3.764984000	-0.097086000
H	4.291266000	4.559477000	0.044867000
H	5.279773000	3.772467000	-1.163840000
H	5.933027000	4.006609000	0.465232000
C	6.165649000	-0.474431000	2.126879000
H	5.905418000	-0.760724000	3.151717000
H	7.080600000	0.118403000	2.154900000
H	6.365739000	-1.410422000	1.595635000
C	2.592693000	2.904984000	-1.049979000
C	2.996956000	2.824155000	-2.400435000
C	2.382014000	3.668377000	-3.323162000
H	2.677107000	3.620441000	-4.367892000
C	1.385581000	4.557244000	-2.933268000
H	0.920165000	5.208968000	-3.667823000
C	0.973129000	4.591476000	-1.610053000
H	0.179317000	5.272074000	-1.306921000
C	1.560384000	3.766208000	-0.644161000
C	4.026103000	1.800500000	-2.844239000
H	4.748228000	1.661274000	-2.027994000
C	3.350993000	0.449538000	-3.087971000
H	2.644828000	0.510706000	-3.923478000
H	2.759459000	0.118213000	-2.224474000
H	4.095333000	-0.323952000	-3.313916000
C	4.819377000	2.227450000	-4.073895000
H	5.625236000	1.514264000	-4.271639000
H	5.267737000	3.219298000	-3.951706000
H	4.192739000	2.254251000	-4.971666000
C	1.079937000	3.857545000	0.789871000
H	1.605517000	3.093331000	1.377757000
C	1.413803000	5.218534000	1.399645000
H	2.489606000	5.423798000	1.380541000
H	1.080379000	5.271925000	2.441183000
H	0.915991000	6.026914000	0.851673000
C	-0.413989000	3.562834000	0.893472000
H	-0.743028000	3.572039000	1.938469000
H	-0.651237000	2.573933000	0.472544000
H	-1.011811000	4.310122000	0.357792000
C	3.745808000	-1.697797000	1.666655000
C	3.503742000	-2.025550000	3.016298000
C	3.431245000	-3.374344000	3.367827000
H	3.244336000	-3.642511000	4.405203000
C	3.587357000	-4.376042000	2.417119000
H	3.533851000	-5.420733000	2.711577000
C	3.786124000	-4.034532000	1.087235000
H	3.876373000	-4.816721000	0.335569000
C	3.860203000	-2.698190000	0.683200000
C	3.258856000	-0.954097000	4.060440000
H	3.664883000	-0.008806000	3.679494000
C	1.754144000	-0.757605000	4.258377000
H	1.299587000	-1.667656000	4.669237000
H	1.241970000	-0.535096000	3.313833000
H	1.554513000	0.061898000	4.957605000
C	3.946230000	-1.244079000	5.391497000
H	3.828516000	-0.397828000	6.075140000
H	5.018433000	-1.432019000	5.269710000
H	3.515329000	-2.118783000	5.890297000
C	4.021494000	-2.380183000	-0.790255000
H	4.173466000	-1.297204000	-0.893265000
C	2.747466000	-2.749612000	-1.552914000
H	2.544937000	-3.824927000	-1.469886000
H	2.839994000	-2.506098000	-2.616839000
H	1.866406000	-2.214680000	-1.175866000
C	5.243077000	-3.065571000	-1.398264000
H	5.145776000	-4.156749000	-1.374604000
H	6.164839000	-2.805849000	-0.867470000
H	5.364301000	-2.774849000	-2.446709000
H	-0.886908000	-0.277048000	1.540032000
C	-0.014170000	0.270196000	-2.065622000
C	-0.132741000	1.308150000	-2.960776000
O	0.180522000	-0.888921000	-2.817496000
C	-0.002694000	0.783488000	-4.282124000
H	-0.276258000	2.345836000	-2.687286000
C	0.189486000	-0.559809000	-4.148112000
H	-0.031933000	1.329168000	-5.218085000
C	0.413819000	-1.663419000	-5.105868000
H	0.501145000	-1.269801000	-6.121825000
H	1.332944000	-2.220883000	-4.880663000
H	-0.402780000	-2.398813000	-5.101231000

Int-5’_M06L.log

SCF (M06L) = -2685.72227427

E(SCF)+ZPE(0 K)= -2684.502685
H(298 K)= -2684.437229
G(298 K)= -2684.602592
Lowest Frequency = 9.1280 cm-1

Pd	-0.368572000	0.355435000	0.272887000
Al	1.798616000	0.015844000	-0.466727000
N	2.651881000	-1.507265000	-1.248695000
N	3.324979000	1.145057000	-0.639542000
C	3.813588000	-1.497765000	-1.910740000
C	4.589081000	-0.338321000	-2.054428000
H	5.490491000	-0.431522000	-2.648868000
C	4.392542000	0.891048000	-1.410911000
C	4.323455000	-2.775232000	-2.501934000
H	4.414933000	-3.547164000	-1.730918000
H	5.293035000	-2.637325000	-2.980976000
H	3.618140000	-3.169559000	-3.241386000
C	5.452124000	1.939639000	-1.558304000
H	5.045933000	2.839934000	-2.031227000
H	6.289385000	1.578471000	-2.156142000
H	5.824033000	2.253793000	-0.577523000
C	1.966372000	-2.745031000	-0.985352000
C	2.137477000	-3.341652000	0.280028000
C	1.369007000	-4.466965000	0.586383000
H	1.485215000	-4.933372000	1.562540000
C	0.463555000	-4.990914000	-0.326134000
H	-0.133647000	-5.860652000	-0.066170000
C	0.322640000	-4.397398000	-1.573874000
H	-0.388809000	-4.807526000	-2.288484000
C	1.061934000	-3.266952000	-1.928753000
C	3.133578000	-2.824924000	1.302173000
H	3.622952000	-1.932520000	0.887366000
C	2.453073000	-2.412648000	2.606297000
H	1.716174000	-1.618603000	2.448366000
H	3.195463000	-2.050632000	3.326924000
H	1.932299000	-3.257967000	3.070553000
C	4.228402000	-3.860182000	1.562559000
H	3.810648000	-4.772019000	2.004100000
H	4.974069000	-3.468848000	2.262456000
H	4.748886000	-4.150357000	0.643765000
C	0.838135000	-2.629735000	-3.286185000
H	1.576034000	-1.827550000	-3.414599000
C	-0.549103000	-1.992212000	-3.357876000
H	-0.712679000	-1.504129000	-4.325264000
H	-0.674421000	-1.240710000	-2.571034000
H	-1.332267000	-2.750642000	-3.229460000
C	1.026256000	-3.631548000	-4.424696000
H	2.003768000	-4.124112000	-4.388116000
H	0.934833000	-3.135594000	-5.395980000
H	0.266260000	-4.419649000	-4.390354000
C	3.276811000	2.361285000	0.130682000
C	2.845837000	3.555998000	-0.472570000
C	2.799482000	4.711404000	0.312010000
H	2.464601000	5.643516000	-0.137992000
C	3.156110000	4.681656000	1.651545000
H	3.118132000	5.589804000	2.246680000
C	3.535173000	3.481102000	2.241787000
H	3.774278000	3.463383000	3.300401000
C	3.591925000	2.294775000	1.507187000
C	2.359567000	3.609241000	-1.907579000
H	2.632155000	2.666943000	-2.399251000
C	0.832661000	3.717826000	-1.942285000
H	0.502101000	4.662331000	-1.494020000
H	0.356547000	2.899035000	-1.390252000
H	0.466504000	3.689636000	-2.974638000
C	2.993452000	4.749452000	-2.701738000
H	2.688938000	5.727082000	-2.313085000
H	2.681285000	4.708931000	-3.749752000
H	4.087949000	4.717027000	-2.675171000
C	3.932577000	0.973721000	2.181172000
H	3.166576000	0.247313000	1.851788000
C	3.846695000	1.029797000	3.701714000
H	4.647771000	1.646185000	4.126689000
H	3.959399000	0.023842000	4.116270000
H	2.886401000	1.417596000	4.047841000
C	5.304979000	0.423400000	1.783756000
H	5.390388000	0.193864000	0.718968000
H	5.509100000	-0.504038000	2.330708000
H	6.096529000	1.136708000	2.042468000
H	0.243794000	0.503361000	-1.401091000
C	-0.207247000	0.052632000	2.287715000
C	0.311970000	0.784994000	3.330539000
O	-0.783887000	-1.086335000	2.841476000
C	0.023526000	0.097766000	4.550723000
H	0.818539000	1.736344000	3.217383000
C	-0.643181000	-1.038393000	4.205662000
H	0.277084000	0.400370000	5.559315000
C	-1.226928000	-2.161932000	4.967595000
H	-1.048040000	-2.027272000	6.036888000
H	-2.312100000	-2.241079000	4.817816000
H	-0.798708000	-3.128988000	4.674453000
P	-2.772439000	0.660945000	-0.095922000
C	-3.137445000	1.936041000	-1.418731000
C	-2.552724000	1.506244000	-2.769290000
C	-4.577111000	2.437710000	-1.580577000
H	-2.539498000	2.799831000	-1.079159000
C	-2.648715000	2.625586000	-3.799715000
H	-3.099691000	0.627300000	-3.145582000
H	-1.510545000	1.187421000	-2.634653000
C	-4.656630000	3.552743000	-2.620890000
H	-5.232709000	1.612066000	-1.890043000
H	-4.969602000	2.798778000	-0.622550000
C	-4.084135000	3.109836000	-3.961282000
H	-2.239905000	2.291108000	-4.760643000
H	-2.021410000	3.468215000	-3.472540000
H	-5.694042000	3.888809000	-2.732870000
H	-4.088409000	4.421991000	-2.257519000
H	-4.139403000	3.924702000	-4.692285000
H	-4.699085000	2.290756000	-4.363933000
C	-3.942553000	1.129001000	1.291933000
C	-5.392470000	0.643571000	1.179387000
C	-3.375991000	0.740216000	2.662078000
H	-3.951416000	2.232595000	1.244916000
C	-6.252749000	1.204503000	2.308443000
H	-5.398883000	-0.454548000	1.246452000
H	-5.828409000	0.895596000	0.207187000
C	-4.240935000	1.263558000	3.802200000
H	-3.304882000	-0.355524000	2.723563000
H	-2.350274000	1.104009000	2.764872000
C	-5.691592000	0.818700000	3.671136000
H	-7.287549000	0.857958000	2.200801000
H	-6.284612000	2.301694000	2.223389000
H	-3.816839000	0.940834000	4.760727000
H	-4.202764000	2.363608000	3.805147000
H	-6.304808000	1.241002000	4.475860000
H	-5.745945000	-0.274742000	3.781904000
C	-3.292277000	-0.981425000	-0.815052000
C	-3.041228000	-2.106090000	0.200317000
C	-4.646263000	-1.160024000	-1.511765000
H	-2.526331000	-1.104133000	-1.598028000
C	-3.157820000	-3.475070000	-0.460764000
H	-3.773015000	-2.029881000	1.020609000
H	-2.051595000	-1.983371000	0.661767000
C	-4.717698000	-2.529050000	-2.184587000
H	-5.466420000	-1.078683000	-0.789204000
H	-4.808396000	-0.371433000	-2.255536000
C	-4.491027000	-3.649690000	-1.177047000
H	-3.005036000	-4.267766000	0.281308000
H	-2.339811000	-3.579141000	-1.189947000
H	-5.681655000	-2.651690000	-2.692457000
H	-3.945336000	-2.583242000	-2.968069000

H	-4.540594000	-4.627762000	-1.670215000
H	-5.306061000	-3.635731000	-0.437701000

Int-6_M06L.log

SCF (M06L) = -2879.93145710
E(SCF)+ZPE(0 K)= -2878.558688
H(298 K)= -2878.477616
G(298 K)= -2878.674443
Lowest Frequency = 18.0944 cm-1

Pd	0.210373000	0.113268000	0.591983000
Al	-2.346259000	-0.346059000	0.563470000
N	-3.515484000	-1.837299000	0.289711000
N	-3.682518000	0.777169000	1.358841000
C	-4.635453000	-2.025785000	0.992894000
C	-5.165771000	-1.055255000	1.855656000
H	-6.051006000	-1.339609000	2.412785000
C	-4.762269000	0.284552000	1.974869000
C	-5.391379000	-3.307705000	0.822511000
H	-5.876487000	-3.339085000	-0.159532000
H	-6.163275000	-3.418434000	1.584933000
H	-4.718118000	-4.169610000	0.858535000
C	-5.597685000	1.184599000	2.835544000
H	-5.101548000	1.344369000	3.799329000
H	-6.578245000	0.748336000	3.030262000
H	-5.726342000	2.172150000	2.384765000
C	-3.138016000	-2.757734000	-0.744305000
C	-3.732017000	-2.640184000	-2.016804000
C	-3.245352000	-3.448670000	-3.044825000
H	-3.685202000	-3.362098000	-4.037270000
C	-2.199571000	-4.338711000	-2.830776000
H	-1.826903000	-4.948895000	-3.649395000
C	-1.638192000	-4.449377000	-1.565932000
H	-0.827857000	-5.155238000	-1.391887000
C	-2.095582000	-3.670121000	-0.500090000
C	-4.846086000	-1.652742000	-2.315316000
H	-5.145449000	-1.170109000	-1.375484000
C	-4.381890000	-0.549078000	-3.267546000
H	-3.565819000	0.041664000	-2.842678000
H	-5.211672000	0.127650000	-3.502320000
H	-4.026559000	-0.974274000	-4.213902000
C	-6.075935000	-2.354692000	-2.892348000
H	-5.861974000	-2.784362000	-3.877351000
H	-6.902005000	-1.647306000	-3.018547000
H	-6.426668000	-3.172117000	-2.253656000
C	-1.481328000	-3.847703000	0.874979000
H	-1.951830000	-3.127679000	1.555793000
C	0.017487000	-3.566466000	0.871765000
H	0.433446000	-3.654383000	1.881767000
H	0.244215000	-2.550069000	0.517651000
H	0.555415000	-4.277301000	0.233195000
C	-1.761941000	-5.251011000	1.414399000
H	-2.834076000	-5.472074000	1.454672000
H	-1.355831000	-5.366138000	2.424429000
H	-1.294778000	-6.015489000	0.782631000
C	-3.383636000	2.178876000	1.420122000
C	-2.709012000	2.716559000	2.533642000
C	-2.420785000	4.084323000	2.526084000
H	-1.897891000	4.516283000	3.377634000
C	-2.768495000	4.889129000	1.451077000
H	-2.529658000	5.949443000	1.464833000
C	-3.405325000	4.333596000	0.346580000
H	-3.659500000	4.966484000	-0.499228000
C	-3.723367000	2.974807000	0.303556000
C	-2.272677000	1.875759000	3.718560000
H	-2.652371000	0.856018000	3.575193000
C	-0.748672000	1.787555000	3.805071000
H	-0.313951000	2.777013000	3.993800000
H	-0.303959000	1.389922000	2.881949000
H	-0.449610000	1.129290000	4.627894000
C	-2.831772000	2.419056000	5.034714000
H	-2.401064000	3.398844000	5.269997000
H	-2.583943000	1.749976000	5.864610000
H	-3.919331000	2.544076000	5.013394000
C	-4.441514000	2.374348000	-0.892319000
H	-4.049421000	1.357047000	-1.026355000
C	-4.181516000	3.126824000	-2.191091000
H	-4.647724000	4.118850000	-2.193117000
H	-4.604458000	2.573171000	-3.034956000
H	-3.110500000	3.249564000	-2.378391000
C	-5.946685000	2.253331000	-0.644665000
H	-6.177281000	1.583452000	0.188828000
H	-6.450518000	1.853434000	-1.531820000
H	-6.389171000	3.232025000	-0.423896000
Al	2.541772000	0.361133000	0.414547000
N	3.724424000	1.722154000	-0.395690000
N	4.103195000	-0.750690000	0.858888000
C	5.048031000	1.842468000	-0.231329000
C	5.818876000	0.878952000	0.430248000
H	6.877744000	1.090436000	0.527771000
C	5.388817000	-0.376562000	0.878762000
C	5.764701000	3.027083000	-0.811320000
H	5.225602000	3.956983000	-0.607208000
H	5.827852000	2.946197000	-1.902584000
H	6.780003000	3.104719000	-0.419350000
C	6.430276000	-1.327137000	1.392107000
H	6.241890000	-1.579215000	2.441580000
H	7.431595000	-0.902613000	1.309989000
H	6.400964000	-2.276143000	0.847490000
C	3.061029000	2.647098000	-1.271085000
C	3.176105000	2.465364000	-2.665744000
C	2.505284000	3.350600000	-3.509941000
H	2.582927000	3.219457000	-4.587005000
C	1.727271000	4.383228000	-2.998521000
H	1.212044000	5.063768000	-3.671106000
C	1.607066000	4.533534000	-1.625001000
H	0.994338000	5.337271000	-1.221269000
C	2.263970000	3.674989000	-0.737504000
C	3.926872000	1.284606000	-3.251278000
H	4.639985000	0.916314000	-2.503279000
C	2.940269000	0.151715000	-3.534680000
H	2.217286000	0.458457000	-4.299725000
H	2.359745000	-0.116633000	-2.641208000
H	3.457825000	-0.745138000	-3.895792000
C	4.724077000	1.634346000	-4.503809000
H	5.327490000	0.779519000	-4.824149000
H	5.399353000	2.480209000	-4.337266000
H	4.071880000	1.895881000	-5.343888000
C	2.122199000	3.911785000	0.751652000
H	2.629691000	3.093789000	1.279159000
C	2.799630000	5.219948000	1.160836000
H	3.861780000	5.232163000	0.893776000
H	2.723783000	5.377515000	2.241580000
H	2.328112000	6.077783000	0.667394000
C	0.659901000	3.895720000	1.182938000
H	0.571529000	4.079190000	2.259116000
H	0.196076000	2.921604000	0.969545000
H	0.075482000	4.669616000	0.671893000
C	3.789466000	-2.107155000	1.217876000
C	3.621504000	-2.462043000	2.570560000
C	3.362212000	-3.799423000	2.877204000
H	3.230540000	-4.085810000	3.918623000
C	3.261621000	-4.762325000	1.881144000
H	3.068130000	-5.799715000	2.140577000
C	3.376570000	-4.384473000	0.550722000
H	3.258805000	-5.129569000	-0.234465000
C	3.623146000	-3.056805000	0.191500000
C	3.644639000	-1.431914000	3.681803000
H	4.043811000	-0.496084000	3.270637000

C	2.216237000	-1.156332000	4.158836000
H	1.773863000	-2.063925000	4.587711000
H	1.573113000	-0.828244000	3.332011000
H	2.206553000	-0.380496000	4.932605000
C	4.535570000	-1.843329000	4.851562000
H	4.586318000	-1.043037000	5.596318000
H	5.558868000	-2.072046000	4.534118000
H	4.146766000	-2.731713000	5.361148000
C	3.679896000	-2.691371000	-1.279965000
H	3.881150000	-1.614480000	-1.359619000
C	2.334292000	-2.960143000	-1.956286000
H	2.045912000	-4.014125000	-1.863563000
H	2.386423000	-2.726119000	-3.025477000
H	1.531440000	-2.355506000	-1.513564000
C	4.811826000	-3.420419000	-2.001319000
H	4.665949000	-4.506379000	-1.977647000
H	5.785459000	-3.209850000	-1.546973000
H	4.860526000	-3.119933000	-3.053413000
H	-1.180462000	-0.652997000	1.722273000
C	-1.548072000	0.271217000	-1.145228000
C	-1.040084000	1.470092000	-1.618292000
O	-1.294612000	-0.687292000	-2.135050000
C	-0.506817000	1.247864000	-2.919716000
H	-1.083715000	2.415544000	-1.087353000
C	-0.682472000	-0.079124000	-3.184689000
H	-0.024016000	1.970759000	-3.564474000
C	-0.347232000	-0.950047000	-4.330694000
H	0.045494000	-0.358585000	-5.160730000
H	0.406981000	-1.700745000	-4.060463000
H	-1.226672000	-1.500689000	-4.684266000

Int-6b_M06L.log

SCF (M06L) = -2879.93013705
E(SCF)+ZPE(0 K)= -2878.554117
H(298 K)= -2878.474577
G(298 K)= -2878.66355
Lowest Frequency = 21.2752 cm-1

Pd	0.032573000	-1.278387000	-0.288456000
Al	1.722911000	0.336357000	0.109555000
N	2.399765000	2.061062000	-0.414655000
N	2.536460000	0.366244000	1.861781000
C	2.980159000	2.933866000	0.418780000
C	3.166765000	2.684833000	1.783744000
H	3.568505000	3.505104000	2.369726000
C	3.016135000	1.463270000	2.454974000
C	3.520246000	4.233932000	-0.103408000
H	4.604988000	4.160717000	-0.237773000
H	3.342815000	5.033116000	0.620778000
H	3.084509000	4.515949000	-1.063041000
C	3.422353000	1.446617000	3.902058000
H	2.649860000	1.944538000	4.499764000
H	4.346140000	2.010143000	4.052672000
H	3.554351000	0.437752000	4.295616000
C	2.474928000	2.262350000	-1.836239000
C	3.680408000	1.975664000	-2.506760000
C	3.687157000	2.046883000	-3.902064000
H	4.607395000	1.818317000	-4.437067000
C	2.544801000	2.386285000	-4.609872000
H	2.566954000	2.425807000	-5.695774000
C	1.370218000	2.685633000	-3.928338000
H	0.485063000	2.961673000	-4.492497000
C	1.305344000	2.635181000	-2.534291000
C	4.969853000	1.601778000	-1.794921000
H	4.801445000	1.640373000	-0.710337000
C	5.434148000	0.183555000	-2.131847000
H	4.732108000	-0.572915000	-1.773570000
H	6.412627000	-0.011398000	-1.677596000
H	5.542829000	0.052905000	-3.215138000
C	6.083065000	2.596591000	-2.134484000
H	6.379894000	2.508095000	-3.185523000
H	6.974342000	2.403768000	-1.528216000
H	5.778391000	3.635375000	-1.973752000
C	0.030068000	3.004598000	-1.793376000
H	-0.160986000	2.213708000	-1.047832000
C	-1.196738000	3.079515000	-2.691519000
H	-2.087332000	3.249679000	-2.078127000
H	-1.357143000	2.152245000	-3.249709000
H	-1.120898000	3.909999000	-3.404687000
C	0.168378000	4.326611000	-1.033341000
H	0.918968000	4.281738000	-0.241132000
H	-0.785372000	4.587734000	-0.561531000
H	0.441126000	5.141658000	-1.715003000
C	2.602677000	-0.911186000	2.516492000
C	1.537974000	-1.362880000	3.322720000
C	1.664144000	-2.615317000	3.931340000
H	0.860307000	-2.971085000	4.572969000
C	2.779278000	-3.413097000	3.721553000
H	2.852563000	-4.386094000	4.200175000
C	3.798413000	-2.969629000	2.886586000
H	4.660709000	-3.606860000	2.711355000
C	3.736288000	-1.718713000	2.267813000
C	0.293384000	-0.536005000	3.587544000
H	0.300579000	0.310189000	2.888379000
C	-0.994573000	-1.314424000	3.329514000
H	-1.067933000	-2.213873000	3.953098000
H	-1.065023000	-1.608843000	2.275690000
H	-1.867426000	-0.690200000	3.555575000
C	0.279829000	0.006008000	5.018053000
H	0.239694000	-0.814201000	5.744644000
H	-0.605678000	0.631401000	5.181530000
H	1.164962000	0.604207000	5.252804000
C	4.867890000	-1.243756000	1.369852000
H	4.417902000	-0.629376000	0.578025000
C	5.608570000	-2.385369000	0.682535000
H	6.193093000	-2.982400000	1.391801000
H	6.313673000	-1.983325000	-0.051128000
H	4.923534000	-3.053478000	0.152596000
C	5.867526000	-0.364400000	2.124175000
H	5.412291000	0.551682000	2.508215000
H	6.688581000	-0.066908000	1.462015000
H	6.303421000	-0.904561000	2.972972000
Al	-2.011464000	0.014081000	-0.116634000
N	-3.146328000	-1.660047000	-0.124928000
N	-3.190291000	0.848252000	1.217105000
C	-4.037036000	-2.003745000	0.792520000
C	-4.426597000	-1.133130000	1.834922000
H	-5.147487000	-1.533233000	2.540571000
C	-4.089254000	0.212526000	1.986829000
C	-4.717247000	-3.342852000	0.750749000
H	-4.427648000	-3.956648000	1.610178000
H	-4.478260000	-3.894697000	-0.160755000
H	-5.801866000	-3.212202000	0.806535000
C	-4.779436000	0.986826000	3.074377000
H	-4.047933000	1.294219000	3.832618000
H	-5.553475000	0.393612000	3.563504000
H	-5.230344000	1.908577000	2.694509000
C	-2.594471000	-2.564811000	-1.087389000
C	-2.820326000	-2.287338000	-2.456041000
C	-2.074238000	-2.981512000	-3.410046000
H	-2.229427000	-2.767223000	-4.463588000
C	-1.122539000	-3.923564000	-3.037669000
H	-0.525224000	-4.427955000	-3.792879000
C	-0.947025000	-4.225602000	-1.695408000
H	-0.224727000	-4.985882000	-1.404061000
C	-1.677874000	-3.575583000	-0.692369000
C	-3.883929000	-1.285854000	-2.863527000
H	-3.825116000	-0.445273000	-2.162400000
C	-3.677345000	-0.709251000	-4.256481000

H	-3.811804000	-1.461107000	-5.042882000
H	-2.675751000	-0.279693000	-4.364270000
H	-4.404923000	0.085707000	-4.446836000
C	-5.280073000	-1.893722000	-2.721651000
H	-6.051343000	-1.162856000	-2.990984000
H	-5.474685000	-2.215224000	-1.693189000
H	-5.403402000	-2.765605000	-3.374949000
C	-1.525027000	-4.070950000	0.737986000
H	-2.093300000	-3.406444000	1.400884000
C	-2.092388000	-5.490100000	0.859581000
H	-3.111266000	-5.573460000	0.472736000
H	-2.096008000	-5.820375000	1.903888000
H	-1.472642000	-6.195585000	0.294448000
C	-0.081714000	-4.075431000	1.235853000
H	-0.034065000	-4.452789000	2.263533000
H	0.358453000	-3.067336000	1.238124000
H	0.558024000	-4.716922000	0.618280000
C	-3.305242000	2.267193000	1.063044000
C	-2.513084000	3.153334000	1.811810000
C	-2.630250000	4.525997000	1.568783000
H	-2.015079000	5.218511000	2.141163000
C	-3.512068000	5.015035000	0.615617000
H	-3.593002000	6.084751000	0.442522000
C	-4.291867000	4.126805000	-0.117530000
H	-4.986751000	4.510865000	-0.861643000
C	-4.201894000	2.747954000	0.079050000
C	-1.544704000	2.667044000	2.868171000
H	-1.676446000	1.580361000	2.967689000
C	-0.101031000	2.923095000	2.444039000
H	0.085920000	3.991984000	2.280965000
H	0.140762000	2.400580000	1.507739000
H	0.600108000	2.579210000	3.213113000
C	-1.819234000	3.310814000	4.226622000
H	-1.144895000	2.910789000	4.991570000
H	-2.848619000	3.142140000	4.559071000
H	-1.662714000	4.394791000	4.192669000
C	-5.091644000	1.814754000	-0.723718000
H	-4.713953000	0.793044000	-0.595507000
C	-5.055111000	2.119049000	-2.218034000
H	-5.438625000	3.119816000	-2.446920000
H	-5.679340000	1.403582000	-2.765683000
H	-4.033816000	2.044277000	-2.605478000
C	-6.528501000	1.840873000	-0.200778000
H	-6.965464000	2.842333000	-0.292768000
H	-6.585569000	1.550031000	0.853143000
H	-7.159133000	1.148455000	-0.769425000
H	-2.382271000	0.899647000	-1.392013000
C	2.379551000	-1.139865000	-1.095038000
C	2.440585000	-2.517216000	-0.922521000
O	2.370034000	-0.917704000	-2.474210000
C	2.508087000	-3.128541000	-2.210056000
H	2.493716000	-3.016289000	0.039201000
C	2.451480000	-2.113621000	-3.118118000
H	2.570561000	-4.184230000	-2.440004000
C	2.440187000	-2.058068000	-4.594248000
H	2.541682000	-3.059968000	-5.016560000
H	1.505373000	-1.622295000	-4.966614000
H	3.256650000	-1.436804000	-4.980280000

Int-6'_M06L.log

SCF (M06L) = -2685.76806044
E(SCF)+ZPE(0 K)= -2684.547594
H(298 K)= -2684.482074
G(298 K)= -2684.646939
Lowest Frequency = 13.4550 cm-1

Pd	0.962983000	-0.516545000	-1.278133000
Al	-1.417844000	-0.057864000	-0.606304000
N	-2.074942000	1.712841000	-1.118146000
N	-2.999667000	-0.986191000	-1.206899000
C	-2.821475000	1.824175000	-2.215621000
C	-3.402820000	0.712053000	-2.862497000
H	-3.912747000	0.926921000	-3.795674000
C	-3.580235000	-0.575414000	-2.345813000
C	-3.111736000	3.166735000	-2.822678000
H	-2.876966000	3.988987000	-2.144305000
H	-4.162207000	3.235361000	-3.115351000
H	-2.517297000	3.301733000	-3.732899000
C	-4.472443000	-1.522519000	-3.093967000
H	-3.907635000	-2.416508000	-3.381942000
H	-4.882260000	-1.060903000	-3.993078000
H	-5.300001000	-1.877485000	-2.473074000
C	-1.505707000	2.855121000	-0.462667000
C	-2.019761000	3.224168000	0.800751000
C	-1.380192000	4.244995000	1.504511000
H	-1.757182000	4.534702000	2.481177000
C	-0.271745000	4.899035000	0.979258000
H	0.217203000	5.687532000	1.545615000
C	0.200946000	4.545634000	-0.274547000
H	1.060362000	5.066373000	-0.693504000
C	-0.395095000	3.522664000	-1.019419000
C	-3.271063000	2.566413000	1.348485000
H	-3.202795000	1.492748000	1.127643000
C	-3.425260000	2.706179000	2.857220000
H	-2.534227000	2.358213000	3.388322000
H	-4.272325000	2.108419000	3.205675000
H	-3.619800000	3.743122000	3.155539000
C	-4.514172000	3.106815000	0.637053000
H	-4.598255000	4.192101000	0.767478000
H	-5.423767000	2.651652000	1.045351000
H	-4.496625000	2.897879000	-0.437290000
C	0.177973000	3.209415000	-2.389458000
H	-0.403300000	2.392999000	-2.836552000
C	1.621932000	2.723406000	-2.292482000
H	2.028376000	2.509053000	-3.287335000
H	1.673232000	1.796639000	-1.702779000
H	2.267502000	3.476695000	-1.821677000
C	0.102586000	4.430381000	-3.309232000
H	-0.904307000	4.854470000	-3.363690000
H	0.418014000	4.170329000	-4.324937000
H	0.768890000	5.225685000	-2.956514000
C	-3.544421000	-2.126861000	-0.537041000
C	-2.938478000	-3.389897000	-0.667196000
C	-3.508590000	-4.472966000	0.007552000
H	-3.044905000	-5.453406000	-0.081869000
C	-4.649202000	-4.318592000	0.784105000
H	-5.084906000	-5.174903000	1.291700000
C	-5.220626000	-3.059286000	0.923204000
H	-6.101515000	-2.934426000	1.549867000
C	-4.676622000	-1.942581000	0.285345000
C	-1.657671000	-3.584715000	-1.451553000
H	-1.457550000	-2.665107000	-2.017115000
C	-0.493251000	-3.785081000	-0.479522000
H	-0.597837000	-4.731703000	0.066049000
H	-0.459746000	-2.977461000	0.260559000
H	0.468852000	-3.789747000	-1.005722000
C	-1.742152000	-4.737007000	-2.447859000
H	-1.878957000	-5.699672000	-1.942351000
H	-0.819526000	-4.806671000	-3.032311000
H	-2.575275000	-4.613462000	-3.147229000
C	-5.290526000	-0.573093000	0.511807000
H	-4.601410000	0.172346000	0.095965000
C	-5.447348000	-0.272383000	2.000965000
H	-6.138061000	-0.970248000	2.487133000
H	-5.854877000	0.734351000	2.146384000
H	-4.485440000	-0.329281000	2.521404000
C	-6.631023000	-0.419111000	-0.206513000
H	-6.534481000	-0.534957000	-1.290332000

H	-7.059950000	0.571199000	-0.018786000
H	-7.354087000	-1.165030000	0.144126000
H	-0.500080000	-0.379201000	-2.128611000
C	-1.365302000	-0.261873000	1.364218000
C	-1.858331000	-1.139783000	2.302123000
O	-0.593386000	0.671782000	2.050353000
C	-1.395050000	-0.727260000	3.585405000
H	-2.493844000	-1.992376000	2.092411000
C	-0.624300000	0.379616000	3.378737000
H	-1.600892000	-1.188156000	4.542416000
C	0.170588000	1.262185000	4.259673000
H	-0.087968000	1.097014000	5.307631000
H	1.250053000	1.084868000	4.150212000
H	-0.000225000	2.318383000	4.020945000
P	2.996277000	-0.657695000	-0.136946000
C	4.276680000	-1.392013000	-1.281399000
C	4.578551000	-0.408972000	-2.418964000
C	5.560415000	-1.981548000	-0.689515000
H	3.724057000	-2.234357000	-1.731420000
C	5.455696000	-1.044462000	-3.491267000
H	5.094164000	0.474801000	-2.012881000
H	3.635014000	-0.043441000	-2.849098000
C	6.425941000	-2.606777000	-1.781367000
H	6.137979000	-1.202352000	-0.173999000
H	5.316804000	-2.736083000	0.068396000
C	6.737486000	-1.608414000	-2.889633000
H	5.683688000	-0.314072000	-4.275854000
H	4.896680000	-1.856997000	-3.977810000
H	7.349858000	-3.005419000	-1.346846000
H	5.892111000	-3.466540000	-2.212677000
H	7.355238000	-2.075281000	-3.665042000
H	7.335159000	-0.782863000	-2.474337000
C	3.020681000	-1.775542000	1.357214000
C	4.125683000	-1.588076000	2.402119000
C	1.661977000	-1.773850000	2.062685000
H	3.149349000	-2.773576000	0.898909000
C	4.060168000	-2.685426000	3.462989000
H	3.981414000	-0.613039000	2.892339000
H	5.120396000	-1.559907000	1.945032000
C	1.578196000	-2.872018000	3.112309000
H	1.515149000	-0.798056000	2.546381000
H	0.854257000	-1.857530000	1.329654000
C	2.694674000	-2.731593000	4.139776000
H	4.855250000	-2.539805000	4.203714000
H	4.260867000	-3.655440000	2.982974000
H	0.594615000	-2.846105000	3.595405000
H	1.657033000	-3.851658000	2.615320000
H	2.657924000	-3.543344000	4.875468000
H	2.541665000	-1.797274000	4.702399000
C	3.626537000	1.035082000	0.337416000
C	2.641102000	1.692873000	1.313511000
C	5.077034000	1.221227000	0.796574000
H	3.531510000	1.579401000	-0.616649000
C	2.985531000	3.155356000	1.565787000
H	2.664574000	1.146318000	2.269985000
H	1.613047000	1.596616000	0.935805000
C	5.387638000	2.700390000	1.015390000
H	5.258566000	0.677965000	1.731506000
H	5.769793000	0.804439000	0.056403000
C	4.428827000	3.324520000	2.021282000
H	2.287087000	3.582313000	2.295895000
H	2.829719000	3.720993000	0.634359000
H	6.427655000	2.822068000	1.339844000
H	5.298415000	3.231823000	0.055338000
H	4.665847000	4.383683000	2.174544000
H	4.562354000	2.833596000	2.997438000

Int-7_M06L.log

SCF (M06L) = -2879.88558798

E(SCF)+ZPE(0 K)= -2878.513648
H(298 K)= -2878.432858
G(298 K)= -2878.627298
Lowest Frequency = 12.5040 cm-1

Pd	-0.442052000	-0.085112000	0.757339000
Al	1.867527000	-0.215923000	0.061048000
N	2.600345000	-1.565695000	-1.102064000
N	3.171351000	1.120668000	-0.449219000
C	2.674708000	-1.125594000	-2.364025000
C	2.783205000	0.247208000	-2.662538000
H	2.721319000	0.513716000	-3.713127000
C	3.159295000	1.278409000	-1.787868000
C	2.600481000	-2.079861000	-3.516911000
H	2.987097000	-3.068383000	-3.259850000
H	3.136645000	-1.697164000	-4.387254000
H	1.549461000	-2.207281000	-3.804869000
C	3.485936000	2.603659000	-2.414001000
H	4.159629000	2.494754000	-3.267369000
H	3.914319000	3.312980000	-1.705513000
H	2.551755000	3.034493000	-2.796140000
C	2.682695000	-2.965777000	-0.798848000
C	3.941228000	-3.496054000	-0.438298000
C	4.017159000	-4.849787000	-0.106560000
H	4.975196000	-5.278929000	0.171380000
C	2.887402000	-5.659620000	-0.118960000
H	2.967637000	-6.709906000	0.147513000
C	1.660961000	-5.119439000	-0.469831000
H	0.774559000	-5.750378000	-0.481523000
C	1.529178000	-3.770436000	-0.815934000
C	5.190428000	-2.633528000	-0.451126000
H	4.901291000	-1.632873000	-0.091562000
C	6.293871000	-3.172368000	0.454012000
H	5.931043000	-3.400815000	1.461157000
H	7.107042000	-2.446128000	0.540819000
H	6.732706000	-4.090188000	0.047441000
C	5.740180000	-2.462165000	-1.870374000
H	5.944864000	-3.438434000	-2.324991000
H	6.679947000	-1.898843000	-1.855839000
H	5.049390000	-1.924665000	-2.524874000
C	0.161848000	-3.261557000	-1.225174000
H	0.235128000	-2.190983000	-1.465228000
C	-0.846616000	-3.403593000	-0.084730000
H	-1.825350000	-3.005169000	-0.373398000
H	-0.523904000	-2.855595000	0.808502000
H	-0.990152000	-4.457110000	0.186870000
C	-0.327240000	-4.002001000	-2.472913000
H	0.433242000	-4.037296000	-3.259710000
H	-1.218104000	-3.521545000	-2.886879000
H	-0.593545000	-5.038925000	-2.235591000
C	3.932558000	2.005812000	0.374451000
C	3.274484000	2.754034000	1.378464000
C	4.035792000	3.583113000	2.202154000
H	3.535905000	4.158422000	2.975852000
C	5.411820000	3.695408000	2.042638000
H	5.985685000	4.356698000	2.685959000
C	6.047071000	2.948220000	1.063300000
H	7.127306000	3.016983000	0.949539000
C	5.335864000	2.081517000	0.226350000
C	1.774676000	2.680823000	1.524949000
H	1.471032000	1.624920000	1.449763000
C	1.249562000	3.136967000	2.877696000
H	1.327948000	4.224033000	3.005288000
H	1.781916000	2.653486000	3.702750000
H	0.192897000	2.867164000	2.964165000
C	1.054661000	3.399408000	0.386983000
H	1.175064000	4.487774000	0.464593000
H	-0.014182000	3.161914000	0.411114000
H	1.420007000	3.089098000	-0.596015000
C	6.132332000	1.228723000	-0.746574000

H	5.446055000	0.559443000	-1.280788000
C	7.132949000	0.353833000	0.009866000
H	7.870407000	0.965843000	0.540447000
H	7.684673000	-0.296378000	-0.678051000
H	6.637056000	-0.278998000	0.751480000
C	6.877173000	2.074851000	-1.779577000
H	6.211713000	2.745895000	-2.328379000
H	7.389441000	1.436048000	-2.506820000
H	7.639684000	2.696606000	-1.296790000
Al	-2.771899000	0.518447000	0.455382000
N	-4.542362000	-0.385339000	0.522587000
N	-3.606238000	2.013046000	-0.590118000
C	-5.745523000	0.161403000	0.306880000
C	-5.913958000	1.436305000	-0.245884000
H	-6.936235000	1.770277000	-0.384503000
C	-4.909445000	2.267931000	-0.758905000
C	-6.987010000	-0.629938000	0.602799000
H	-7.864064000	0.018250000	0.643953000
H	-6.895456000	-1.176368000	1.545909000
H	-7.163531000	-1.384550000	-0.172068000
C	-5.349641000	3.472558000	-1.543769000
H	-5.145017000	4.402050000	-1.002012000
H	-6.419148000	3.432655000	-1.755519000
H	-4.802915000	3.545940000	-2.488836000
C	-4.481877000	-1.791753000	0.798621000
C	-4.693450000	-2.698480000	-0.263385000
C	-4.586050000	-4.065569000	-0.004114000
H	-4.734773000	-4.772770000	-0.817632000
C	-4.274689000	-4.535175000	1.265951000
H	-4.194304000	-5.603189000	1.449389000
C	-4.059325000	-3.631011000	2.295685000
H	-3.814004000	-3.995496000	3.291085000
C	-4.154013000	-2.251552000	2.088415000
C	-4.947531000	-2.232702000	-1.686489000
H	-5.213065000	-1.168851000	-1.671884000
C	-3.663800000	-2.371400000	-2.503259000
H	-3.357557000	-3.423213000	-2.548638000
H	-2.836150000	-1.808551000	-2.051651000
H	-3.799897000	-2.015825000	-3.531170000
C	-6.092120000	-2.983647000	-2.362443000
H	-6.303041000	-2.556801000	-3.347924000
H	-7.014948000	-2.943988000	-1.774318000
H	-5.849613000	-4.040781000	-2.515211000
C	-3.950487000	-1.317645000	3.263647000
H	-3.947715000	-0.288425000	2.882894000
C	-5.104190000	-1.440845000	4.259229000
H	-6.071694000	-1.222483000	3.794240000
H	-4.971586000	-0.749229000	5.097561000
H	-5.160406000	-2.454644000	4.672555000
C	-2.605762000	-1.552367000	3.944640000
H	-2.457674000	-0.827964000	4.753268000
H	-1.783020000	-1.437175000	3.228936000
H	-2.545315000	-2.553477000	4.386962000
C	-2.649620000	2.895712000	-1.192765000
C	-2.398043000	4.159818000	-0.623775000
C	-1.442207000	4.979356000	-1.229362000
H	-1.231380000	5.956107000	-0.799526000
C	-0.732502000	4.554183000	-2.344049000
H	0.016997000	5.201972000	-2.792317000
C	-0.969127000	3.290913000	-2.873668000
H	-0.400625000	2.955494000	-3.737790000
C	-1.927301000	2.441095000	-2.318028000
C	-3.067418000	4.604961000	0.663871000
H	-3.972014000	4.003550000	0.809266000
C	-2.152309000	4.320484000	1.854834000
H	-1.219233000	4.890986000	1.774931000
H	-1.882801000	3.257967000	1.904205000
H	-2.635984000	4.594525000	2.798647000
C	-3.488825000	6.071307000	0.641953000
H	-4.064547000	6.318353000	1.539104000
H	-4.105364000	6.309387000	-0.231716000
H	-2.623963000	6.742935000	0.623335000
C	-2.225894000	1.090837000	-2.941376000
H	-2.449201000	0.398743000	-2.116185000
C	-1.050665000	0.485181000	-3.696891000
H	-0.818381000	1.038043000	-4.615181000
H	-1.292247000	-0.542023000	-3.994980000
H	-0.152330000	0.452506000	-3.069399000
C	-3.469492000	1.161054000	-3.829205000
H	-3.341850000	1.905782000	-4.623876000
H	-4.365030000	1.427345000	-3.258669000
H	-3.660980000	0.194481000	-4.307952000
H	0.347050000	0.283784000	-0.814952000
C	0.854730000	-0.614827000	2.383011000
C	1.014225000	-0.563264000	3.739239000
O	2.299827000	-0.672543000	1.913743000
C	2.393509000	-0.567590000	4.120637000
H	0.175269000	-0.509069000	4.421072000
C	3.159325000	-0.616788000	3.004462000
H	2.788799000	-0.540159000	5.128070000
C	4.610926000	-0.639331000	2.754063000
H	5.149039000	-0.631362000	3.704137000
H	4.920132000	-1.535375000	2.201476000
H	4.942271000	0.233774000	2.176259000

Int-7'_M06L.log

SCF (M06L) = -2685.71478448
E(SCF)+ZPE(0 K)= -2684.496546
H(298 K)= -2684.431238
G(298 K)= -2684.596005
Lowest Frequency = 4.4903 cm-1

Pd	0.900715000	0.093151000	0.046294000
Al	-1.435004000	0.002883000	0.415934000
N	-2.787384000	-1.236333000	0.964398000
N	-2.384240000	1.548749000	1.079730000
C	-3.153880000	-1.077088000	2.255163000
C	-3.141026000	0.167047000	2.890685000
H	-3.455800000	0.181839000	3.928590000
C	-2.927992000	1.428080000	2.289458000
C	-3.553367000	-2.280517000	3.057328000
H	-4.260089000	-2.921435000	2.525823000
H	-3.984501000	-1.997928000	4.018781000
H	-2.660874000	-2.889748000	3.245014000
C	-3.335525000	2.635647000	3.078366000
H	-4.280550000	2.458200000	3.596794000
H	-3.429429000	3.524126000	2.450887000
H	-2.582097000	2.847729000	3.845209000
C	-3.196288000	-2.423500000	0.275109000
C	-4.528207000	-2.502664000	-0.200080000
C	-4.923177000	-3.671022000	-0.855056000
H	-5.942163000	-3.755480000	-1.223110000
C	-4.033377000	-4.717822000	-1.070537000
H	-4.361798000	-5.612942000	-1.591513000
C	-2.723690000	-4.606093000	-0.629644000
H	-2.021328000	-5.418117000	-0.807641000
C	-2.282322000	-3.471048000	0.057815000
C	-5.506570000	-1.344632000	-0.058162000
H	-4.909332000	-0.421161000	-0.091359000
C	-6.531240000	-1.296768000	-1.193058000
H	-6.082125000	-1.435339000	-2.180356000
H	-7.053314000	-0.334099000	-1.188522000
H	-7.298187000	-2.069832000	-1.070751000
C	-6.274525000	-1.336022000	1.268443000
H	-6.762431000	-2.302472000	1.441671000
H	-7.058654000	-0.571557000	1.240965000
H	-5.642604000	-1.112528000	2.129434000
C	-0.846440000	-3.418622000	0.540583000
H	-0.704513000	-2.505178000	1.132624000

C	0.128848000	-3.353909000	-0.635088000
H	1.159396000	-3.273348000	-0.268965000
H	-0.055910000	-2.482647000	-1.273739000
H	0.059810000	-4.257085000	-1.254599000
C	-0.511311000	-4.601735000	1.447877000
H	-1.203264000	-4.679862000	2.292746000
H	0.502241000	-4.499749000	1.849474000
H	-0.552779000	-5.551575000	0.902594000
C	-2.184168000	2.818683000	0.442124000
C	-1.120029000	3.656373000	0.826650000
C	-0.896235000	4.813596000	0.073466000
H	-0.073573000	5.468865000	0.353066000
C	-1.690372000	5.134642000	-1.016407000
H	-1.490766000	6.035078000	-1.590666000
C	-2.746271000	4.302327000	-1.371171000
H	-3.367448000	4.560685000	-2.223570000
C	-3.012209000	3.135399000	-0.656262000
C	-0.220926000	3.377715000	2.016563000
H	-0.539408000	2.437251000	2.484438000
C	1.235246000	3.192637000	1.595079000
H	1.624740000	4.091144000	1.100725000
H	1.335693000	2.344385000	0.903142000
H	1.865072000	2.990773000	2.468828000
C	-0.325704000	4.499342000	3.052763000
H	0.086778000	5.434675000	2.657973000
H	0.243458000	4.248869000	3.953829000
H	-1.359485000	4.703244000	3.348563000
C	-4.178284000	2.236642000	-1.016968000
H	-3.821576000	1.201020000	-0.918070000
C	-4.675981000	2.417948000	-2.444634000
H	-5.142341000	3.398706000	-2.590313000
H	-5.438975000	1.666759000	-2.675471000
H	-3.864416000	2.312263000	-3.171420000
C	-5.339052000	2.407060000	-0.033429000
H	-5.058335000	2.143605000	0.990675000
H	-6.182437000	1.766061000	-0.316168000
H	-5.695310000	3.443438000	-0.029540000
H	-0.002598000	-0.269228000	1.594710000
C	-0.198737000	0.462769000	-1.714303000
C	-0.155008000	0.910784000	-3.005925000
O	-1.561629000	-0.143052000	-1.624153000
C	-1.359740000	0.587773000	-3.705683000
H	0.706113000	1.413029000	-3.428061000
C	-2.198130000	-0.055808000	-2.856103000
H	-1.576954000	0.777796000	-4.749324000
C	-3.496084000	-0.734876000	-3.005931000
H	-3.895298000	-0.560432000	-4.008029000
H	-3.411962000	-1.819050000	-2.852678000
H	-4.235586000	-0.371116000	-2.284829000
P	3.242658000	-0.119324000	0.110525000
C	4.195470000	1.400128000	-0.429391000
C	5.625807000	1.217687000	-0.948334000
C	3.391976000	2.228347000	-1.442189000
H	4.248059000	1.994157000	0.501985000
C	6.297466000	2.566728000	-1.193222000
H	5.587897000	0.663143000	-1.898175000
H	6.227809000	0.611446000	-0.263877000
C	4.053493000	3.576651000	-1.703005000
H	3.321207000	1.662970000	-2.384557000
H	2.357996000	2.353590000	-1.098550000
C	5.494911000	3.415429000	-2.171654000
H	7.321211000	2.420469000	-1.557758000
H	6.385062000	3.100058000	-0.234211000
H	3.471513000	4.149921000	-2.434199000
H	4.041246000	4.163815000	-0.771786000
H	5.969409000	4.393363000	-2.314204000
H	5.499082000	2.923894000	-3.155954000
C	3.923772000	-0.480950000	1.817387000
C	3.451571000	-1.867091000	2.271028000
C	5.407984000	-0.262017000	2.124502000
H	3.366597000	0.249419000	2.429222000
C	3.767585000	-2.114943000	3.741069000
H	3.945090000	-2.642854000	1.664913000
H	2.372434000	-1.959380000	2.080561000
C	5.702921000	-0.521038000	3.600941000
H	6.032425000	-0.926571000	1.511828000
H	5.701926000	0.762440000	1.863821000
C	5.251080000	-1.912505000	4.027389000
H	3.450005000	-3.122942000	4.033531000
H	3.182003000	-1.415384000	4.355527000
H	6.771281000	-0.380981000	3.803669000
H	5.173030000	0.228485000	4.207611000
H	5.467228000	-2.078386000	5.089175000
H	5.831755000	-2.665612000	3.473094000
C	3.759237000	-1.567599000	-0.955070000
C	3.343013000	-1.331499000	-2.412705000
C	5.179345000	-2.139415000	-0.884591000
H	3.088782000	-2.354755000	-0.568928000
C	3.462716000	-2.609261000	-3.235386000
H	3.991682000	-0.557073000	-2.852881000
H	2.318847000	-0.936867000	-2.446022000
C	5.279195000	-3.424747000	-1.704339000
H	5.903489000	-1.410739000	-1.270159000
H	5.466704000	-2.342287000	0.152959000
C	4.866295000	-3.197477000	-3.153343000
H	3.183395000	-2.417179000	-4.277864000
H	2.738761000	-3.344802000	-2.852951000
H	6.296565000	-3.830053000	-1.650075000
H	4.619956000	-4.184138000	-1.256285000
H	4.929918000	-4.131861000	-3.723181000
H	5.576872000	-2.500623000	-3.623067000

Int-8_M06L.log

SCF (M06L) = -2879.94954584
E(SCF)+ZPE(0 K)= -2878.575658
H(298 K)= -2878.477616
G(298 K)= -2878.688901
Lowest Frequency = 14.6129 cm-1

Pd	0.577367000	0.157115000	-0.328018000
Al	-2.465200000	0.247460000	0.648581000
N	-2.765369000	1.712850000	-0.570917000
N	-3.104663000	-1.107544000	-0.553117000
C	-2.350737000	1.505540000	-1.820443000
C	-2.134966000	0.203976000	-2.328890000
H	-1.717992000	0.153161000	-3.329490000
C	-2.635079000	-1.001568000	-1.807732000
C	-2.106471000	2.643492000	-2.765432000
H	-2.525443000	3.582183000	-2.398648000
H	-2.525795000	2.423935000	-3.750256000
H	-1.027069000	2.783808000	-2.899281000
C	-2.666241000	-2.189548000	-2.722451000
H	-3.679251000	-2.362810000	-3.100473000
H	-2.370456000	-3.102939000	-2.196904000
H	-2.009155000	-2.035377000	-3.579949000
C	-3.046239000	3.018341000	-0.049854000
C	-4.390167000	3.323018000	0.255606000
C	-4.674163000	4.551561000	0.851157000
H	-5.703020000	4.803975000	1.090008000
C	-3.661827000	5.456058000	1.151326000
H	-3.901270000	6.405231000	1.623123000
C	-2.347465000	5.138708000	0.849003000
H	-1.551756000	5.841143000	1.089214000
C	-2.007682000	3.921985000	0.247627000
C	-5.498917000	2.360641000	-0.119907000
H	-5.120329000	1.340703000	0.035800000
C	-6.749229000	2.521114000	0.733716000
H	-6.515946000	2.445823000	1.800334000

H	-7.479112000	1.743784000	0.492423000
H	-7.242993000	3.483771000	0.560730000
C	-5.831860000	2.500266000	-1.607440000
H	-6.185127000	3.513368000	-1.831886000
H	-6.619416000	1.798206000	-1.904528000
H	-4.958103000	2.305191000	-2.238191000
C	-0.547246000	3.646871000	-0.042093000
H	-0.454857000	2.672158000	-0.545732000
C	0.276035000	3.542081000	1.239535000
H	1.299895000	3.236115000	0.995082000
H	-0.135545000	2.792006000	1.920527000
H	0.324544000	4.508668000	1.758871000
C	0.046949000	4.719267000	-0.954499000
H	-0.517328000	4.842857000	-1.884796000
H	1.080204000	4.467328000	-1.208705000
H	0.067001000	5.693936000	-0.451991000
C	-3.839565000	-2.286256000	-0.196074000
C	-3.260282000	-3.244843000	0.665270000
C	-3.979590000	-4.409878000	0.936948000
H	-3.541913000	-5.160432000	1.588763000
C	-5.241127000	-4.627203000	0.395028000
H	-5.779253000	-5.545505000	0.614250000
C	-5.819806000	-3.650218000	-0.399999000
H	-6.827862000	-3.792641000	-0.785156000
C	-5.142729000	-2.464597000	-0.698989000
C	-1.890619000	-3.018253000	1.266761000
H	-1.851677000	-1.979303000	1.617035000
C	-1.595717000	-3.877442000	2.488212000
H	-1.458503000	-4.934935000	2.228230000
H	-2.389499000	-3.810453000	3.239321000
H	-0.664827000	-3.535873000	2.952344000
C	-0.779152000	-3.179351000	0.233273000
H	-0.802384000	-4.181826000	-0.212287000
H	0.203679000	-3.033410000	0.697419000
H	-0.849320000	-2.439886000	-0.569881000
C	-5.887505000	-1.372431000	-1.442318000
H	-5.191143000	-0.550500000	-1.652627000
C	-6.988487000	-0.829065000	-0.529821000
H	-7.717490000	-1.613191000	-0.295622000
H	-7.531024000	-0.005593000	-1.007584000
H	-6.577243000	-0.468480000	0.418646000
C	-6.487576000	-1.837579000	-2.766778000
H	-5.735705000	-2.254089000	-3.444506000
H	-6.973680000	-1.002761000	-3.282562000
H	-7.249002000	-2.610087000	-2.611805000
Al	2.832421000	-0.248533000	-0.388000000
N	4.360774000	0.891491000	-0.335721000
N	3.815396000	-1.886962000	-0.418521000
C	5.632215000	0.484631000	-0.454627000
C	5.990113000	-0.860537000	-0.605827000
H	7.048162000	-1.064460000	-0.720643000
C	5.147828000	-1.977694000	-0.550559000
C	6.733542000	1.498955000	-0.397157000
H	7.704320000	1.040389000	-0.586187000
H	6.760333000	1.981237000	0.586030000
H	6.570017000	2.304539000	-1.119626000
C	5.789147000	-3.331742000	-0.604639000
H	5.710934000	-3.835244000	0.365366000
H	6.844638000	-3.256316000	-0.867435000
H	5.282670000	-3.983155000	-1.322926000
C	4.114843000	2.292600000	-0.108529000
C	3.868188000	3.135380000	-1.207054000
C	3.681428000	4.498185000	-0.960864000
H	3.492217000	5.164223000	-1.800999000
C	3.713625000	5.008930000	0.329891000
H	3.557088000	6.070592000	0.500475000
C	3.917639000	4.152229000	1.403979000
H	3.902759000	4.546867000	2.417485000
C	4.118188000	2.783476000	1.211556000
C	3.770642000	2.615564000	-2.627946000
H	3.950792000	1.533822000	-2.609443000
C	2.363306000	2.833629000	-3.183837000
H	2.134915000	3.902392000	-3.276540000
H	1.609129000	2.377568000	-2.531394000
H	2.266265000	2.385188000	-4.178197000
C	4.818710000	3.249483000	-3.540815000
H	4.747590000	2.843380000	-4.554705000
H	5.838078000	3.073375000	-3.181774000
H	4.679785000	4.334032000	-3.612323000
C	4.248636000	1.867947000	2.413692000
H	4.595745000	0.885291000	2.065013000
C	5.261854000	2.376066000	3.435882000
H	6.248305000	2.545359000	2.991433000
H	5.379045000	1.654261000	4.249886000
H	4.939791000	3.319633000	3.889046000
C	2.872949000	1.671848000	3.055513000
H	2.932702000	0.994583000	3.914320000
H	2.147456000	1.262026000	2.339820000
H	2.471382000	2.628316000	3.410936000
C	3.077545000	-3.111929000	-0.232843000
C	3.032409000	-3.689835000	1.053263000
C	2.365550000	-4.906482000	1.204294000
H	2.312545000	-5.365735000	2.188117000
C	1.766793000	-5.537896000	0.120035000
H	1.258758000	-6.488740000	0.256966000
C	1.792497000	-4.934665000	-1.128350000
H	1.294502000	-5.412645000	-1.969956000
C	2.431645000	-3.707380000	-1.329207000
C	3.623786000	-2.980229000	2.256349000
H	4.440473000	-2.333839000	1.906776000
C	2.563614000	-2.076593000	2.891545000
H	1.734554000	-2.676240000	3.285468000
H	2.126078000	-1.374350000	2.167898000
H	2.984414000	-1.499263000	3.722356000
C	4.211636000	-3.930598000	3.294097000
H	4.726991000	-3.366135000	4.076703000
H	4.929415000	-4.630608000	2.853754000
H	3.435096000	-4.521961000	3.790418000
C	2.394173000	-3.068671000	-2.703177000
H	2.955812000	-2.127234000	-2.661282000
C	0.957538000	-2.724676000	-3.095308000
H	0.334383000	-3.626061000	-3.147688000
H	0.929693000	-2.240073000	-4.077756000
H	0.503545000	-2.037930000	-2.369298000
C	3.053512000	-3.956039000	-3.757615000
H	2.523961000	-4.909389000	-3.864123000
H	4.094154000	-4.185938000	-3.505955000
H	3.046211000	-3.466861000	-4.736701000
H	1.788051000	0.039841000	-1.775445000
C	-0.616892000	0.161986000	1.257987000
C	-0.525257000	0.058833000	2.615799000
O	-3.483010000	0.257821000	2.090349000
C	-1.615549000	0.010713000	3.575488000
H	0.462772000	-0.014277000	3.095316000
C	-2.953193000	0.101790000	3.316916000
H	-1.344670000	-0.110306000	4.622315000
C	-3.988073000	0.032892000	4.389061000
H	-3.543353000	-0.073122000	5.380838000
H	-4.612029000	0.934390000	4.378457000
H	-4.664737000	-0.812594000	4.215947000

Int-8'-1_M06L.log

SCF (M06L) = -2685.74644536
E(SCF)+ZPE(0 K)= -2684.526953
H(298 K)= -2684.461957
G(298 K)= -2684.623163
Lowest Frequency = 15.7388 cm-1

Pd	-0.848129000	-0.017116000	-1.027750000

Al	1.532870000	-0.020760000	-0.445952000
N	2.104226000	1.344427000	0.803148000
N	1.996242000	-1.509691000	0.714670000
C	1.828239000	1.166409000	2.097613000
C	1.595952000	-0.105371000	2.643933000
H	1.383128000	-0.132102000	3.706847000
C	1.804047000	-1.354854000	2.030977000
C	1.748743000	2.348485000	3.018357000
H	2.559643000	3.060797000	2.845551000
H	1.756950000	2.043062000	4.066008000
H	0.813448000	2.890690000	2.829359000
C	1.836189000	-2.551891000	2.935091000
H	1.844059000	-2.252349000	3.983845000
H	2.714742000	-3.172980000	2.734950000
H	0.965514000	-3.192630000	2.766789000
C	2.637796000	2.593741000	0.351023000
C	4.041780000	2.728147000	0.296819000
C	4.570651000	3.935930000	-0.160237000
H	5.649592000	4.061385000	-0.206557000
C	3.741715000	4.978204000	-0.562551000
H	4.172996000	5.910495000	-0.916947000
C	2.364180000	4.818961000	-0.520860000
H	1.715520000	5.628061000	-0.851821000
C	1.785288000	3.628597000	-0.069654000
C	4.945100000	1.600144000	0.757745000
H	4.401433000	0.663944000	0.576749000
C	6.263201000	1.527942000	-0.003907000
H	6.111136000	1.495415000	-1.086426000
H	6.813882000	0.626063000	0.285794000
H	6.915458000	2.379743000	0.219925000
C	5.223863000	1.688449000	2.260533000
H	5.673772000	2.654960000	2.517120000
H	5.921473000	0.903031000	2.571419000
H	4.316222000	1.571103000	2.859185000
C	0.277863000	3.476898000	-0.091686000
H	0.021787000	2.503183000	0.349500000
C	-0.232758000	3.469979000	-1.532474000
H	-1.308090000	3.258290000	-1.560524000
H	0.263332000	2.702797000	-2.135660000
H	-0.066461000	4.443094000	-2.010933000
C	-0.429717000	4.561304000	0.718730000
H	-0.075155000	4.605474000	1.754009000
H	-1.511561000	4.380625000	0.740823000
H	-0.277437000	5.554253000	0.280020000
C	2.262395000	-2.828828000	0.209892000
C	1.216281000	-3.761736000	0.055775000
C	1.538166000	-5.046069000	-0.393761000
H	0.738610000	-5.774243000	-0.517294000
C	2.843519000	-5.399505000	-0.698601000
H	3.072244000	-6.404253000	-1.043257000
C	3.855716000	-4.453673000	-0.585123000
H	4.872600000	-4.726473000	-0.852373000
C	3.590043000	-3.157336000	-0.140649000
C	-0.246171000	-3.419150000	0.276830000
H	-0.308941000	-2.394666000	0.670895000
C	-0.990806000	-3.442805000	-1.060322000
H	-0.999932000	-4.456033000	-1.481040000
H	-0.537616000	-2.768195000	-1.792943000
H	-2.033347000	-3.130567000	-0.923185000
C	-0.952384000	-4.359128000	1.254349000
H	-0.967710000	-5.388051000	0.877548000
H	-1.996461000	-4.050391000	1.387311000
H	-0.487618000	-4.384785000	2.245239000
C	4.699934000	-2.129752000	-0.024746000
H	4.275853000	-1.171135000	-0.350430000
C	5.887556000	-2.424766000	-0.933316000
H	6.460129000	-3.296385000	-0.595303000
H	6.576756000	-1.573927000	-0.938599000
H	5.573788000	-2.610746000	-1.965344000
C	5.176852000	-1.963627000	1.419376000
H	4.390274000	-1.578777000	2.075953000
H	6.013297000	-1.256127000	1.467486000
H	5.527712000	-2.918377000	1.829377000
H	-0.284575000	0.062927000	0.535682000
C	-0.114877000	-0.075639000	-2.774582000
C	0.674873000	0.013311000	-3.844289000
O	2.777405000	0.105053000	-1.684345000
C	2.102126000	0.187403000	-3.980528000
H	0.114852000	-0.031368000	-4.785191000
C	3.023351000	0.245720000	-2.982298000
H	2.479809000	0.306334000	-4.991374000
C	4.465684000	0.510089000	-3.272731000
H	4.676843000	0.523847000	-4.343830000
H	4.758632000	1.480210000	-2.851473000
H	5.102603000	-0.244089000	-2.795114000
P	-2.995207000	-0.028264000	0.016268000
C	-4.234146000	-1.204856000	-0.735687000
C	-5.722088000	-0.946133000	-0.475300000
C	-3.997311000	-1.390641000	-2.240396000
H	-3.972724000	-2.163228000	-0.249885000
C	-6.572985000	-2.095025000	-1.010122000
H	-6.014537000	-0.017841000	-0.988294000
H	-5.921871000	-0.785879000	0.589331000
C	-4.849873000	-2.528653000	-2.789297000
H	-4.248253000	-0.453714000	-2.760789000
H	-2.932752000	-1.561940000	-2.438615000
C	-6.331056000	-2.317376000	-2.497873000
H	-7.634021000	-1.901427000	-0.814576000
H	-6.319784000	-3.012256000	-0.456886000
H	-4.680615000	-2.640924000	-3.865959000
H	-4.520458000	-3.471952000	-2.327078000
H	-6.922570000	-3.165240000	-2.861350000
H	-6.684081000	-1.435121000	-3.052095000
C	-2.838226000	-0.587428000	1.792672000
C	-2.165820000	0.480169000	2.665214000
C	-4.064622000	-1.172834000	2.499525000
H	-2.104412000	-1.407682000	1.693166000
C	-1.782362000	-0.099190000	4.021583000
H	-2.852727000	1.326730000	2.815024000
H	-1.277784000	0.874788000	2.155171000
C	-3.681687000	-1.730502000	3.869401000
H	-4.835911000	-0.398915000	2.623999000
H	-4.519007000	-1.967121000	1.894281000
C	-2.995537000	-0.681410000	4.735787000
H	-1.293540000	0.666607000	4.636941000
H	-1.034475000	-0.892292000	3.863531000
H	-4.568224000	-2.131763000	4.373492000
H	-2.996494000	-2.579886000	3.726348000
H	-2.709209000	-1.110906000	5.702472000
H	-3.707524000	0.128008000	4.956480000
C	-3.701239000	1.693004000	0.053855000
C	-3.951447000	2.203905000	-1.371709000
C	-4.902697000	2.009196000	0.951724000
H	-2.841976000	2.266371000	0.447971000
C	-4.250057000	3.699248000	-1.371768000
H	-4.808830000	1.663306000	-1.802109000
H	-3.089477000	1.972778000	-2.012273000
C	-5.176894000	3.511239000	0.958953000
H	-5.793364000	1.485145000	0.583419000
H	-4.735609000	1.652107000	1.973835000
C	-5.417463000	4.036467000	-0.451557000
H	-4.447338000	4.045775000	-2.392417000
H	-3.353177000	4.240195000	-1.032042000
H	-6.032061000	3.736179000	1.606518000
H	-4.311471000	4.030761000	1.398260000
H	-5.594848000	5.117612000	-0.434795000
H	-6.335129000	3.579596000	-0.851327000

Int-8’-2_M06L.log

SCF (M06L) = -2685.74567278

E(SCF)+ZPE(0 K)= -2684.525938
H(298 K)= -2684.461009
G(298 K)= -2684.622944
Lowest Frequency = 14.4153 cm-1

Pd	-0.936028000	0.772250000	-0.329995000
Al	1.808481000	0.002940000	-0.607629000
N	2.231250000	1.323513000	0.720329000
N	1.866511000	-1.499736000	0.575867000
C	1.744280000	1.171269000	1.957817000
C	1.307255000	-0.072666000	2.444679000
H	0.900537000	-0.071899000	3.450301000
C	1.464438000	-1.332662000	1.847315000
C	1.608138000	2.358095000	2.857840000
H	2.428124000	3.070022000	2.738817000
H	1.529558000	2.065386000	3.905920000
H	0.680942000	2.879728000	2.581333000
C	1.190712000	-2.537163000	2.696690000
H	1.014375000	-2.255918000	3.735575000
H	2.024698000	-3.245060000	2.655543000
H	0.313134000	-3.079975000	2.329391000
C	2.970044000	2.507131000	0.369860000
C	4.367460000	2.480573000	0.554858000
C	5.106581000	3.613825000	0.211573000
H	6.183175000	3.611216000	0.367398000
C	4.492559000	4.739443000	-0.323097000
H	5.084500000	5.611505000	-0.587486000
C	3.119596000	4.739470000	-0.521405000
H	2.633913000	5.614401000	-0.949315000
C	2.333463000	3.634755000	-0.180515000
C	5.066087000	1.277043000	1.153570000
H	4.366107000	0.434351000	1.119120000
C	6.303923000	0.872615000	0.360586000
H	6.046581000	0.627673000	-0.675179000
H	6.773615000	-0.007963000	0.812720000
H	7.061759000	1.663260000	0.345151000
C	5.408460000	1.523930000	2.623045000
H	6.084905000	2.379845000	2.731324000
H	5.901701000	0.650603000	3.063948000
H	4.512229000	1.734636000	3.216416000
C	0.845278000	3.684903000	-0.436282000
H	0.393960000	2.778243000	-0.006634000
C	0.552254000	3.681439000	-1.935628000
H	-0.527633000	3.626812000	-2.116352000
H	1.010690000	2.822310000	-2.435919000
H	0.931929000	4.594428000	-2.410469000
C	0.170742000	4.871682000	0.245826000
H	0.385084000	4.902145000	1.319205000
H	-0.916211000	4.808205000	0.122595000
H	0.496542000	5.826562000	-0.183035000
C	2.116488000	-2.832375000	0.092251000
C	1.054773000	-3.667572000	-0.309296000
C	1.354709000	-4.973233000	-0.710952000
H	0.541358000	-5.625810000	-1.022117000
C	2.658459000	-5.440933000	-0.737881000
H	2.868953000	-6.461068000	-1.047110000
C	3.699117000	-4.585749000	-0.397297000
H	4.722620000	-4.943619000	-0.456280000
C	3.456276000	-3.272415000	0.009050000
C	-0.384429000	-3.200616000	-0.413537000
H	-0.453684000	-2.172837000	-0.025840000
C	-0.801326000	-3.162312000	-1.884875000
H	-0.803228000	-4.171639000	-2.313745000
H	-0.129954000	-2.534697000	-2.474983000
H	-1.811878000	-2.752473000	-1.988465000
C	-1.356594000	-4.089794000	0.361805000
H	-1.350199000	-5.116920000	-0.019757000
H	-2.383311000	-3.716578000	0.257529000
H	-1.130982000	-4.141183000	1.432929000
C	4.608263000	-2.347749000	0.354662000
H	4.340037000	-1.349955000	-0.017477000
C	5.909764000	-2.741917000	-0.334505000
H	6.324567000	-3.672674000	0.068804000
H	6.665907000	-1.966428000	-0.185303000
H	5.772124000	-2.873927000	-1.411976000
C	4.821856000	-2.243477000	1.866019000
H	3.964357000	-1.794551000	2.377914000
H	5.695744000	-1.620414000	2.089357000
H	5.000809000	-3.232645000	2.304145000
H	-1.246018000	1.638374000	1.062196000
C	0.204516000	0.103883000	-1.798898000
C	0.531285000	0.028263000	-3.117405000
O	3.158759000	0.025694000	-1.758421000
C	1.831297000	0.150737000	-3.738137000
H	-0.281010000	-0.177292000	-3.833119000
C	3.030316000	0.139415000	-3.082238000
H	1.869209000	0.206280000	-4.822805000
C	4.340094000	0.227054000	-3.790388000
H	4.220740000	0.270150000	-4.874623000
H	4.888585000	1.118094000	-3.462065000
H	4.968984000	-0.634317000	-3.536039000
P	-3.034476000	0.120263000	0.120622000
C	-3.862088000	-0.849378000	-1.246552000
C	-5.394236000	-0.874171000	-1.264847000
C	-3.334146000	-0.445981000	-2.628663000
H	-3.509705000	-1.879342000	-1.053918000
C	-5.905624000	-1.833933000	-2.336355000
H	-5.760895000	0.137862000	-1.492157000
H	-5.806423000	-1.140629000	-0.285843000
C	-3.857035000	-1.379672000	-3.713957000
H	-3.649087000	0.587032000	-2.844615000
H	-2.239081000	-0.430529000	-2.609282000
C	-5.378783000	-1.455482000	-3.714810000
H	-7.001544000	-1.852628000	-2.330726000
H	-5.577766000	-2.854540000	-2.086264000
H	-3.482858000	-1.061015000	-4.693613000
H	-3.446795000	-2.386966000	-3.543655000
H	-5.732381000	-2.165946000	-4.470473000
H	-5.789729000	-0.474727000	-3.996675000
C	-3.028968000	-1.027373000	1.592115000
C	-2.727512000	-0.275758000	2.893504000
C	-4.234737000	-1.960360000	1.759181000
H	-2.153471000	-1.666567000	1.387226000
C	-2.520219000	-1.248880000	4.048222000
H	-3.567618000	0.393675000	3.134630000
H	-1.847714000	0.363208000	2.750701000
C	-4.022804000	-2.916596000	2.930911000
H	-5.146700000	-1.371724000	1.932824000
H	-4.410701000	-2.536197000	0.841723000
C	-3.722523000	-2.167972000	4.223015000
H	-2.312765000	-0.698110000	4.973028000
H	-1.626498000	-1.858744000	3.843578000
H	-4.900111000	-3.562619000	3.049078000
H	-3.178676000	-3.581980000	2.695830000
H	-3.557072000	-2.873562000	5.044932000
H	-4.598800000	-1.565167000	4.505012000
C	-4.078701000	1.590454000	0.579639000
C	-4.099340000	2.601150000	-0.573097000
C	-5.479044000	1.398941000	1.171072000
H	-3.449652000	2.031199000	1.369019000
C	-4.688142000	3.932717000	-0.122189000
H	-4.698848000	2.197279000	-1.404445000
H	-3.079318000	2.737261000	-0.959411000
C	-6.037703000	2.743534000	1.634009000
H	-6.161497000	0.970245000	0.428311000
H	-5.452475000	0.696220000	2.012282000
C	-6.071352000	3.752450000	0.492016000
H	-4.725476000	4.634777000	-0.962789000
H	-4.015669000	4.379263000	0.624840000
H	-7.038341000	2.607477000	2.059988000
H	-5.405648000	3.134193000	2.445373000

H	-6.467181000	4.713276000	0.839670000
H	-6.766707000	3.394520000	-0.282037000

Int-9_M06L.log

SCF (M06L) = -2879.93042703
E(SCF)+ZPE(0 K)= -2878.557136
H(298 K)= -2878.476357
G(298 K)= -2878.670696
Lowest Frequency = 12.8420 cm-1

Pd	0.689967000	0.450397000	-0.532495000
Al	-2.590333000	0.365894000	-0.545588000
N	-3.064167000	-1.337589000	0.192080000
N	-2.739347000	1.320976000	1.110953000
C	-2.837718000	-1.659759000	1.466554000
C	-2.511271000	-0.694549000	2.431754000
H	-2.312954000	-1.064827000	3.431437000
C	-2.551044000	0.699650000	2.288644000
C	-2.923892000	-3.089516000	1.904270000
H	-3.726142000	-3.627315000	1.392662000
H	-3.062609000	-3.172521000	2.983858000
H	-1.984814000	-3.593996000	1.646821000
C	-2.406931000	1.520800000	3.534971000
H	-2.376484000	0.888116000	4.422980000
H	-3.230253000	2.235635000	3.632762000
H	-1.487479000	2.114190000	3.503338000
C	-3.728111000	-2.245443000	-0.700020000
C	-5.133138000	-2.145090000	-0.793883000
C	-5.795736000	-2.964780000	-1.707766000
H	-6.879487000	-2.908682000	-1.782803000
C	-5.094872000	-3.848340000	-2.520198000
H	-5.626681000	-4.475493000	-3.230635000
C	-3.713864000	-3.926094000	-2.418439000
H	-3.164505000	-4.619409000	-3.052356000
C	-3.001761000	-3.132073000	-1.513116000
C	-5.924722000	-1.218402000	0.108582000
H	-5.225776000	-0.494677000	0.544376000
C	-6.982858000	-0.420340000	-0.643430000
H	-6.521554000	0.209735000	-1.409977000
H	-7.526659000	0.230155000	0.051062000
H	-7.727118000	-1.064801000	-1.123892000
C	-6.547894000	-1.999066000	1.266958000
H	-7.245960000	-2.759671000	0.897978000
H	-7.103544000	-1.331252000	1.934551000
H	-5.788831000	-2.511918000	1.867106000
C	-1.497653000	-3.289889000	-1.424149000
H	-1.108498000	-2.567901000	-0.691994000
C	-0.821125000	-2.993511000	-2.761188000
H	0.266956000	-3.075099000	-2.664869000
H	-1.048268000	-1.981021000	-3.105243000
H	-1.141468000	-3.704157000	-3.532671000
C	-1.140001000	-4.701501000	-0.955128000
H	-1.631328000	-4.965242000	-0.012407000
H	-0.060035000	-4.796846000	-0.806433000
H	-1.440904000	-5.449330000	-1.698423000
C	-2.876944000	2.754629000	1.105776000
C	-1.737156000	3.579954000	1.160583000
C	-1.918328000	4.965146000	1.210352000
H	-1.041061000	5.607776000	1.256220000
C	-3.186044000	5.524833000	1.189550000
H	-3.309997000	6.603338000	1.236254000
C	-4.298800000	4.699430000	1.078997000
H	-5.287311000	5.145797000	1.025740000
C	-4.172968000	3.309636000	1.022986000
C	-0.323975000	3.040319000	1.098273000
H	-0.371136000	1.938786000	1.147054000
C	0.321002000	3.434573000	-0.236506000
H	0.479346000	4.519549000	-0.282252000
H	-0.301119000	3.144369000	-1.088469000
H	1.306356000	2.956991000	-0.355806000
C	0.552808000	3.505240000	2.259671000
H	0.809784000	4.567388000	2.167029000
H	1.489691000	2.935680000	2.269703000
H	0.070891000	3.369100000	3.233426000
C	-5.398377000	2.426907000	0.869763000
H	-5.119910000	1.612378000	0.189090000
C	-6.572610000	3.154072000	0.224490000
H	-7.005279000	3.911780000	0.887975000
H	-7.370767000	2.444622000	-0.011841000
H	-6.278068000	3.649182000	-0.705767000
C	-5.833176000	1.800052000	2.195853000
H	-5.079103000	1.118857000	2.603034000
H	-6.753320000	1.219393000	2.059526000
H	-6.038348000	2.571468000	2.947955000
Al	2.901007000	0.165778000	0.502261000
N	4.631951000	0.626902000	-0.314838000
N	3.728422000	-1.320110000	1.464120000
C	5.845600000	0.198990000	0.041723000
C	6.031798000	-0.762575000	1.047923000
H	7.057497000	-1.005752000	1.302005000
C	5.036746000	-1.522070000	1.674553000
C	7.063718000	0.747250000	-0.642719000
H	7.279960000	1.763572000	-0.294041000
H	6.911175000	0.820068000	-1.723290000
H	7.941882000	0.130685000	-0.445569000
C	5.468476000	-2.633343000	2.584049000
H	4.865689000	-2.650721000	3.496986000
H	6.522680000	-2.541841000	2.849531000
H	5.324620000	-3.607397000	2.103683000
C	4.455085000	1.529917000	-1.416234000
C	3.918096000	1.008294000	-2.614896000
C	3.613105000	1.903519000	-3.641309000
H	3.192157000	1.525523000	-4.568437000
C	3.821119000	3.269827000	-3.491337000
H	3.567626000	3.949751000	-4.299814000
C	4.343284000	3.763341000	-2.304207000
H	4.490204000	4.834668000	-2.184568000
C	4.665806000	2.910153000	-1.245394000
C	3.716322000	-0.484870000	-2.797147000
H	3.402975000	-0.896890000	-1.826526000
C	2.612742000	-0.836625000	-3.784281000
H	2.871805000	-0.557129000	-4.811781000
H	1.670554000	-0.348981000	-3.512129000
H	2.439437000	-1.917885000	-3.780706000
C	5.029082000	-1.175688000	-3.169328000
H	4.872599000	-2.249895000	-3.317343000
H	5.789168000	-1.059087000	-2.389962000
H	5.437880000	-0.767234000	-4.100875000
C	5.147350000	3.497918000	0.066795000
H	5.532224000	2.681895000	0.689771000
C	6.274858000	4.509067000	-0.122819000
H	7.110072000	4.088293000	-0.692636000
H	6.659866000	4.845150000	0.844955000
H	5.932118000	5.400176000	-0.659085000
C	3.974355000	4.118182000	0.823676000
H	4.290000000	4.518303000	1.793340000
H	3.188938000	3.375041000	0.999200000
H	3.523896000	4.936488000	0.249567000
C	2.771712000	-2.293467000	1.905342000
C	1.891460000	-1.980448000	2.959950000
C	0.877072000	-2.890914000	3.269747000
H	0.190360000	-2.658778000	4.082569000
C	0.735337000	-4.079851000	2.567172000
H	-0.056787000	-4.778601000	2.825655000
C	1.615389000	-4.375406000	1.533084000
H	1.497930000	-5.303062000	0.976249000
C	2.637216000	-3.494668000	1.176045000
C	2.028521000	-0.718253000	3.788990000
H	2.830946000	-0.109406000	3.352208000

C	0.753279000	0.120537000	3.770133000
H	-0.082790000	-0.412646000	4.237911000
H	0.456970000	0.365908000	2.741838000
H	0.898013000	1.055358000	4.323471000
C	2.434923000	-1.053040000	5.224435000
H	2.568959000	-0.141688000	5.815970000
H	3.370476000	-1.620096000	5.262194000
H	1.665777000	-1.657845000	5.718271000
C	3.506115000	-3.821433000	-0.025619000
H	4.325272000	-3.093600000	-0.079276000
C	2.693439000	-3.682208000	-1.312858000
H	1.919980000	-4.457230000	-1.368670000
H	3.330885000	-3.790641000	-2.197975000
H	2.185219000	-2.711614000	-1.366582000
C	4.126432000	-5.214412000	0.062233000
H	3.359535000	-5.996115000	0.035091000
H	4.701750000	-5.357033000	0.983043000
H	4.797504000	-5.391521000	-0.783911000
H	0.764850000	-0.953431000	-1.205748000
C	-0.999715000	0.483421000	-1.618705000
C	-1.193784000	0.681026000	-2.954011000
O	-3.888416000	0.831136000	-1.677289000
C	-2.461029000	0.963641000	-3.595812000
H	-0.348831000	0.620455000	-3.655587000
C	-3.684265000	1.008056000	-2.987720000
H	-2.452229000	1.127156000	-4.670698000
C	-4.948952000	1.224808000	-3.750254000
H	-4.763357000	1.431118000	-4.806315000
H	-5.589207000	0.335990000	-3.677755000
H	-5.521635000	2.056646000	-3.322619000

Int-9’_M06L.log

SCF (M06L) = -2685.75902092
E(SCF)+ZPE(0 K)= -2684.539452
H(298 K)= -2684.474384
G(298 K)= -2684.636316
Lowest Frequency = 14.3640 cm-1

Pd	1.072438000	0.081917000	-1.250012000
Al	-2.013302000	-0.040206000	-0.724407000
N	-1.832280000	-1.374848000	0.648330000
N	-2.023643000	1.446357000	0.477544000
C	-1.146838000	-1.103459000	1.760338000
C	-0.714333000	0.198410000	2.077431000
H	-0.087357000	0.291863000	2.958071000
C	-1.197416000	1.402930000	1.533679000
C	-0.856030000	-2.207969000	2.728475000
H	-1.768267000	-2.770173000	2.950493000
H	-0.440305000	-1.826277000	3.660780000
H	-0.151027000	-2.930468000	2.301608000
C	-0.814688000	2.676882000	2.224528000
H	-0.131232000	2.487910000	3.054010000
H	-1.699986000	3.198083000	2.604585000
H	-0.334102000	3.366172000	1.521973000
C	-2.307516000	-2.708500000	0.404328000
C	-3.630284000	-3.034034000	0.763419000
C	-4.104362000	-4.313820000	0.468859000
H	-5.125537000	-4.572822000	0.742143000
C	-3.299030000	-5.253286000	-0.160072000
H	-3.684982000	-6.243930000	-0.383789000
C	-1.996233000	-4.918197000	-0.500330000
H	-1.363005000	-5.652412000	-0.994311000
C	-1.472564000	-3.650470000	-0.230268000
C	-4.543339000	-2.056568000	1.475575000
H	-3.983304000	-1.127367000	1.639679000
C	-5.767593000	-1.722524000	0.625180000
H	-5.476133000	-1.224371000	-0.303918000
H	-6.448527000	-1.061936000	1.172011000
H	-6.329158000	-2.628432000	0.369362000
C	-4.964944000	-2.589415000	2.844993000
H	-5.551310000	-3.510382000	2.751053000
H	-5.588670000	-1.858288000	3.370277000
H	-4.103356000	-2.814161000	3.482454000
C	-0.041300000	-3.360477000	-0.642103000
H	0.211020000	-2.328743000	-0.342466000
C	0.121038000	-3.446188000	-2.159052000
H	1.138664000	-3.162838000	-2.451617000
H	-0.569582000	-2.774403000	-2.674446000
H	-0.059330000	-4.469192000	-2.510293000
C	0.945299000	-4.308599000	0.040930000
H	0.872057000	-4.282520000	1.133938000
H	1.975487000	-4.051822000	-0.230303000
H	0.773386000	-5.344964000	-0.270721000
C	-2.683742000	2.687507000	0.162516000
C	-2.040380000	3.673044000	-0.608086000
C	-2.709042000	4.881209000	-0.828851000
H	-2.218699000	5.650332000	-1.422203000
C	-3.980192000	5.104036000	-0.324231000
H	-4.481858000	6.051600000	-0.500984000
C	-4.626683000	4.094882000	0.380077000
H	-5.639985000	4.259778000	0.733648000
C	-4.005262000	2.869258000	0.626461000
C	-0.705198000	3.441987000	-1.280123000
H	-0.282337000	2.491739000	-0.922918000
C	-0.909639000	3.309784000	-2.790087000
H	-1.225566000	4.268248000	-3.220534000
H	-1.668719000	2.560895000	-3.030971000
H	0.020890000	2.998583000	-3.276558000
C	0.312589000	4.541370000	-0.994252000
H	-0.002231000	5.505284000	-1.410668000
H	1.272480000	4.284778000	-1.456542000
H	0.487258000	4.690616000	0.077521000
C	-4.739469000	1.755755000	1.351536000
H	-4.455635000	0.817303000	0.853857000
C	-6.254944000	1.879680000	1.248845000
H	-6.632941000	2.751508000	1.794294000
H	-6.735321000	1.001808000	1.690541000
H	-6.587715000	1.959348000	0.209550000
C	-4.331328000	1.641545000	2.821822000
H	-3.273831000	1.392509000	2.946545000
H	-4.910151000	0.855071000	3.319587000
H	-4.524807000	2.580673000	3.353747000
H	1.386514000	1.040476000	-2.455563000
C	-0.688711000	-0.036448000	-2.145232000
C	-1.197003000	-0.186239000	-3.400613000
O	-3.556470000	-0.279726000	-1.558692000
C	-2.577986000	-0.463983000	-3.738660000
H	-0.538505000	-0.089600000	-4.271782000
C	-3.640678000	-0.494525000	-2.880814000
H	-2.806537000	-0.640525000	-4.786746000
C	-5.034674000	-0.766818000	-3.336961000
H	-5.096023000	-0.882792000	-4.420751000
H	-5.419535000	-1.680730000	-2.867520000
H	-5.704372000	0.044861000	-3.029545000
P	3.151489000	0.237909000	-0.107567000
C	4.451490000	-0.718566000	-1.053591000
C	5.731692000	-1.148042000	-0.330510000
C	3.814217000	-1.927328000	-1.753306000
H	4.734469000	-0.004859000	-1.847481000
C	6.709735000	-1.816233000	-1.293695000
H	5.472545000	-1.865556000	0.462719000
H	6.211402000	-0.298967000	0.167383000
C	4.790245000	-2.617992000	-2.697457000
H	3.474589000	-2.644826000	-0.988769000
H	2.911297000	-1.603699000	-2.287778000
C	6.075911000	-3.017332000	-1.984086000
H	7.620293000	-2.111597000	-0.759719000
H	7.021323000	-1.082353000	-2.052205000
H	4.312566000	-3.491362000	-3.156893000

H	5.032972000	-1.931536000	-3.521880000
H	6.782089000	-3.476447000	-2.685048000
H	5.847443000	-3.784538000	-1.229128000
C	3.771753000	1.998175000	-0.118819000
C	2.948839000	2.837722000	0.866395000
C	5.279140000	2.236578000	0.028319000
H	3.490055000	2.335107000	-1.130172000
C	3.334007000	4.312288000	0.827088000
H	3.096181000	2.464597000	1.891418000
H	1.879799000	2.704187000	0.640965000
C	5.614709000	3.724993000	-0.015649000
H	5.646681000	1.819067000	0.974441000
H	5.819491000	1.709696000	-0.767822000
C	4.831021000	4.500399000	1.035670000
H	2.758121000	4.869433000	1.576383000
H	3.056776000	4.734832000	-0.148627000
H	6.693202000	3.869147000	0.115327000
H	5.368173000	4.122481000	-1.011122000
H	5.093126000	5.563811000	1.006200000
H	5.111195000	4.139902000	2.037382000
C	3.178087000	-0.291290000	1.688216000
C	2.763676000	-1.764124000	1.788174000
C	4.398889000	0.008693000	2.566981000
H	2.334232000	0.292249000	2.097956000
C	2.582100000	-2.208579000	3.234325000
H	3.525869000	-2.399961000	1.311379000
H	1.837976000	-1.919961000	1.213544000
C	4.181868000	-0.456321000	4.005125000
H	5.289696000	-0.488130000	2.165124000
H	4.615683000	1.082251000	2.560162000
C	3.829231000	-1.936087000	4.063336000
H	2.302089000	-3.268791000	3.273942000
H	1.743055000	-1.649677000	3.674099000
H	5.073489000	-0.242112000	4.605266000
H	3.361382000	0.125264000	4.452902000
H	3.682550000	-2.259962000	5.099724000
H	4.669230000	-2.527194000	3.668888000

Int-10_M06L.log

SCF (M06L) = -2879.97074556
E(SCF)+ZPE(0 K)= -2878.593234
H(298 K)= -2878.51266
G(298 K)= -2878.708256
Lowest Frequency = 12.5599 cm-1

Pd	-0.759698000	-0.406674000	-0.476824000
Al	2.582470000	-0.326782000	-0.665858000
N	3.133534000	1.380579000	-0.008096000
N	2.723017000	-1.230182000	1.032687000
C	2.917977000	1.740000000	1.267283000
C	2.557207000	0.830280000	2.268294000
H	2.372262000	1.250696000	3.250238000
C	2.563497000	-0.571775000	2.187734000
C	3.088172000	3.175369000	1.659304000
H	4.065196000	3.561684000	1.353742000
H	2.971307000	3.310127000	2.735380000
H	2.338788000	3.791713000	1.151210000
C	2.424830000	-1.342238000	3.465463000
H	2.445440000	-0.680723000	4.332585000
H	3.216271000	-2.090537000	3.566509000
H	1.476942000	-1.890419000	3.473044000
C	3.763040000	2.311453000	-0.906549000
C	5.170680000	2.290624000	-0.996754000
C	5.796650000	3.161104000	-1.890587000
H	6.882819000	3.153590000	-1.960016000
C	5.060021000	4.028588000	-2.685118000
H	5.562276000	4.696653000	-3.379338000
C	3.676744000	4.037195000	-2.583236000
H	3.096353000	4.717902000	-3.202832000
C	3.000133000	3.191974000	-1.698084000
C	6.029156000	1.380827000	-0.140181000
H	5.366562000	0.745392000	0.459527000
C	6.896752000	0.463469000	-0.998372000
H	6.282335000	-0.209876000	-1.604597000
H	7.549363000	-0.146517000	-0.365089000
H	7.543012000	1.037157000	-1.671882000
C	6.890421000	2.191249000	0.828582000
H	7.584607000	2.846197000	0.289609000
H	7.488072000	1.530170000	1.465018000
H	6.283303000	2.824631000	1.483602000
C	1.487975000	3.283311000	-1.633122000
H	1.116138000	2.563744000	-0.888429000
C	0.860426000	2.933667000	-2.984169000
H	-0.230106000	2.870491000	-2.898678000
H	1.223355000	1.973471000	-3.359559000
H	1.092786000	3.700275000	-3.732955000
C	1.048071000	4.686775000	-1.211990000
H	1.466011000	4.988167000	-0.245680000
H	-0.041359000	4.742981000	-1.129070000
H	1.363098000	5.433205000	-1.950115000
C	2.942146000	-2.656266000	1.075253000
C	1.849270000	-3.541682000	1.110831000
C	2.103962000	-4.915616000	1.175657000
H	1.259838000	-5.601892000	1.205795000
C	3.397885000	-5.407350000	1.196958000
H	3.578963000	-6.477475000	1.251648000
C	4.467411000	-4.521925000	1.131458000
H	5.479029000	-4.915915000	1.126174000
C	4.271556000	-3.140576000	1.061975000
C	0.409441000	-3.084594000	1.052533000
H	0.381863000	-1.981700000	1.042704000
C	-0.250300000	-3.566996000	-0.241610000
H	-0.323528000	-4.662483000	-0.256469000
H	0.319666000	-3.252276000	-1.122128000
H	-1.261222000	-3.149997000	-0.337311000
C	-0.399952000	-3.551732000	2.261734000
H	-0.599568000	-4.629686000	2.219130000
H	-1.365396000	-3.036145000	2.286576000
H	0.108631000	-3.354007000	3.211449000
C	5.465417000	-2.204883000	0.973310000
H	5.187342000	-1.405611000	0.275857000
C	6.705994000	-2.885864000	0.404776000
H	7.115026000	-3.635590000	1.091377000
H	7.497222000	-2.150624000	0.236241000
H	6.500212000	-3.379309000	-0.550117000
C	5.811940000	-1.555747000	2.315444000
H	5.025148000	-0.887188000	2.676265000
H	6.724722000	-0.955780000	2.221056000
H	5.995136000	-2.314361000	3.085929000
Al	-2.884467000	0.019207000	0.381242000
N	-4.696201000	-0.518485000	-0.185998000
N	-3.626722000	1.497043000	1.429961000
C	-5.867676000	-0.019631000	0.225069000
C	-5.962188000	0.986957000	1.196967000
H	-6.962517000	1.270752000	1.504120000
C	-4.907607000	1.725874000	1.745384000
C	-7.144071000	-0.533466000	-0.375073000
H	-7.295139000	-1.593810000	-0.146644000
H	-7.111550000	-0.464373000	-1.467146000
H	-8.007650000	0.024685000	-0.011364000
C	-5.243551000	2.841961000	2.690087000
H	-4.614774000	2.795938000	3.584884000
H	-6.292703000	2.810301000	2.987390000
H	-5.046517000	3.817592000	2.232056000
C	-4.680767000	-1.515422000	-1.221313000
C	-4.335181000	-1.133279000	-2.532104000
C	-4.240951000	-2.129919000	-3.507579000
H	-3.981960000	-1.847091000	-4.525794000
C	-4.467569000	-3.463541000	-3.200290000

H	-4.383174000	-4.224016000	-3.971570000
C	-4.802980000	-3.823183000	-1.900219000
H	-4.973460000	-4.869903000	-1.660776000
C	-4.917829000	-2.865505000	-0.891838000
C	-4.107284000	0.312597000	-2.930057000
H	-4.105390000	0.922204000	-2.016187000
C	-2.758813000	0.516750000	-3.616147000
H	-2.687453000	-0.057236000	-4.546964000
H	-1.938328000	0.206652000	-2.954828000
H	-2.613058000	1.572759000	-3.870195000
C	-5.250537000	0.818344000	-3.810610000
H	-5.105792000	1.872170000	-4.071539000
H	-6.222123000	0.726306000	-3.313428000
H	-5.305956000	0.250668000	-4.746580000
C	-5.211976000	-3.290906000	0.533959000
H	-5.633109000	-2.430034000	1.067663000
C	-6.225531000	-4.426513000	0.628918000
H	-7.145441000	-4.202216000	0.078882000
H	-6.493018000	-4.612962000	1.673405000
H	-5.825554000	-5.365247000	0.231624000
C	-3.909172000	-3.658114000	1.241356000
H	-4.080031000	-3.912984000	2.293201000
H	-3.193857000	-2.827530000	1.199598000
H	-3.429567000	-4.517199000	0.757359000
C	-2.618644000	2.431111000	1.842210000
C	-1.737448000	2.095530000	2.885556000
C	-0.689544000	2.975169000	3.175243000
H	-0.000126000	2.727144000	3.981105000
C	-0.520111000	4.152398000	2.461074000
H	0.296839000	4.827853000	2.703781000
C	-1.405744000	4.472181000	1.437231000
H	-1.268702000	5.397184000	0.882890000
C	-2.459243000	3.622045000	1.101217000
C	-1.905541000	0.840071000	3.718027000
H	-2.733288000	0.258184000	3.291810000
C	-0.659084000	-0.038470000	3.678182000
H	0.201041000	0.473210000	4.126505000
H	-0.396112000	-0.298551000	2.643539000
H	-0.821082000	-0.964818000	4.241495000
C	-2.277392000	1.187407000	5.159758000
H	-2.433846000	0.280645000	5.752907000
H	-3.191821000	1.787153000	5.214405000
H	-1.479606000	1.764151000	5.641819000
C	-3.355505000	3.938007000	-0.082705000
H	-4.308616000	3.413371000	0.056964000
C	-2.736510000	3.400746000	-1.374658000
H	-1.847618000	3.982110000	-1.644018000
H	-3.442785000	3.468045000	-2.210573000
H	-2.411389000	2.355405000	-1.275188000
C	-3.672850000	5.423499000	-0.219594000
H	-2.783758000	6.006233000	-0.484791000
H	-4.079340000	5.844529000	0.705818000
H	-4.406019000	5.585458000	-1.015452000
H	0.294845000	0.464091000	-1.988213000
C	1.074196000	-0.317054000	-1.868370000
C	1.144706000	-1.197280000	-2.927022000
O	3.782866000	-1.262345000	-1.613334000
C	2.212066000	-2.113107000	-3.199028000
H	0.342035000	-1.195655000	-3.674176000
C	3.441755000	-2.095722000	-2.589361000
H	2.083929000	-2.822098000	-4.011879000
C	4.537264000	-3.030460000	-2.980162000
H	4.277014000	-3.633857000	-3.851829000
H	5.457710000	-2.474332000	-3.190479000
H	4.763343000	-3.705777000	-2.144886000

Int10'_M06L.log

SCF (M06L) = -2685.80715878
E(SCF)+ZPE(0 K)= -2684.582156
H(298 K)= -2684.517594
G(298 K)= -2684.678921
Lowest Frequency = 8.7678 cm-1

Pd	0.944802000	-0.025659000	-1.140920000
Al	-1.855507000	-0.042827000	-0.750617000
N	-1.534367000	-1.300890000	0.665725000
N	-1.955573000	1.503073000	0.373478000
C	-0.875541000	-0.934865000	1.769686000
C	-0.677992000	0.410866000	2.115537000
H	-0.092289000	0.594629000	3.010281000
C	-1.266777000	1.543179000	1.525011000
C	-0.336642000	-1.997524000	2.675988000
H	-1.126275000	-2.665635000	3.033200000
H	0.184950000	-1.572929000	3.536209000
H	0.362745000	-2.628654000	2.113709000
C	-1.118244000	2.851786000	2.242304000
H	-0.641622000	2.718211000	3.214680000
H	-2.088425000	3.338657000	2.382302000
H	-0.509995000	3.547487000	1.653980000
C	-2.090580000	-2.622515000	0.564402000
C	-3.373211000	-2.833516000	1.118520000
C	-3.955777000	-4.094836000	0.986819000
H	-4.935565000	-4.273554000	1.424561000
C	-3.314746000	-5.117505000	0.298782000
H	-3.789759000	-6.089237000	0.194803000
C	-2.064609000	-4.887275000	-0.254355000
H	-1.559733000	-5.683867000	-0.797315000
C	-1.423787000	-3.650178000	-0.126952000
C	-4.120373000	-1.743868000	1.864340000
H	-3.618546000	-0.790219000	1.661586000
C	-5.561371000	-1.606440000	1.383859000
H	-5.594983000	-1.346736000	0.321684000
H	-6.074288000	-0.817853000	1.945113000
H	-6.136198000	-2.526944000	1.532230000
C	-4.080886000	-1.975301000	3.375566000
H	-4.549575000	-2.930478000	3.640018000
H	-4.620881000	-1.182247000	3.904322000
H	-3.057570000	-1.991180000	3.763511000
C	-0.045619000	-3.484640000	-0.730860000
H	0.322029000	-2.471129000	-0.490079000
C	-0.084559000	-3.603673000	-2.253076000
H	0.897641000	-3.369102000	-2.679445000
H	-0.811630000	-2.916398000	-2.692338000
H	-0.351313000	-4.622803000	-2.558803000
C	0.931134000	-4.510221000	-0.154052000
H	1.002291000	-4.449536000	0.938257000
H	1.936373000	-4.365397000	-0.565578000
H	0.621487000	-5.531517000	-0.403292000
C	-2.595431000	2.699048000	-0.112741000
C	-1.853132000	3.674865000	-0.807925000
C	-2.521263000	4.816997000	-1.260644000
H	-1.957420000	5.572933000	-1.803354000
C	-3.877762000	4.995388000	-1.043388000
H	-4.376851000	5.892406000	-1.399609000
C	-4.601205000	4.009209000	-0.384779000
H	-5.668775000	4.142213000	-0.238304000
C	-3.987437000	2.844428000	0.080403000
C	-0.380007000	3.532329000	-1.136211000
H	0.014722000	2.625440000	-0.647396000
C	-0.184801000	3.362227000	-2.643842000
H	-0.439410000	4.288861000	-3.172392000
H	-0.812877000	2.562815000	-3.043600000
H	0.859580000	3.117299000	-2.870578000
C	0.428852000	4.739381000	-0.662491000
H	0.156458000	5.641415000	-1.222081000
H	1.500451000	4.573586000	-0.822135000
H	0.276498000	4.962640000	0.398393000
C	-4.803656000	1.773486000	0.780356000
H	-4.403135000	0.803250000	0.461739000
C	-6.274791000	1.797627000	0.380800000

H	-6.794737000	2.680682000	0.769235000
H	-6.790539000	0.922163000	0.784829000
H	-6.396385000	1.786696000	-0.706821000
C	-4.666016000	1.856779000	2.302058000
H	-3.645817000	1.644412000	2.637825000
H	-5.324394000	1.128295000	2.789294000
H	-4.945779000	2.851447000	2.669374000
H	-0.019055000	0.517794000	-2.837346000
C	-0.921356000	-0.013527000	-2.470971000
C	-1.626074000	-0.555418000	-3.520409000
O	-3.493351000	-0.574852000	-1.256610000
C	-2.926979000	-1.153398000	-3.492141000
H	-1.179202000	-0.513997000	-4.519833000
C	-3.784725000	-1.129980000	-2.423837000
H	-3.297378000	-1.603601000	-4.408336000
C	-5.150757000	-1.727168000	-2.482070000
H	-5.393623000	-2.107596000	-3.475987000
H	-5.229442000	-2.552264000	-1.762771000
H	-5.903903000	-0.985470000	-2.191083000
P	2.916877000	0.264008000	-0.063831000
C	4.291497000	-0.137359000	-1.283214000
C	5.697678000	-0.464589000	-0.765606000
C	3.842732000	-1.226077000	-2.271074000
H	4.359602000	0.801628000	-1.861588000
C	6.688088000	-0.606449000	-1.919461000
H	5.667464000	-1.413759000	-0.210495000
H	6.053230000	0.291625000	-0.059959000
C	4.835027000	-1.391904000	-3.416137000
H	3.737165000	-2.181405000	-1.733027000
H	2.839616000	-0.985800000	-2.649756000
C	6.241943000	-1.677187000	-2.906153000
H	7.688362000	-0.829866000	-1.530426000
H	6.768702000	0.359455000	-2.441048000
H	4.499754000	-2.185993000	-4.093103000
H	4.848562000	-0.466378000	-4.010914000
H	6.949350000	-1.753763000	-3.739773000
H	6.252111000	-2.654862000	-2.401566000
C	3.264571000	2.042570000	0.432128000
C	2.448499000	2.398267000	1.682328000
C	4.727427000	2.488094000	0.544057000
H	2.823336000	2.610005000	-0.404989000
C	2.632815000	3.847097000	2.123475000
H	2.736012000	1.743300000	2.517386000
H	1.391301000	2.177653000	1.483070000
C	4.838286000	3.952665000	0.957761000
H	5.262902000	1.875860000	1.280544000
H	5.239264000	2.332159000	-0.413291000
C	4.104906000	4.208265000	2.267578000
H	2.092005000	4.020749000	3.062027000
H	2.181702000	4.516992000	1.380532000
H	5.892940000	4.240360000	1.036090000
H	4.401678000	4.587762000	0.171984000
H	4.215925000	5.253233000	2.577934000
H	4.560155000	3.598314000	3.062925000
C	3.219317000	-0.735876000	1.498826000
C	3.157477000	-2.227545000	1.154209000
C	4.431173000	-0.431166000	2.388482000
H	2.313690000	-0.508319000	2.090440000
C	3.233577000	-3.126054000	2.384048000
H	3.991778000	-2.482988000	0.483901000
H	2.242561000	-2.418662000	0.580465000
C	4.447560000	-1.327339000	3.624836000
H	5.361488000	-0.586398000	1.828748000
H	4.432307000	0.618379000	2.699516000
C	4.450160000	-2.800230000	3.239761000
H	3.241105000	-4.178570000	2.075868000
H	2.328550000	-2.990741000	2.994016000
H	5.314214000	-1.085702000	4.250851000
H	3.556079000	-1.115465000	4.235832000
H	4.476917000	-3.436203000	4.131718000
H	5.366553000	-3.022691000	2.672511000

TS-3_M06L.log

SCF (M06L) = -2879.88387107
E(SCF)+ZPE(0 K)= -2878.510984
H(298 K)= -2878.430723
G(298 K)= -2878.623263
Lowest Frequency = -769.9919 cm-1

Pd	-0.064741000	-0.284203000	1.306081000
Al	-1.768795000	0.053143000	-0.404700000
N	-2.852266000	1.705871000	-0.334343000
N	-2.481123000	-0.404534000	-2.181120000
C	-3.557016000	2.247891000	-1.333169000
C	-3.687756000	1.639514000	-2.591237000
H	-4.289306000	2.172712000	-3.319302000
C	-3.280881000	0.351275000	-2.951213000
C	-4.331825000	3.515316000	-1.108964000
H	-5.284775000	3.294230000	-0.613737000
H	-4.558009000	4.006399000	-2.057389000
H	-3.799272000	4.209862000	-0.454961000
C	-3.844166000	-0.211306000	-4.225242000
H	-3.111244000	-0.793058000	-4.789294000
H	-4.244712000	0.580434000	-4.860444000
H	-4.664324000	-0.901531000	-3.994228000
C	-3.011023000	2.264139000	0.985476000
C	-4.200509000	2.000126000	1.694292000
C	-4.344278000	2.541430000	2.972251000
H	-5.255987000	2.345242000	3.532123000
C	-3.337022000	3.303509000	3.547756000
H	-3.464397000	3.709521000	4.547891000
C	-2.158906000	3.524247000	2.849918000
H	-1.363514000	4.102576000	3.311042000
C	-1.967345000	3.009966000	1.565131000
C	-5.265898000	1.064285000	1.156803000
H	-5.165039000	0.999317000	0.065851000
C	-5.031622000	-0.338550000	1.718643000
H	-4.022910000	-0.707147000	1.491087000
H	-5.760391000	-1.050403000	1.313537000
H	-5.125268000	-0.340810000	2.810795000
C	-6.688104000	1.533125000	1.449444000
H	-6.917083000	1.498233000	2.519737000
H	-7.414919000	0.886433000	0.948150000
H	-6.860019000	2.560538000	1.111077000
C	-0.691609000	3.308349000	0.807061000
H	-0.484694000	2.447273000	0.155656000
C	0.526641000	3.454843000	1.709076000
H	1.429669000	3.519241000	1.093543000
H	0.630877000	2.590104000	2.374553000
H	0.487487000	4.366876000	2.317576000
C	-0.864371000	4.539922000	-0.081543000
H	-1.645112000	4.391703000	-0.835321000
H	0.067792000	4.771003000	-0.608844000
H	-1.139755000	5.416355000	0.517970000
C	-2.238221000	-1.757654000	-2.602567000
C	-1.057719000	-2.065334000	-3.310713000
C	-0.893602000	-3.369844000	-3.783671000
H	-0.003366000	-3.617816000	-4.356876000
C	-1.842042000	-4.354784000	-3.537098000
H	-1.694979000	-5.361579000	-3.918156000
C	-2.960691000	-4.051183000	-2.774338000
H	-3.679567000	-4.832421000	-2.535958000
C	-3.176397000	-2.760028000	-2.285271000
C	-0.008125000	-1.012830000	-3.614650000
H	-0.132863000	-0.200822000	-2.884770000
C	1.420028000	-1.528731000	-3.467452000
H	1.645116000	-2.351605000	-4.155019000
H	1.624913000	-1.882924000	-2.451626000

H	2.137188000	-0.730582000	-3.689841000
C	-0.209283000	-0.423535000	-5.011071000
H	-0.172988000	-1.209131000	-5.774995000
H	0.580535000	0.299704000	-5.242405000
H	-1.169660000	0.092122000	-5.108955000
C	-4.354812000	-2.516230000	-1.361436000
H	-4.434794000	-1.436812000	-1.171894000
C	-4.093389000	-3.209959000	-0.021335000
H	-3.914533000	-4.281376000	-0.169907000
H	-4.947615000	-3.102860000	0.653914000
H	-3.212066000	-2.795723000	0.482584000
C	-5.681349000	-2.979579000	-1.958668000
H	-5.877850000	-2.523879000	-2.934818000
H	-6.511527000	-2.720051000	-1.293891000
H	-5.704544000	-4.066361000	-2.094662000
Al	1.968415000	0.001263000	0.025506000
N	3.123744000	-1.607491000	-0.040038000
N	3.162500000	0.966063000	-1.202306000
C	4.133484000	-1.839170000	-0.879432000
C	4.515826000	-0.918826000	-1.870445000
H	5.295316000	-1.242193000	-2.551291000
C	4.114833000	0.414257000	-1.978615000
C	4.959212000	-3.085357000	-0.748413000
H	4.361037000	-3.946760000	-0.442919000
H	5.722914000	-2.944590000	0.025542000
H	5.476767000	-3.311544000	-1.682696000
C	4.819783000	1.265666000	-2.994021000
H	5.276784000	2.144241000	-2.526787000
H	4.111478000	1.649585000	-3.736286000
H	5.594643000	0.701425000	-3.514300000
C	2.910079000	-2.508961000	1.062228000
C	3.737518000	-2.418985000	2.196502000
C	3.472195000	-3.260765000	3.279644000
H	4.097280000	-3.191481000	4.167672000
C	2.417636000	-4.160493000	3.248744000
H	2.222195000	-4.803798000	4.102092000
C	1.597640000	-4.219861000	2.128911000
H	0.758529000	-4.910000000	2.116796000
C	1.813768000	-3.393955000	1.024626000
C	4.838825000	-1.385111000	2.328338000
H	4.982979000	-0.893971000	1.356285000
C	4.418112000	-0.309979000	3.331475000
H	4.274645000	-0.745580000	4.326548000
H	3.469711000	0.161290000	3.046466000
H	5.186423000	0.467352000	3.415922000
C	6.175588000	-1.998859000	2.739948000
H	6.964216000	-1.239160000	2.749686000
H	6.489441000	-2.801715000	2.064741000
H	6.125362000	-2.425276000	3.747668000
C	0.914853000	-3.485046000	-0.191179000
H	0.831653000	-2.472449000	-0.614686000
C	1.527076000	-4.396860000	-1.254884000
H	2.485409000	-4.014512000	-1.623125000
H	0.855317000	-4.489998000	-2.114969000
H	1.700569000	-5.401472000	-0.850584000
C	-0.507810000	-3.912187000	0.138978000
H	-1.137290000	-3.813486000	-0.751111000
H	-0.932875000	-3.279592000	0.927320000
H	-0.567232000	-4.959561000	0.459819000
C	3.051037000	2.401741000	-1.202909000
C	2.282178000	3.079399000	-2.167995000
C	2.251795000	4.477159000	-2.131900000
H	1.654757000	5.010245000	-2.869130000
C	2.956913000	5.191907000	-1.176031000
H	2.927412000	6.277893000	-1.171947000
C	3.677298000	4.509010000	-0.204364000
H	4.195430000	5.071434000	0.566570000
C	3.723796000	3.113590000	-0.181933000
C	1.459803000	2.366801000	-3.224140000
H	1.722329000	1.300771000	-3.201352000
C	-0.032610000	2.485438000	-2.908978000
H	-0.344230000	3.536080000	-2.871869000
H	-0.270733000	2.038984000	-1.934641000
H	-0.642003000	1.984488000	-3.669571000
C	1.742268000	2.902134000	-4.628163000
H	1.188301000	2.333105000	-5.381420000
H	2.805431000	2.856562000	-4.885745000
H	1.430193000	3.947092000	-4.727023000
C	4.459221000	2.392511000	0.934644000
H	3.885884000	1.478430000	1.161671000
C	4.519307000	3.204646000	2.223424000
H	5.185286000	4.069571000	2.130312000
H	4.910451000	2.589870000	3.038308000
H	3.531239000	3.567755000	2.521970000
C	5.864685000	1.952026000	0.522121000
H	6.472563000	2.817492000	0.232826000
H	5.854055000	1.249749000	-0.316663000
H	6.370812000	1.455385000	1.358202000
H	0.587039000	-0.396312000	2.785485000
C	-1.037649000	-0.924439000	3.018793000
C	-1.242336000	-2.158349000	3.590570000
O	-1.779941000	0.002105000	3.727816000
C	-2.174544000	-1.988241000	4.656806000
H	-0.763930000	-3.076492000	3.275200000
C	-2.470556000	-0.659826000	4.711397000
H	-2.569746000	-2.750107000	5.316661000
C	-3.338167000	0.157787000	5.583610000
H	-3.793766000	-0.471390000	6.352090000
H	-2.779068000	0.956128000	6.087499000
H	-4.145523000	0.650275000	5.025465000

TS-3'_M06L.log

SCF (M06L) = -2685.70739882
E(SCF)+ZPE(0 K)= -2684.489977
H(298 K)= -2684.424282
G(298 K)= -2684.590835
Lowest Frequency = -764.7459 cm-1

Pd	0.894282000	-0.401541000	-0.983824000
Al	-1.472957000	0.006951000	-0.708790000
N	-2.589839000	1.545714000	-1.194015000
N	-3.006507000	-1.205063000	-0.871544000
C	-3.752874000	1.491095000	-1.856681000
C	-4.415737000	0.286538000	-2.125611000
H	-5.323525000	0.355552000	-2.713990000
C	-4.112437000	-0.972803000	-1.591247000
C	-4.416718000	2.762472000	-2.295792000
H	-5.217195000	2.562793000	-3.009491000
H	-3.697328000	3.455830000	-2.741169000
H	-4.851457000	3.286647000	-1.437269000
C	-5.094929000	-2.082961000	-1.818415000
H	-4.629349000	-2.904094000	-2.372884000
H	-5.966332000	-1.735749000	-2.374682000
H	-5.428820000	-2.511856000	-0.867861000
C	-2.121294000	2.830586000	-0.751921000
C	-2.696552000	3.398765000	0.404760000
C	-2.254388000	4.655514000	0.820638000
H	-2.688661000	5.100504000	1.714104000
C	-1.263872000	5.336311000	0.123821000
H	-0.936077000	6.317022000	0.457743000
C	-0.678173000	4.743017000	-0.985348000
H	0.113211000	5.263272000	-1.521268000
C	-1.081050000	3.483024000	-1.439715000
C	-3.721243000	2.660818000	1.246165000
H	-4.047991000	1.767535000	0.698463000
C	-3.074844000	2.189209000	2.549622000
H	-2.204885000	1.546478000	2.363487000
H	-3.789807000	1.627023000	3.161280000
H	-2.728958000	3.043286000	3.143974000

C	-4.961000000	3.500713000	1.544018000
H	-4.718985000	4.371311000	2.163262000
H	-5.703915000	2.911079000	2.090968000
H	-5.436282000	3.873005000	0.630867000
C	-0.395290000	2.886748000	-2.651750000
H	-0.769004000	1.864844000	-2.791680000
C	1.113763000	2.787759000	-2.437259000
H	1.596784000	2.286561000	-3.280565000
H	1.342993000	2.194197000	-1.541818000
H	1.569649000	3.778688000	-2.321929000
C	-0.716716000	3.682255000	-3.916290000
H	-1.793153000	3.719513000	-4.115580000
H	-0.230309000	3.235733000	-4.788974000
H	-0.362437000	4.716434000	-3.832705000
C	-2.874708000	-2.472449000	-0.205592000
C	-2.367934000	-3.594570000	-0.886043000
C	-2.289499000	-4.807537000	-0.194419000
H	-1.896677000	-5.681480000	-0.710193000
C	-2.693405000	-4.911587000	1.127454000
H	-2.635636000	-5.865279000	1.644852000
C	-3.141832000	-3.780511000	1.800530000
H	-3.416827000	-3.859751000	2.848658000
C	-3.219048000	-2.541047000	1.163037000
C	-1.833965000	-3.522581000	-2.302067000
H	-2.043971000	-2.521430000	-2.699558000
C	-0.315524000	-3.705919000	-2.285846000
H	-0.051790000	-4.717580000	-1.953666000
H	0.163135000	-2.986588000	-1.609970000
H	0.112972000	-3.553192000	-3.281622000
C	-2.479158000	-4.551083000	-3.229528000
H	-2.246206000	-5.573513000	-2.911935000
H	-2.102378000	-4.436417000	-4.250340000
H	-3.570275000	-4.463870000	-3.263949000
C	-3.637513000	-1.299177000	1.931077000
H	-3.019459000	-0.467628000	1.552112000
C	-3.370330000	-1.407747000	3.428038000
H	-4.045758000	-2.122167000	3.911532000
H	-3.531663000	-0.440453000	3.912702000
H	-2.343040000	-1.722517000	3.638166000
C	-5.098933000	-0.917344000	1.688264000
H	-5.298364000	-0.669091000	0.641940000
H	-5.372347000	-0.041894000	2.289187000
H	-5.766683000	-1.737769000	1.976372000
H	0.669847000	-0.721216000	-2.556919000
C	2.283944000	-0.972088000	-2.428406000
C	2.919859000	-2.166145000	-2.682450000
O	2.893860000	-0.001255000	-3.208504000
C	3.974158000	-1.910359000	-3.610331000
H	2.662709000	-3.116311000	-2.232190000
C	3.922098000	-0.581528000	-3.904369000
H	4.686315000	-2.619094000	-4.012786000
C	4.724316000	0.315404000	-4.761259000
H	5.520322000	-0.247452000	-5.253516000
H	4.119401000	0.794670000	-5.540382000
H	5.191988000	1.122009000	-4.182758000
P	2.208973000	-0.195449000	1.002071000
C	3.990944000	-0.501654000	0.553163000
C	4.538461000	0.670105000	-0.271484000
C	4.992900000	-0.945312000	1.622632000
H	3.886942000	-1.343151000	-0.153977000
C	5.883039000	0.315888000	-0.897242000
H	4.663775000	1.552175000	0.375624000
H	3.814913000	0.946635000	-1.049572000
C	6.334079000	-1.295022000	0.980102000
H	5.146306000	-0.150417000	2.366271000
H	4.610588000	-1.813252000	2.174833000
C	6.883307000	-0.132033000	0.162274000
H	6.275538000	1.172117000	-1.458938000
H	5.728953000	-0.491591000	-1.628634000
H	7.053215000	-1.602522000	1.748557000
H	6.194752000	-2.163343000	0.319421000
H	7.839203000	-0.406302000	-0.298853000
H	7.097482000	0.711391000	0.836735000
C	1.855573000	-1.484561000	2.309035000
C	2.282659000	-1.161655000	3.744465000
C	0.393211000	-1.949423000	2.283955000
H	2.463304000	-2.339551000	1.963440000
C	2.067268000	-2.354016000	4.671681000
H	1.683980000	-0.313960000	4.110489000
H	3.328144000	-0.837432000	3.779270000
C	0.171367000	-3.129756000	3.223370000
H	-0.259102000	-1.107327000	2.574727000
H	0.097595000	-2.207209000	1.257902000
C	0.616446000	-2.816502000	4.646044000
H	2.377410000	-2.100013000	5.692086000
H	2.718180000	-3.180442000	4.348054000
H	-0.881627000	-3.434084000	3.201258000
H	0.741601000	-3.992728000	2.847297000
H	0.475910000	-3.687033000	5.297182000
H	-0.021002000	-2.017962000	5.056365000
C	2.165279000	1.517320000	1.750967000
C	0.756936000	1.895129000	2.224628000
C	3.207926000	1.922415000	2.800780000
H	2.364403000	2.133231000	0.855579000
C	0.665996000	3.380068000	2.555357000
H	0.497961000	1.301100000	3.116937000
H	0.025128000	1.628291000	1.450266000
C	3.114027000	3.415997000	3.103451000
H	3.045480000	1.362524000	3.729730000
H	4.219047000	1.674163000	2.461956000
C	1.716685000	3.796824000	3.576353000
H	-0.343410000	3.628414000	2.906758000
H	0.808907000	3.953212000	1.627075000
H	3.866640000	3.697353000	3.849342000
H	3.355382000	3.982321000	2.191212000
H	1.656376000	4.872979000	3.776522000
H	1.513013000	3.292903000	4.533422000

TS-4_M06L.log

SCF (M06L) = -2879.89521711
E(SCF)+ZPE(0 K)= -2878.523333
H(298 K)= -2878.443198
G(298 K)= -2878.636069
Lowest Frequency = -103.8561-cm-1

Pd	0.039262000	0.004932000	0.415743000
Al	-2.226796000	-0.530945000	0.674718000
N	-3.189951000	-2.125865000	0.243176000
N	-3.751919000	0.408239000	1.368299000
C	-4.359179000	-2.465964000	0.800586000
C	-5.125220000	-1.569849000	1.556086000
H	-6.048805000	-1.956805000	1.970419000
C	-4.878690000	-0.205071000	1.760352000
C	-4.886119000	-3.856035000	0.605053000
H	-5.164532000	-4.035809000	-0.438557000
H	-5.761377000	-4.037187000	1.229823000
H	-4.114820000	-4.595270000	0.843293000
C	-5.946650000	0.596071000	2.440741000
H	-5.536501000	1.187807000	3.264652000
H	-6.741647000	-0.046492000	2.819808000
H	-6.387686000	1.317022000	1.744223000
C	-2.563992000	-3.020816000	-0.692699000
C	-3.053603000	-3.059096000	-2.016334000
C	-2.383341000	-3.867143000	-2.933532000
H	-2.742498000	-3.923909000	-3.957462000
C	-1.256404000	-4.596418000	-2.565979000
H	-0.739852000	-5.205130000	-3.303621000
C	-0.807261000	-4.558581000	-1.255645000

H	0.058134000	-5.149364000	-0.960819000
C	-1.454091000	-3.780614000	-0.289736000
C	-4.257900000	-2.230585000	-2.430613000
H	-4.954118000	-2.199678000	-1.581952000
C	-3.878854000	-0.781409000	-2.732449000
H	-3.308840000	-0.315858000	-1.919090000
H	-4.778233000	-0.178210000	-2.901363000
H	-3.251440000	-0.708617000	-3.626243000
C	-5.021696000	-2.827865000	-3.607109000
H	-4.442600000	-2.771659000	-4.535645000
H	-5.948458000	-2.272374000	-3.778580000
H	-5.281350000	-3.878557000	-3.440120000
C	-0.980419000	-3.839213000	1.149587000
H	-1.557779000	-3.112987000	1.735350000
C	0.492654000	-3.474293000	1.285862000
H	0.805777000	-3.500760000	2.335488000
H	0.696649000	-2.463294000	0.905686000
H	1.131327000	-4.175678000	0.737095000
C	-1.241024000	-5.226110000	1.741087000
H	-2.297435000	-5.510589000	1.685418000
H	-0.938688000	-5.262237000	2.792594000
H	-0.668951000	-5.992837000	1.205887000
C	-3.638349000	1.829129000	1.579979000
C	-2.980664000	2.306143000	2.731825000
C	-2.894188000	3.688747000	2.914370000
H	-2.394815000	4.069318000	3.803639000
C	-3.422580000	4.576610000	1.988021000
H	-3.337542000	5.648196000	2.147366000
C	-4.056485000	4.086757000	0.854067000
H	-4.468574000	4.780043000	0.123359000
C	-4.185385000	2.714161000	0.629118000
C	-2.402871000	1.391089000	3.795112000
H	-2.526522000	0.353485000	3.463695000
C	-0.906602000	1.627177000	3.989665000
H	-0.706066000	2.646066000	4.343764000
H	-0.351343000	1.467698000	3.057896000
H	-0.505651000	0.931854000	4.734742000
C	-3.146678000	1.552628000	5.121453000
H	-3.042665000	2.571412000	5.512204000
H	-2.745101000	0.868619000	5.875807000
H	-4.218279000	1.348601000	5.023450000
C	-4.903792000	2.241104000	-0.620947000
H	-4.984865000	1.146678000	-0.581766000
C	-4.106041000	2.604555000	-1.871984000
H	-3.964860000	3.689895000	-1.939158000
H	-4.630584000	2.282723000	-2.778133000
H	-3.117105000	2.136263000	-1.873492000
C	-6.318635000	2.812944000	-0.716712000
H	-6.920719000	2.602027000	0.173374000
H	-6.842826000	2.394931000	-1.582109000
H	-6.299386000	3.901023000	-0.843697000
Al	2.403912000	0.589545000	0.470725000
N	3.343157000	2.080065000	-0.414695000
N	4.122369000	-0.284711000	0.878738000
C	4.639072000	2.396551000	-0.286360000
C	5.565007000	1.565936000	0.356867000
H	6.584495000	1.930856000	0.410204000
C	5.341719000	0.268203000	0.831843000
C	5.157926000	3.668662000	-0.891677000
H	4.495144000	4.511047000	-0.672901000
H	5.199624000	3.591779000	-1.983950000
H	6.162067000	3.894798000	-0.529771000
C	6.532659000	-0.519084000	1.293716000
H	6.437503000	-0.790135000	2.350925000
H	7.457105000	0.043949000	1.160036000
H	6.611557000	-1.464919000	0.748215000
C	2.546951000	2.890763000	-1.297994000
C	2.675786000	2.696658000	-2.690697000
C	1.913792000	3.492640000	-3.544501000
H	1.994784000	3.348001000	-4.618888000
C	1.042406000	4.454612000	-3.046255000
H	0.459444000	5.068209000	-3.727972000
C	0.909194000	4.613494000	-1.675601000
H	0.214551000	5.353208000	-1.281747000
C	1.646014000	3.834171000	-0.777073000
C	3.551996000	1.593153000	-3.252042000
H	4.362414000	1.394010000	-2.538634000
C	2.729052000	0.310845000	-3.380381000
H	1.925663000	0.440973000	-4.114057000
H	2.237355000	0.041568000	-2.435678000
H	3.356907000	-0.530814000	-3.697100000
C	4.203903000	1.948957000	-4.583886000
H	4.911060000	1.168560000	-4.881131000
H	4.748746000	2.897815000	-4.536009000
H	3.466763000	2.031562000	-5.389612000
C	1.438881000	4.041563000	0.708159000
H	2.085543000	3.336791000	1.247711000
C	1.835284000	5.453147000	1.138407000
H	2.878376000	5.678282000	0.892241000
H	1.709789000	5.580522000	2.218497000
H	1.211963000	6.207281000	0.644459000
C	-0.004038000	3.733985000	1.100261000
H	-0.154568000	3.874854000	2.175852000
H	-0.269277000	2.693997000	0.855083000
H	-0.712750000	4.390843000	0.581357000
C	4.022091000	-1.659446000	1.288042000
C	3.990096000	-1.986606000	2.658559000
C	3.930571000	-3.334454000	3.017464000
H	3.903903000	-3.600264000	4.072092000
C	3.897737000	-4.336121000	2.055252000
H	3.860221000	-5.380205000	2.354318000
C	3.882079000	-3.993172000	0.710871000
H	3.816171000	-4.773633000	-0.045453000
C	3.927157000	-2.658071000	0.300116000
C	3.947217000	-0.920206000	3.734898000
H	4.232229000	0.035851000	3.278284000
C	2.515817000	-0.774112000	4.258119000
H	2.189782000	-1.701381000	4.744930000
H	1.809204000	-0.560725000	3.446932000
H	2.447769000	0.033710000	4.995232000
C	4.917248000	-1.192182000	4.881605000
H	4.916347000	-0.359236000	5.591400000
H	5.944944000	-1.332536000	4.529416000
H	4.639115000	-2.091036000	5.442453000
C	3.835600000	-2.341628000	-1.180562000
H	3.955050000	-1.257509000	-1.309152000
C	2.456488000	-2.727593000	-1.719929000
H	2.257154000	-3.793774000	-1.554972000
H	2.386299000	-2.538920000	-2.796639000
H	1.651388000	-2.159723000	-1.235235000
C	4.946862000	-3.018430000	-1.980147000
H	4.869416000	-4.110447000	-1.930297000
H	5.940781000	-2.742307000	-1.613040000
H	4.888130000	-2.737063000	-3.036815000
H	-0.970820000	-0.645062000	1.917688000
C	-0.626233000	0.265485000	-1.539792000
C	-0.781898000	1.396716000	-2.311000000
O	-0.591366000	-0.820357000	-2.419033000
C	-0.810130000	1.007241000	-3.681747000
H	-0.809920000	2.407067000	-1.920951000
C	-0.684757000	-0.350405000	-3.701087000
H	-0.892163000	1.649636000	-4.550383000
C	-0.626734000	-1.352811000	-4.785555000
H	-0.537901000	-0.855790000	-5.754970000
H	0.231415000	-2.026024000	-4.666389000
H	-1.519876000	-1.993050000	-4.816788000

TS-4b_M06L.log

SCF (M06L) = -2879.90360819
E(SCF)+ZPE(0 K)= -2878.529165

H(298 K)= -2878.449953

G(298 K)= -2878.638655

Lowest Frequency = -114.7815 cm-1

Pd	-0.224542000	1.251892000	-0.306960000
Al	-1.447427000	-0.574854000	0.215410000
N	-2.236990000	-2.064315000	-0.702182000
N	-2.289435000	-0.961904000	1.893494000
C	-2.776456000	-3.125782000	-0.086629000
C	-2.903333000	-3.215280000	1.306220000
H	-3.277585000	-4.155528000	1.695153000
C	-2.749098000	-2.177034000	2.231769000
C	-3.331254000	-4.261992000	-0.892793000
H	-4.393453000	-4.090069000	-1.100019000
H	-3.258718000	-5.196644000	-0.332644000
H	-2.828900000	-4.374646000	-1.855188000
C	-3.159575000	-2.440886000	3.649127000
H	-2.323998000	-2.267073000	4.333768000
H	-3.511428000	-3.465513000	3.773829000
H	-3.952354000	-1.754818000	3.963738000
C	-2.362494000	-1.937850000	-2.131494000
C	-3.609230000	-1.572257000	-2.674847000
C	-3.685094000	-1.360472000	-4.053516000
H	-4.637844000	-1.064976000	-4.489645000
C	-2.569402000	-1.500923000	-4.864321000
H	-2.645533000	-1.319033000	-5.933028000
C	-1.352508000	-1.880007000	-4.309564000
H	-0.487511000	-1.996053000	-4.954688000
C	-1.217785000	-2.111376000	-2.939007000
C	-4.858921000	-1.366468000	-1.836439000
H	-4.656366000	-1.693606000	-0.808356000
C	-5.240106000	0.111515000	-1.771974000
H	-4.448446000	0.719558000	-1.325223000
H	-6.154076000	0.249013000	-1.181849000
H	-5.433318000	0.509348000	-2.775268000
C	-6.037144000	-2.187875000	-2.361076000
H	-6.375875000	-1.818682000	-3.335206000
H	-6.888685000	-2.118037000	-1.676101000
H	-5.788233000	-3.246659000	-2.487455000
C	0.103140000	-2.579818000	-2.350761000
H	0.300891000	-1.972299000	-1.451127000
C	1.290490000	-2.383765000	-3.283357000
H	2.216983000	-2.624768000	-2.751836000
H	1.371953000	-1.349456000	-3.629922000
H	1.227314000	-3.044163000	-4.157292000
C	0.045276000	-4.046082000	-1.914198000
H	-0.677999000	-4.220929000	-1.113630000
H	1.024922000	-4.365608000	-1.541938000
H	-0.221081000	-4.692777000	-2.759217000
C	-2.387223000	0.095791000	2.867352000
C	-1.352489000	0.309434000	3.796251000
C	-1.537090000	1.295735000	4.770456000
H	-0.749972000	1.464773000	5.503229000
C	-2.688733000	2.066053000	4.806957000
H	-2.815363000	2.824798000	5.574454000
C	-3.669241000	1.887250000	3.837103000
H	-4.549986000	2.522591000	3.846960000
C	-3.542645000	0.911125000	2.846522000
C	-0.037361000	-0.445306000	3.762963000
H	-0.066663000	-1.142350000	2.915687000
C	1.129471000	0.511363000	3.519774000
H	1.191535000	1.279134000	4.301070000
H	1.028043000	1.011193000	2.549826000
H	2.080997000	-0.032607000	3.514873000
C	0.219323000	-1.247292000	5.038599000
H	0.261249000	-0.592524000	5.916424000
H	1.183757000	-1.764040000	4.972542000
H	-0.548804000	-2.003328000	5.232655000
C	-4.602929000	0.760627000	1.766370000
H	-4.064874000	0.594177000	0.820020000
C	-5.448499000	2.016140000	1.579286000
H	-6.161671000	2.148124000	2.401717000
H	-6.028217000	1.937514000	0.654496000
H	-4.840483000	2.920897000	1.509059000
C	-5.541236000	-0.429784000	1.987004000
H	-5.037754000	-1.397144000	1.923392000
H	-6.329413000	-0.430030000	1.225221000
H	-6.032622000	-0.363099000	2.965376000
Al	1.997703000	0.131894000	-0.149886000
N	2.983194000	1.848923000	0.203043000
N	3.311426000	-0.883046000	0.912490000
C	3.875489000	2.060242000	1.156199000
C	4.402163000	1.010769000	1.944420000
H	5.115040000	1.303750000	2.708369000
C	4.196984000	-0.359366000	1.776998000
C	4.399781000	3.439458000	1.436644000
H	4.066867000	3.786632000	2.420746000
H	4.068005000	4.163062000	0.688961000
H	5.493335000	3.430437000	1.457067000
C	5.005757000	-1.295345000	2.630639000
H	4.346253000	-1.803775000	3.346197000
H	5.777350000	-0.766521000	3.192150000
H	5.478841000	-2.083584000	2.037529000
C	2.317735000	2.889169000	-0.523948000
C	2.556171000	2.979658000	-1.915654000
C	1.733104000	3.810173000	-2.678735000
H	1.897507000	3.880696000	-3.749941000
C	0.689662000	4.524045000	-2.101025000
H	0.031375000	5.129378000	-2.717570000
C	0.491525000	4.458891000	-0.729915000
H	-0.315537000	5.029021000	-0.274483000
C	1.300730000	3.664279000	0.092999000
C	3.705000000	2.214382000	-2.544105000
H	3.732725000	1.227593000	-2.067376000
C	3.531954000	1.976547000	-4.037239000
H	3.580930000	2.906563000	-4.615427000
H	2.574303000	1.491882000	-4.253747000
H	4.329108000	1.326277000	-4.410375000
C	5.041123000	2.897472000	-2.249790000
H	5.871615000	2.329144000	-2.684613000
H	5.223866000	2.977145000	-1.173230000
H	5.072837000	3.909247000	-2.671117000
C	1.107760000	3.757168000	1.597955000
H	1.765344000	3.021746000	2.077586000
C	1.502302000	5.154422000	2.088608000
H	2.504824000	5.448223000	1.765807000
H	1.467138000	5.207007000	3.182143000
H	0.803672000	5.904739000	1.701337000
C	-0.319346000	3.456638000	2.051027000
H	-0.393137000	3.509472000	3.143094000
H	-0.647459000	2.454517000	1.737929000
H	-1.031598000	4.178694000	1.635002000
C	3.527197000	-2.211011000	0.423595000
C	2.839794000	-3.313383000	0.960056000
C	3.045100000	-4.574746000	0.390725000
H	2.512389000	-5.432059000	0.799662000
C	3.913316000	-4.749974000	-0.677158000
H	4.064008000	-5.737789000	-1.104100000
C	4.590582000	-3.651876000	-1.196590000
H	5.275580000	-3.789160000	-2.030816000
C	4.408436000	-2.371064000	-0.672822000
C	1.900302000	-3.180763000	2.139404000
H	1.931223000	-2.134205000	2.473665000
C	0.463400000	-3.503667000	1.737545000
H	0.372539000	-4.530104000	1.360944000
H	0.112691000	-2.835280000	0.938104000
H	-0.219437000	-3.404567000	2.589369000
C	2.331911000	-4.071647000	3.304164000
H	1.670980000	-3.936510000	4.167059000
H	3.356792000	-3.857776000	3.623874000
H	2.292906000	-5.131437000	3.028618000
C	5.186856000	-1.204878000	-1.255563000

H	4.739280000	-0.277669000	-0.877996000
C	5.102219000	-1.153372000	-2.777626000
H	5.552416000	-2.034794000	-3.248187000
H	5.641005000	-0.277458000	-3.156748000
H	4.060406000	-1.079741000	-3.106721000
C	6.644835000	-1.232951000	-0.794502000
H	7.149819000	-2.145475000	-1.133132000
H	6.730489000	-1.192456000	0.296203000
H	7.195014000	-0.377290000	-1.201224000
H	2.391064000	-0.363522000	-1.623434000
C	-2.127813000	1.860296000	-1.027831000
C	-2.960692000	2.896760000	-0.658221000
O	-2.347955000	1.651069000	-2.396560000
C	-3.672813000	3.346506000	-1.806757000
H	-3.013473000	3.305576000	0.344202000
C	-3.261996000	2.555537000	-2.841866000
H	-4.400473000	4.145837000	-1.870826000
C	-3.626088000	2.479721000	-4.272701000
H	-4.340209000	3.265965000	-4.527640000
H	-2.749721000	2.590335000	-4.921615000
H	-4.079038000	1.511621000	-4.524500000

TS-4'_M06L.log

SCF (M06L) = -2685.7207341
E(SCF)+ZPE(0 K)= -2684.502535
H(298 K)= -2684.437698
G(298 K)= -2684.600315
Lowest Frequency = -48.8465 cm-1

Pd	0.487957000	-0.419299000	-0.020980000
Al	-1.686720000	-0.159733000	-0.753187000
N	-2.612929000	1.362077000	-1.434388000
N	-3.206874000	-1.302585000	-0.793366000
C	-3.748149000	1.292330000	-2.136186000
C	-4.448379000	0.089826000	-2.318566000
H	-5.314083000	0.128256000	-2.969434000
C	-4.234463000	-1.116190000	-1.635886000
C	-4.315053000	2.547118000	-2.722125000
H	-4.655155000	3.216717000	-1.923932000
H	-5.160473000	2.337320000	-3.377876000
H	-3.552950000	3.101122000	-3.279007000
C	-5.217729000	-2.228448000	-1.839403000
H	-4.713220000	-3.111898000	-2.245573000
H	-6.016443000	-1.934551000	-2.521000000
H	-5.659944000	-2.543225000	-0.889131000
C	-2.006051000	2.631012000	-1.131147000
C	-2.410125000	3.312892000	0.033070000
C	-1.697025000	4.453641000	0.407399000
H	-1.983584000	4.979515000	1.316247000
C	-0.630232000	4.918178000	-0.350952000
H	-0.080936000	5.802083000	-0.038258000
C	-0.273831000	4.252977000	-1.516677000
H	0.550453000	4.626951000	-2.121594000
C	-0.946081000	3.100055000	-1.929114000
C	-3.575619000	2.851157000	0.887124000
H	-4.044419000	1.987735000	0.396061000
C	-3.107859000	2.395168000	2.267461000
H	-2.398640000	1.564115000	2.205748000
H	-3.960441000	2.071507000	2.875743000
H	-2.612016000	3.212027000	2.804262000
C	-4.638755000	3.942125000	1.014377000
H	-4.253724000	4.810142000	1.560941000
H	-5.508973000	3.570076000	1.565099000
H	-4.985355000	4.300988000	0.039307000
C	-0.524255000	2.412983000	-3.213094000
H	-1.153194000	1.524197000	-3.349364000
C	0.923939000	1.937845000	-3.138699000
H	1.205117000	1.389081000	-4.044402000
H	1.063297000	1.267921000	-2.282242000
H	1.615559000	2.783439000	-3.033778000
C	-0.729343000	3.327803000	-4.420772000
H	-1.765641000	3.670335000	-4.510560000
H	-0.464840000	2.811631000	-5.349033000
H	-0.097636000	4.220408000	-4.349414000
C	-3.183388000	-2.475723000	0.040475000
C	-2.553730000	-3.647837000	-0.412085000
C	-2.570142000	-4.768641000	0.423763000
H	-2.086424000	-5.683744000	0.087986000
C	-3.179332000	-4.726142000	1.668246000
H	-3.190192000	-5.608934000	2.301492000
C	-3.746880000	-3.539761000	2.121068000
H	-4.178023000	-3.503735000	3.116949000
C	-3.746798000	-2.386781000	1.333438000
C	-1.810622000	-3.718692000	-1.731461000
H	-1.971304000	-2.775963000	-2.270190000
C	-0.306489000	-3.853155000	-1.483651000
H	-0.077188000	-4.802162000	-0.984382000
H	0.069763000	-3.035788000	-0.854433000
H	0.244839000	-3.826078000	-2.430063000
C	-2.310661000	-4.857619000	-2.618373000
H	-2.127645000	-5.833609000	-2.155414000
H	-1.791622000	-4.853231000	-3.581779000
H	-3.385671000	-4.789237000	-2.816385000
C	-4.271082000	-1.067795000	1.880610000
H	-3.553095000	-0.294407000	1.561539000
C	-4.312077000	-1.033369000	3.404471000
H	-5.111173000	-1.670571000	3.801211000
H	-4.512037000	-0.014561000	3.749239000
H	-3.365809000	-1.351129000	3.848647000
C	-5.647984000	-0.676399000	1.337874000
H	-5.649888000	-0.488124000	0.261685000
H	-5.994904000	0.242579000	1.824178000
H	-6.387631000	-1.457927000	1.549457000
H	-0.270648000	-0.680957000	-1.752831000
C	-0.268118000	0.086306000	1.829966000
C	-0.891189000	-0.604843000	2.847200000
O	0.148009000	1.302759000	2.367989000
C	-0.809426000	0.177906000	4.040082000
H	-1.320190000	-1.595365000	2.740491000
C	-0.170811000	1.332348000	3.699573000
H	-1.182338000	-0.072306000	5.025618000
C	0.235842000	2.544287000	4.440365000
H	-0.107516000	2.492431000	5.476024000
H	1.326769000	2.669245000	4.454186000
H	-0.177749000	3.456059000	3.990348000
P	2.900155000	-0.573889000	0.038039000
C	3.593357000	-1.854069000	-1.135554000
C	3.255988000	-1.476925000	-2.582635000
C	5.056953000	-2.284953000	-0.996966000
H	2.981295000	-2.740504000	-0.893145000
C	3.610764000	-2.599228000	-3.551145000
H	3.815242000	-0.571133000	-2.865997000
H	2.189583000	-1.218528000	-2.654282000
C	5.391866000	-3.406321000	-1.978139000
H	5.724581000	-1.432929000	-1.184598000
H	5.262260000	-2.615769000	0.028438000
C	5.072659000	-3.007180000	-3.413545000
H	3.387582000	-2.296005000	-4.580727000
H	2.973111000	-3.470514000	-3.340070000
H	6.445732000	-3.691601000	-1.879447000
H	4.804918000	-4.298967000	-1.715036000
H	5.313717000	-3.824380000	-4.103106000
H	5.712603000	-2.159819000	-3.702983000
C	3.732210000	-0.974087000	1.670194000
C	5.152341000	-0.442013000	1.889563000
C	2.841603000	-0.574288000	2.852634000
H	3.783893000	-2.077706000	1.658193000
C	5.732157000	-0.938170000	3.211550000

H	5.114561000	0.657211000	1.914640000
H	5.814116000	-0.708816000	1.058696000
C	3.423523000	-1.031474000	4.184507000
H	2.722636000	0.519560000	2.857377000
H	1.832572000	-0.973844000	2.717516000
C	4.849028000	-0.534133000	4.385357000
H	6.751994000	-0.556934000	3.342157000
H	5.815001000	-2.035408000	3.177519000
H	2.773501000	-0.698342000	5.002622000
H	3.420285000	-2.131657000	4.218359000
H	5.264505000	-0.907467000	5.328737000
H	4.841133000	0.563517000	4.463639000
C	3.521298000	1.080149000	-0.572872000
C	3.008614000	2.197768000	0.348728000
C	4.991632000	1.294463000	-0.950496000
H	2.954994000	1.175522000	-1.512447000
C	3.253048000	3.576041000	-0.255091000
H	3.518295000	2.131898000	1.323423000
H	1.939160000	2.051383000	0.555246000
C	5.184193000	2.674340000	-1.576508000
H	5.637714000	1.212314000	-0.069171000
H	5.323904000	0.520904000	-1.652604000
C	4.713763000	3.777950000	-0.637075000
H	2.916200000	4.355796000	0.438532000
H	2.628455000	3.680041000	-1.155323000
H	6.235152000	2.820151000	-1.852792000
H	4.607437000	2.727057000	-2.513290000
H	4.862583000	4.763410000	-1.093968000
H	5.333149000	3.762690000	0.272546000

TS-5_M06L.log

SCF (M06L) = -2879.94954584
E(SCF)+ZPE(0 K)= -2878.512585
H(298 K)= -2878.432133
G(298 K)= -2878.625997
Lowest Frequency = -328.5649 cm-1

Pd	-0.443879000	-0.104638000	0.764859000
Al	1.908473000	-0.266566000	0.121185000
N	2.567807000	-1.622676000	-1.081023000
N	3.193241000	1.067568000	-0.453903000
C	2.638020000	-1.192534000	-2.344724000
C	2.759912000	0.177027000	-2.654153000
H	2.692510000	0.435785000	-3.706521000
C	3.156019000	1.211726000	-1.793599000
C	2.555978000	-2.154230000	-3.491132000
H	2.942458000	-3.141468000	-3.228362000
H	3.090150000	-1.778322000	-4.365664000
H	1.504371000	-2.283166000	-3.775552000
C	3.486924000	2.527071000	-2.439529000
H	4.180657000	2.405250000	-3.275250000
H	3.896836000	3.252916000	-1.736778000
H	2.559847000	2.944416000	-2.851675000
C	2.629497000	-3.020973000	-0.765904000
C	3.874259000	-3.558341000	-0.371436000
C	3.933172000	-4.909952000	-0.028910000
H	4.881704000	-5.343839000	0.273557000
C	2.797839000	-5.711515000	-0.061956000
H	2.863962000	-6.760871000	0.212076000
C	1.583155000	-5.163318000	-0.440297000
H	0.691506000	-5.786595000	-0.463348000
C	1.469267000	-3.815127000	-0.795962000
C	5.127926000	-2.703647000	-0.365689000
H	4.838989000	-1.698867000	-0.017250000
C	6.211990000	-3.241073000	0.562760000
H	5.828474000	-3.460939000	1.564091000
H	7.026693000	-2.517358000	0.660437000
H	6.655173000	-4.163360000	0.171010000
C	5.698624000	-2.551408000	-1.778788000
H	5.902271000	-3.534809000	-2.218310000
H	6.641576000	-1.994443000	-1.759483000
H	5.018304000	-2.018784000	-2.448499000
C	0.110445000	-3.292123000	-1.215164000
H	0.199183000	-2.223482000	-1.459459000
C	-0.900922000	-3.416467000	-0.074784000
H	-1.873441000	-3.002428000	-0.362814000
H	-0.568603000	-2.874861000	0.818564000
H	-1.060752000	-4.467643000	0.196854000
C	-0.387234000	-4.033602000	-2.458712000
H	0.373775000	-4.086400000	-3.243902000
H	-1.270328000	-3.543419000	-2.878171000
H	-0.669455000	-5.064695000	-2.214383000
C	3.969530000	1.961452000	0.346377000
C	3.331042000	2.711014000	1.363020000
C	4.107170000	3.540530000	2.172088000
H	3.622047000	4.116190000	2.954698000
C	5.480035000	3.652827000	1.987963000
H	6.065806000	4.312832000	2.621813000
C	6.095812000	2.909312000	0.994054000
H	7.173416000	2.979857000	0.858699000
C	5.369175000	2.044005000	0.168803000
C	1.833749000	2.638084000	1.538489000
H	1.535392000	1.577957000	1.513560000
C	1.328443000	3.148061000	2.879536000
H	1.412512000	4.239095000	2.962005000
H	1.870226000	2.695140000	3.715660000
H	0.271540000	2.887176000	2.989791000
C	1.091407000	3.316818000	0.389689000
H	1.211535000	4.407095000	0.433533000
H	0.023148000	3.080048000	0.439418000
H	1.438168000	2.979111000	-0.590413000
C	6.150760000	1.200550000	-0.824129000
H	5.456089000	0.545995000	-1.365278000
C	7.148876000	0.305019000	-0.088759000
H	7.878639000	0.902759000	0.468124000
H	7.709143000	-0.320718000	-0.792029000
H	6.648530000	-0.353028000	0.627796000
C	6.894860000	2.060346000	-1.846608000
H	6.231713000	2.754135000	-2.369604000
H	7.388258000	1.432425000	-2.595811000
H	7.672946000	2.659485000	-1.360290000
Al	-2.765995000	0.523897000	0.411777000
N	-4.555065000	-0.329232000	0.512809000
N	-3.570145000	2.038755000	-0.617490000
C	-5.747290000	0.237717000	0.287062000
C	-5.889605000	1.510211000	-0.277995000
H	-6.904813000	1.862319000	-0.423428000
C	-4.867502000	2.320183000	-0.790253000
C	-7.004682000	-0.528146000	0.582542000
H	-7.868486000	0.137714000	0.621153000
H	-6.926639000	-1.076217000	1.525719000
H	-7.194923000	-1.279135000	-0.192697000
C	-5.280311000	3.532824000	-1.577249000
H	-5.063466000	4.457917000	-1.032645000
H	-6.348820000	3.512155000	-1.796593000
H	-4.725367000	3.597721000	-2.518093000
C	-4.521434000	-1.732798000	0.809291000
C	-4.747203000	-2.650305000	-0.240155000
C	-4.665218000	-4.015059000	0.039976000
H	-4.824852000	-4.731979000	-0.762809000
C	-4.366294000	-4.470546000	1.318110000
H	-4.306490000	-5.536931000	1.517969000
C	-4.136457000	-3.555442000	2.335187000
H	-3.900890000	-3.910201000	3.336239000
C	-4.203692000	-2.177834000	2.106707000
C	-4.990839000	-2.199197000	-1.669937000
H	-5.245088000	-1.132448000	-1.669226000
C	-3.706170000	-2.359424000	-2.481279000

H	-3.409443000	-3.414362000	-2.514578000
H	-2.874099000	-1.800024000	-2.033436000
H	-3.836084000	-2.012982000	-3.513125000
C	-6.141248000	-2.945823000	-2.340641000
H	-6.345566000	-2.527554000	-3.331125000
H	-7.064841000	-2.890552000	-1.754949000
H	-5.909130000	-4.006873000	-2.481626000
C	-3.987027000	-1.226706000	3.266000000
H	-3.924417000	-0.208415000	2.861718000
C	-5.173814000	-1.270114000	4.229068000
H	-6.115079000	-1.012294000	3.731619000
H	-5.030271000	-0.568816000	5.057547000
H	-5.293243000	-2.271300000	4.659432000
C	-2.676138000	-1.505503000	3.994268000
H	-2.513787000	-0.760831000	4.781282000
H	-1.828109000	-1.459454000	3.301347000
H	-2.680352000	-2.490539000	4.474633000
C	-2.592846000	2.901935000	-1.215692000
C	-2.317703000	4.159825000	-0.644160000
C	-1.342207000	4.959329000	-1.244800000
H	-1.112660000	5.931110000	-0.813310000
C	-0.637209000	4.520385000	-2.357240000
H	0.127477000	5.152727000	-2.801831000
C	-0.897732000	3.262766000	-2.888888000
H	-0.333490000	2.916044000	-3.751343000
C	-1.875078000	2.432405000	-2.337384000
C	-2.983885000	4.617891000	0.640652000
H	-3.901359000	4.035209000	0.781452000
C	-2.080733000	4.315495000	1.836466000
H	-1.136652000	4.868152000	1.761463000
H	-1.830717000	3.248236000	1.887850000
H	-2.564287000	4.599144000	2.777496000
C	-3.375233000	6.092567000	0.617000000
H	-3.949881000	6.351392000	1.511504000
H	-3.982764000	6.343095000	-0.259466000
H	-2.496765000	6.746355000	0.602303000
C	-2.196768000	1.088012000	-2.961775000
H	-2.453661000	0.404467000	-2.139286000
C	-1.022541000	0.449350000	-3.690940000
H	-0.755782000	0.993285000	-4.605159000
H	-1.284680000	-0.571764000	-3.992298000
H	-0.139135000	0.395701000	-3.044066000
C	-3.422251000	1.185358000	-3.871493000
H	-3.257861000	1.914750000	-4.673551000
H	-4.317966000	1.488880000	-3.319903000
H	-3.636846000	0.218659000	-4.340150000
H	0.402020000	0.255764000	-0.777578000
C	0.694612000	-0.604502000	2.423296000
C	1.020292000	-0.341827000	3.715022000
O	2.373446000	-0.767337000	1.872472000
C	2.412601000	-0.283512000	4.047006000
H	0.219822000	-0.177829000	4.429980000
C	3.171121000	-0.511521000	2.946128000
H	2.818132000	-0.100703000	5.034436000
C	4.638521000	-0.576511000	2.778339000
H	5.138155000	-0.389810000	3.731167000
H	4.957106000	-1.559708000	2.410397000
H	4.996294000	0.171494000	2.059480000

TS-5’-1_M06L.log

SCF (M06L) = -2685.71015777
E(SCF)+ZPE(0 K)= -2684.493508
H(298 K)= -2684.42844
G(298 K)= -2684.59189
Lowest Frequency = -330.7362 cm-1

Pd	0.905553000	0.138392000	-0.162292000
Al	-1.460366000	0.001848000	0.225644000
N	-2.709391000	-1.265813000	0.941420000
N	-2.329482000	1.529744000	1.039072000
C	-2.939108000	-1.125744000	2.262595000
C	-2.869714000	0.113323000	2.905125000
H	-3.077116000	0.116145000	3.969925000
C	-2.737030000	1.382639000	2.299058000
C	-3.247832000	-2.340275000	3.087758000
H	-4.008037000	-2.974662000	2.626320000
H	-3.572835000	-2.071981000	4.094063000
H	-2.340942000	-2.951618000	3.169149000
C	-3.080328000	2.571998000	3.145360000
H	-3.971190000	2.372847000	3.745554000
H	-3.243565000	3.470345000	2.546928000
H	-2.261985000	2.779845000	3.843484000
C	-3.167552000	-2.449401000	0.279077000
C	-4.534315000	-2.536324000	-0.078372000
C	-4.976487000	-3.701787000	-0.708165000
H	-6.023356000	-3.791862000	-0.985878000
C	-4.100356000	-4.738485000	-1.010651000
H	-4.466092000	-5.632681000	-1.507937000
C	-2.756464000	-4.616498000	-0.690649000
H	-2.064913000	-5.417986000	-0.943253000
C	-2.265556000	-3.482110000	-0.037055000
C	-5.500874000	-1.385719000	0.162410000
H	-4.910650000	-0.459877000	0.091879000
C	-6.616234000	-1.325371000	-0.881929000
H	-6.246933000	-1.439562000	-1.905113000
H	-7.143898000	-0.367955000	-0.815204000
H	-7.364529000	-2.108117000	-0.714175000
C	-6.148405000	-1.401069000	1.551201000
H	-6.621872000	-2.370075000	1.748729000
H	-6.928964000	-0.634661000	1.609892000
H	-5.439421000	-1.196970000	2.355541000
C	-0.787200000	-3.396537000	0.284886000
H	-0.611289000	-2.498497000	0.891216000
C	0.040042000	-3.251259000	-0.993396000
H	1.098554000	-3.104237000	-0.747655000
H	-0.274982000	-2.387402000	-1.588580000
H	-0.042578000	-4.149402000	-1.618806000
C	-0.303468000	-4.593117000	1.101686000
H	-0.881519000	-4.722858000	2.022308000
H	0.748402000	-4.463886000	1.378460000
H	-0.378252000	-5.527288000	0.533337000
C	-2.214627000	2.813522000	0.408596000
C	-1.142164000	3.675012000	0.710280000
C	-1.020598000	4.853201000	-0.033909000
H	-0.194861000	5.527217000	0.185531000
C	-1.916719000	5.171378000	-1.042165000
H	-1.794715000	6.088088000	-1.612406000
C	-2.975676000	4.313952000	-1.318969000
H	-3.678523000	4.569061000	-2.106563000
C	-3.145284000	3.128310000	-0.605460000
C	-0.123539000	3.402925000	1.801429000
H	-0.361866000	2.440467000	2.272836000
C	1.290458000	3.283216000	1.235407000
H	1.601446000	4.208238000	0.734239000
H	1.352495000	2.459963000	0.510688000
H	2.008276000	3.082242000	2.038972000
C	-0.160730000	4.498434000	2.869974000
H	0.185238000	5.453814000	2.459523000
H	0.499959000	4.246858000	3.706023000
H	-1.165856000	4.665140000	3.268699000
C	-4.322255000	2.211352000	-0.870455000
H	-3.941757000	1.181481000	-0.814256000
C	-4.948585000	2.396326000	-2.245919000
H	-5.446020000	3.368228000	-2.338307000
H	-5.712802000	1.630949000	-2.418650000
H	-4.201776000	2.316768000	-3.042447000
C	-5.390171000	2.353961000	0.217084000
H	-5.009095000	2.089918000	1.208283000
H	-6.244381000	1.698781000	0.009418000

H	-5.763060000	3.383492000	0.262125000
H	0.034425000	-0.278185000	1.360031000
C	-0.088498000	0.590837000	-1.875892000
C	-0.359363000	1.012346000	-3.134296000
O	-1.722624000	-0.155537000	-1.633025000
C	-1.639080000	0.687472000	-3.694642000
H	0.420017000	1.537168000	-3.679713000
C	-2.392851000	-0.001793000	-2.800902000
H	-1.951365000	0.906252000	-4.708544000
C	-3.695875000	-0.683387000	-2.955990000
H	-4.135737000	-0.460800000	-3.931118000
H	-3.584035000	-1.771637000	-2.862375000
H	-4.407499000	-0.376570000	-2.182507000
P	3.243902000	-0.102995000	0.087304000
C	4.270389000	1.341572000	-0.522780000
C	5.727921000	1.085819000	-0.920663000
C	3.560335000	2.093584000	-1.657140000
H	4.275700000	2.016114000	0.353254000
C	6.449659000	2.395056000	-1.229671000
H	5.741833000	0.452132000	-1.820382000
H	6.264548000	0.529671000	-0.145459000
C	4.273583000	3.400277000	-1.984379000
H	3.534057000	1.449205000	-2.550279000
H	2.510950000	2.271383000	-1.395105000
C	5.740041000	3.172025000	-2.331529000
H	7.492251000	2.197556000	-1.505791000
H	6.483540000	3.006742000	-0.315075000
H	3.759899000	3.918568000	-2.802526000
H	4.208431000	4.066327000	-1.110081000
H	6.247912000	4.124873000	-2.520861000
H	5.801614000	2.598191000	-3.268189000
C	3.785298000	-0.303507000	1.868374000
C	3.276676000	-1.641648000	2.416724000
C	5.244228000	-0.053683000	2.261143000
H	3.187147000	0.479543000	2.365489000
C	3.478850000	-1.740607000	3.924131000
H	3.815779000	-2.469542000	1.930509000
H	2.215585000	-1.759997000	2.154436000
C	5.426900000	-0.162896000	3.773757000
H	5.905829000	-0.778044000	1.766555000
H	5.565705000	0.938486000	1.920343000
C	4.936870000	-1.505771000	4.301500000
H	3.136122000	-2.715558000	4.290224000
H	2.850555000	-0.985702000	4.419416000
H	6.477924000	-0.001183000	4.040584000
H	4.857502000	0.642765000	4.260949000
H	5.070729000	-1.564070000	5.387881000
H	5.554490000	-2.308766000	3.871071000
C	3.801325000	-1.660052000	-0.786217000
C	3.481279000	-1.569840000	-2.284357000
C	5.208093000	-2.226459000	-0.565044000
H	3.098434000	-2.398833000	-0.361937000
C	3.655128000	-2.918908000	-2.973312000
H	4.155917000	-0.835096000	-2.752524000
H	2.461402000	-1.186162000	-2.422035000
C	5.352738000	-3.585253000	-1.247599000
H	5.962709000	-1.541707000	-0.970984000
H	5.423334000	-2.325391000	0.504585000
C	5.046325000	-3.494110000	-2.737195000
H	3.451200000	-2.824901000	-4.046194000
H	2.903135000	-3.618175000	-2.577471000
H	6.359912000	-3.985734000	-1.082432000
H	4.656333000	-4.296912000	-0.778121000
H	5.145018000	-4.477118000	-3.212198000
H	5.792666000	-2.843131000	-3.217200000

TS-5’-2_M06L.log

SCF (M06L) = -2685.74365806

E(SCF)+ZPE(0 K)= -2684.524158
H(298 K)= -2684.459877
G(298 K)= -2684.619276
Lowest Frequency = -39.1130 cm-1

Pd	-0.844183000	0.240431000	-0.688531000
Al	1.654997000	0.016370000	-0.509777000
N	2.211120000	1.328898000	0.797828000
N	1.856572000	-1.506835000	0.665737000
C	1.871452000	1.161436000	2.077574000
C	1.514280000	-0.091717000	2.601345000
H	1.247940000	-0.108441000	3.652800000
C	1.626140000	-1.345487000	1.979271000
C	1.839829000	2.338958000	3.004664000
H	2.656721000	3.039423000	2.814881000
H	1.865105000	2.030879000	4.051122000
H	0.905186000	2.890424000	2.836845000
C	1.522465000	-2.553034000	2.863144000
H	1.442594000	-2.267228000	3.912549000
H	2.394684000	-3.203113000	2.738415000
H	0.653244000	-3.161843000	2.600869000
C	2.874951000	2.527947000	0.373176000
C	4.285628000	2.530049000	0.371915000
C	4.945729000	3.676222000	-0.072722000
H	6.032901000	3.697862000	-0.071982000
C	4.238165000	4.786989000	-0.518821000
H	4.769832000	5.668951000	-0.865759000
C	2.851276000	4.761589000	-0.521935000
H	2.294802000	5.627124000	-0.876914000
C	2.143784000	3.639789000	-0.078422000
C	5.065463000	1.337864000	0.888029000
H	4.414222000	0.461162000	0.794138000
C	6.330383000	1.057031000	0.086779000
H	6.108894000	0.922454000	-0.976309000
H	6.812819000	0.143005000	0.450721000
H	7.069219000	1.860617000	0.180801000
C	5.397404000	1.504299000	2.372167000
H	5.994560000	2.408134000	2.541451000
H	5.973077000	0.648596000	2.742164000
H	4.494378000	1.582617000	2.985962000
C	0.631018000	3.651357000	-0.117997000
H	0.265827000	2.731423000	0.356369000
C	0.129797000	3.640038000	-1.561669000
H	-0.965968000	3.611328000	-1.589508000
H	0.495191000	2.764337000	-2.109932000
H	0.456381000	4.538585000	-2.099494000
C	0.046337000	4.837021000	0.646706000
H	0.424851000	4.890849000	1.673009000
H	-1.046199000	4.760721000	0.694752000
H	0.283494000	5.790872000	0.161359000
C	2.070175000	-2.839207000	0.161770000
C	0.986990000	-3.713378000	-0.068731000
C	1.265815000	-5.011706000	-0.507696000
H	0.436023000	-5.692325000	-0.688479000
C	2.564352000	-5.439744000	-0.730426000
H	2.757342000	-6.454599000	-1.067270000
C	3.617406000	-4.553656000	-0.542515000
H	4.632675000	-4.882729000	-0.743451000
C	3.397378000	-3.246090000	-0.105465000
C	-0.469687000	-3.307592000	0.062164000
H	-0.512046000	-2.270177000	0.428412000
C	-1.142371000	-3.338733000	-1.312422000
H	-1.196271000	-4.365365000	-1.694963000
H	-0.608188000	-2.719304000	-2.036907000
H	-2.167045000	-2.954553000	-1.243300000
C	-1.263188000	-4.203792000	1.015185000
H	-1.237811000	-5.249655000	0.689285000
H	-2.317259000	-3.900498000	1.030347000
H	-0.899756000	-4.176738000	2.047788000
C	4.564457000	-2.297155000	0.092655000

H	4.229430000	-1.305493000	-0.234889000
C	5.777038000	-2.657606000	-0.758517000
H	6.275758000	-3.567300000	-0.404673000
H	6.517485000	-1.852644000	-0.717625000
H	5.504343000	-2.812013000	-1.807312000
C	4.969532000	-2.190802000	1.564286000
H	4.181383000	-1.748106000	2.181814000
H	5.858102000	-1.557768000	1.672727000
H	5.214369000	-3.177109000	1.976590000
H	-0.751534000	0.837851000	0.833108000
C	0.127084000	-0.224710000	-2.276535000
C	0.783885000	-0.355607000	-3.440803000
O	3.020269000	0.152086000	-1.628957000
C	2.157675000	-0.140908000	-3.820102000
H	0.128119000	-0.688810000	-4.255616000
C	3.173269000	0.099696000	-2.948466000
H	2.402466000	-0.192862000	-4.876382000
C	4.578642000	0.331233000	-3.393734000
H	4.683305000	0.252438000	-4.477601000
H	4.913756000	1.328482000	-3.082156000
H	5.257999000	-0.388518000	-2.921061000
P	-3.031749000	0.063910000	0.040085000
C	-4.177088000	-0.900248000	-1.083331000
C	-5.680487000	-0.619574000	-0.979881000
C	-3.736131000	-0.829871000	-2.550720000
H	-4.010403000	-1.940983000	-0.751689000
C	-6.475221000	-1.612357000	-1.824723000
H	-5.877989000	0.398260000	-1.347461000
H	-6.024468000	-0.644716000	0.059455000
C	-4.532050000	-1.809112000	-3.405487000
H	-3.886630000	0.195426000	-2.922474000
H	-2.658967000	-1.019389000	-2.627444000
C	-6.033440000	-1.578747000	-3.282268000
H	-7.548039000	-1.404290000	-1.739123000
H	-6.324106000	-2.624544000	-1.419541000
H	-4.215389000	-1.738381000	-4.452065000
H	-4.295104000	-2.834428000	-3.082088000
H	-6.589572000	-2.317857000	-3.869905000
H	-6.282784000	-0.595487000	-3.707957000
C	-3.106103000	-0.844910000	1.671346000
C	-2.571362000	-0.007873000	2.839547000
C	-4.431538000	-1.513312000	2.056046000
H	-2.373435000	-1.653798000	1.506982000
C	-2.399014000	-0.868821000	4.085339000
H	-3.274900000	0.809089000	3.060634000
H	-1.619903000	0.458122000	2.559123000
C	-4.265517000	-2.357845000	3.318332000
H	-5.199930000	-0.745624000	2.230016000
H	-4.804368000	-2.145536000	1.241225000
C	-3.707212000	-1.543157000	4.477923000
H	-2.009075000	-0.263126000	4.911766000
H	-1.638221000	-1.638739000	3.880177000
H	-5.223485000	-2.815525000	3.590282000
H	-3.577789000	-3.188625000	3.100261000
H	-3.570324000	-2.177677000	5.360834000
H	-4.437585000	-0.771069000	4.762604000
C	-3.710852000	1.772275000	0.310186000
C	-3.734750000	2.536145000	-1.020622000
C	-5.027305000	1.972158000	1.067842000
H	-2.909181000	2.221364000	0.921953000
C	-4.008685000	4.018679000	-0.799184000
H	-4.521214000	2.113622000	-1.665398000
H	-2.786208000	2.385706000	-1.554155000
C	-5.273001000	3.461251000	1.304511000
H	-5.864484000	1.561546000	0.490700000
H	-5.016655000	1.434200000	2.022931000
C	-5.293822000	4.235777000	-0.008811000
H	-4.049783000	4.543877000	-1.760223000
H	-3.163478000	4.456600000	-0.247053000
H	-6.210797000	3.606348000	1.852786000
H	-4.473357000	3.858059000	1.947978000
H	-5.456110000	5.303304000	0.177245000
H	-6.148826000	3.894595000	-0.611495000

TS-6_M06L.log

SCF (M06L) = -2879.93018381
E(SCF)+ZPE(0 K)= -2878.557597
H(298 K)= -2878.477219
G(298 K)= -2878.671349
Lowest Frequency = -340.8341 cm-1

Pd	0.684661000	0.430550000	-0.554844000
Al	-2.583305000	0.377584000	-0.559975000
N	-3.077963000	-1.314697000	0.189043000
N	-2.712643000	1.348340000	1.088853000
C	-2.853464000	-1.629997000	1.465865000
C	-2.511097000	-0.662158000	2.422680000
H	-2.316671000	-1.027965000	3.424768000
C	-2.534001000	0.731579000	2.270601000
C	-2.958984000	-3.054614000	1.916133000
H	-3.761248000	-3.589546000	1.401742000
H	-3.109266000	-3.125586000	2.995065000
H	-2.021910000	-3.570404000	1.674110000
C	-2.383913000	1.558328000	3.512535000
H	-2.362509000	0.930512000	4.404223000
H	-3.199618000	2.282567000	3.604240000
H	-1.458095000	2.141526000	3.479500000
C	-3.760709000	-2.218939000	-0.692851000
C	-5.165670000	-2.105493000	-0.773344000
C	-5.845815000	-2.923841000	-1.675591000
H	-6.929691000	-2.857485000	-1.739371000
C	-5.162502000	-3.818739000	-2.490261000
H	-5.707747000	-4.444846000	-3.191353000
C	-3.781433000	-3.908810000	-2.402521000
H	-3.245507000	-4.610985000	-3.038111000
C	-3.052063000	-3.116870000	-1.509107000
C	-5.940168000	-1.165189000	0.129710000
H	-5.230027000	-0.447834000	0.557758000
C	-6.992826000	-0.358575000	-0.621052000
H	-6.528134000	0.263896000	-1.391800000
H	-7.527505000	0.299833000	0.072913000
H	-7.745395000	-0.996969000	-1.096547000
C	-6.566770000	-1.932433000	1.295141000
H	-7.276252000	-2.685970000	0.933526000
H	-7.110565000	-1.254491000	1.962280000
H	-5.811077000	-2.451876000	1.893804000
C	-1.548920000	-3.292465000	-1.435919000
H	-1.140513000	-2.564550000	-0.719746000
C	-0.887446000	-3.027718000	-2.787404000
H	0.200826000	-3.120525000	-2.706060000
H	-1.109359000	-2.019331000	-3.147176000
H	-1.226462000	-3.747958000	-3.541639000
C	-1.206858000	-4.702086000	-0.949174000
H	-1.682402000	-4.939195000	0.008567000
H	-0.126142000	-4.816970000	-0.821887000
H	-1.538489000	-5.456667000	-1.672241000
C	-2.841917000	2.783498000	1.079843000
C	-1.697688000	3.602875000	1.130630000
C	-1.871922000	4.988974000	1.184417000
H	-0.990985000	5.626676000	1.227842000
C	-3.136446000	5.555550000	1.171446000
H	-3.254483000	6.634570000	1.221554000
C	-4.253832000	4.736096000	1.064779000
H	-5.240338000	5.187546000	1.018127000
C	-4.135490000	3.345814000	1.005328000
C	-0.285936000	3.059718000	1.063329000
H	-0.335276000	1.957622000	1.101669000
C	0.359523000	3.465698000	-0.267169000

H	0.506175000	4.552583000	-0.306688000
H	-0.258161000	3.172000000	-1.121246000
H	1.346090000	2.994007000	-0.387782000
C	0.590222000	3.513065000	2.230067000
H	0.856158000	4.573450000	2.142409000
H	1.522472000	2.936279000	2.242058000
H	0.103777000	3.377505000	3.201634000
C	-5.368066000	2.471815000	0.859367000
H	-5.098773000	1.652782000	0.180588000
C	-6.538702000	3.206778000	0.216013000
H	-6.964499000	3.967955000	0.879933000
H	-7.342474000	2.503027000	-0.018087000
H	-6.242675000	3.699422000	-0.715062000
C	-5.803524000	1.853867000	2.189504000
H	-5.055488000	1.165286000	2.595482000
H	-6.730927000	1.283396000	2.059630000
H	-5.996338000	2.629941000	2.940096000
Al	2.891608000	0.127812000	0.473472000
N	4.647886000	0.578406000	-0.299948000
N	3.692817000	-1.344059000	1.479984000
C	5.849957000	0.133658000	0.077593000
C	6.009017000	-0.827316000	1.088324000
H	7.027660000	-1.086557000	1.354414000
C	4.994704000	-1.567147000	1.706696000
C	7.086473000	0.658542000	-0.592666000
H	7.300219000	1.683638000	-0.269600000
H	6.958993000	0.701004000	-1.678205000
H	7.956275000	0.043222000	-0.358341000
C	5.397175000	-2.683957000	2.622868000
H	4.792775000	-2.681931000	3.534971000
H	6.452967000	-2.618282000	2.889527000
H	5.227797000	-3.656159000	2.146705000
C	4.517290000	1.487378000	-1.403413000
C	4.005822000	0.981359000	-2.619146000
C	3.765523000	1.883906000	-3.656360000
H	3.367322000	1.517027000	-4.597872000
C	4.009946000	3.243177000	-3.499906000
H	3.807469000	3.929364000	-4.317432000
C	4.502692000	3.722424000	-2.294353000
H	4.676612000	4.789023000	-2.169904000
C	4.762727000	2.861231000	-1.225190000
C	3.758419000	-0.504019000	-2.807262000
H	3.399178000	-0.902163000	-1.846892000
C	2.673000000	-0.817730000	-3.826993000
H	2.975774000	-0.557561000	-4.847627000
H	1.744302000	-0.288213000	-3.589188000
H	2.454055000	-1.890658000	-3.820931000
C	5.055883000	-1.242669000	-3.138405000
H	4.865136000	-2.310541000	-3.292600000
H	5.794429000	-1.154200000	-2.334942000
H	5.508927000	-0.849649000	-4.056054000
C	5.212130000	3.434453000	0.104915000
H	5.574842000	2.610845000	0.731165000
C	6.349776000	4.441200000	-0.040872000
H	7.198355000	4.022974000	-0.592627000
H	6.709613000	4.762023000	0.941602000
H	6.027450000	5.341715000	-0.574039000
C	4.020459000	4.052775000	0.833442000
H	4.308475000	4.440890000	1.816551000
H	3.226795000	3.311577000	0.976972000
H	3.590925000	4.878981000	0.254634000
C	2.719616000	-2.301244000	1.921454000
C	1.850596000	-1.979456000	2.981706000
C	0.829028000	-2.879765000	3.297789000
H	0.150173000	-2.641584000	4.115524000
C	0.670225000	-4.066146000	2.594331000
H	-0.126213000	-4.757730000	2.858822000
C	1.537764000	-4.368580000	1.551420000
H	1.405025000	-5.293162000	0.993046000
C	2.566371000	-3.498059000	1.189439000
C	2.004975000	-0.715390000	3.804335000
H	2.816318000	-0.120990000	3.364357000
C	0.741121000	0.140170000	3.777621000
H	-0.102522000	-0.380273000	4.246213000
H	0.449834000	0.383736000	2.747425000
H	0.896323000	1.075995000	4.326409000
C	2.403689000	-1.046507000	5.242651000
H	2.549711000	-0.133359000	5.828533000
H	3.330917000	-1.626702000	5.286295000
H	1.624982000	-1.636809000	5.739064000
C	3.421260000	-3.822858000	-0.022384000
H	4.258503000	-3.115155000	-0.064654000
C	2.603496000	-3.633483000	-1.300185000
H	1.815979000	-4.392969000	-1.369412000
H	3.232811000	-3.730955000	-2.192484000
H	2.111758000	-2.652578000	-1.325275000
C	4.008495000	-5.231290000	0.031937000
H	3.224142000	-5.994989000	-0.006583000
H	4.586674000	-5.405459000	0.945559000
H	4.669899000	-5.405678000	-0.822348000
H	0.559120000	-0.856904000	-1.435864000
C	-1.001798000	0.456785000	-1.653281000
C	-1.205019000	0.649550000	-2.988906000
O	-3.881804000	0.857736000	-1.684748000
C	-2.473343000	0.948615000	-3.618344000
H	-0.368654000	0.572315000	-3.697105000
C	-3.689304000	1.019969000	-2.998306000
H	-2.472417000	1.103559000	-4.694412000
C	-4.957595000	1.253541000	-3.749640000
H	-4.778981000	1.450467000	-4.808666000
H	-5.611813000	0.376031000	-3.665676000
H	-5.511947000	2.097290000	-3.321372000

TS-6'_M06L.log

SCF (M06L) = -2685.75879113
E(SCF)+ZPE(0 K)= -2684.540027
H(298 K)= -2684.475306
G(298 K)= -2684.636732
Lowest Frequency = - 216.7738 cm-1

Pd	1.073172000	0.056988000	-1.236163000
Al	-2.004619000	-0.040694000	-0.729263000
N	-1.818230000	-1.376040000	0.643745000
N	-2.027714000	1.444257000	0.473819000
C	-1.143123000	-1.100474000	1.761216000
C	-0.721499000	0.203716000	2.082458000
H	-0.099564000	0.300693000	2.966292000
C	-1.206154000	1.405091000	1.534354000
C	-0.852457000	-2.203396000	2.731307000
H	-1.763249000	-2.769525000	2.949322000
H	-0.442824000	-1.819570000	3.665448000
H	-0.142287000	-2.922968000	2.308059000
C	-0.831007000	2.681923000	2.224231000
H	-0.147586000	2.497361000	3.054752000
H	-1.718966000	3.199692000	2.602731000
H	-0.352905000	3.372392000	1.520992000
C	-2.289507000	-2.711479000	0.400263000
C	-3.612464000	-3.040194000	0.756500000
C	-4.082903000	-4.321279000	0.461719000
H	-5.104172000	-4.582261000	0.732762000
C	-3.273999000	-5.259667000	-0.164131000
H	-3.657263000	-6.251298000	-0.388117000
C	-1.970790000	-4.922201000	-0.500162000
H	-1.334110000	-5.655731000	-0.990713000
C	-1.450663000	-3.653094000	-0.229702000
C	-4.529731000	-2.065798000	1.467477000
H	-3.973277000	-1.134570000	1.631832000
C	-5.754995000	-1.736766000	0.616598000
H	-5.465223000	-1.235926000	-0.311523000

H	-6.439702000	-1.080363000	1.163693000
H	-6.311841000	-2.645147000	0.359245000
C	-4.950218000	-2.599761000	2.836868000
H	-5.533468000	-3.522701000	2.742876000
H	-5.576678000	-1.870508000	3.361548000
H	-4.088298000	-2.821426000	3.474963000
C	-0.017871000	-3.362002000	-0.634668000
H	0.230256000	-2.328917000	-0.336463000
C	0.152942000	-3.451456000	-2.150406000
H	1.172049000	-3.168330000	-2.437981000
H	-0.535307000	-2.781186000	-2.670911000
H	-0.024816000	-4.475325000	-2.500492000
C	0.966423000	-4.306480000	0.056652000
H	0.887497000	-4.276611000	1.149158000
H	1.997697000	-4.049290000	-0.210106000
H	0.797606000	-5.344220000	-0.252166000
C	-2.696751000	2.681321000	0.161031000
C	-2.063117000	3.669452000	-0.613946000
C	-2.737249000	4.875505000	-0.829472000
H	-2.253583000	5.646638000	-1.425718000
C	-4.005447000	5.093788000	-0.315619000
H	-4.511533000	6.039821000	-0.487892000
C	-4.643599000	4.081460000	0.391695000
H	-5.655306000	4.242130000	0.751764000
C	-4.016232000	2.857830000	0.632938000
C	-0.732548000	3.442545000	-1.296012000
H	-0.304622000	2.493573000	-0.942109000
C	-0.950087000	3.311419000	-2.804217000
H	-1.286123000	4.265804000	-3.228321000
H	-1.699255000	2.550821000	-3.039997000
H	-0.019781000	3.019103000	-3.302805000
C	0.284441000	4.544284000	-1.016810000
H	-0.034574000	5.507476000	-1.431707000
H	1.242443000	4.289355000	-1.483900000
H	0.464416000	4.693791000	0.054009000
C	-4.743239000	1.740553000	1.359298000
H	-4.454155000	0.804008000	0.861250000
C	-6.259511000	1.855922000	1.257554000
H	-6.642175000	2.725739000	1.802941000
H	-6.734559000	0.975552000	1.700089000
H	-6.593425000	1.933225000	0.218431000
C	-4.333870000	1.628909000	2.829421000
H	-3.275206000	1.384967000	2.954022000
H	-4.908707000	0.839838000	3.327667000
H	-4.531789000	2.567293000	3.361059000
H	1.111133000	1.024279000	-2.477383000
C	-0.690095000	-0.041334000	-2.154058000
C	-1.190916000	-0.207504000	-3.411227000
O	-3.549938000	-0.287316000	-1.560974000
C	-2.573181000	-0.485912000	-3.741631000
H	-0.533239000	-0.134007000	-4.284889000
C	-3.634539000	-0.510153000	-2.881139000
H	-2.804532000	-0.672744000	-4.787332000
C	-5.028666000	-0.787255000	-3.334599000
H	-5.091278000	-0.909502000	-4.417633000
H	-5.411293000	-1.699158000	-2.859450000
H	-5.699289000	0.024980000	-3.030761000
P	3.150795000	0.244096000	-0.114331000
C	4.460841000	-0.712916000	-1.047003000
C	5.738882000	-1.132594000	-0.314517000
C	3.830587000	-1.928718000	-1.740358000
H	4.746032000	-0.004425000	-1.844657000
C	6.722956000	-1.807342000	-1.267056000
H	5.478170000	-1.843089000	0.484448000
H	6.214265000	-0.277452000	0.177149000
C	4.812663000	-2.625899000	-2.673300000
H	3.487671000	-2.640219000	-0.971610000
H	2.929636000	-1.610919000	-2.281924000
C	6.095280000	-3.016160000	-1.949466000
H	7.631693000	-2.095600000	-0.726144000
H	7.036411000	-1.079741000	-2.030840000
H	4.339061000	-3.504313000	-3.127327000
H	5.058622000	-1.946462000	-3.502582000
H	6.806036000	-3.480360000	-2.642395000
H	5.864271000	-3.776765000	-1.188628000
C	3.761690000	2.008340000	-0.140835000
C	2.935735000	2.852705000	0.837563000
C	5.268097000	2.254182000	0.004986000
H	3.478956000	2.335893000	-1.154955000
C	3.314339000	4.328545000	0.784751000
H	3.086060000	2.489495000	1.865680000
H	1.867142000	2.711834000	0.614648000
C	5.597764000	3.743466000	-0.052301000
H	5.636244000	1.846780000	0.955229000
H	5.811514000	1.722208000	-0.785652000
C	4.810508000	4.525262000	0.991579000
H	2.736070000	4.890000000	1.528975000
H	3.035244000	4.741105000	-0.194715000
H	6.675626000	3.893078000	0.077817000
H	5.350174000	4.130934000	-1.051469000
H	5.067943000	5.589497000	0.952154000
H	5.092162000	4.175411000	1.996632000
C	3.177884000	-0.266044000	1.687436000
C	2.768548000	-1.739325000	1.802691000
C	4.395136000	0.047932000	2.566113000
H	2.330732000	0.318385000	2.089090000
C	2.583914000	-2.168060000	3.253203000
H	3.534263000	-2.377903000	1.335175000
H	1.844971000	-1.904678000	1.227091000
C	4.174236000	-0.400209000	4.008954000
H	5.288624000	-0.451517000	2.173524000
H	4.609280000	1.121858000	2.547044000
C	3.826804000	-1.880478000	4.083585000
H	2.308620000	-3.229013000	3.304151000
H	1.740642000	-1.608034000	3.683404000
H	5.062678000	-0.175600000	4.610021000
H	3.349870000	0.183807000	4.446367000
H	3.677398000	-2.192559000	5.123206000
H	4.670631000	-2.472950000	3.699468000

TS-7_M06L.log

SCF (M06L) = -2879.86316621
E(SCF)+ZPE(0 K)= -2878.488549
H(298 K) = -2878.409141
G(298 K) = -2878.602351
Lowest Frequency = -501.9330 cm-1

Pd	-0.388782000	0.067498000	-1.024823000
Al	1.887829000	-0.075493000	-0.154339000
N	2.988455000	1.563707000	0.082403000
N	2.860168000	-1.026068000	1.257504000
C	3.302433000	1.923190000	1.327501000
C	3.129739000	1.064966000	2.432771000
H	3.271448000	1.509540000	3.411969000
C	3.040175000	-0.332213000	2.397624000
C	3.936568000	3.255486000	1.601420000
H	3.559707000	4.042506000	0.944195000
H	5.018002000	3.193007000	1.430396000
H	3.787137000	3.546601000	2.642981000
C	3.163145000	-1.069154000	3.699704000
H	3.219752000	-0.374392000	4.539397000
H	4.047200000	-1.714584000	3.719103000
H	2.300517000	-1.729513000	3.844607000
C	3.382700000	2.440687000	-0.985356000
C	4.712392000	2.414309000	-1.446864000
C	5.082463000	3.297720000	-2.464729000
H	6.105153000	3.275270000	-2.836059000
C	4.171491000	4.189351000	-3.011224000
H	4.479279000	4.874402000	-3.796565000
C	2.857223000	4.187255000	-2.560688000

H	2.136993000	4.871396000	-3.002071000
C	2.432614000	3.316136000	-1.554128000
C	5.734663000	1.422044000	-0.931092000
H	5.313300000	0.916549000	-0.052573000
C	6.000088000	0.360294000	-1.999312000
H	5.080737000	-0.172075000	-2.262791000
H	6.739063000	-0.369946000	-1.652721000
H	6.394943000	0.822361000	-2.911595000
C	7.039653000	2.090968000	-0.504740000
H	7.550941000	2.555384000	-1.355141000
H	7.729302000	1.354487000	-0.079001000
H	6.879440000	2.873193000	0.244631000
C	0.984333000	3.314793000	-1.110969000
H	0.744705000	2.305562000	-0.740392000
C	0.022651000	3.596509000	-2.263280000
H	-0.991857000	3.305572000	-1.978585000
H	0.281583000	3.025565000	-3.162068000
H	-0.000523000	4.659004000	-2.537562000
C	0.722351000	4.265786000	0.053422000
H	1.297779000	3.990527000	0.943289000
H	-0.335073000	4.229785000	0.339008000
H	0.972864000	5.301797000	-0.208727000
C	3.071703000	-2.444834000	1.329883000
C	1.999752000	-3.321734000	1.578081000
C	2.272552000	-4.688004000	1.705072000
H	1.447427000	-5.372353000	1.891277000
C	3.565098000	-5.177253000	1.603825000
H	3.760591000	-6.239512000	1.723281000
C	4.608729000	-4.304550000	1.315965000
H	5.612132000	-4.701031000	1.191527000
C	4.385716000	-2.936255000	1.149278000
C	0.564023000	-2.851546000	1.650442000
H	0.556427000	-1.753125000	1.700718000
C	-0.165740000	-3.242995000	0.363780000
H	-0.338529000	-4.327948000	0.323465000
H	0.406474000	-2.952081000	-0.523814000
H	-1.129113000	-2.729144000	0.283972000
C	-0.165140000	-3.382599000	2.882846000
H	-0.345360000	-4.462435000	2.817866000
H	-1.142518000	-2.898909000	2.989318000
H	0.400196000	-3.203463000	3.804956000
C	5.512299000	-2.020787000	0.698174000
H	5.052800000	-1.278956000	0.027347000
C	6.582070000	-2.766216000	-0.094872000
H	7.159349000	-3.446808000	0.540733000
H	7.297946000	-2.058503000	-0.523450000
H	6.154367000	-3.353954000	-0.913112000
C	6.180096000	-1.253799000	1.842066000
H	5.506651000	-0.541419000	2.325179000
H	7.036534000	-0.683063000	1.465169000
H	6.556835000	-1.943652000	2.606918000
Al	-2.617524000	0.166187000	-0.021339000
N	-4.290897000	-0.832139000	-0.465558000
N	-3.576841000	1.137480000	1.398994000
C	-5.521584000	-0.648098000	0.006520000
C	-5.776815000	0.187391000	1.106111000
H	-6.801695000	0.216962000	1.460900000
C	-4.866063000	1.011638000	1.768294000
C	-6.710839000	-1.300532000	-0.641746000
H	-7.391965000	-1.710168000	0.108813000
H	-6.427211000	-2.093399000	-1.335910000
H	-7.275876000	-0.547694000	-1.202979000
C	-5.388736000	1.809958000	2.931257000
H	-4.683280000	1.803899000	3.766809000
H	-6.347871000	1.416074000	3.271212000
H	-5.533622000	2.861840000	2.662223000
C	-3.998682000	-1.817421000	-1.463119000
C	-3.641729000	-1.389163000	-2.756639000
C	-3.113532000	-2.336040000	-3.637781000
H	-2.821894000	-2.025106000	-4.638722000
C	-2.953339000	-3.659921000	-3.253447000
H	-2.524979000	-4.379650000	-3.945861000
C	-3.373484000	-4.076264000	-1.993741000
H	-3.283810000	-5.124385000	-1.722045000
C	-3.909005000	-3.172462000	-1.074248000
C	-3.954841000	0.017838000	-3.233852000
H	-4.100722000	0.650624000	-2.346741000
C	-2.849102000	0.661106000	-4.058753000
H	-2.657419000	0.113017000	-4.988490000
H	-1.918279000	0.702445000	-3.481890000
H	-3.131231000	1.682476000	-4.337582000
C	-5.276141000	0.005673000	-4.007329000
H	-5.563613000	1.018179000	-4.309839000
H	-6.092549000	-0.417090000	-3.412321000
H	-5.187081000	-0.601125000	-4.915799000
C	-4.429675000	-3.630891000	0.281347000
H	-5.359389000	-3.079323000	0.469391000
C	-4.793191000	-5.111320000	0.309701000
H	-5.462499000	-5.385663000	-0.511404000
H	-5.290801000	-5.360854000	1.251517000
H	-3.903141000	-5.746491000	0.241364000
C	-3.493773000	-3.314786000	1.447640000
H	-3.994197000	-3.501362000	2.404551000
H	-3.158165000	-2.269997000	1.449085000
H	-2.601812000	-3.946454000	1.408510000
C	-2.796078000	2.155912000	2.042256000
C	-1.788310000	1.783931000	2.955432000
C	-1.023047000	2.800675000	3.534363000
H	-0.231517000	2.541863000	4.231349000
C	-1.253672000	4.137285000	3.236367000
H	-0.648912000	4.910458000	3.702763000
C	-2.244077000	4.483873000	2.326609000
H	-2.403219000	5.530772000	2.080513000
C	-3.021945000	3.506926000	1.702552000
C	-1.562010000	0.327943000	3.323812000
H	-1.604414000	-0.255198000	2.387206000
C	-0.186811000	0.071564000	3.919763000
H	-0.060183000	0.554897000	4.895971000
H	0.605581000	0.430129000	3.253590000
H	-0.042630000	-1.001238000	4.076184000
C	-2.654183000	-0.208843000	4.250305000
H	-2.435852000	-1.243248000	4.539129000
H	-3.639926000	-0.204449000	3.776384000
H	-2.716095000	0.384858000	5.170221000
C	-4.025729000	3.896971000	0.630987000
H	-4.816802000	3.138920000	0.599109000
C	-3.354708000	3.887373000	-0.742049000
H	-2.558837000	4.640217000	-0.790517000
H	-4.074234000	4.103932000	-1.539570000
H	-2.893491000	2.914219000	-0.957340000
C	-4.699214000	5.240396000	0.890531000
H	-3.997919000	6.075483000	0.789881000
H	-5.133799000	5.294605000	1.894222000
H	-5.500602000	5.410970000	0.165536000
H	1.561644000	1.220501000	-2.690952000
C	1.188517000	0.242383000	-2.387363000
C	0.519367000	-0.668453000	-3.218538000
O	2.616725000	-0.682854000	-1.899121000
C	1.064256000	-1.978874000	-3.024845000
H	0.007461000	-0.376358000	-4.131002000
C	2.215277000	-1.963883000	-2.301324000
H	0.674472000	-2.886055000	-3.475186000
C	3.209714000	-3.050608000	-2.150274000
H	2.853223000	-3.952341000	-2.655092000
H	4.164659000	-2.755891000	-2.607459000
H	3.423952000	-3.314349000	-1.109982000
